# A Genome-Scale Metabolic Model of 2,3-Butanediol Production by Thermophilic Bacteria *Geobacillus icigianus*

**DOI:** 10.3390/microorganisms8071002

**Published:** 2020-07-04

**Authors:** Mikhail Kulyashov, Sergey E. Peltek, Ilya R. Akberdin

**Affiliations:** 1 Biosoft.ru, 630058 Novosibirsk, Russia; m.kulyashov@mail.ru; 2 Department of Natural Sciences, Novosibirsk State University, 630090 Novosibirsk, Russia; 3 Department of Bioinformatics, Federal Research Center for Information and Computational Technologies, 630090 Novosibirsk, Russia; 4 Department of Molecular Biotechnology, Institute of Cytology and Genetics SB RAS, 630090 Novosibirsk, Russia; peltek@bionet.nsc.ru

**Keywords:** genome-scale, metabolic model, 2,3-butanediol, flux balance analysis, *Geobacillus icigianus*

## Abstract

The thermophilic strain of the genus *Geobacillus*, *Geobacillus icigianus* is a promising bacterial chassis for a wide range of biotechnological applications. In this study, we explored the metabolic potential of *Geobacillus icigianus* for the production of 2,3-butanediol (2,3-BTD), one of the cost-effective commodity chemicals. Here we present a genome-scale metabolic model *iMK1321* for *Geobacillus icigianus* constructed using an auto-generating pipeline with consequent thorough manual curation. The model contains 1321 genes and includes 1676 reactions and 1589 metabolites, representing the most-complete and publicly available model of the genus *Geobacillus*. The developed model provides new insights into thermophilic bacterial metabolism and highlights new strategies for biotechnological applications of the strain. Our analysis suggests that *Geobacillus icigianus* has a potential for 2,3-butanediol production from a variety of utilized carbon sources, including glycerine, a common byproduct of biofuel production. We identified a set of solutions for enhancing 2,3-BTD production, including cultivation under anaerobic or microaerophilic conditions and decreasing the TCA flux to succinate via reducing citrate synthase activity. Both in silico predicted metabolic alternatives have been previously experimentally verified for closely related strains including the genus *Bacillus.*

## 1. Introduction

Biological factories designed for the production of bulk chemical and fuels are crucial components of the future manufacturing, focused on eliminating environmental pollution and reduction of fossil fuel and oil price dependencies. 2,3-butanediol (butadiene glycol-2,3 or 2,3-BTD) has a broad list of industrial applications and it represents one of the essential commodity chemicals [1]. The potential of the microbial 2,3-butanediol production was shown in the early 20th century and many bacterial species including genera *Klebsiella, Enterobacter, Bacillus, Lactobacillus* and *Serratia* have been studied so far as microorganisms which are able to synthesize 2,3-BTD [1,2]. While many *Klebsiella spp*. strains are stable under a wide range of growth conditions and possess the highest product yield, the strain biosafety level 2 assignment restricts its biotechnological applications for the industrial-scale 2,3-BTD production. In a search for an alternative producer, a novel aerobic thermophilic *Geobacillus* strain excreting 2,3-BTD has been identified. The thermophilic properties of the strain provide additional cost-effective strategies for microbial fermentation of 2,3-butanediol [3].

The majority of species of the genus *Geobacillus* have optimal growth conditions at temperatures near 50–60 °C that promote extremely high growth and carbon conversion rates [4]. Furthermore, members of the genus *Geobacillus* are capable of utilizing various carbon sources [4,5,6]. Many *Geobacillus* spp. became a source of thermostable proteins and enzymes [4,5]. The strains have been proposed as whole cell biocatalysts for biotechnological applications at elevated temperatures [4,5]. The *Geobacillus icigianus* G1w1T is a new thermophilic strain of the genus *Geobacillus* that was firstly isolated from sludge of the hydrothermal vent located in the Valley of Geysers (Kamchatka, Russia) [6]. Availability of the annotated bacterium’s genome [7] enables a systems-level investigation of the *Geobacillus icigianus* metabolism including whole-genome metabolic reconstruction and flux balance analysis.

A genome-scale metabolic (GSM) modeling approach together with constraint-based flux balance analysis (FBA) have been developed as computational platforms to provide a holistic view of the cellular metabolism in pro- and eukaryotes and predict flux distribution in a global metabolic network both under a range of environmental conditions and genetic perturbations [8]. Herein, we used a genome-scale metabolic modeling approach [9,10] to investigate microbial metabolism and estimate the capability of *Geobacillus icigianus* G1w1T strain to generate and excrete 2,3-BTD under certain growth conditions. Based on the analysis of the developed *iMK1321* model by means of diverse evolutionary optimization algorithms we have found two feasible and alternative ways to enhance 2,3-BTD production from different carbon sources in this particular microorganism. The first mechanism is an operating mode of the culture growth in anaerobic or microaerophilic conditions, while the second one is related to genetic modifications of the TCA cycle leading to the reduction of succinate production.

## 2. Materials and Methods

### 2.1. Model Reconstruction

The complete genome of *Geobacillus icigianus* strain (GenBank assembly accession: GCA_000750005.1) [7] was used for reannotation by means of the standard RAST (Rapid Annotation Subsystem Technology) annotation scheme [11]. The KBase web-tool [12] was employed to generate a draft genome-scale metabolic model of the strain with standard parameters including an in-built gap-filling algorithm [13]. The vast majority of gene-protein-reaction (GPR) associations were added automatically at the process of the model generation. The biomass equation was adapted from a closely-related *Bacillus subtilis* strain [14], since there is no measured biomass composition for *G. icigianus*. SEED [15] reaction names and IDs of the draft model were changed to BiGG Models IDs [16] using an original script on Python 3.6 in Cobrapy [17] to improve the model consistency and make it comparable with other GSM models. The quality of the resulting draft model was checked out using the Memote web-service [18], which demonstrated that the consistency of the developed model is 91% (see Supplementary Materials and https://github.com/mkulyashov/Geobacillus_icigianus_supplementary).

### 2.2. Applied Constraints and Model Curation

To simulate a growth of the strain on glucose as a single carbon source the lower bound of the glucose uptake rate was considered as −16 mmol gDCW^−1^ h^−1^ according to the published data for closely related species [19]. A lower bound of exchange reactions for components of the Pfennig medium (NH3^+^, PO4^3−^, Mg^2+^, Ca^2+^, K^+^ and Na^+^) was set to −1000 mmol gDCW^−1^ h^−1^, while the upper bound was set as +1000 mmol gDCW^−1^ h^−1^ for them. The upper bound for the majority of cellular metabolic reactions was assumed to be +1000 mmol gDCW^−1^ h^−1^, unless stated otherwise. The lower bounds of the reversible and irreversible cellular metabolic reactions were set as −1000 mmol gDCW^−1^ h^−1^ and 0 mmol gDCW^−1^ h^−1^, respectively. 

A set of carbon source exchange reactions were added for substrates known to support the growth of *Geobacillus* spp: glycerine, L-arabinose and D-xylose [6]. The presence of all corresponding enzymatic pathways in the *Geobacillus icigianus* genome was confirmed by BLASTP alignment [20] (see Supplementary Materials and https://github.com/mkulyashov/Geobacillus_icigianus_supplementary), while GPR for ribulose-5-phosphate 4-epimerase (EC: 5.1.3.4) was based on the BioCyc annotation [21] for *G.icigianus*. Additionally, the ability to grow on xylose as a single carbon source was experimentally shown [6] and predicted from the genome-based metabolic reconstruction, previously published information for related *Geobacillus species* and similarity of the pathway to the metabolic pathways of *Bacillus subtilis* in SEED, KEGG [22] and BIGG databases. The BLASTP analysis identified D-xylose-specific transporter via ABC system for *G.icigianus* as well. The uptake rates for other carbon sources (glycerine, L-arabinose and D-xylose) were proportionally calculated on the basis of the number of carbon molecules regarding the glucose since these rates were not measured in experiments yet. 

### 2.3. Model Modification for 2,3-Butanediol Production

Reactions required for 2,3-butanediol production were manually evaluated. The metabolic routes for 2,3-BTD production to synthesize the substance comprises: acetolactate synthase (EC:2.2.1.6), acetolactate decarboxylase (EC: 4.1.1.5) and (R,R)-butanediol dehydrogenase (EC:1.1.1.4). Only acetolactate synthase was originally presented in the draft model, the newly identified metabolic reactions were added using Cobrapy. It is noteworthy that the BLASTP analysis demonstrated that acetolactate decarboxylase (EC: 4.1.1.5) is not encoded in *Geobacillus icigianus* genome confirming the previous finding that this enzyme is absent in all thermophilic microorganisms [23]. However, the production of 2,3-BTD was shown for other thermophilic organisms and for the genus *Geobacillus* too [3]. Furthermore, it has been demonstrated that the first protein of the pathway, acetolactate synthase of *B.subtilis* is also capable to catalyze the decarboxylation of 2-ketoisovalerate in the isobutanol production pathway [24]. Given the similarity of these pathways (isobutanol production and 2.3-BTD biosynthesis), it has been assumed that acetolactate synthase may catalyze the second reaction in *Geobacillus icigianus* as well. (R,R)-butanediol transport and (R,R)-butanediol exchange reactions were also added similarly to the *i*YO844 model [14]. The final version of the GSM model was uploaded into the Memote web-service which demonstrated that the overall score of the model did not change compared to the draft model and equals 91% indicating the model quality and ensuring its applicability for further studies. 

### 2.4. Flux Balance Analysis

The maximization of the biomass equation was used as an objective function of the model for parsimonious flux balance analysis (pFBA) which attempts to minimize overall cellular flux while maximizing the growth rate [25]. Optflux tool [26] was harnessed to perform in silico simulations in order to predict both growth rates for different carbon sources and production rate of 2,3-BTD. The Escher web-tool was used for the visualization of the central metabolic pathways including glycolysis, pentose phosphate pathway, TCA cycle, biochemical reactions of the oxidative phosphorylation and their relevant fluxes [27]. The model file in json and sbml formats as well as flux distribution map are available online at https://github.com/mkulyashov/Geobacillus_icigianus_supplementary/tree/master/model_files. The model is also available online at BioUML web-service (https://ict.biouml.org/bioumlweb/#de=data/Collaboration/FBA%20models/) [28]. The list of reactions and metabolites of the developed model as well as flux distribution on the metabolic map for different carbon sources (glucose, glycerine, xylose and arabinose) are presented in the Supplementary Materials in Excel format and can be also downloaded from corresponding folder on github.

### 2.5. Model Analysis for 2,3-Butanediol Production Optimization

To identify genetic modifications which are essential to enhance 2,3-butanediol production a Biomass-Product Coupled Yield (BPCY) as the objective function of an evolutionary optimization approach has been used in the Optflux tool. To conduct this type of analysis, the following simulation algorithms have been selected: pFBA, Minimization of Metabolites Balance (MiMBL) [29], Linear implementation of Minimization Of Metabolic Adjustment (LMOMA) [30]. All algorithms were started with 5000 maximum evolutionary functions and with the maximum number of modifications equal to 2. The optimization algorithm was chosen considering specific options of simulation methods. LMOMA and pFBA simulation methods as well as the MiMBL algorithm were run with default options for Strength Pareto Evolutionary Algorithm 2 (SPEA2). 

## 3. Results

### 3.1. Model Reconstruction

The genome-scale metabolic model was reconstructed using a semi-automatic pipeline including genome annotation with RAST [11] and subsequent generation via Kbase methodology [12]. The developed model contains 1676 reactions, 1589 metabolites and 1321 genes. According to recently published phylogenetic re-annotation of the genus *Geobacillus, G.icigianus* belongs to the Clade 1 while *G. thermoglucosidasius* strains are included in the second clade [31]. Although the almost 200 genome assemblies are available at NCBI for the genus *Geobacillus*, there are only a few published GSM models for this genus. Table 1 demonstrates the model statistics compared to the previously published genome-scale metabolic models of other strains Furthermore, analysis and verification of the model quality by the Memote service (https://memote.io/, [18]), the current standard tool for GSM models verification and comprehensive overview [32,33,34], demonstrates the highest level of the GSM model consistency and completeness (see Supplementary Files: MemoteReportInitialModel.html and MemoteReportFinalModel.html and at github).

**Figure 1:**
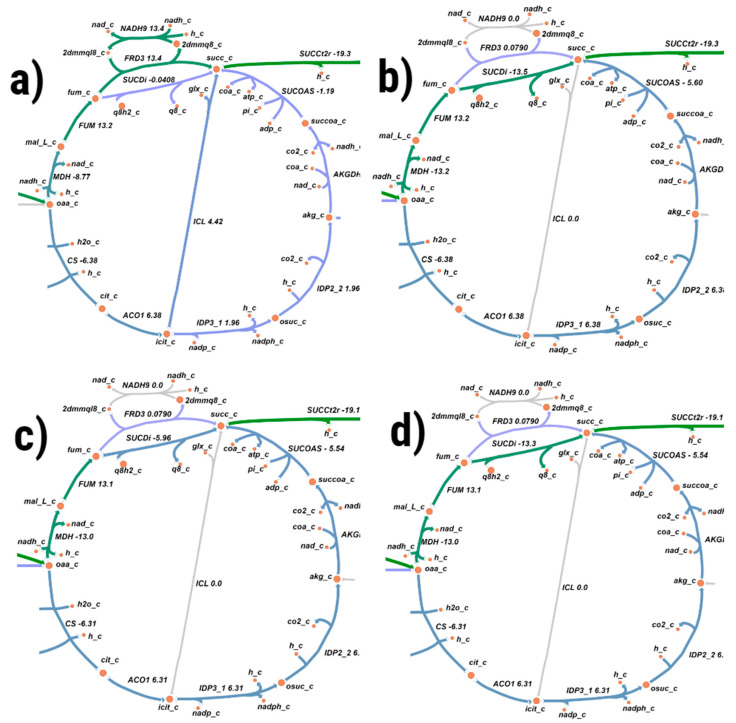
The flux distribution maps for TCA cycle predicted by *iMK1321* for growth of *Geobacillus icigianus* on: (**a**) glucose, (**b**) glycerine, (**c**) xylose, (**d**) arabinose. The maps were drawn in Escher web-tool, where the circles represent metabolites, while the arrows refer to reactions [27]. The flux value through the reaction is reflected by colour, where numbers represent corresponding range of the reaction fluxes in mmol gDCW^−1^ h^−1^: purple—0–1; blue—5–10; green—10–30; red—30–80 mmol gDCW^−1^ h^−1^.

A manual curation of the model enabled us to enhance the number of gene-protein-reaction associations (only 56 enzymatic reactions without assigned genes, while the total number of enzymatic reactions is 1405) and incorporate reactions required for uptake and initial steps of metabolic conversion of diverse carbon sources as well as a set of metabolic reactions for 2,3-BTD production. Parsimonious flux balance analysis was conducted via the Optflux software [26]. Figure 1 represents predicted flux distribution for different carbon sources on the metabolic network for TCA cycle reconstructed via the Escher web-tool ([27], detailed figures of the metabolic network for different carbon sources are provided as Supplemental Figures S1–S4). 

The observed distribution of fluxes through *G. icigianus* metabolic map differs from the *B. subtilis* flux distribution, despite the fact that the *B. subtilis* metabolic pathways and biomass equation were used as a template for our draft model. For instance, *G. icigianus* mainly uses FRD3 reaction catalyzed by fumarate reductase as a reaction in the electron transfer system, when glucose is a carbon source and SUCDi reaction for other carbon sources, while *B. subtilis* employs SUCDi and NADH4 reactions. Furthermore, a metabolic reaction catalyzed by 6-phosphogluconolactonase (PGL, EC:3.1.1.31) is absent in the pentose phosphate pathway of *G.icigianus* according to the reconstructed *iMK1321* model, while this reaction is presented in *iYO844* model for *B.subtilis* loaded from BIGG database (http://bigg.ucsd.edu/models/iYO844). Seemingly, it is due to the evolutionary elimination of the gene encoding 6-phosphogluconolactonase in the genus *Geobacillus* since an experimental verification indicated that this reaction can spontaneously proceed at room temperature and the reaction rate might be sufficient in thermophilic microorganisms for the functionality of the pentose phosphate pathway [4]. It was also experimentally demonstrated that some *Geobacillus* spp. may have an alternative oxidation route for the production of ribulose-5-phosphate via reactions catalyzed by 6-phospho-3-hexuloisomerase and 3-hexulose-6-phosphate synthase [4]. However, these enzymes were not identified by the BLASTP analysis in the *G.icigianus* genome (see Supplementary Materials and https://github.com/mkulyashov/Geobacillus_icigianus_supplementary). Further analysis of the flux distribution for different carbon sources shows that lactate and succinate are excreted compounds during the growth on xylose, glucose and arabinose and it is consistent with published experimental data [6]. pFBA predicts glucose and glycerine as the most promising substrates for active growth of *G.icigianus* (0.5 mmol gDCWl^−1^ h^−1^), but growth on glycerine requires more oxygen (Table 2 and corresponding Supplementary Tables for *G.icigianus*). A comparative analysis of the pFBA predictions (Table 2) conducted for *iMK1321* model and published GSM model of *Bacillus subtilis* (*iYO844*, [14]) demonstrates significantly lower growth rate for *B.subtilis* if a glucose uptake rate corresponds to the used one in the original model (−1.7 mmol gDCWl^−1^ h^−1^) and slightly enhanced growth rate for *B.subtilis* comparing to *G.icigianus* if the glucose consumption rate equals to the used one in *iMK1321* model (−16 mmol gDCWl^−1^ h^−1^) experimentally measured for *Geobacillus thermoglucosidasius* NCIMB 11955 [19]. It is noteworthy that the high level of substrate consumption has never been experimentally demonstrated for *Bacillus subtilis* while all published growth rates for strains of the genus *Geobacillus* are much higher than the estimation for *B.subtilis* [35,36].

### 3.2. iMK1321 Model Optimization for 2,3-Butanediol Production

Since the genome-scale metabolic modeling provides a phenotype prediction based on the studied organism genotype, this approach is a powerful computational tool for rational strain optimization within such a booming field as metabolic engineering. To develop a strategy for achievement of desired cellular behaviour using genetic manipulations, a myriad of in silico optimization methods have been proposed [37]. Herein, we used a set of optimization algorithms implemented in the Optflux tool [38]: the parsimonious enzyme usage FBA (pFBA), a Linear implementation of Minimization Of Metabolic Adjustment method (LMOMA) and a more recent Minimization of Metabolites Balance (MiMBl) approach in order to identify genetic mutations required for more effective 2,3-BTD production by *G.icigianus* and take into account the various strain optimization strategies that may predict different routes for metabolic engineering of the strain. 

The LMOMA algorithm was able to discover 78 solutions for growth on glucose; 90 solutions for growth on both glycerine and xylose; and 72 solutions for growth where arabinose is a carbon source. Optimization results with the most benefit for 2,3-BTD production are presented in Table 3, and show that reactions, which mostly require modifications are oxygen transport and citrate synthase (CS). It should be noted that reduction of the flux via CS, which was revealed by the LMOMA algorithm as one of the best solutions for 2,3-BTD production, was recently experimentally demonstrated by using a weak promoter for the gene encoding CS to increase the production of (3R)-acetoin, a precursor of 2,3-BTD in *Corynebacterium glutamicum* [39]. Some other reactions, which are predicted by this method for enhanced 2,3-BTD biosynthesis, are also related to TCA cycle and succinate production.

Strain optimization to increase the level of 2,3-butanediol produced by the cell using the MiMBL algorithm predicts 43 optimal solutions for glucose, 83 solutions for xylose, 100 solutions for arabinose and 26 solutions for glycerine, as substrates for bacterial growth. The best optimization results for this algorithm are presented in Table 4, and demonstrate that the most common reactions are catalyzed by a and b aconitases (ACONTa, ACONTb), 1–3 fructose bisphosphate aldolases (FBA, FBA2, FBA3), citrate hydroxy mutase (ACO1) are related to the oxygen transportation. Although the reactions predicted by the MiMBL as required for modification mostly differ from the list of reactions which were identified by the LMOMA, all of them are associated with TCA cycle and succinate production. Cultivation of the strain in anaerobic/microaerobic conditions predicted by the method as a way to increase the accumulation of 2,3-BTD, corresponds to LMOMA results.

The pFBA algorithm was able to identify a significantly lower number of optimal solutions for 2,3-BTD production: 1 solution for cells grown on glucose, arabinose and glycerine and 2 solutions for growth on xylose Table 5. Apparently, the algorithm is not effective to predict optimal genetic manipulations for 2,3- BTD production. However, identified solutions via oxygen transport reactions coincide with predictions of two above-mentioned optimization methods.

Thus, in silico optimization algorithms indicate two possibilities to enhance the production of 2,3-BTD by the strain including genetic modifications of the TCA cycle which lead to decrease in succinate production and growth of *G.icigianus* in oxygen-limited conditions.

**Figure 2:**
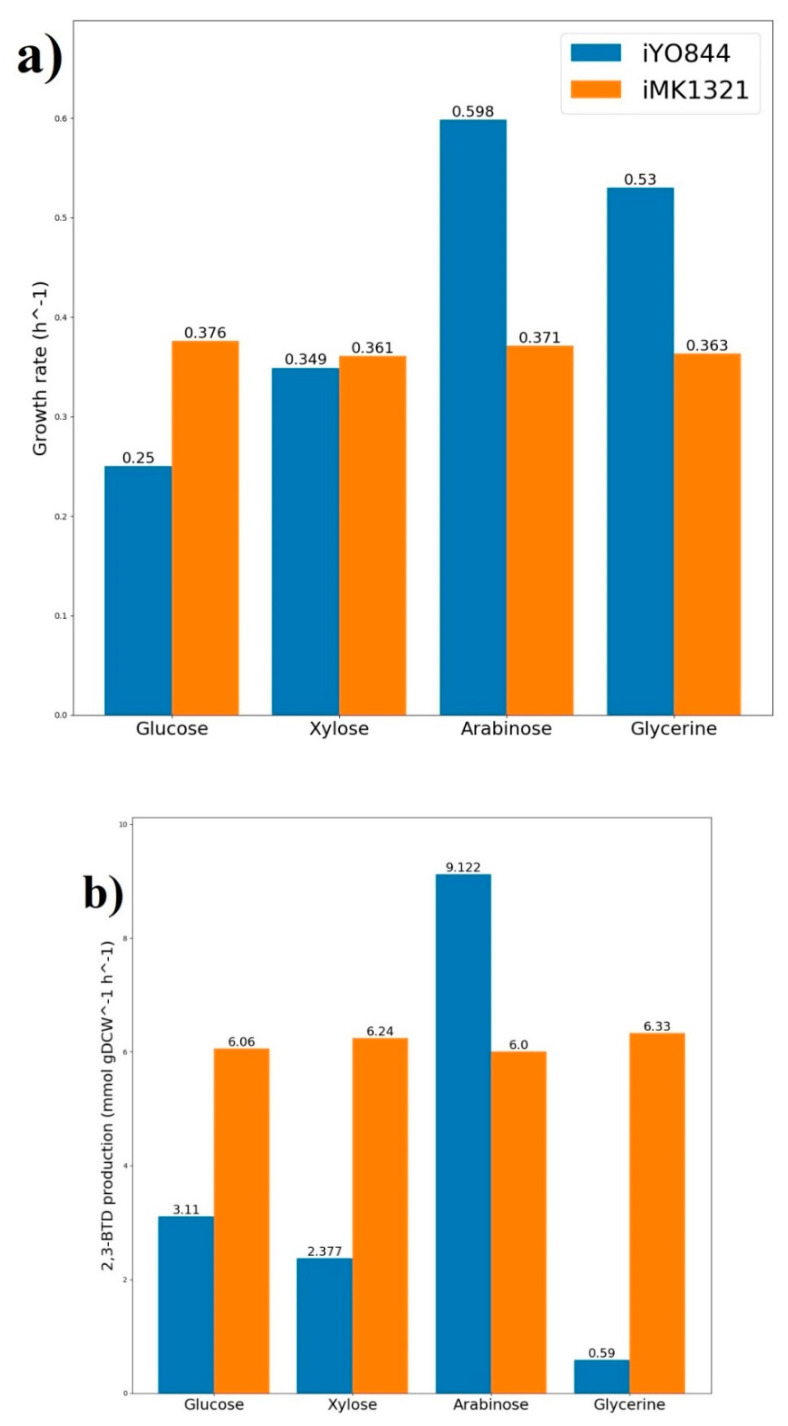
Comparison of 2,3-butanediol production with the LMOMA algorithm between *iMK1321* (orange) and *iYO844* (blue) models on different carbon sources: (**a**) growth rate (**b**) 2,3-butanediol production rate.

In addition to that, a computational optimization of 2,3-butanediol production by *Bacillus subtilis* strain has been carried out with the LMOMA algorithm. Results of the optimization on different substrates demonstrate that *Geobacillus icigianus* gives more effective production of 2,3-BTD in most cases. However, *Bacilus subtilis* can be a more suitable microorganism for 2,3-BTD biosynthesis if arabinose is a carbon source for the growth Figure 2 that is based on the availability of two arabinose transporter systems in the bacterium. Flux balance analysis predicts that the arabinose transporter via proton symport is the only transport system for growth on arabinose, while a maximization of 2,3-BTD production with simultaneous growth requires more carbon source uptake leading to the activation of arabinose ABC transport system. As can be seen from the Figure 2, *G. icigianus* has the highest level of 2,3-butanediol production on glycerine-dependent growth. The outcome proposes the biotechnological potential of the strain in conversion of the industrial by-product into value-added target compounds.

To verify the model predictions a thorough analysis of the published experimental data on mechanisms of strain improvements for accumulation of 2,3-BTD including closely related species has been undertaken. A transition of the bacterial growth to microaerobic or anaerobic conditions is one of the common experimental approaches to increase the production of 2,3-BTD in many biotechnological species [40,41,42]. Furthermore, a new avenue via the oxidative TCA cycle for metabolic engineering of *Escherichia coli* was revealed to increase the production of another related commodity chemical, 1,4-butanediol [41]. Data from the literature support the predictive power of the developed *iMK1321* genome-scale metabolic model.

## 4. Discussion

With the development of industrial biotechnology, thermophilic microorganisms are considered as a unique and attractive platform in whole cell applications. In this study, a genome scale metabolic model, *iMK1321* for a recently isolated thermophilic strain of the genus *Geobacillus*, *Geobacillus icigianus* has been reconstructed. The model has been built using Kbase web-service with consequent manual curation. The final version of the model represents the most comprehensive GSM reconstruction developed for this genus, enabling in silico interrogation of the bacterial growth on diverse substrates including glucose, glycerine, xylose and arabinose. Computational analysis of the model indicates glycerine and glucose as the most suitable carbon sources for *G.icigianus* cultivation. To validate the model predictions, continuous culture cultivation of the strain on different carbon sources is planned to be conducted in future studies. Furthermore, a comparative analysis of the model simulations with distribution of the fluxes predicted by the *iYO844* model for closely related *Bacillus subtilis* demonstrates some differences in metabolic rearrangements between these microbial systems. The developed model has also been employed to predict optimal solutions for enhanced 2,3-butanediol production by this particular thermophilic strain. In silico predictions based on different optimization algorithms to accelerate biosynthesis of 2,3-BTD comprise genetic modifications related to some steps of the TCA cycle which result in a decrease in succinate production and cultivation of the strain in oxygen-limited conditions. Identified strategies of metabolic engineering for *G.icigianus* have been already experimentally demonstrated as capabilities for active 2,3-BTD biosynthesis in closely related species [39,40,42,43,44,45], thereby affirming a benefit of the computational modeling in biotechnological strain design.

## Figures and Tables

**Table 1:** Network statistics for published genome-scale models of *Geobacillus* strains and comparison with *iMK1321*.

Species	# of Genes in the Model	# of Reactions in the Model	# of Metabolites in the Model	Genome Size and RefSeq ID	# of Protein-Coding Genes	Reference
*Geobacillus thermoglucosidasius* (C56-YS93)	736	1159	1163	NZ_CP012712.1 Size = 3.87 Mb	3659	[35]
*Geobacillus thermoglucosidasius* (NCIMB 11955)	859	1011	1050	NZ_CP012712.1 Size = 3.87 Mb	3615	[19]
*Geobacillus icigianus* (G1W1)	1321	1676	1589	NZ_JPYA00000000.1 Size = 3.46 Mb	3183	this study

**Table 2:** Comparison of parsimonious flux balance analysis (pFBA) predictions for *iMK1321* and *i*YO844 models on different substrates.

Organism	Growth Rate on Glucose (h^−1^)	Growth Rate on Xylose (h^−1^)	Growth Rate on Arabinose (h^−1^)	Growth Rate on Glycerine (h^−1^)
*B.subtilis* *	0.118	0.113	0.128	0.140
*G.icigianus*	0.502	0.497	0.497	0.502
*B.subtilis* **	0.624	0.624	0.624	0.624

* with glucose rate −1.7 mmol gDCW^−1^ h^−1^ [14]; ** glucose uptake rate is the same as for *G.icigianus* and equals to −16 mmol gDCW^−1^ h^−1^ [19].

**Table 3:** The most effective optimization results of iMK1321 model for 2,3-butanediol production with the Linear implementation of Minimization Of Metabolic Adjustment (LMOMA) algorithm.

Substrate	Production of 2,3-Butanediol (mmol gDCW^−1^ h^−1^)	Growth Rate (h^−1^)	Reaction Modifications *
Glucose	6.06	0.376	CS = 0.03125
			GLYO1 = 0.03125
Glucose	6.06	0.376	CS = 0.03125
			R00014 = 0.03125
Glucose	4.14	0.4136	O2t = 0.03125
Arabinose	6.00	0.371	CS = 0.03125
Arabinose	4.64	0.4	CS = 0.25
Xylose	6.24	0.3612	CS = 0.03125
			ALKP = 0.03125
Xylose	6.24	0.3612	CS = 0.03125
			R01440 = 0.125
Xylose	6.14	0.3613	CS = 0.03125
			SUCCt2r = 0.03125
Glycerine	6.33	0.363	EX_o2_e = 0.5
			GF6PTA = 32.0
Glycerine	6.33	0.363	EX_o2_e = 0.5

* Value less than 1—a decrease in the flux value through the reaction compared to wildtype. A value greater than 1 is an increase in the flux value through the reaction compared to the wildtype. CS—citrate synthase; GLYO1—glycine oxidase; O2t—oxygen transport into cell; ALKP—alkaline phosphatase; R01440—formaldehyde glycolaldehyde transferase; SUCCt2r—transport of succinate from the extracellular space into the cell; EX_o2_e-exchange oxygen reaction; R00014—thiamine diphosphate acetaldehyde.

**Table 4:** Optimization results of *iMK1321* model for 2,3-butanediol production with the MiMBL algorithm.

Substrate	Production of 2,3-Butanediol (mmol gDCW^−1^ h^−1^)	Growth Rate (h^−1^)	Genetic Modifications **	Reaction Modifications *
Glucose	5.69	0.32	EP10_15190 = 0.03125	ACO1 = 0.03125 ACONTb = 0.03125 ACONTa = 0.03125
			EP10_02485 = 0.03125	FEROpp = 0.03125 R00092 = 0.03125
Glucose	5.17	0.293	EP10_09595 = 0.03125	R0467 = 0.515625 R00014 = 0.7578125 FEROpp = 0.03125 R03050 = 0.515625
			EP10_15190 = 0.03125	ACO1 = 0.03125 ACONTa = 0.03125 ACONTb = 0.03125
Arabinose	6.32	0.35	KFX31147.1 = 16.0	URIDK3 = 8.5 URA6_1 = 4.75
			KFX31511,1 = 0.03125	ACO1 = 0.03125 ACONTa = 0.03125 ACONTb = 0.03125
Arabinose	6.30	0.35	EP10_02065 = 0.03125	GDH2 = 0.03125 GLUDy = 0.03125
			KFX31511.1 = 0.03125	ACO1 = 0.03125 ACONTa = 0.03125 ACONTb = 0.03125
Xylose	5.95	0.34	EP10_12945 = 4.0	PGM = 2.5
			EP10_15190 = 0.03125	ACO1 = 0.03125 ACONTa = 0.03125 ACONTb = 0.03125
Xylose	5.95	0.34	EP10_12945 = 4.0	PGM = 2.5
			KFX31511.1 = 0.03125	ACO1 = 0.03125 ACONTa = 0.03125 ACONTb = 0.03125
Glycerine	6.55	0.357	EP10_13815 = 0.03125	FBA = 0.03125 FBA2 = 0.03125 FBA3 = 0.03125
			EP10_15190 = 0.03125	ACO1 = 0.03125 ACONTa = 0.03125 ACONTb = 0.03125
Glycerine	6.55	0.357	EP10_13815 = 0.0625	FBA = 0.0625 FBA2 = 0.0625 FBA3 = 0.0625
			EP10_15190 = 0.03125	ACO1 = 0.03125 ACONTa = 0.03125 ACONTb = 0.03125
Glycerine	6.46	0.359	EP10_10240 = 0.03125	R01056 = 0.03125
			EP10_15190 = 0.03125	ACO1 = 0.03125 ACONTa = 0.03125 ACONTb = 0.03125

* Value less than 1—a decrease in the flux value through the reaction compared to wildtype. ** Value less than 1 is a gene knockout. while a value greater than 1 is a gene overexpression. A value greater than 1 is an increase in the flux value through the reaction compared to the wildtype. FEROpp—ferrooxidase; ACO1—Hydroxymutase Citrate; ACONTa—aconitase (citrate hydro-lyase); ACONTb—aconitase (hydrolysis isocitrate); R00092—Fe2 + NAD + oxidoreductase; R0467—(S)-2- Aceto-2-hydroxybutanoate pyruvate lyase; R03050—2-Acetolactate pyruvate lyase; R00014—Thiamine diphosphate, acetaldehyde transferase; URIDK3—dUMP phosphotransferase; URA6_1—UMP phosphotransferase; GDH2—l-Glutamate NAD + oxidoreductase; GLUDy—l-Glutamate dehydrogenase; PGM—phosphoglyceratmutase; FBA—fructose bisphosphate aldolase; FBA2-D—Fructose 1-phosphate D-glyceraldehyde 3-phosphate lyase; FBA3—Sedoheptulose-1,7-bisphosphate D—glyceraldehyde-3-phosphate lyase; R01056-D—ribose-5-phosphate-aldose-ketose-isomerase.

**Table 5:** Optimization results of *Geobacillus icigianus* model for 2,3-butanediol production with the pFBA algorithm.

Substrate	Production of 2,3-Butanediol (mmol gDCW^−1^ h^−1^)	Growth Rate (h^−1^)	Reaction Modifications *
Glucose	4.16	0.42	EX_o2_e = 0.03125
Arabinose	4.11	0.41	O2t = 0.03125
Xylose	4.12	0.41	O2t = 0.03125
			FE3abc = 0.03125
Xylose	4.11	0.41	O2t = 0.03125
Glycerine	12.23	0.25	O2t = 0.0625

* Value less than 1—a decrease in the flux value through the reaction compared to wildtype. A value greater than 1 is an increase in the flux value through the reaction compared to the wildtype. O2t—oxygen transport from the extracellular space into the cell; EX_o2_e—exchange reaction for oxygen; FE3abc—ATP phosphohydrolase (iron ion transport).

## Supplementary Materials

The Supplementary materials are available online at https://www.mdpi.com/2076-2607/8/7/1002/s1. The program code used for model extension and manual curation as well as other supplementary materials are available online at https://github.com/mkulyashov/Geobacillus_icigianus_supplementary.

## References

[1] Ji X.-J., Huang H., Ouyang P.-K. (2011). Microbial 2,3-butanediol production: A state-of-the-art review. Biotechnol. Adv..

[2] Celińska E., Grajek W. (2009). Biotechnological production of 2,3-butanediol—Current state and prospects. Biotechnol. Adv..

[3] Xiao Z., Wang X., Huang Y., Huo F., Zhu X., Xi L., Lu J.R. (2012). Thermophilic fermentation of acetoin and 2,3-butanediol by a novel Geobacillus strain. Biotechnol. Biofuels.

[4] Hussein A.H., Lisowska B.K., Leak D.J. (2015). The Genus *Geobacillus* and Their Biotechnological Potential. Advances in Applied Microbiology.

[5] Suzuki H. (2018). Peculiarities and biotechnological potential of environmental adaptation by Geobacillus species. Appl. Microbiol. Biotechnol..

[6] Bryanskaya A.V., Rozanov A.S., Slynko N.M., Shekhovtsov S.V., Peltek S.E. (2015). Geobacillus icigianus sp. nov., a thermophilic bacterium isolated from a hot spring. Int. J. Syst. Evol. Microbiol..

[7] Bryanskaya A.V., Rozanov A.S., Logacheva M.D., Kotenko A.V., Peltek S.E. (2014). Draft Genome Sequence of Geobacillus icigianus Strain G1w1T Isolated from Hot Springs in the Valley of Geysers, Kamchatka (Russian Federation). Genome Announc..

[8] Orth J.D., Thiele I., Palsson B.Ø. (2010). What is flux balance analysis?. Nat. Biotechnol..

[9] Simeonidis V., Price N.D. (2015). Genome-scale modeling for metabolic engineering. J. Ind. Microbiol. Biotechnol..

[10] Gu C., Kim G.B., Kim W.J., Kim H.U., Lee S.Y. (2019). Current status and applications of genome-scale metabolic models. Genome Biol..

[11] Aziz R.K., Bartels D., Best A.A., DeJongh M., Disz T., Edwards R.A., Formsma K., Gerdes S., Glass E.M., Kubal M. (2008). The RAST Server: Rapid Annotations using Subsystems Technology. BMC Genom..

[12] Arkin A.P., Cottingham R.W., Henry C.S., Harris N.L., Stevens R.L., Maslov S., Dehal P., Ware D., Perez F., Canon S. (2018). KBase: The United States Department of Energy Systems Biology Knowledgebase. Nat. Biotechnol..

[13] Latendresse M. (2014). Efficiently gap-filling reaction networks. BMC Bioinform..

[14] Oh Y.-K., Palsson B., Park S.M., Schilling C.H., Mahadevan R. (2007). Genome-scale Reconstruction of Metabolic Network inBacillus subtilisBased on High-throughput Phenotyping and Gene Essentiality Data. J. Biol. Chem..

[15] Devoid S., Overbeek R., DeJongh M., Vonstein V., Best A.A., Henry C. (2013). Automated genome annotation and metabolic model reconstruction in the SEED and model SEED. Systems Metabolic Engineering.

[16] Norsigian C.J., Pusarla N., McConn J.L., Yurkovich J.T., Dräger A., Palsson B., King Z. (2020). BiGG Models 2020: Multi-strain genome-scale models and expansion across the phylogenetic tree. Nucleic Acids Res..

[17] Ebrahim A., Lerman J., Palsson B., Hyduke D.R. (2013). COBRApy: COnstraints-Based Reconstruction and Analysis for Python. BMC Syst. Biol..

[18] Lieven C., Beber M.E., Olivier B.G., Bergmann F.T., Ataman M., Babaei P., Bartell J.A., Blank L.M., Chauhan S., Correia K. (2020). MEMOTE for standardized genome-scale metabolic model testing. Nat. Biotechnol..

[19] Lisowska B. (2016). Genomic analysis and metabolic modelling of Geobacillus Thermoglucosidasius NCIBM 11955. Ph.D. Thesis.

[20] Altschul S.F., Gish W., Miller W., Myers E.W., Lipman D.J. (1990). Basic local alignment search tool. J. Mol. Biol..

[21] Karp P.D., Billington R., Caspi R., Fulcher C.A., Latendresse M., Kothari A., Keseler I.M., Krummenacker M., Midford P.E., Ong Q. (2019). The BioCyc collection of microbial genomes and metabolic pathways. Briefings Bioinform..

[22] Kanehisa M. (2002). The KEGG database. Novartis Foundation Symposium.

[23] Jia X., Liu Y., Han Y. (2017). A thermophilic cell-free cascade enzymatic reaction for acetoin synthesis from pyruvate. Sci. Rep..

[24] Atsumi S., Li Z., Liao J.C. (2009). Acetolactate Synthase from Bacillus subtilis Serves as a 2-Ketoisovalerate Decarboxylase for Isobutanol Biosynthesis in Escherichia coli. Appl. Environ. Microbiol..

[25] Lewis N.E., Hixson K.K., Conrad T.M., Lerman J.A., Charusanti P., Polpitiya A.D., Adkins J.N., Schramm G., O Purvine S., Lopez-Ferrer D. (2010). Omic data from evolved E. coli are consistent with computed optimal growth from genome-scale models. Mol. Syst. Biol..

[26] Rocha I., Maia P., Evangelista P.T., Vilaça P., Soares S., Pinto J.P.B.G.P., Nielsen J., Patil K.R., Ferreira E.C., Rocha M. (2010). OptFlux: An open-source software platform for in silico metabolic engineering. BMC Syst. Biol..

[27] King Z.A., Dräger A., Ebrahim A., Sonnenschein N., E Lewis N., Palsson B. (2015). Escher: A Web Application for Building, Sharing, and Embedding Data-Rich Visualizations of Biological Pathways. PLoS Comput. Biol..

[28] Kolpakov F., Akberdin I., Kashapov T., Kiselev L., Kolmykov S.K., Kondrakhin Y., Kutumova E., Mandrik N., Pintus S., Ryabova A. (2019). BioUML: An integrated environment for systems biology and collaborative analysis of biomedical data. Nucleic Acids Res..

[29] Brochado A.R., Andrejev S., Maranas C., Patil K.R. (2012). Impact of Stoichiometry Representation on Simulation of Genotype-Phenotype Relationships in Metabolic Networks. PLoS Comput. Biol..

[30] Becker S.A., Feist A.M., Mo M.L., Hannum G., Palsson B., Herrgard M.J. (2007). Quantitative prediction of cellular metabolism with constraint-based models: The COBRA Toolbox. Nat. Protoc..

[31] Aliyu H., Lebre P., Blom J., Cowan D., De Maayer P. (2016). Phylogenomic re-assessment of the thermophilic genus Geobacillus. Syst. Appl. Microbiol..

[32] López-Agudelo V.A., Mendum T.A., Laing E., Wu H., Baena A., Barrera L.F., Beste D.J.V., Rios-Estepa R. (2020). A systematic evaluation of Mycobacterium tuberculosis Genome-Scale Metabolic Networks. PLoS Comput. Biol..

[33] Robinson J.L., Kocabaş P., Wang H., Cholley P.-E., Cook D.J., Nilsson A., Anton M., Ferreira R., Domenzain I., Billa V. (2020). An atlas of human metabolism. Sci. Signal..

[34] Nogales J., Mueller J., Gudmundsson S., Canalejo F.J., Duque E., Monk J., Feist A.M., Ramos J.L., Niu W., Palsson B.O. (2019). High-quality genome-scale metabolic modelling of Pseudomonas putida highlights its broad metabolic capabilities. Environ. Microbiol..

[35] Ahmad A., Hartman H.B., Krishnakumar S., Fell D., Poolman M., Srivastava S. (2017). A Genome Scale Model of Geobacillus thermoglucosidasius (C56-YS93) reveals its biotechnological potential on rice straw hydrolysate. J. Biotechnol..

[36] Cordova L.T., Long C., Venkataramanan K.P., Antoniewicz M.R. (2015). Complete genome sequence, metabolic model construction and phenotypic characterization of Geobacillus LC300, an extremely thermophilic, fast growing, xylose-utilizing bacterium. Metab. Eng..

[37] Maia P., Rocha I., Rocha M. An integrated framework for strain optimization. Proceedings of the 2013 IEEE Congress on Evolutionary Computation.

[38] Vilaça P., Maia P., Giesteira H., Rocha I., Rocha M. (2018). Analyzing and designing cell factories with OptFlux. Methods in Molecular Biology.

[39] Lu L., Mao Y., Kou M., Cui Z., Jin B., Chang Z., Wang Z., Ma H., Chen T. (2020). Engineering central pathways for industrial-level (3R)-acetoin biosynthesis in Corynebacterium glutamicum. Microb. Cell Factories.

[40] Song C.W., Park J.M., Chung S.C., Lee S.Y., Song H. (2019). Microbial production of 2,3-butanediol for industrial applications. J. Ind. Microbiol. Biotechnol..

[41] Yim H., Haselbeck R., Niu W., Pujol-Baxley C., Burgard A., Boldt J., Khandurina J., Trawick J.D., E Osterhout R., Stephen R. (2011). Metabolic engineering of Escherichia coli for direct production of 1,4-butanediol. Nat. Methods.

[42] Biswas R., Yamaoka M., Nakayama H., Kondo T., Yoshida K.-I., Bisaria V.S., Kondo A. (2012). Enhanced production of 2,3-butanediol by engineered Bacillus subtilis. Appl. Microbiol. Biotechnol..

[43] Li L., Zhang L., Li K., Wang Y., Gao C., Han B., Ma C., Xu P. (2013). A newly isolated Bacillus licheniformis strain thermophilically produces 2,3-butanediol, a platform and fuel bio-chemical. Biotechnol. Biofuels.

[44] Xiao Z., Lu J.R. (2014). Strategies for enhancing fermentative production of acetoin: A review. Biotechnol. Adv..

[45] Nguyen A.D., Hwang I.Y., Lee O.K., Kim D., Kalyuzhnaya M.G., Mariyana R., Hadiyati S., Kim M.S., Lee E.Y. (2018). Systematic metabolic engineering of Methylomicrobium alcaliphilum 20Z for 2,3-butanediol production from methane. Metab. Eng..

