# Supplementary material for: A Genome-Scale Metabolic Model of 2,3-Butanediol Production by Thermophilic Bacteria Geobacillus icigianus
Source: Microorganisms. 2020 Jul 4;8(7):1002. doi: 10.3390/microorganisms8071002 (PMC7409357; doi:10.3390/microorganisms8071002)
Supplement: Supplementary file 1 [file microorganisms-08-01002-s001.zip › Geobacillus_icigianus_supplementary/Blast_results/3-hexulose-6-phosphate synthase/Gthermoglucosidasius_Gicigianus.html]

NCBI Blast:tr|A0A369X4I9|A0A369X4I9\_PARTM 6-phospho-3-hexuloisomerase...


 


- NCBI Home
- Sign in to NCBI
- Skip to Main Content
- Skip to Navigation
- About NCBI Accesskeys

National Institutes of Health

U.S. National Library of Medicine

National Center for Biotechnology Information

NCBI homepage

Log in


Show account info

Close

#### Account

Logged in as:  
**username**

- Dashboard (My NCBI)
- Publications (My Bibliography)
- Account settings
- Log out

COVID-19 is an emerging, rapidly evolving situation.  
Get the latest public health information from CDC: https://www.coronavirus.gov .  
Get the latest research from NIH: https://www.nih.gov/coronavirus.  
Find NCBI SARS-CoV-2 literature, sequence, and clinical content: https://www.ncbi.nlm.nih.gov/sars-cov-2/.

BLAST ® » blastp suite-2sequences »

# results for RID-F3WUV6D3114

- Home
- Recent Results
- Saved Strategies
- Help


- Edit Search
- Save Search
- Search Summary

  Search Parameters

  | Search parameter name | Search parameter value |
  | --- | --- |
  | Program | blastp |
  | Word size | 3 |
  | Expect value | 10 |
  | Hitlist size | 100 |
  | Gapcosts | 11,1 |
  | Matrix | BLOSUM62 |
  | Filter string | F |
  | Genetic Code | 1 |
  | Window Size | 40 |
  | Threshold | 11 |
  | Composition-based stats | 2 |

  Karlin-Altschul statistics

  | Params | Ungapped | Gapped |
  | --- | --- | --- |
  | Lambda | 0.318322 | 0.267 |
  | K | 0.133732 | 0.041 |
  | H | 0.365455 | 0.14 |

  Results Statistics

  | Results Statistics parameter name | Results Statistics parameter value |
  | --- | --- |
  | Effective search space | 75062751 |

- How to read this report?
- BLAST Help Videos
- Back to Traditional Results Page


Job Title
:   tr|A0A369X4I9|A0A369X4I9\_PARTM 6-phospho-3-hexuloisomerase...
    ...

    tr|A0A369X4I9|A0A369X4I9\_PARTM 6-phospho-3-hexuloisomerase...

RID
:   F3WUV6D3114
    Search expires on 06-24 20:19 pm

    - Download All
      - Text
      - XML
      - ASN.1
      - JSON Seq-align
      - Hit Table(text)
      - Hit Table(csv)
      - Multiple-file XML2
      - Single-file XML2
      - Multiple-file JSON
      - Single-file JSON
      - SAM

Results for
:   lcl|Query\_30600 tr|A0A369X4I9|A0A369X4I9\_PARTM 6-phospho-3-hexuloisomerase OS=Parageobacillus thermoglucosidasius O...(187aa)

Program
:   Blast 2 sequences

    - Citation

      Reference 

      Stephen F. Altschul, Thomas L. Madden, Alejandro A. Schäffer, Jinghui Zhang, Zheng Zhang, Webb Miller, and David J. Lipman (1997), "Gapped BLAST and PSI-BLAST: a new generation of protein database search programs", Nucleic Acids Res. 25:3389-3402.

      Reference - compositional score matrix adjustment

      Stephen F. Altschul, John C. Wootton, E. Michael Gertz, Richa Agarwala, Aleksandr Morgulis, Alejandro A. Schäffer, and Yi-Kuo Yu (2005) "Protein database searches using compositionally adjusted substitution matrices", FEBS J. 272:5101-5109.

Query ID
:   lcl|Query\_30600
    lcl|Query\_30600
    (amino acid)

Query Descr
:   tr|A0A369X4I9|A0A369X4I9\_PARTM 6-phospho-3-hexuloisomerase OS=Parageobacillus thermoglucosidasius OX=1426 GN=hxlB PE=4 SV=1
    ...

    tr|A0A369X4I9|A0A369X4I9\_PARTM 6-phospho-3-hexuloisomerase OS=Parageobacillus thermoglucosidasius OX=1426 GN=hxlB PE=4 SV=1

Query Length
:   187

Subject ID
:   lcl|Query\_30602 and 3229 more subject(s)
    (amino acid)

Subject Descr
:   - See details

      Multiple subjects information

      | Subject ID | Description | Length |
      | --- | --- | --- |
      | lcl|Query\_30602 | KFX36825.1 ZIP family metal transporter [Geobacillus icigianus] | 244 |
      | lcl|Query\_30603 | KFX36828.1 S9 family peptidase [Geobacillus icigianus] | 672 |
      | lcl|Query\_30604 | KFX36829.1 amino acid permease [Geobacillus icigianus] | 474 |
      | lcl|Query\_30605 | KFX36830.1 M3 family oligoendopeptidase [Geobacillus icigianus] | 564 |
      | lcl|Query\_30606 | KFX36831.1 hypothetical protein EP10\_00185 [Geobacillus icigianus] | 202 |
      | lcl|Query\_30607 | KFX36832.1 ATP-dependent Clp protease ATP-binding subunit [Geobacillus icigianus] | 711 |
      | lcl|Query\_30608 | KFX36833.1 hypothetical protein EP10\_00195 [Geobacillus icigianus] | 252 |
      | lcl|Query\_30609 | KFX36834.2 hypothetical protein EP10\_00200 [Geobacillus icigianus] | 358 |
      | lcl|Query\_30610 | PUA93360.1 methyl-accepting chemotaxis protein [Geobacillus icigianus] | 553 |
      | lcl|Query\_30611 | KFX36835.1 spore germination protein [Geobacillus icigianus] | 504 |
      | lcl|Query\_30612 | KFX36836.1 Ger(x)C family spore germination protein [Geobacillus icigianus] | 362 |
      | lcl|Query\_30613 | KFX36837.1 spore gernimation protein GerB [Geobacillus icigianus] | 370 |
      | lcl|Query\_30614 | KFX36838.1 7-cyano-7-deazaguanine synthase QueC [Geobacillus icigianus] | 223 |
      | lcl|Query\_30615 | KFX36839.1 6-carboxytetrahydropterin synthase QueD [Geobacillus icigianus] | 145 |
      | lcl|Query\_30616 | KFX36840.1 7-carboxy-7-deazaguanine synthase QueE [Geobacillus icigianus] | 244 |
      | lcl|Query\_30617 | KFX36841.1 NADPH-dependent 7-cyano-7-deazaguanine reductase QueF [Geobacillus icigianus] | 165 |
      | lcl|Query\_30618 | KFX36842.1 oxidoreductase [Geobacillus icigianus] | 227 |
      | lcl|Query\_30619 | PUA93361.1 DUF2187 domain-containing protein [Geobacillus icigianus] | 58 |
      | lcl|Query\_30620 | KFX36843.1 LysM peptidoglycan-binding domain-containing protein [Geobacillus icigianus] | 194 |
      | lcl|Query\_30621 | KFX36844.1 hypothetical protein EP10\_00260 [Geobacillus icigianus] | 64 |
      | lcl|Query\_30622 | KFX36845.1 hypothetical protein EP10\_00265 [Geobacillus icigianus] | 189 |
      | lcl|Query\_30623 | KFX36846.1 3-oxoacyl-ACP reductase [Geobacillus icigianus] | 249 |
      | lcl|Query\_30624 | KFX36847.1 hypothetical protein EP10\_00280 [Geobacillus icigianus] | 81 |
      | lcl|Query\_30625 | KFX36848.1 PAS domain-containing sensor histidine kinase [Geobacillus icigianus] | 420 |
      | lcl|Query\_30626 | KFX36849.1 cation:proton antiporter [Geobacillus icigianus] | 382 |
      | lcl|Query\_30627 | PUA93362.1 hypothetical protein EP10\_19080 [Geobacillus icigianus] | 93 |
      | lcl|Query\_30628 | KFX36851.1 transcription antiterminator [Geobacillus icigianus] | 285 |
      | lcl|Query\_30629 | KFX36852.1 PTS glucose transporter subunit IICBA [Geobacillus icigianus] | 671 |
      | lcl|Query\_30630 | KFX36853.1 phosphocarrier protein HPr [Geobacillus icigianus] | 88 |
      | lcl|Query\_30631 | KFX36854.1 phosphoenolpyruvate--protein phosphotransferase [Geobacillus icigianus] | 573 |
      | lcl|Query\_30632 | KFX36855.1 HAMP domain-containing protein [Geobacillus icigianus] | 658 |
      | lcl|Query\_30633 | KFX36856.1 2-oxoglutarate dehydrogenase E1 component [Geobacillus icigianus] | 950 |
      | lcl|Query\_30634 | KFX36857.1 2-oxoglutarate dehydrogenase complex dihydrolipoyllysine-residue succinyltransferase [Geobacillus icigianus] | 424 |
      | lcl|Query\_30635 | KFX36858.1 hypothetical protein EP10\_00335 [Geobacillus icigianus] | 82 |
      | lcl|Query\_30636 | KFX36859.1 hypothetical protein EP10\_00340 [Geobacillus icigianus] | 249 |
      | lcl|Query\_30637 | PUA93363.1 hypothetical protein EP10\_19085 [Geobacillus icigianus] | 84 |
      | lcl|Query\_30638 | KFX36811.1 B12-binding domain-containing radical SAM protein [Geobacillus icigianus] | 590 |
      | lcl|Query\_30639 | KFX36820.1 pyridoxal phosphate-dependent aminotransferase [Geobacillus icigianus] | 391 |
      | lcl|Query\_30640 | KFX36826.1 MarR family transcriptional regulator [Geobacillus icigianus] | 153 |
      | lcl|Query\_30641 | KFX36827.1 phosphate propanoyltransferase [Geobacillus icigianus] | 188 |
      | lcl|Query\_30642 | KFX36763.1 hypothetical protein EP10\_00380 [Geobacillus icigianus] | 71 |
      | lcl|Query\_30643 | KFX36764.1 preprotein translocase subunit SecG [Geobacillus icigianus] | 76 |
      | lcl|Query\_30644 | KFX36765.2 carboxylesterase [Geobacillus icigianus] | 247 |
      | lcl|Query\_30645 | KFX36766.1 ribonuclease R [Geobacillus icigianus] | 755 |
      | lcl|Query\_30646 | KFX36767.1 SsrA-binding protein SmpB [Geobacillus icigianus] | 155 |
      | lcl|Query\_30647 | KFX36734.1 hypothetical protein EP10\_00415, partial [Geobacillus icigianus] | 336 |
      | lcl|Query\_30648 | KFX36735.1 glycosyltransferase [Geobacillus icigianus] | 342 |
      | lcl|Query\_30649 | KFX36736.1 hydroxyethylthiazole kinase [Geobacillus icigianus] | 269 |
      | lcl|Query\_30650 | KFX36737.1 bifunctional hydroxymethylpyrimidine kinase/phosphomethylpyrimidine kinase [Geobacillus icigianus] | 267 |
      | lcl|Query\_30651 | KFX36738.1 thiamine phosphate synthase [Geobacillus icigianus] | 221 |
      | lcl|Query\_30652 | KFX36739.1 acyltransferase [Geobacillus icigianus] | 331 |
      | lcl|Query\_30653 | KFX36740.1 twin-arginine translocase TatA/TatE family subunit [Geobacillus icigianus] | 56 |
      | lcl|Query\_30654 | PUA93356.1 GHKL domain-containing protein [Geobacillus icigianus] | 382 |
      | lcl|Query\_30655 | KFX36744.2 AbrB family transcriptional regulator [Geobacillus icigianus] | 374 |
      | lcl|Query\_30656 | KFX36745.1 BAX inhibitor (BI)-1/YccA family protein [Geobacillus icigianus] | 212 |
      | lcl|Query\_30657 | KFX36747.1 hydroxypyruvate isomerase [Geobacillus icigianus] | 264 |
      | lcl|Query\_30658 | KFX36748.1 NAD(P)-dependent oxidoreductase [Geobacillus icigianus] | 298 |
      | lcl|Query\_30659 | KFX36749.1 FAD-binding oxidoreductase [Geobacillus icigianus] | 440 |
      | lcl|Query\_30660 | KFX36750.2 glycolate oxidase [Geobacillus icigianus] | 453 |
      | lcl|Query\_30661 | KFX36751.1 FAD-binding protein [Geobacillus icigianus] | 482 |
      | lcl|Query\_30662 | KFX36752.1 malate synthase G [Geobacillus icigianus] | 727 |
      | lcl|Query\_30663 | KFX36753.1 NAD-dependent malic enzyme [Geobacillus icigianus] | 405 |
      | lcl|Query\_30664 | KFX36755.1 DUF1998 domain-containing protein [Geobacillus icigianus] | 758 |
      | lcl|Query\_30665 | PUA93357.1 hypothetical protein EP10\_19130 [Geobacillus icigianus] | 431 |
      | lcl|Query\_30666 | PUA93358.1 spore coat protein CotH [Geobacillus icigianus] | 83 |
      | lcl|Query\_30667 | KFX36756.1 cell division regulator GpsB [Geobacillus icigianus] | 98 |
      | lcl|Query\_30668 | KFX36741.1 hypothetical protein EP10\_00455 [Geobacillus icigianus] | 191 |
      | lcl|Query\_30669 | KFX36742.1 peptidase S8 [Geobacillus icigianus] | 457 |
      | lcl|Query\_30670 | KFX36743.1 cysteine synthase A [Geobacillus icigianus] | 307 |
      | lcl|Query\_30671 | KFX36754.1 IclR family transcriptional regulator [Geobacillus icigianus] | 261 |
      | lcl|Query\_30672 | KFX36757.2 AAA family ATPase, partial [Geobacillus icigianus] | 64 |
      | lcl|Query\_30673 | PUA93359.1 general secretion pathway protein A [Geobacillus icigianus] | 57 |
      | lcl|Query\_30674 | KFX36715.1 endonuclease I [Geobacillus icigianus] | 315 |
      | lcl|Query\_30675 | KFX36716.1 sodium:proton antiporter [Geobacillus icigianus] | 501 |
      | lcl|Query\_30676 | KFX36717.1 metallophosphoesterase [Geobacillus icigianus] | 285 |
      | lcl|Query\_30677 | KFX36718.1 MerR family DNA-binding transcriptional regulator [Geobacillus icigianus] | 101 |
      | lcl|Query\_30678 | PUA93355.1 Fur-regulated basic protein FbpA [Geobacillus icigianus] | 71 |
      | lcl|Query\_30679 | KFX36719.1 hypothetical protein EP10\_00605 [Geobacillus icigianus] | 123 |
      | lcl|Query\_30680 | KFX36720.1 hypothetical protein EP10\_00610 [Geobacillus icigianus] | 129 |
      | lcl|Query\_30681 | KFX36714.1 DUF4829 domain-containing protein [Geobacillus icigianus] | 169 |
      | lcl|Query\_30682 | KFX36708.1 type VII secretion protein EsaA [Geobacillus icigianus] | 927 |
      | lcl|Query\_30683 | KFX36709.1 type VII secretion protein EssA [Geobacillus icigianus] | 159 |
      | lcl|Query\_30684 | PUA93352.1 hypothetical protein EP10\_19155 [Geobacillus icigianus] | 359 |
      | lcl|Query\_30685 | KFX36711.1 hypothetical protein EP10\_00650 [Geobacillus icigianus] | 85 |
      | lcl|Query\_30686 | KFX36712.2 DUF5082 domain-containing protein [Geobacillus icigianus] | 100 |
      | lcl|Query\_30687 | PUA93353.1 hypothetical protein EP10\_19165 [Geobacillus icigianus] | 392 |
      | lcl|Query\_30688 | PUA93354.1 type VII secretion protein EssC, partial [Geobacillus icigianus] | 673 |
      | lcl|Query\_30689 | KFX36710.2 DUF5082 domain-containing protein [Geobacillus icigianus] | 100 |
      | lcl|Query\_30690 | KFX36660.1 acyltransferase [Geobacillus icigianus] | 165 |
      | lcl|Query\_30691 | KFX36661.1 pyrophosphatase PpaX [Geobacillus icigianus] | 222 |
      | lcl|Query\_30692 | KFX36662.1 hypothetical protein EP10\_00680 [Geobacillus icigianus] | 312 |
      | lcl|Query\_30693 | KFX36663.1 prolipoprotein diacylglyceryl transferase [Geobacillus icigianus] | 270 |
      | lcl|Query\_30694 | KFX36664.1 HPr kinase/phosphorylase [Geobacillus icigianus] | 311 |
      | lcl|Query\_30695 | KFX36665.1 hypothetical protein EP10\_00695 [Geobacillus icigianus] | 67 |
      | lcl|Query\_30696 | KFX36666.1 phage holin family protein [Geobacillus icigianus] | 120 |
      | lcl|Query\_30697 | KFX36667.1 DUF4870 domain-containing protein [Geobacillus icigianus] | 108 |
      | lcl|Query\_30698 | KFX36668.1 excinuclease ABC subunit UvrA [Geobacillus icigianus] | 952 |
      | lcl|Query\_30699 | KFX36669.1 excinuclease ABC subunit UvrB [Geobacillus icigianus] | 658 |
      | lcl|Query\_30700 | KFX36670.1 DUF2198 domain-containing protein [Geobacillus icigianus] | 74 |
      | lcl|Query\_30701 | KFX36671.1 PDZ domain-containing protein [Geobacillus icigianus] | 390 |
      | lcl|Query\_30702 | KFX36672.1 helix-turn-helix domain-containing protein [Geobacillus icigianus] | 184 |
      | lcl|Query\_30703 | KFX36673.1 hypothetical protein EP10\_00740 [Geobacillus icigianus] | 169 |
      | lcl|Query\_30704 | KFX36674.1 hypothetical protein EP10\_00745 [Geobacillus icigianus] | 556 |
      | lcl|Query\_30705 | KFX36675.2 glycosyltransferase [Geobacillus icigianus] | 330 |
      | lcl|Query\_30706 | KFX36676.1 PDZ domain-containing protein [Geobacillus icigianus] | 487 |
      | lcl|Query\_30707 | KFX36677.1 peptidase M23 [Geobacillus icigianus] | 428 |
      | lcl|Query\_30708 | KFX36678.1 ABC transporter permease [Geobacillus icigianus] | 297 |
      | lcl|Query\_30709 | KFX36679.1 cell division ATP-binding protein FtsE [Geobacillus icigianus] | 228 |
      | lcl|Query\_30710 | KFX36680.1 cytochrome c [Geobacillus icigianus] | 111 |
      | lcl|Query\_30711 | KFX36681.1 YitT family protein [Geobacillus icigianus] | 287 |
      | lcl|Query\_30712 | KFX36682.1 type II toxin-antitoxin system RelE/ParE family toxin [Geobacillus icigianus] | 86 |
      | lcl|Query\_30713 | KFX36683.2 hypothetical protein EP10\_00800 [Geobacillus icigianus] | 86 |
      | lcl|Query\_30714 | KFX36685.1 preprotein translocase subunit SecA [Geobacillus icigianus] | 837 |
      | lcl|Query\_30715 | KFX36686.1 hypothetical protein EP10\_00815 [Geobacillus icigianus] | 178 |
      | lcl|Query\_30716 | KFX36687.1 ribosome-associated translation inhibitor RaiA [Geobacillus icigianus] | 182 |
      | lcl|Query\_30717 | KFX36688.1 flagellar protein FliT [Geobacillus icigianus] | 116 |
      | lcl|Query\_30718 | KFX36689.1 flagella export chaperone FliS [Geobacillus icigianus] | 132 |
      | lcl|Query\_30719 | KFX36690.1 flagellar hook-associated protein 2 [Geobacillus icigianus] | 523 |
      | lcl|Query\_30720 | KFX36691.1 flagellar biosynthesis protein FlaG [Geobacillus icigianus] | 115 |
      | lcl|Query\_30721 | KFX36692.1 flagellin [Geobacillus icigianus] | 269 |
      | lcl|Query\_30722 | KFX36693.1 methionyl-tRNA formyltransferase [Geobacillus icigianus] | 268 |
      | lcl|Query\_30723 | KFX36684.2 peptide chain release factor 2 [Geobacillus icigianus] | 366 |
      | lcl|Query\_30724 | PUA93351.1 hypothetical protein EP10\_19170, partial [Geobacillus icigianus] | 393 |
      | lcl|Query\_30725 | KFX36649.1 glycosyl transferase family 2, partial [Geobacillus icigianus] | 81 |
      | lcl|Query\_30726 | KFX36650.1 hydrolase, partial [Geobacillus icigianus] | 209 |
      | lcl|Query\_30727 | KFX36577.1 HD domain-containing protein [Geobacillus icigianus] | 353 |
      | lcl|Query\_30728 | KFX36578.1 DNA gyrase subunit A [Geobacillus icigianus] | 820 |
      | lcl|Query\_30729 | KFX36579.1 DNA topoisomerase (ATP-hydrolyzing) subunit B [Geobacillus icigianus] | 639 |
      | lcl|Query\_30730 | KFX36580.1 DNA replication and repair protein RecF [Geobacillus icigianus] | 372 |
      | lcl|Query\_30731 | KFX36581.1 S4 domain-containing protein YaaA [Geobacillus icigianus] | 73 |
      | lcl|Query\_30732 | KFX36582.1 DNA polymerase III subunit beta [Geobacillus icigianus] | 378 |
      | lcl|Query\_30733 | KFX36583.1 chromosomal replication initiator protein DnaA [Geobacillus icigianus] | 450 |
      | lcl|Query\_30734 | KFX36584.1 50S ribosomal protein L34 [Geobacillus icigianus] | 44 |
      | lcl|Query\_30735 | KFX36585.1 ribonuclease P protein component [Geobacillus icigianus] | 121 |
      | lcl|Query\_30736 | KFX36586.1 Membrane integrase YidC [Geobacillus icigianus] | 254 |
      | lcl|Query\_30737 | KFX36587.1 protein jag [Geobacillus icigianus] | 218 |
      | lcl|Query\_30738 | KFX36588.1 tRNA uridine-5-carboxymethylaminomethyl(34) synthesis GTPase MnmE [Geobacillus icigianus] | 462 |
      | lcl|Query\_30739 | KFX36589.1 tRNA uridine-5-carboxymethylaminomethyl(34) synthesis enzyme MnmG [Geobacillus icigianus] | 629 |
      | lcl|Query\_30740 | KFX36591.1 nucleoid occlusion protein [Geobacillus icigianus] | 281 |
      | lcl|Query\_30741 | KFX36592.1 ParA family protein [Geobacillus icigianus] | 253 |
      | lcl|Query\_30742 | KFX36593.1 ParB/RepB/Spo0J family partition protein [Geobacillus icigianus] | 289 |
      | lcl|Query\_30743 | KFX36594.1 DUF554 domain-containing protein [Geobacillus icigianus] | 232 |
      | lcl|Query\_30744 | KFX36595.1 spore protease YyaC [Geobacillus icigianus] | 207 |
      | lcl|Query\_30745 | KFX36596.2 mechanosensitive ion channel protein MscS [Geobacillus icigianus] | 299 |
      | lcl|Query\_30746 | KFX36597.1 DUF951 domain-containing protein [Geobacillus icigianus] | 65 |
      | lcl|Query\_30747 | KFX36598.1 redox-regulated ATPase YchF [Geobacillus icigianus] | 366 |
      | lcl|Query\_30748 | KFX36599.1 30S ribosomal protein S6 [Geobacillus icigianus] | 95 |
      | lcl|Query\_30749 | KFX36600.1 single-stranded DNA-binding protein [Geobacillus icigianus] | 164 |
      | lcl|Query\_30750 | KFX36601.1 30S ribosomal protein S18 [Geobacillus icigianus] | 78 |
      | lcl|Query\_30751 | KFX36602.1 DUF2232 domain-containing protein [Geobacillus icigianus] | 308 |
      | lcl|Query\_30752 | KFX36603.1 DHH family phosphoesterase [Geobacillus icigianus] | 657 |
      | lcl|Query\_30753 | KFX36604.1 50S ribosomal protein L9 [Geobacillus icigianus] | 149 |
      | lcl|Query\_30754 | KFX36607.1 DNA-binding response regulator [Geobacillus icigianus] | 237 |
      | lcl|Query\_30755 | KFX36608.1 cell wall metabolism sensor histidine kinase WalK [Geobacillus icigianus] | 609 |
      | lcl|Query\_30756 | KFX36609.1 transcriptional regulator [Geobacillus icigianus] | 443 |
      | lcl|Query\_30757 | KFX36610.1 transcriptional regulator [Geobacillus icigianus] | 260 |
      | lcl|Query\_30758 | KFX36612.1 PDZ domain-containing protein [Geobacillus icigianus] | 406 |
      | lcl|Query\_30759 | PUA93350.1 CxxH/CxxC protein [Geobacillus icigianus] | 49 |
      | lcl|Query\_30760 | KFX36613.1 23S rRNA (pseudouridine(1915)-N(3))-methyltransferase RlmH [Geobacillus icigianus] | 159 |
      | lcl|Query\_30761 | KFX36614.1 GGDEF domain-containing protein [Geobacillus icigianus] | 412 |
      | lcl|Query\_30762 | KFX36615.1 glycerate kinase [Geobacillus icigianus] | 380 |
      | lcl|Query\_30763 | KFX36616.1 mannose-6-phosphate isomerase, class I [Geobacillus icigianus] | 320 |
      | lcl|Query\_30764 | KFX36617.1 FAD-dependent oxidoreductase [Geobacillus icigianus] | 507 |
      | lcl|Query\_30765 | KFX36618.1 hypothetical protein EP10\_01090 [Geobacillus icigianus] | 63 |
      | lcl|Query\_30766 | KFX36619.1 transcriptional regulator [Geobacillus icigianus] | 466 |
      | lcl|Query\_30767 | KFX36620.1 amino acid permease [Geobacillus icigianus] | 479 |
      | lcl|Query\_30768 | KFX36590.1 16S rRNA (guanine(527)-N(7))-methyltransferase RsmG [Geobacillus icigianus] | 238 |
      | lcl|Query\_30769 | KFX36605.1 replicative DNA helicase [Geobacillus icigianus] | 454 |
      | lcl|Query\_30770 | KFX36606.1 adenylosuccinate synthase [Geobacillus icigianus] | 428 |
      | lcl|Query\_30771 | KFX36611.1 MBL fold metallo-hydrolase [Geobacillus icigianus] | 264 |
      | lcl|Query\_30772 | KFX36575.1 IS4 family transposase [Geobacillus icigianus] | 377 |
      | lcl|Query\_30773 | PUA93349.1 hypothetical protein EP10\_19180 [Geobacillus icigianus] | 75 |
      | lcl|Query\_30774 | KFX36545.1 cation transporter, partial [Geobacillus icigianus] | 312 |
      | lcl|Query\_30775 | KFX36546.1 antibiotic biosynthesis monooxygenase [Geobacillus icigianus] | 103 |
      | lcl|Query\_30776 | PUA93348.1 ABC transporter ATP-binding protein [Geobacillus icigianus] | 278 |
      | lcl|Query\_30777 | KFX36547.1 iron ABC transporter permease [Geobacillus icigianus] | 346 |
      | lcl|Query\_30778 | KFX36548.1 ABC transporter substrate-binding protein [Geobacillus icigianus] | 307 |
      | lcl|Query\_30779 | KFX36549.1 LysE family translocator [Geobacillus icigianus] | 208 |
      | lcl|Query\_30780 | KFX36550.1 N-acetyltransferase [Geobacillus icigianus] | 184 |
      | lcl|Query\_30781 | KFX36551.1 hypothetical protein EP10\_01150 [Geobacillus icigianus] | 506 |
      | lcl|Query\_30782 | KFX36552.1 iron ABC transporter permease [Geobacillus icigianus] | 327 |
      | lcl|Query\_30783 | KFX36554.1 iron-siderophore ABC transporter substrate-binding protein [Geobacillus icigianus] | 313 |
      | lcl|Query\_30784 | KFX36555.1 FAD-binding oxidoreductase [Geobacillus icigianus] | 408 |
      | lcl|Query\_30785 | KFX36556.1 hypothetical protein EP10\_01175 [Geobacillus icigianus] | 71 |
      | lcl|Query\_30786 | KFX36553.1 iron ABC transporter permease [Geobacillus icigianus] | 332 |
      | lcl|Query\_30787 | KFX36495.1 sigma factor G inhibitor Gin [Geobacillus icigianus] | 68 |
      | lcl|Query\_30788 | KFX36496.1 aminotransferase class V-fold PLP-dependent enzyme [Geobacillus icigianus] | 480 |
      | lcl|Query\_30789 | KFX36497.1 dTMP kinase [Geobacillus icigianus] | 209 |
      | lcl|Query\_30790 | KFX36498.1 DNA polymerase III subunit delta' [Geobacillus icigianus] | 330 |
      | lcl|Query\_30791 | KFX36499.1 stage 0 sporulation protein [Geobacillus icigianus] | 275 |
      | lcl|Query\_30792 | KFX36500.1 DNA replication initiation control protein YabA [Geobacillus icigianus] | 120 |
      | lcl|Query\_30793 | KFX36501.1 tRNA1(Val) (adenine(37)-N6)-methyltransferase [Geobacillus icigianus] | 249 |
      | lcl|Query\_30794 | KFX36502.1 16S rRNA (cytidine(1402)-2'-O)-methyltransferase [Geobacillus icigianus] | 301 |
      | lcl|Query\_30795 | KFX36503.1 AbrB/MazE/SpoVT family DNA-binding domain-containing protein [Geobacillus icigianus] | 98 |
      | lcl|Query\_30796 | KFX36462.1 DUF814 domain-containing protein [Geobacillus icigianus] | 579 |
      | lcl|Query\_30797 | KFX36463.1 calcium-translocating P-type ATPase, SERCA-type [Geobacillus icigianus] | 890 |
      | lcl|Query\_30798 | KFX36464.1 YicC family protein [Geobacillus icigianus] | 291 |
      | lcl|Query\_30799 | KFX36465.1 DUF370 domain-containing protein [Geobacillus icigianus] | 97 |
      | lcl|Query\_30800 | KFX36466.1 guanylate kinase [Geobacillus icigianus] | 205 |
      | lcl|Query\_30801 | KFX36467.1 DNA-directed RNA polymerase subunit omega [Geobacillus icigianus] | 65 |
      | lcl|Query\_30802 | KFX36468.1 bifunctional phosphopantothenoylcysteine decarboxylase/phosphopantothenate--cysteine ligase CoaBC [Geobacillus icigianus] | 401 |
      | lcl|Query\_30803 | KFX36469.1 primosomal protein N' [Geobacillus icigianus] | 801 |
      | lcl|Query\_30804 | KFX36471.1 methionyl-tRNA formyltransferase [Geobacillus icigianus] | 319 |
      | lcl|Query\_30805 | KFX36472.1 16S rRNA (cytosine(967)-C(5))-methyltransferase, partial [Geobacillus icigianus] | 154 |
      | lcl|Query\_30806 | KFX36470.1 peptide deformylase [Geobacillus icigianus] | 157 |
      | lcl|Query\_30807 | KFX36440.1 RNA methyltransferase [Geobacillus icigianus] | 246 |
      | lcl|Query\_30808 | KFX36441.1 cytochrome aa3 quinol oxidase subunit II [Geobacillus icigianus] | 301 |
      | lcl|Query\_30809 | KFX36442.1 cytochrome aa3 quinol oxidase subunit I [Geobacillus icigianus] | 648 |
      | lcl|Query\_30810 | KFX36443.1 cytochrome aa3 quinol oxidase subunit III [Geobacillus icigianus] | 204 |
      | lcl|Query\_30811 | KFX36444.1 cytochrome aa3 quinol oxidase subunit IV [Geobacillus icigianus] | 99 |
      | lcl|Query\_30812 | KFX36445.1 amino acid ABC transporter ATP-binding protein [Geobacillus icigianus] | 240 |
      | lcl|Query\_30813 | KFX36446.1 amino acid ABC transporter permease [Geobacillus icigianus] | 218 |
      | lcl|Query\_30814 | KFX36447.1 basic amino acid ABC transporter substrate-binding protein [Geobacillus icigianus] | 273 |
      | lcl|Query\_30815 | KFX36438.2 MFS transporter [Geobacillus icigianus] | 413 |
      | lcl|Query\_30816 | KFX36439.1 IS701 family transposase, partial [Geobacillus icigianus] | 220 |
      | lcl|Query\_30817 | KFX36414.1 4'-phosphopantetheinyl transferase [Geobacillus icigianus] | 253 |
      | lcl|Query\_30818 | KFX36415.1 iron-uptake system-binding protein [Geobacillus icigianus] | 313 |
      | lcl|Query\_30819 | PUA93344.1 type II toxin-antitoxin system death-on-curing family toxin [Geobacillus icigianus] | 137 |
      | lcl|Query\_30820 | PUA93345.1 hypothetical protein EP10\_19210 [Geobacillus icigianus] | 67 |
      | lcl|Query\_30821 | PUA93346.1 hypothetical protein EP10\_19215 [Geobacillus icigianus] | 77 |
      | lcl|Query\_30822 | KFX36417.1 hypothetical protein EP10\_01430 [Geobacillus icigianus] | 125 |
      | lcl|Query\_30823 | KFX36418.1 ABC transporter ATP-binding protein [Geobacillus icigianus] | 222 |
      | lcl|Query\_30824 | KFX36419.1 hypothetical protein EP10\_01440 [Geobacillus icigianus] | 661 |
      | lcl|Query\_30825 | KFX36420.1 acyl-CoA thioesterase [Geobacillus icigianus] | 145 |
      | lcl|Query\_30826 | KFX36421.1 5-methyltetrahydropteroyltriglutamate--homocysteine S-methyltransferase [Geobacillus icigianus] | 768 |
      | lcl|Query\_30827 | KFX36422.1 hypothetical protein EP10\_01460 [Geobacillus icigianus] | 73 |
      | lcl|Query\_30828 | PUA93347.1 ATP-dependent helicase, partial [Geobacillus icigianus] | 364 |
      | lcl|Query\_30829 | KFX36412.1 IS1182 family transposase [Geobacillus icigianus] | 522 |
      | lcl|Query\_30830 | KFX36243.1 RNA polymerase sporulation sigma factor SigK [Geobacillus icigianus] | 237 |
      | lcl|Query\_30831 | PUA93342.1 YrzI family small protein [Geobacillus icigianus] | 47 |
      | lcl|Query\_30832 | KFX36244.1 hypothetical protein EP10\_01500 [Geobacillus icigianus] | 98 |
      | lcl|Query\_30833 | KFX36245.1 hypothetical protein EP10\_01505 [Geobacillus icigianus] | 82 |
      | lcl|Query\_30834 | KFX36246.1 bifunctional cystathionine gamma-lyase/homocysteine desulfhydrase [Geobacillus icigianus] | 377 |
      | lcl|Query\_30835 | KFX36247.1 cysteine synthase family protein [Geobacillus icigianus] | 308 |
      | lcl|Query\_30836 | KFX36248.1 5'-methylthioadenosine/S-adenosylhomocysteine nucleosidase [Geobacillus icigianus] | 234 |
      | lcl|Query\_30837 | KFX36249.1 class I SAM-dependent methyltransferase [Geobacillus icigianus] | 215 |
      | lcl|Query\_30838 | KFX36250.1 DUF2536 domain-containing protein [Geobacillus icigianus] | 69 |
      | lcl|Query\_30839 | KFX36251.1 DUF1510 domain-containing protein [Geobacillus icigianus] | 208 |
      | lcl|Query\_30840 | KFX36252.1 penicillin-binding protein 2 [Geobacillus icigianus] | 589 |
      | lcl|Query\_30841 | KFX36253.1 transcription elongation factor GreA [Geobacillus icigianus] | 158 |
      | lcl|Query\_30842 | KFX36254.1 uridine kinase [Geobacillus icigianus] | 211 |
      | lcl|Query\_30843 | KFX36255.1 U32 family peptidase [Geobacillus icigianus] | 422 |
      | lcl|Query\_30844 | KFX36256.1 U32 family peptidase [Geobacillus icigianus] | 309 |
      | lcl|Query\_30845 | KFX36257.1 O-methyltransferase [Geobacillus icigianus] | 214 |
      | lcl|Query\_30846 | KFX36258.1 endolytic transglycosylase MltG [Geobacillus icigianus] | 363 |
      | lcl|Query\_30847 | KFX36259.1 DUF1292 domain-containing protein [Geobacillus icigianus] | 98 |
      | lcl|Query\_30848 | KFX36260.1 Holliday junction resolvase RuvX [Geobacillus icigianus] | 138 |
      | lcl|Query\_30849 | KFX36262.1 alanine--tRNA ligase [Geobacillus icigianus] | 878 |
      | lcl|Query\_30850 | KFX36263.1 AI-2E family transporter [Geobacillus icigianus] | 353 |
      | lcl|Query\_30851 | PUA93343.1 DUF3918 domain-containing protein [Geobacillus icigianus] | 44 |
      | lcl|Query\_30852 | KFX36264.1 hypothetical protein EP10\_01615 [Geobacillus icigianus] | 63 |
      | lcl|Query\_30853 | KFX36265.1 photosystem reaction center subunit H [Geobacillus icigianus] | 157 |
      | lcl|Query\_30854 | KFX36266.1 ATP-dependent RecD-like DNA helicase [Geobacillus icigianus] | 785 |
      | lcl|Query\_30855 | KFX36267.1 tetratricopeptide repeat protein [Geobacillus icigianus] | 220 |
      | lcl|Query\_30856 | KFX36268.1 tRNA 2-thiouridine(34) synthase MnmA [Geobacillus icigianus] | 371 |
      | lcl|Query\_30857 | KFX36269.1 cysteine desulfurase [Geobacillus icigianus] | 380 |
      | lcl|Query\_30858 | KFX36270.1 hypothetical protein EP10\_01645 [Geobacillus icigianus] | 61 |
      | lcl|Query\_30859 | KFX36271.1 Rrf2 family transcriptional regulator [Geobacillus icigianus] | 138 |
      | lcl|Query\_30860 | KFX36272.1 YitT family protein [Geobacillus icigianus] | 216 |
      | lcl|Query\_30861 | KFX36273.1 replication-associated recombination protein A [Geobacillus icigianus] | 431 |
      | lcl|Query\_30862 | KFX36274.1 RsfA family transcriptional regulator [Geobacillus icigianus] | 216 |
      | lcl|Query\_30863 | KFX36275.1 tRNA threonylcarbamoyladenosine dehydratase [Geobacillus icigianus] | 253 |
      | lcl|Query\_30864 | KFX36276.1 aspartate--tRNA ligase [Geobacillus icigianus] | 590 |
      | lcl|Query\_30865 | KFX36277.1 histidine--tRNA ligase [Geobacillus icigianus] | 427 |
      | lcl|Query\_30866 | KFX36278.1 alkyl hydroperoxide reductase subunit F [Geobacillus icigianus] | 507 |
      | lcl|Query\_30867 | KFX36279.1 peroxiredoxin [Geobacillus icigianus] | 187 |
      | lcl|Query\_30868 | KFX36281.1 D-tyrosyl-tRNA(Tyr) deacylase [Geobacillus icigianus] | 152 |
      | lcl|Query\_30869 | KFX36282.1 bifunctional (p)ppGpp synthetase/guanosine-3',5'-bis(diphosphate) 3'-pyrophosphohydrolase [Geobacillus icigianus] | 732 |
      | lcl|Query\_30870 | KFX36284.1 adenine phosphoribosyltransferase [Geobacillus icigianus] | 170 |
      | lcl|Query\_30871 | KFX36287.1 hypothetical protein EP10\_01730 [Geobacillus icigianus] | 63 |
      | lcl|Query\_30872 | KFX36288.1 protein translocase subunit SecDF [Geobacillus icigianus] | 750 |
      | lcl|Query\_30873 | KFX36289.1 transcriptional regulator [Geobacillus icigianus] | 94 |
      | lcl|Query\_30874 | KFX36290.1 stage V sporulation protein B [Geobacillus icigianus] | 520 |
      | lcl|Query\_30875 | KFX36291.1 TIGR04086 family membrane protein [Geobacillus icigianus] | 127 |
      | lcl|Query\_30876 | KFX36292.1 preprotein translocase subunit YajC [Geobacillus icigianus] | 90 |
      | lcl|Query\_30877 | KFX36293.1 tRNA guanosine(34) transglycosylase Tgt [Geobacillus icigianus] | 380 |
      | lcl|Query\_30878 | KFX36294.1 S-adenosylmethionine:tRNA ribosyltransferase-isomerase [Geobacillus icigianus] | 342 |
      | lcl|Query\_30879 | KFX36295.2 DUF2905 domain-containing protein [Geobacillus icigianus] | 68 |
      | lcl|Query\_30880 | KFX36296.1 Holliday junction branch migration DNA helicase RuvB [Geobacillus icigianus] | 333 |
      | lcl|Query\_30881 | KFX36297.1 Holliday junction branch migration protein RuvA [Geobacillus icigianus] | 194 |
      | lcl|Query\_30882 | KFX36298.1 Forespore regulator of the sigma-K checkpoint, BofC [Geobacillus icigianus] | 158 |
      | lcl|Query\_30883 | KFX36299.1 DUF3231 domain-containing protein [Geobacillus icigianus] | 337 |
      | lcl|Query\_30884 | KFX36300.1 YebC/PmpR family DNA-binding transcriptional regulator [Geobacillus icigianus] | 247 |
      | lcl|Query\_30885 | KFX36301.1 multidrug ABC transporter ATP-binding protein [Geobacillus icigianus] | 221 |
      | lcl|Query\_30886 | KFX36302.1 hypothetical protein EP10\_01805 [Geobacillus icigianus] | 266 |
      | lcl|Query\_30887 | KFX36303.1 hypothetical protein EP10\_01810 [Geobacillus icigianus] | 111 |
      | lcl|Query\_30888 | KFX36261.1 IreB family regulatory phosphoprotein [Geobacillus icigianus] | 88 |
      | lcl|Query\_30889 | KFX36285.1 single-stranded-DNA-specific exonuclease RecJ [Geobacillus icigianus] | 788 |
      | lcl|Query\_30890 | KFX36286.2 cation transporter [Geobacillus icigianus] | 302 |
      | lcl|Query\_30891 | PUA93341.1 hypothetical protein EP10\_19245 [Geobacillus icigianus] | 174 |
      | lcl|Query\_30892 | KFX36220.1 ABC transporter ATP-binding protein [Geobacillus icigianus] | 201 |
      | lcl|Query\_30893 | KFX36219.1 hypothetical protein EP10\_01825 [Geobacillus icigianus] | 303 |
      | lcl|Query\_30894 | KFX36170.2 23S rRNA (uracil(1939)-C(5))-methyltransferase RlmD [Geobacillus icigianus] | 460 |
      | lcl|Query\_30895 | KFX36171.1 sporulation protein SpoOM [Geobacillus icigianus] | 253 |
      | lcl|Query\_30896 | KFX36172.1 gfo/Idh/MocA family oxidoreductase [Geobacillus icigianus] | 388 |
      | lcl|Query\_30897 | KFX36173.1 mannose-1-phosphate guanylyltransferase [Geobacillus icigianus] | 347 |
      | lcl|Query\_30898 | KFX36174.1 glycosyl transferase family 1 [Geobacillus icigianus] | 395 |
      | lcl|Query\_30899 | KFX36175.1 glycosyl transferase [Geobacillus icigianus] | 371 |
      | lcl|Query\_30900 | KFX36176.1 phosphomannomutase/phosphoglucomutase [Geobacillus icigianus] | 468 |
      | lcl|Query\_30901 | KFX36177.1 LuxR family transcriptional regulator [Geobacillus icigianus] | 547 |
      | lcl|Query\_30902 | KFX36178.1 ATP-binding protein [Geobacillus icigianus] | 133 |
      | lcl|Query\_30903 | KFX36179.1 anti-sigma factor antagonist [Geobacillus icigianus] | 110 |
      | lcl|Query\_30904 | KFX36180.1 PAS domain S-box protein [Geobacillus icigianus] | 509 |
      | lcl|Query\_30905 | KFX36181.1 methyl-accepting chemotaxis protein [Geobacillus icigianus] | 417 |
      | lcl|Query\_30906 | KFX36182.1 phosphomethylpyrimidine synthase ThiC [Geobacillus icigianus] | 563 |
      | lcl|Query\_30907 | PUA93339.1 hypothetical protein EP10\_19265, partial [Geobacillus icigianus] | 98 |
      | lcl|Query\_30908 | KFX36159.1 MFS transporter [Geobacillus icigianus] | 415 |
      | lcl|Query\_30909 | KFX36160.1 transposase [Geobacillus icigianus] | 304 |
      | lcl|Query\_30910 | KFX36161.1 DUF2584 domain-containing protein [Geobacillus icigianus] | 80 |
      | lcl|Query\_30911 | KFX36162.1 S9 family peptidase [Geobacillus icigianus] | 262 |
      | lcl|Query\_30912 | KFX36163.1 XRE family transcriptional regulator [Geobacillus icigianus] | 72 |
      | lcl|Query\_30913 | KFX36164.2 holin [Geobacillus icigianus] | 67 |
      | lcl|Query\_30914 | KFX36165.1 hemolysin activation protein [Geobacillus icigianus] | 72 |
      | lcl|Query\_30915 | PUA93340.1 integrase [Geobacillus icigianus] | 329 |
      | lcl|Query\_30916 | KFX36148.1 long-chain fatty acid--CoA ligase [Geobacillus icigianus] | 560 |
      | lcl|Query\_30917 | KFX36149.1 phosphotriesterase-related protein [Geobacillus icigianus] | 326 |
      | lcl|Query\_30918 | KFX36150.2 hypothetical protein EP10\_02015, partial [Geobacillus icigianus] | 237 |
      | lcl|Query\_30919 | PUA93338.1 IS5 family transposase [Geobacillus icigianus] | 493 |
      | lcl|Query\_30920 | KFX36105.1 hypothetical protein EP10\_02040, partial [Geobacillus icigianus] | 409 |
      | lcl|Query\_30921 | KFX36106.1 ABC transporter permease [Geobacillus icigianus] | 260 |
      | lcl|Query\_30922 | KFX36107.1 molybdenum cofactor guanylyltransferase [Geobacillus icigianus] | 192 |
      | lcl|Query\_30923 | KFX36109.1 hypothetical protein EP10\_02060 [Geobacillus icigianus] | 344 |
      | lcl|Query\_30924 | KFX36099.2 glutamate dehydrogenase, partial [Geobacillus icigianus] | 153 |
      | lcl|Query\_30925 | KFX36100.2 hypothetical protein EP10\_02070 [Geobacillus icigianus] | 89 |
      | lcl|Query\_30926 | KFX36101.2 DUF3419 domain-containing protein [Geobacillus icigianus] | 371 |
      | lcl|Query\_30927 | KFX36102.1 DNA-binding response regulator [Geobacillus icigianus] | 223 |
      | lcl|Query\_30928 | KFX36103.1 histidine kinase [Geobacillus icigianus] | 749 |
      | lcl|Query\_30929 | KFX36104.1 competence pheromone ComX [Geobacillus icigianus] | 51 |
      | lcl|Query\_30930 | KFX36044.1 transcriptional regulator [Geobacillus icigianus] | 435 |
      | lcl|Query\_30931 | KFX36045.1 UDP-glucose/GDP-mannose dehydrogenase family protein [Geobacillus icigianus] | 453 |
      | lcl|Query\_30932 | KFX36046.1 branched-chain amino acid transport system II carrier protein [Geobacillus icigianus] | 436 |
      | lcl|Query\_30933 | KFX36047.1 N-acetylmuramoyl-L-alanine amidase [Geobacillus icigianus] | 447 |
      | lcl|Query\_30934 | KFX36048.1 hypothetical protein EP10\_02125 [Geobacillus icigianus] | 431 |
      | lcl|Query\_30935 | KFX36049.1 plasmid pRiA4b ORF-3 family protein [Geobacillus icigianus] | 238 |
      | lcl|Query\_30936 | KFX36050.1 hypothetical protein EP10\_02135 [Geobacillus icigianus] | 181 |
      | lcl|Query\_30937 | KFX36051.1 hypothetical protein EP10\_02140 [Geobacillus icigianus] | 547 |
      | lcl|Query\_30938 | KFX36052.1 ATP-dependent helicase [Geobacillus icigianus] | 920 |
      | lcl|Query\_30939 | KFX36053.1 Uma2 family endonuclease [Geobacillus icigianus] | 187 |
      | lcl|Query\_30940 | KFX36054.1 PTS fructose transporter subunit IIA [Geobacillus icigianus] | 151 |
      | lcl|Query\_30941 | KFX36055.1 PTS fructose transporter subunit IIBC [Geobacillus icigianus] | 463 |
      | lcl|Query\_30942 | KFX36056.2 PRD domain-containing protein [Geobacillus icigianus] | 671 |
      | lcl|Query\_30943 | KFX36058.1 Appr-1-p processing protein [Geobacillus icigianus] | 162 |
      | lcl|Query\_30944 | KFX36059.1 DUF488 domain-containing protein [Geobacillus icigianus] | 131 |
      | lcl|Query\_30945 | KFX36060.1 hypothetical protein EP10\_02190 [Geobacillus icigianus] | 143 |
      | lcl|Query\_30946 | KFX36061.1 hypothetical protein EP10\_02195 [Geobacillus icigianus] | 437 |
      | lcl|Query\_30947 | KFX36062.1 UTP--glucose-1-phosphate uridylyltransferase [Geobacillus icigianus] | 294 |
      | lcl|Query\_30948 | KFX36063.1 glycosidase [Geobacillus icigianus] | 352 |
      | lcl|Query\_30949 | KFX36064.1 DUF1861 domain-containing protein [Geobacillus icigianus] | 315 |
      | lcl|Query\_30950 | KFX36065.1 carbohydrate ABC transporter permease [Geobacillus icigianus] | 276 |
      | lcl|Query\_30951 | KFX36066.1 sugar ABC transporter permease [Geobacillus icigianus] | 292 |
      | lcl|Query\_30952 | KFX36067.1 sugar ABC transporter substrate-binding protein [Geobacillus icigianus] | 441 |
      | lcl|Query\_30953 | KFX36068.1 LacI family DNA-binding transcriptional regulator [Geobacillus icigianus] | 337 |
      | lcl|Query\_30954 | KFX36069.1 transposase [Geobacillus icigianus] | 293 |
      | lcl|Query\_30955 | KFX36071.1 hypothetical protein EP10\_02250 [Geobacillus icigianus] | 157 |
      | lcl|Query\_30956 | KFX36072.1 L,D-transpeptidase [Geobacillus icigianus] | 165 |
      | lcl|Query\_30957 | KFX36073.1 alcohol dehydrogenase [Geobacillus icigianus] | 330 |
      | lcl|Query\_30958 | KFX36074.1 N-acetyltransferase [Geobacillus icigianus] | 150 |
      | lcl|Query\_30959 | KFX36075.1 hypothetical protein EP10\_02275 [Geobacillus icigianus] | 74 |
      | lcl|Query\_30960 | KFX36076.1 zinc ribbon domain-containing protein [Geobacillus icigianus] | 462 |
      | lcl|Query\_30961 | KFX36077.2 zinc ribbon domain-containing protein [Geobacillus icigianus] | 542 |
      | lcl|Query\_30962 | KFX36078.1 zinc ribbon domain-containing protein [Geobacillus icigianus] | 160 |
      | lcl|Query\_30963 | KFX36079.1 LytR family transcriptional regulator [Geobacillus icigianus] | 312 |
      | lcl|Query\_30964 | KFX36080.1 hypothetical protein EP10\_02300 [Geobacillus icigianus] | 122 |
      | lcl|Query\_30965 | KFX36081.1 3-hydroxyacyl-[acyl-carrier-protein] dehydratase FabZ [Geobacillus icigianus] | 145 |
      | lcl|Query\_30966 | KFX36082.1 DNA-directed RNA polymerase subunit beta [Geobacillus icigianus] | 119 |
      | lcl|Query\_30967 | KFX36083.1 flagellar hook-basal body protein [Geobacillus icigianus] | 273 |
      | lcl|Query\_30968 | KFX36084.1 flagellar hook-basal body protein [Geobacillus icigianus] | 276 |
      | lcl|Query\_30969 | KFX36085.1 rod shape-determining protein [Geobacillus icigianus] | 333 |
      | lcl|Query\_30970 | KFX36086.1 sporulation transcriptional regulator SpoIIID [Geobacillus icigianus] | 87 |
      | lcl|Query\_30971 | KFX36087.1 proteinase inhibitor [Geobacillus icigianus] | 169 |
      | lcl|Query\_30972 | KFX36088.1 gamma-glutamyltransferase family protein [Geobacillus icigianus] | 534 |
      | lcl|Query\_30973 | KFX36057.1 DUF1861 domain-containing protein [Geobacillus icigianus] | 323 |
      | lcl|Query\_30974 | KFX36070.1 EamA/RhaT family transporter [Geobacillus icigianus] | 311 |
      | lcl|Query\_30975 | PUA93334.1 DNA primase [Geobacillus icigianus] | 617 |
      | lcl|Query\_30976 | KFX35862.1 hypothetical protein EP10\_02350 [Geobacillus icigianus] | 77 |
      | lcl|Query\_30977 | PUA93335.1 alpha/beta hydrolase [Geobacillus icigianus] | 161 |
      | lcl|Query\_30978 | KFX35864.1 hypothetical protein EP10\_02360 [Geobacillus icigianus] | 172 |
      | lcl|Query\_30979 | KFX35865.1 DNA polymerase B [Geobacillus icigianus] | 562 |
      | lcl|Query\_30980 | KFX35866.1 hypothetical protein EP10\_02370 [Geobacillus icigianus] | 156 |
      | lcl|Query\_30981 | KFX35867.1 alpha/beta hydrolase [Geobacillus icigianus] | 287 |
      | lcl|Query\_30982 | KFX35868.1 endonuclease [Geobacillus icigianus] | 297 |
      | lcl|Query\_30983 | KFX35869.2 ATP-dependent helicase [Geobacillus icigianus] | 349 |
      | lcl|Query\_30984 | KFX35870.1 VRR-NUC domain-containing protein [Geobacillus icigianus] | 105 |
      | lcl|Query\_30985 | PUA93336.1 hypothetical protein EP10\_19320 [Geobacillus icigianus] | 51 |
      | lcl|Query\_30986 | KFX35871.1 DNA-entry nuclease [Geobacillus icigianus] | 89 |
      | lcl|Query\_30987 | KFX35872.1 hypothetical protein EP10\_02410 [Geobacillus icigianus] | 87 |
      | lcl|Query\_30988 | KFX35873.1 hypothetical protein EP10\_02415 [Geobacillus icigianus] | 98 |
      | lcl|Query\_30989 | KFX35874.1 hypothetical protein EP10\_02420 [Geobacillus icigianus] | 61 |
      | lcl|Query\_30990 | KFX35875.1 transcriptional regulator [Geobacillus icigianus] | 72 |
      | lcl|Query\_30991 | KFX35876.1 helix-turn-helix domain-containing protein [Geobacillus icigianus] | 225 |
      | lcl|Query\_30992 | KFX35877.1 recombinase family protein [Geobacillus icigianus] | 462 |
      | lcl|Query\_30993 | KFX35878.1 ABC transporter ATP-binding protein [Geobacillus icigianus] | 273 |
      | lcl|Query\_30994 | KFX35879.1 ABC transporter permease [Geobacillus icigianus] | 269 |
      | lcl|Query\_30995 | KFX35880.1 nucleoside triphosphatase YtkD [Geobacillus icigianus] | 155 |
      | lcl|Query\_30996 | KFX35881.1 hydrolase [Geobacillus icigianus] | 112 |
      | lcl|Query\_30997 | KFX35882.1 hypothetical protein EP10\_02475 [Geobacillus icigianus] | 63 |
      | lcl|Query\_30998 | KFX35883.1 holin [Geobacillus icigianus] | 131 |
      | lcl|Query\_30999 | KFX35884.1 DNA starvation/stationary phase protection protein [Geobacillus icigianus] | 146 |
      | lcl|Query\_31000 | KFX35885.2 YtzI protein [Geobacillus icigianus] | 52 |
      | lcl|Query\_31001 | KFX35886.1 S-ribosylhomocysteine lyase [Geobacillus icigianus] | 158 |
      | lcl|Query\_31002 | KFX35887.1 membrane protein insertion efficiency factor YidD [Geobacillus icigianus] | 77 |
      | lcl|Query\_31003 | KFX35888.1 adhesin [Geobacillus icigianus] | 340 |
      | lcl|Query\_31004 | KFX35889.1 cytochrome ubiquinol oxidase subunit I [Geobacillus icigianus] | 449 |
      | lcl|Query\_31005 | KFX35890.1 hypothetical protein EP10\_02515 [Geobacillus icigianus] | 343 |
      | lcl|Query\_31006 | KFX35891.2 cytochrome c peroxidase [Geobacillus icigianus] | 652 |
      | lcl|Query\_31007 | KFX35892.1 hypothetical protein EP10\_02525 [Geobacillus icigianus] | 692 |
      | lcl|Query\_31008 | KFX35893.1 hypothetical protein EP10\_02535 [Geobacillus icigianus] | 157 |
      | lcl|Query\_31009 | KFX35894.1 DUF2325 domain-containing protein [Geobacillus icigianus] | 96 |
      | lcl|Query\_31010 | PUA93337.1 DUF1540 domain-containing protein [Geobacillus icigianus] | 53 |
      | lcl|Query\_31011 | KFX35895.1 hypothetical protein EP10\_02550 [Geobacillus icigianus] | 64 |
      | lcl|Query\_31012 | KFX35896.1 o-succinylbenzoate--CoA ligase [Geobacillus icigianus] | 493 |
      | lcl|Query\_31013 | KFX35897.1 1,4-dihydroxy-2-naphthoyl-CoA synthase [Geobacillus icigianus] | 272 |
      | lcl|Query\_31014 | KFX35898.1 2-succinyl-6-hydroxy-2,4-cyclohexadiene-1-carboxylate synthase [Geobacillus icigianus] | 274 |
      | lcl|Query\_31015 | KFX35899.1 2-succinyl-5-enolpyruvyl-6-hydroxy-3-cyclohexene-1-carboxylic-acid synthase [Geobacillus icigianus] | 584 |
      | lcl|Query\_31016 | KFX35900.1 isochorismate synthase [Geobacillus icigianus] | 459 |
      | lcl|Query\_31017 | KFX35901.2 1,4-dihydroxy-2-naphthoate polyprenyltransferase [Geobacillus icigianus] | 308 |
      | lcl|Query\_31018 | KFX35902.1 hypothetical protein EP10\_02585 [Geobacillus icigianus] | 269 |
      | lcl|Query\_31019 | KFX35903.1 hypothetical protein EP10\_02595 [Geobacillus icigianus] | 104 |
      | lcl|Query\_31020 | KFX35847.1 pro-sigmaK processing inhibitor BofA [Geobacillus icigianus] | 87 |
      | lcl|Query\_31021 | KFX35849.1 recombination protein RecR [Geobacillus icigianus] | 198 |
      | lcl|Query\_31022 | KFX35850.1 nucleoid-associated protein, YbaB/EbfC family [Geobacillus icigianus] | 108 |
      | lcl|Query\_31023 | KFX35851.1 DNA polymerase III subunit gamma/tau [Geobacillus icigianus] | 559 |
      | lcl|Query\_31024 | KFX35852.2 tRNA adenosine(34) deaminase TadA [Geobacillus icigianus] | 174 |
      | lcl|Query\_31025 | KFX35853.1 LysM peptidoglycan-binding domain-containing protein [Geobacillus icigianus] | 430 |
      | lcl|Query\_31026 | KFX35854.1 serine--tRNA ligase [Geobacillus icigianus] | 424 |
      | lcl|Query\_31027 | KFX35855.1 pyridoxal 5'-phosphate synthase glutaminase subunit PdxT [Geobacillus icigianus] | 195 |
      | lcl|Query\_31028 | KFX35856.1 pyridoxal 5'-phosphate synthase lyase subunit PdxS [Geobacillus icigianus] | 294 |
      | lcl|Query\_31029 | KFX35857.1 D-alanyl-D-alanine carboxypeptidase [Geobacillus icigianus] | 452 |
      | lcl|Query\_31030 | KFX35858.1 IMP dehydrogenase [Geobacillus icigianus] | 488 |
      | lcl|Query\_31031 | KFX35859.1 hypothetical protein EP10\_02675 [Geobacillus icigianus] | 330 |
      | lcl|Query\_31032 | KFX35848.1 DUF2508 domain-containing protein [Geobacillus icigianus] | 83 |
      | lcl|Query\_31033 | KFX35842.1 hypothetical protein EP10\_02695 [Geobacillus icigianus] | 130 |
      | lcl|Query\_31034 | KFX35843.1 hypothetical protein EP10\_02700 [Geobacillus icigianus] | 695 |
      | lcl|Query\_31035 | KFX35844.1 ABC transporter ATP-binding protein [Geobacillus icigianus] | 287 |
      | lcl|Query\_31036 | KFX35845.1 hypothetical protein EP10\_02710 [Geobacillus icigianus] | 212 |
      | lcl|Query\_31037 | KFX35730.1 macrolide ABC transporter ATP-binding protein/permease [Geobacillus icigianus] | 643 |
      | lcl|Query\_31038 | PUA93331.1 hypothetical protein EP10\_19350 [Geobacillus icigianus] | 67 |
      | lcl|Query\_31039 | KFX35732.1 MurR/RpiR family transcriptional regulator [Geobacillus icigianus] | 255 |
      | lcl|Query\_31040 | KFX35733.1 6-phospho-beta-glucosidase [Geobacillus icigianus] | 447 |
      | lcl|Query\_31041 | KFX35734.1 sugar ABC transporter substrate-binding protein [Geobacillus icigianus] | 337 |
      | lcl|Query\_31042 | PUA93332.1 HAMP domain-containing protein [Geobacillus icigianus] | 246 |
      | lcl|Query\_31043 | KFX35735.1 carbohydrate ABC transporter permease [Geobacillus icigianus] | 273 |
      | lcl|Query\_31044 | KFX35736.2 alpha/beta hydrolase [Geobacillus icigianus] | 261 |
      | lcl|Query\_31045 | KFX35737.1 FMN-dependent monooxygenase [Geobacillus icigianus] | 463 |
      | lcl|Query\_31046 | KFX35738.1 hypothetical protein EP10\_02790 [Geobacillus icigianus] | 276 |
      | lcl|Query\_31047 | KFX35739.1 ABC transporter permease [Geobacillus icigianus] | 218 |
      | lcl|Query\_31048 | KFX35740.1 methionine ABC transporter ATP-binding protein [Geobacillus icigianus] | 337 |
      | lcl|Query\_31049 | KFX35741.2 acyl-CoA dehydrogenase [Geobacillus icigianus] | 417 |
      | lcl|Query\_31050 | KFX35742.1 FMN-dependent monooxygenase [Geobacillus icigianus] | 437 |
      | lcl|Query\_31051 | KFX35743.1 LLM class flavin-dependent oxidoreductase [Geobacillus icigianus] | 372 |
      | lcl|Query\_31052 | KFX35744.1 hypothetical protein EP10\_02820 [Geobacillus icigianus] | 119 |
      | lcl|Query\_31053 | KFX35745.1 LysR family transcriptional regulator [Geobacillus icigianus] | 302 |
      | lcl|Query\_31054 | KFX35746.1 FMN reductase (NADPH) [Geobacillus icigianus] | 184 |
      | lcl|Query\_31055 | KFX35749.1 TetR/AcrR family transcriptional regulator [Geobacillus icigianus] | 307 |
      | lcl|Query\_31056 | KFX35750.1 MFS transporter [Geobacillus icigianus] | 535 |
      | lcl|Query\_31057 | KFX35751.1 DUF779 domain-containing protein [Geobacillus icigianus] | 119 |
      | lcl|Query\_31058 | KFX35752.1 hypothetical protein EP10\_02865 [Geobacillus icigianus] | 354 |
      | lcl|Query\_31059 | KFX35753.1 aldehyde dehydrogenase family protein [Geobacillus icigianus] | 506 |
      | lcl|Query\_31060 | KFX35755.1 DUF1284 domain-containing protein [Geobacillus icigianus] | 144 |
      | lcl|Query\_31061 | KFX35756.1 aspartate kinase [Geobacillus icigianus] | 458 |
      | lcl|Query\_31062 | KFX35757.1 hypothetical protein EP10\_02890 [Geobacillus icigianus] | 94 |
      | lcl|Query\_31063 | KFX35758.2 processed acidic surface protein [Geobacillus icigianus] | 304 |
      | lcl|Query\_31064 | KFX35759.1 class D sortase [Geobacillus icigianus] | 219 |
      | lcl|Query\_31065 | KFX35760.1 hypothetical protein EP10\_02905 [Geobacillus icigianus] | 150 |
      | lcl|Query\_31066 | KFX35761.1 hypothetical protein EP10\_02910 [Geobacillus icigianus] | 60 |
      | lcl|Query\_31067 | KFX35762.1 single-stranded DNA-binding protein [Geobacillus icigianus] | 115 |
      | lcl|Query\_31068 | KFX35763.1 XRE family transcriptional regulator [Geobacillus icigianus] | 135 |
      | lcl|Query\_31069 | KFX35764.1 hypothetical protein EP10\_02925 [Geobacillus icigianus] | 161 |
      | lcl|Query\_31070 | KFX35765.1 peptidase P60 [Geobacillus icigianus] | 447 |
      | lcl|Query\_31071 | KFX35766.1 hypothetical protein EP10\_02935 [Geobacillus icigianus] | 259 |
      | lcl|Query\_31072 | KFX35767.1 S-layer protein [Geobacillus icigianus] | 906 |
      | lcl|Query\_31073 | KFX35768.1 tetratricopeptide repeat protein [Geobacillus icigianus] | 161 |
      | lcl|Query\_31074 | KFX35769.1 O-antigen ligase family protein [Geobacillus icigianus] | 530 |
      | lcl|Query\_31075 | KFX35770.1 hypothetical protein EP10\_02960 [Geobacillus icigianus] | 320 |
      | lcl|Query\_31076 | KFX35771.1 P-loop containing nucleoside triphosphate hydrolase [Geobacillus icigianus] | 456 |
      | lcl|Query\_31077 | KFX35772.1 UTP--glucose-1-phosphate uridylyltransferase [Geobacillus icigianus] | 293 |
      | lcl|Query\_31078 | KFX35773.2 flippase [Geobacillus icigianus] | 419 |
      | lcl|Query\_31079 | KFX35774.1 UDP-galactopyranose mutase [Geobacillus icigianus] | 383 |
      | lcl|Query\_31080 | PUA93333.1 hypothetical protein EP10\_19375 [Geobacillus icigianus] | 313 |
      | lcl|Query\_31081 | KFX35747.1 fructokinase [Geobacillus icigianus] | 296 |
      | lcl|Query\_31082 | KFX35754.1 hypothetical protein EP10\_02875 [Geobacillus icigianus] | 186 |
      | lcl|Query\_31083 | KFX35699.2 IS630 family transposase [Geobacillus icigianus] | 205 |
      | lcl|Query\_31084 | KFX35700.1 homocysteine synthase [Geobacillus icigianus] | 434 |
      | lcl|Query\_31085 | KFX35701.1 Asp-tRNA(Asn)/Glu-tRNA(Gln) amidotransferase subunit GatB [Geobacillus icigianus] | 476 |
      | lcl|Query\_31086 | KFX35702.1 Asp-tRNA(Asn)/Glu-tRNA(Gln) amidotransferase subunit GatA [Geobacillus icigianus] | 485 |
      | lcl|Query\_31087 | KFX35703.1 Asp-tRNA(Asn)/Glu-tRNA(Gln) amidotransferase GatCAB subunit C [Geobacillus icigianus] | 96 |
      | lcl|Query\_31088 | KFX35705.1 cytosolic protein [Geobacillus icigianus] | 257 |
      | lcl|Query\_31089 | KFX35706.2 flavin monoamine oxidase family protein [Geobacillus icigianus] | 491 |
      | lcl|Query\_31090 | KFX35707.1 CamS family sex pheromone protein [Geobacillus icigianus] | 407 |
      | lcl|Query\_31091 | KFX35708.1 NAD-dependent DNA ligase LigA [Geobacillus icigianus] | 670 |
      | lcl|Query\_31092 | KFX35710.1 heptaprenylglyceryl phosphate synthase [Geobacillus icigianus] | 239 |
      | lcl|Query\_31093 | KFX35711.1 hypothetical protein EP10\_03050 [Geobacillus icigianus] | 108 |
      | lcl|Query\_31094 | KFX35712.2 DUF3048 domain-containing protein [Geobacillus icigianus] | 342 |
      | lcl|Query\_31095 | KFX35713.1 adenine deaminase [Geobacillus icigianus] | 581 |
      | lcl|Query\_31096 | KFX35714.1 DUF2892 domain-containing protein [Geobacillus icigianus] | 81 |
      | lcl|Query\_31097 | KFX35715.1 phosphoribosylamine--glycine ligase [Geobacillus icigianus] | 430 |
      | lcl|Query\_31098 | KFX35716.1 bifunctional phosphoribosylaminoimidazolecarboxamide formyltransferase/IMP cyclohydrolase PurH [Geobacillus icigianus] | 512 |
      | lcl|Query\_31099 | KFX35717.1 phosphoribosylglycinamide formyltransferase [Geobacillus icigianus] | 210 |
      | lcl|Query\_31100 | KFX35718.1 phosphoribosylformylglycinamidine cyclo-ligase [Geobacillus icigianus] | 346 |
      | lcl|Query\_31101 | KFX35719.1 amidophosphoribosyltransferase [Geobacillus icigianus] | 470 |
      | lcl|Query\_31102 | KFX35720.1 phosphoribosylformylglycinamidine synthase subunit PurL [Geobacillus icigianus] | 743 |
      | lcl|Query\_31103 | KFX35721.1 phosphoribosylformylglycinamidine synthase subunit PurQ [Geobacillus icigianus] | 228 |
      | lcl|Query\_31104 | KFX35722.1 phosphoribosylformylglycinamidine synthase subunit PurS [Geobacillus icigianus] | 84 |
      | lcl|Query\_31105 | KFX35723.1 phosphoribosylaminoimidazolesuccinocarboxamide synthase [Geobacillus icigianus] | 242 |
      | lcl|Query\_31106 | KFX35724.1 adenylosuccinate lyase [Geobacillus icigianus] | 431 |
      | lcl|Query\_31107 | KFX35725.2 5-(carboxyamino)imidazole ribonucleotide synthase [Geobacillus icigianus] | 420 |
      | lcl|Query\_31108 | KFX35726.1 5-(carboxyamino)imidazole ribonucleotide mutase [Geobacillus icigianus] | 162 |
      | lcl|Query\_31109 | KFX35727.1 Hsp20/alpha crystallin family protein [Geobacillus icigianus] | 148 |
      | lcl|Query\_31110 | PUA93330.1 IS630 family transposase, partial [Geobacillus icigianus] | 60 |
      | lcl|Query\_31111 | KFX35704.1 hypothetical protein EP10\_03015 [Geobacillus icigianus] | 168 |
      | lcl|Query\_31112 | KFX35709.1 DNA helicase PcrA [Geobacillus icigianus] | 723 |
      | lcl|Query\_31113 | KFX35684.1 hypothetical protein EP10\_03150, partial [Geobacillus icigianus] | 76 |
      | lcl|Query\_31114 | KFX35685.1 amidohydrolase [Geobacillus icigianus] | 389 |
      | lcl|Query\_31115 | KFX35686.1 GNAT family N-acetyltransferase [Geobacillus icigianus] | 279 |
      | lcl|Query\_31116 | KFX35687.1 o-succinylbenzoate synthase [Geobacillus icigianus] | 374 |
      | lcl|Query\_31117 | KFX35688.1 cation acetate symporter [Geobacillus icigianus] | 511 |
      | lcl|Query\_31118 | KFX35689.1 DUF485 domain-containing protein [Geobacillus icigianus] | 113 |
      | lcl|Query\_31119 | KFX35690.1 amino acid permease [Geobacillus icigianus] | 471 |
      | lcl|Query\_31120 | KFX35691.1 RNA polymerase sigma-I factor [Geobacillus icigianus] | 246 |
      | lcl|Query\_31121 | KFX35692.1 hypothetical protein EP10\_03195 [Geobacillus icigianus] | 344 |
      | lcl|Query\_31122 | KFX35693.1 small acid-soluble spore protein [Geobacillus icigianus] | 66 |
      | lcl|Query\_31123 | KFX35694.1 carbohydrate ABC transporter permease [Geobacillus icigianus] | 275 |
      | lcl|Query\_31124 | KFX35678.1 hypothetical protein EP10\_03255 [Geobacillus icigianus] | 152 |
      | lcl|Query\_31125 | PUA93329.1 spore coat protein, partial [Geobacillus icigianus] | 51 |
      | lcl|Query\_31126 | PUA93328.1 hypothetical protein EP10\_19410 [Geobacillus icigianus] | 164 |
      | lcl|Query\_31127 | KFX35652.1 hypothetical protein EP10\_03280 [Geobacillus icigianus] | 112 |
      | lcl|Query\_31128 | KFX35653.1 hypothetical protein EP10\_03285 [Geobacillus icigianus] | 181 |
      | lcl|Query\_31129 | KFX35654.1 D-2-hydroxyacid dehydrogenase [Geobacillus icigianus] | 310 |
      | lcl|Query\_31130 | KFX35655.1 TerC family protein [Geobacillus icigianus] | 228 |
      | lcl|Query\_31131 | KFX35656.1 LysM peptidoglycan-binding domain-containing protein [Geobacillus icigianus] | 420 |
      | lcl|Query\_31132 | KFX35657.1 histidine ammonia-lyase [Geobacillus icigianus] | 504 |
      | lcl|Query\_31133 | KFX35658.1 transcriptional regulator [Geobacillus icigianus] | 149 |
      | lcl|Query\_31134 | KFX35659.1 hypothetical protein EP10\_03325 [Geobacillus icigianus] | 67 |
      | lcl|Query\_31135 | KFX35660.1 toxin-antitoxin system HicB family antitoxin [Geobacillus icigianus] | 58 |
      | lcl|Query\_31136 | KFX35661.1 xanthine dehydrogenase [Geobacillus icigianus] | 343 |
      | lcl|Query\_31137 | KFX35662.1 xanthine dehydrogenase accessory protein PucB [Geobacillus icigianus] | 217 |
      | lcl|Query\_31138 | KFX35663.1 xanthine dehydrogenase family protein molybdopterin-binding subunit [Geobacillus icigianus] | 775 |
      | lcl|Query\_31139 | KFX35664.1 xanthine dehydrogenase [Geobacillus icigianus] | 278 |
      | lcl|Query\_31140 | KFX35665.1 (2Fe-2S)-binding protein [Geobacillus icigianus] | 160 |
      | lcl|Query\_31141 | KFX35666.1 MerR family DNA-binding transcriptional regulator [Geobacillus icigianus] | 141 |
      | lcl|Query\_31142 | KFX35667.1 MFS transporter [Geobacillus icigianus] | 385 |
      | lcl|Query\_31143 | KFX35668.1 saccharopine dehydrogenase [Geobacillus icigianus] | 384 |
      | lcl|Query\_31144 | KFX35669.1 aldehyde dehydrogenase [Geobacillus icigianus] | 493 |
      | lcl|Query\_31145 | KFX35670.1 hypothetical protein EP10\_03380 [Geobacillus icigianus] | 62 |
      | lcl|Query\_31146 | KFX35671.1 acetolactate synthase [Geobacillus icigianus] | 551 |
      | lcl|Query\_31147 | KFX35672.1 LL-diaminopimelate aminotransferase [Geobacillus icigianus] | 392 |
      | lcl|Query\_31148 | KFX35673.1 hypothetical protein EP10\_03395 [Geobacillus icigianus] | 77 |
      | lcl|Query\_31149 | PUA93326.1 hypothetical protein EP10\_19415 [Geobacillus icigianus] | 57 |
      | lcl|Query\_31150 | KFX35630.1 Crp/Fnr family transcriptional regulator [Geobacillus icigianus] | 228 |
      | lcl|Query\_31151 | KFX35631.1 N-acetyl-gamma-glutamyl-phosphate reductase [Geobacillus icigianus] | 344 |
      | lcl|Query\_31152 | KFX35632.1 bifunctional ornithine acetyltransferase/N-acetylglutamate synthase [Geobacillus icigianus] | 410 |
      | lcl|Query\_31153 | KFX35633.1 acetylglutamate kinase [Geobacillus icigianus] | 261 |
      | lcl|Query\_31154 | KFX35634.1 acetylornithine transaminase [Geobacillus icigianus] | 390 |
      | lcl|Query\_31155 | KFX35635.1 carbamoyl phosphate synthase small subunit [Geobacillus icigianus] | 354 |
      | lcl|Query\_31156 | KFX35636.1 carbamoyl phosphate synthase large subunit [Geobacillus icigianus] | 1042 |
      | lcl|Query\_31157 | KFX35637.1 ornithine carbamoyltransferase [Geobacillus icigianus] | 312 |
      | lcl|Query\_31158 | KFX35638.1 YjzC family protein [Geobacillus icigianus] | 59 |
      | lcl|Query\_31159 | KFX35639.1 ATP-dependent chaperone ClpB [Geobacillus icigianus] | 862 |
      | lcl|Query\_31160 | KFX35640.1 TetR/AcrR family transcriptional regulator [Geobacillus icigianus] | 190 |
      | lcl|Query\_31161 | KFX35641.1 MMPL family transporter [Geobacillus icigianus] | 706 |
      | lcl|Query\_31162 | KFX35642.1 DUF2929 domain-containing protein [Geobacillus icigianus] | 62 |
      | lcl|Query\_31163 | KFX35643.1 hypothetical protein EP10\_03480 [Geobacillus icigianus] | 246 |
      | lcl|Query\_31164 | KFX35644.1 hypothetical protein EP10\_03490 [Geobacillus icigianus] | 82 |
      | lcl|Query\_31165 | KFX35645.1 ketoacyl-ACP synthase III [Geobacillus icigianus] | 310 |
      | lcl|Query\_31166 | KFX35646.1 beta-ketoacyl-[acyl-carrier-protein] synthase II [Geobacillus icigianus] | 412 |
      | lcl|Query\_31167 | PUA93327.1 hypothetical protein EP10\_19425 [Geobacillus icigianus] | 76 |
      | lcl|Query\_31168 | KFX35647.1 hypothetical protein EP10\_03505 [Geobacillus icigianus] | 68 |
      | lcl|Query\_31169 | KFX35648.1 DUF3603 domain-containing protein [Geobacillus icigianus] | 246 |
      | lcl|Query\_31170 | KFX35649.1 tryptophan--tRNA ligase, partial [Geobacillus icigianus] | 206 |
      | lcl|Query\_31171 | PUA93325.1 hypothetical protein EP10\_19430, partial [Geobacillus icigianus] | 444 |
      | lcl|Query\_31172 | PUA93324.1 hypothetical protein EP10\_19435 [Geobacillus icigianus] | 72 |
      | lcl|Query\_31173 | KFX35590.1 MarR family transcriptional regulator, partial [Geobacillus icigianus] | 142 |
      | lcl|Query\_31174 | KFX35589.1 IS630 family transposase, partial [Geobacillus icigianus] | 69 |
      | lcl|Query\_31175 | KFX35587.2 MFS transporter, partial [Geobacillus icigianus] | 257 |
      | lcl|Query\_31176 | KFX35552.1 sporulation protein YhbH [Geobacillus icigianus] | 389 |
      | lcl|Query\_31177 | KFX35553.1 protein prkA [Geobacillus icigianus] | 631 |
      | lcl|Query\_31178 | KFX35554.1 tRNA (uridine(34)/cytosine(34)/5-carboxymethylaminomethyluridine(34)-2'-O)-methyltransferase TrmL [Geobacillus icigianus] | 157 |
      | lcl|Query\_31179 | KFX35555.1 hypothetical protein EP10\_03575 [Geobacillus icigianus] | 287 |
      | lcl|Query\_31180 | KFX35556.1 tRNA epoxyqueuosine(34) reductase QueG [Geobacillus icigianus] | 383 |
      | lcl|Query\_31181 | KFX35557.1 hypothetical protein EP10\_03585 [Geobacillus icigianus] | 221 |
      | lcl|Query\_31182 | KFX35501.1 biotin transporter BioY [Geobacillus icigianus] | 187 |
      | lcl|Query\_31183 | KFX35502.1 dethiobiotin synthase [Geobacillus icigianus] | 238 |
      | lcl|Query\_31184 | KFX35503.1 adenosylmethionine--8-amino-7-oxononanoate transaminase [Geobacillus icigianus] | 459 |
      | lcl|Query\_31185 | KFX35504.1 Ger(x)C family spore germination protein [Geobacillus icigianus] | 355 |
      | lcl|Query\_31186 | KFX35505.1 spore gernimation protein GerB [Geobacillus icigianus] | 362 |
      | lcl|Query\_31187 | KFX35506.1 spore germination protein [Geobacillus icigianus] | 492 |
      | lcl|Query\_31188 | KFX35507.1 spore coat protein [Geobacillus icigianus] | 111 |
      | lcl|Query\_31189 | KFX35508.1 hypothetical protein EP10\_03755 [Geobacillus icigianus] | 85 |
      | lcl|Query\_31190 | PUA93322.1 YuzL family protein [Geobacillus icigianus] | 49 |
      | lcl|Query\_31191 | KFX35510.1 3-hydroxyacyl-CoA dehydrogenase [Geobacillus icigianus] | 795 |
      | lcl|Query\_31192 | KFX35511.1 acetyl-CoA C-acetyltransferase [Geobacillus icigianus] | 390 |
      | lcl|Query\_31193 | KFX35512.1 acyl-CoA dehydrogenase [Geobacillus icigianus] | 594 |
      | lcl|Query\_31194 | KFX35513.1 arsenate reductase family protein [Geobacillus icigianus] | 125 |
      | lcl|Query\_31195 | KFX35514.1 glycine cleavage system protein GcvH [Geobacillus icigianus] | 127 |
      | lcl|Query\_31196 | KFX35515.1 DUF2553 domain-containing protein [Geobacillus icigianus] | 78 |
      | lcl|Query\_31197 | KFX35516.1 hypothetical protein EP10\_03800 [Geobacillus icigianus] | 119 |
      | lcl|Query\_31198 | KFX35517.1 thioredoxin [Geobacillus icigianus] | 104 |
      | lcl|Query\_31199 | KFX35518.1 sterol-binding protein [Geobacillus icigianus] | 115 |
      | lcl|Query\_31200 | KFX35519.1 methionine ABC transporter ATP-binding protein [Geobacillus icigianus] | 342 |
      | lcl|Query\_31201 | KFX35520.1 ABC transporter permease [Geobacillus icigianus] | 222 |
      | lcl|Query\_31202 | KFX35521.1 MetQ/NlpA family ABC transporter substrate-binding protein [Geobacillus icigianus] | 282 |
      | lcl|Query\_31203 | KFX35522.1 carboxymuconolactone decarboxylase family protein [Geobacillus icigianus] | 120 |
      | lcl|Query\_31204 | KFX35523.1 Fe-S cluster assembly ATPase SufC [Geobacillus icigianus] | 259 |
      | lcl|Query\_31205 | KFX35524.1 Fe-S cluster assembly protein SufD [Geobacillus icigianus] | 436 |
      | lcl|Query\_31206 | KFX35525.1 cysteine desulfurase [Geobacillus icigianus] | 406 |
      | lcl|Query\_31207 | KFX35526.1 SUF system NifU family Fe-S cluster assembly protein [Geobacillus icigianus] | 149 |
      | lcl|Query\_31208 | KFX35527.1 Fe-S cluster assembly protein SufB [Geobacillus icigianus] | 465 |
      | lcl|Query\_31209 | KFX35528.2 hypothetical protein EP10\_03860 [Geobacillus icigianus] | 299 |
      | lcl|Query\_31210 | KFX35529.1 LysE family translocator [Geobacillus icigianus] | 210 |
      | lcl|Query\_31211 | KFX35531.1 sulfite exporter TauE/SafE family protein [Geobacillus icigianus] | 295 |
      | lcl|Query\_31212 | KFX35532.1 bifunctional metallophosphatase/5'-nucleotidase [Geobacillus icigianus] | 460 |
      | lcl|Query\_31213 | KFX35533.1 DUF1805 domain-containing protein [Geobacillus icigianus] | 100 |
      | lcl|Query\_31214 | PUA93323.1 ethanolamine utilization protein EutJ, partial [Geobacillus icigianus] | 42 |
      | lcl|Query\_31215 | KFX35509.1 proline dehydrogenase [Geobacillus icigianus] | 305 |
      | lcl|Query\_31216 | KFX35530.1 DUF72 domain-containing protein [Geobacillus icigianus] | 282 |
      | lcl|Query\_31217 | KFX35534.1 sporulation protein YunB [Geobacillus icigianus] | 248 |
      | lcl|Query\_31218 | KFX35498.1 hypothetical protein EP10\_03900 [Geobacillus icigianus] | 102 |
      | lcl|Query\_31219 | KFX35499.1 restriction endonuclease [Geobacillus icigianus] | 312 |
      | lcl|Query\_31220 | PUA93321.1 hypothetical protein EP10\_19450, partial [Geobacillus icigianus] | 380 |
      | lcl|Query\_31221 | KFX35452.1 hypothetical protein EP10\_03920, partial [Geobacillus icigianus] | 191 |
      | lcl|Query\_31222 | KFX35453.1 hypothetical protein EP10\_03925 [Geobacillus icigianus] | 144 |
      | lcl|Query\_31223 | KFX35454.1 ABC transporter substrate-binding protein [Geobacillus icigianus] | 438 |
      | lcl|Query\_31224 | KFX35455.1 ABC transporter permease [Geobacillus icigianus] | 435 |
      | lcl|Query\_31225 | KFX35456.1 carbohydrate ABC transporter permease [Geobacillus icigianus] | 275 |
      | lcl|Query\_31226 | PUA93318.1 hypothetical protein EP10\_19460 [Geobacillus icigianus] | 190 |
      | lcl|Query\_31227 | KFX35457.1 hypothetical protein EP10\_03960 [Geobacillus icigianus] | 420 |
      | lcl|Query\_31228 | KFX35458.1 hypothetical protein EP10\_03965 [Geobacillus icigianus] | 177 |
      | lcl|Query\_31229 | KFX35459.1 ABC transporter permease [Geobacillus icigianus] | 269 |
      | lcl|Query\_31230 | KFX35462.1 LacI family transcriptional regulator [Geobacillus icigianus] | 332 |
      | lcl|Query\_31231 | KFX35463.1 gfo/Idh/MocA family oxidoreductase [Geobacillus icigianus] | 359 |
      | lcl|Query\_31232 | KFX35464.1 trehalose utilization protein ThuA [Geobacillus icigianus] | 249 |
      | lcl|Query\_31233 | KFX35465.1 gfo/Idh/MocA family oxidoreductase [Geobacillus icigianus] | 384 |
      | lcl|Query\_31234 | KFX35466.1 sugar phosphate isomerase/epimerase [Geobacillus icigianus] | 323 |
      | lcl|Query\_31235 | KFX35467.1 hypothetical protein EP10\_04010 [Geobacillus icigianus] | 110 |
      | lcl|Query\_31236 | KFX35468.1 hypothetical protein EP10\_04015 [Geobacillus icigianus] | 153 |
      | lcl|Query\_31237 | KFX35469.1 phosphoribosyl-ATP pyrophosphohydrolase [Geobacillus icigianus] | 108 |
      | lcl|Query\_31238 | KFX35470.1 DNA helicase [Geobacillus icigianus] | 815 |
      | lcl|Query\_31239 | PUA93319.1 hypothetical protein EP10\_19475 [Geobacillus icigianus] | 165 |
      | lcl|Query\_31240 | KFX35460.2 ABC transporter ATP-binding protein [Geobacillus icigianus] | 234 |
      | lcl|Query\_31241 | KFX35461.1 glycosyl transferase family 2 [Geobacillus icigianus] | 1109 |
      | lcl|Query\_31242 | PUA93320.1 hypothetical protein EP10\_19480, partial [Geobacillus icigianus] | 92 |
      | lcl|Query\_31243 | PUA93316.1 hypothetical protein EP10\_19485, partial [Geobacillus icigianus] | 159 |
      | lcl|Query\_31244 | KFX35434.1 cytosolic protein [Geobacillus icigianus] | 134 |
      | lcl|Query\_31245 | PUA93317.1 glutaminase A [Geobacillus icigianus] | 323 |
      | lcl|Query\_31246 | KFX35436.1 8-amino-7-oxononanoate synthase [Geobacillus icigianus] | 390 |
      | lcl|Query\_31247 | KFX35437.1 H-type small acid-soluble spore protein [Geobacillus icigianus] | 61 |
      | lcl|Query\_31248 | KFX35438.2 DUF2324 domain-containing protein [Geobacillus icigianus] | 260 |
      | lcl|Query\_31249 | KFX35439.1 ArsR family transcriptional regulator [Geobacillus icigianus] | 94 |
      | lcl|Query\_31250 | KFX35440.1 4-hydroxyphenylacetate 3-monooxygenase, oxygenase component [Geobacillus icigianus] | 481 |
      | lcl|Query\_31251 | KFX35441.1 sodium/proline symporter [Geobacillus icigianus] | 478 |
      | lcl|Query\_31252 | KFX35442.1 aminopeptidase [Geobacillus icigianus] | 413 |
      | lcl|Query\_31253 | KFX35443.1 tetratricopeptide repeat protein [Geobacillus icigianus] | 936 |
      | lcl|Query\_31254 | KFX35444.1 hypothetical protein EP10\_04115 [Geobacillus icigianus] | 99 |
      | lcl|Query\_31255 | KFX35445.1 hypothetical protein EP10\_04120 [Geobacillus icigianus] | 71 |
      | lcl|Query\_31256 | KFX35323.1 coproporphyrinogen III oxidase [Geobacillus icigianus] | 501 |
      | lcl|Query\_31257 | KFX35324.1 Cof-type HAD-IIB family hydrolase [Geobacillus icigianus] | 286 |
      | lcl|Query\_31258 | KFX35325.1 hypothetical protein EP10\_04175 [Geobacillus icigianus] | 74 |
      | lcl|Query\_31259 | KFX35326.1 YlbF family regulator [Geobacillus icigianus] | 119 |
      | lcl|Query\_31260 | KFX35327.1 DUF445 domain-containing protein [Geobacillus icigianus] | 377 |
      | lcl|Query\_31261 | KFX35328.1 alpha-L-glutamate ligase [Geobacillus icigianus] | 359 |
      | lcl|Query\_31262 | KFX35329.1 hypothetical protein EP10\_04200 [Geobacillus icigianus] | 355 |
      | lcl|Query\_31263 | KFX35330.1 glutathione synthetase [Geobacillus icigianus] | 449 |
      | lcl|Query\_31264 | KFX35331.1 hypothetical protein EP10\_04210 [Geobacillus icigianus] | 388 |
      | lcl|Query\_31265 | KFX35332.1 hypothetical protein EP10\_04215 [Geobacillus icigianus] | 133 |
      | lcl|Query\_31266 | KFX35333.1 hypothetical protein EP10\_04220 [Geobacillus icigianus] | 62 |
      | lcl|Query\_31267 | KFX35335.1 ABC transporter ATP-binding protein [Geobacillus icigianus] | 370 |
      | lcl|Query\_31268 | KFX35336.1 hypothetical protein EP10\_04235 [Geobacillus icigianus] | 80 |
      | lcl|Query\_31269 | KFX35337.1 small acid-soluble spore protein [Geobacillus icigianus] | 70 |
      | lcl|Query\_31270 | KFX35338.1 HlyC/CorC family transporter [Geobacillus icigianus] | 423 |
      | lcl|Query\_31271 | KFX35339.1 thiazole biosynthesis adenylyltransferase ThiF [Geobacillus icigianus] | 342 |
      | lcl|Query\_31272 | KFX35340.1 thiazole synthase [Geobacillus icigianus] | 255 |
      | lcl|Query\_31273 | KFX35341.1 thiamine biosynthesis protein ThiS [Geobacillus icigianus] | 67 |
      | lcl|Query\_31274 | KFX35342.1 glycine oxidase ThiO [Geobacillus icigianus] | 379 |
      | lcl|Query\_31275 | KFX35343.1 thiamine phosphate synthase [Geobacillus icigianus] | 203 |
      | lcl|Query\_31276 | KFX35344.1 energy-coupling factor transporter transmembrane protein EcfT [Geobacillus icigianus] | 260 |
      | lcl|Query\_31277 | KFX35345.1 ABC transporter [Geobacillus icigianus] | 484 |
      | lcl|Query\_31278 | KFX35346.1 thiamine ABC transporter permease [Geobacillus icigianus] | 196 |
      | lcl|Query\_31279 | KFX35347.1 thiaminase II [Geobacillus icigianus] | 227 |
      | lcl|Query\_31280 | KFX35348.1 class I SAM-dependent rRNA methyltransferase [Geobacillus icigianus] | 394 |
      | lcl|Query\_31281 | KFX35349.1 MFS transporter [Geobacillus icigianus] | 401 |
      | lcl|Query\_31282 | KFX35350.1 putative metal-dependent hydrolase [Geobacillus icigianus] | 178 |
      | lcl|Query\_31283 | KFX35351.1 alpha-glucosidase [Geobacillus icigianus] | 555 |
      | lcl|Query\_31284 | KFX35352.1 DUF466 domain-containing protein [Geobacillus icigianus] | 67 |
      | lcl|Query\_31285 | KFX35353.1 carbon starvation protein A [Geobacillus icigianus] | 691 |
      | lcl|Query\_31286 | KFX35354.1 hypothetical protein EP10\_04340 [Geobacillus icigianus] | 154 |
      | lcl|Query\_31287 | KFX35355.1 TerC family protein [Geobacillus icigianus] | 230 |
      | lcl|Query\_31288 | KFX35357.1 cytochrome D ubiquinol oxidase subunit II [Geobacillus icigianus] | 342 |
      | lcl|Query\_31289 | KFX35358.1 cytochrome ubiquinol oxidase subunit I [Geobacillus icigianus] | 448 |
      | lcl|Query\_31290 | KFX35359.1 stage V sporulation protein R [Geobacillus icigianus] | 473 |
      | lcl|Query\_31291 | KFX35360.1 DedA family protein [Geobacillus icigianus] | 201 |
      | lcl|Query\_31292 | KFX35361.1 MFS transporter [Geobacillus icigianus] | 82 |
      | lcl|Query\_31293 | KFX35362.1 hypothetical protein EP10\_04385 [Geobacillus icigianus] | 85 |
      | lcl|Query\_31294 | KFX35363.1 phospho-sugar mutase [Geobacillus icigianus] | 585 |
      | lcl|Query\_31295 | KFX35364.1 CBS domain-containing protein [Geobacillus icigianus] | 148 |
      | lcl|Query\_31296 | KFX35365.1 disulfide bond formation protein B [Geobacillus icigianus] | 145 |
      | lcl|Query\_31297 | KFX35366.1 thiol reductase thioredoxin [Geobacillus icigianus] | 157 |
      | lcl|Query\_31298 | KFX35367.1 hypothetical protein EP10\_04410 [Geobacillus icigianus] | 132 |
      | lcl|Query\_31299 | KFX35368.1 RluA family pseudouridine synthase [Geobacillus icigianus] | 300 |
      | lcl|Query\_31300 | KFX35369.1 HlyC/CorC family transporter [Geobacillus icigianus] | 452 |
      | lcl|Query\_31301 | KFX35370.1 peptidase [Geobacillus icigianus] | 230 |
      | lcl|Query\_31302 | KFX35371.1 aldo/keto reductase [Geobacillus icigianus] | 275 |
      | lcl|Query\_31303 | KFX35372.1 dicarboxylate/amino acid:cation symporter [Geobacillus icigianus] | 420 |
      | lcl|Query\_31304 | KFX35374.1 sensor histidine kinase [Geobacillus icigianus] | 528 |
      | lcl|Query\_31305 | KFX35375.1 HTH domain-containing protein [Geobacillus icigianus] | 226 |
      | lcl|Query\_31306 | KFX35376.1 LacI family DNA-binding transcriptional regulator [Geobacillus icigianus] | 325 |
      | lcl|Query\_31307 | KFX35377.1 hypothetical protein EP10\_04475 [Geobacillus icigianus] | 229 |
      | lcl|Query\_31308 | PUA93315.1 hypothetical protein EP10\_19510 [Geobacillus icigianus] | 86 |
      | lcl|Query\_31309 | KFX35379.1 glucose-6-phosphate isomerase [Geobacillus icigianus] | 449 |
      | lcl|Query\_31310 | KFX35380.1 NADH-dependent alcohol dehydrogenase [Geobacillus icigianus] | 387 |
      | lcl|Query\_31311 | KFX35381.1 DUF378 domain-containing protein [Geobacillus icigianus] | 77 |
      | lcl|Query\_31312 | KFX35382.1 general stress protein 13 [Geobacillus icigianus] | 121 |
      | lcl|Query\_31313 | KFX35383.1 aminotransferase [Geobacillus icigianus] | 390 |
      | lcl|Query\_31314 | KFX35384.1 Lrp/AsnC family transcriptional regulator [Geobacillus icigianus] | 166 |
      | lcl|Query\_31315 | KFX35385.1 DUF1871 domain-containing protein [Geobacillus icigianus] | 88 |
      | lcl|Query\_31316 | KFX35386.1 pyridoxal phosphate-dependent aminotransferase [Geobacillus icigianus] | 386 |
      | lcl|Query\_31317 | KFX35389.1 putative 3'-5' exonuclease KapD [Geobacillus icigianus] | 206 |
      | lcl|Query\_31318 | KFX35390.1 hypothetical protein EP10\_04545 [Geobacillus icigianus] | 148 |
      | lcl|Query\_31319 | KFX35391.1 hypothetical protein EP10\_04550 [Geobacillus icigianus] | 440 |
      | lcl|Query\_31320 | KFX35392.1 hypothetical protein EP10\_04555 [Geobacillus icigianus] | 116 |
      | lcl|Query\_31321 | KFX35393.1 biotin transporter BioY [Geobacillus icigianus] | 201 |
      | lcl|Query\_31322 | KFX35394.1 inorganic phosphate transporter [Geobacillus icigianus] | 332 |
      | lcl|Query\_31323 | KFX35395.1 DUF47 domain-containing protein [Geobacillus icigianus] | 206 |
      | lcl|Query\_31324 | KFX35396.1 cobalamin-binding protein [Geobacillus icigianus] | 269 |
      | lcl|Query\_31325 | KFX35398.1 hypothetical protein EP10\_04605 [Geobacillus icigianus] | 77 |
      | lcl|Query\_31326 | KFX35399.1 hypothetical protein EP10\_04610 [Geobacillus icigianus] | 107 |
      | lcl|Query\_31327 | KFX35400.1 NUDIX domain-containing protein [Geobacillus icigianus] | 167 |
      | lcl|Query\_31328 | KFX35403.1 NAD(P)/FAD-dependent oxidoreductase [Geobacillus icigianus] | 330 |
      | lcl|Query\_31329 | KFX35404.1 DUF1049 domain-containing protein [Geobacillus icigianus] | 103 |
      | lcl|Query\_31330 | KFX35405.1 iron-sulfur cluster assembly accessory protein [Geobacillus icigianus] | 121 |
      | lcl|Query\_31331 | KFX35407.1 DUF1450 domain-containing protein [Geobacillus icigianus] | 79 |
      | lcl|Query\_31332 | KFX35408.1 NAD(P)/FAD-dependent oxidoreductase [Geobacillus icigianus] | 356 |
      | lcl|Query\_31333 | KFX35409.1 DUF1462 domain-containing protein [Geobacillus icigianus] | 109 |
      | lcl|Query\_31334 | KFX35411.1 homoserine kinase [Geobacillus icigianus] | 304 |
      | lcl|Query\_31335 | KFX35412.1 threonine synthase [Geobacillus icigianus] | 353 |
      | lcl|Query\_31336 | KFX35413.1 homoserine dehydrogenase [Geobacillus icigianus] | 432 |
      | lcl|Query\_31337 | KFX35414.1 D-glycerate dehydrogenase [Geobacillus icigianus] | 324 |
      | lcl|Query\_31338 | KFX35415.1 spore coat protein YutH [Geobacillus icigianus] | 333 |
      | lcl|Query\_31339 | KFX35416.1 hypothetical protein EP10\_04695 [Geobacillus icigianus] | 60 |
      | lcl|Query\_31340 | KFX35417.1 phosphatidylglycerophosphatase A [Geobacillus icigianus] | 165 |
      | lcl|Query\_31341 | KFX35419.1 DUF86 domain-containing protein [Geobacillus icigianus] | 145 |
      | lcl|Query\_31342 | KFX35420.1 DUF3055 domain-containing protein [Geobacillus icigianus] | 89 |
      | lcl|Query\_31343 | KFX35421.1 cytosolic protein [Geobacillus icigianus] | 94 |
      | lcl|Query\_31344 | KFX35422.1 DUF1027 domain-containing protein [Geobacillus icigianus] | 91 |
      | lcl|Query\_31345 | KFX35423.1 sporulation protein [Geobacillus icigianus] | 228 |
      | lcl|Query\_31346 | KFX35424.1 lipoyl synthase [Geobacillus icigianus] | 298 |
      | lcl|Query\_31347 | KFX35425.1 ABC transporter ATP-binding protein [Geobacillus icigianus] | 235 |
      | lcl|Query\_31348 | KFX35426.1 ABC transporter ATP-binding protein [Geobacillus icigianus] | 259 |
      | lcl|Query\_31349 | KFX35427.1 branched-chain amino acid ABC transporter permease [Geobacillus icigianus] | 323 |
      | lcl|Query\_31350 | KFX35428.1 branched-chain amino acid ABC transporter permease [Geobacillus icigianus] | 292 |
      | lcl|Query\_31351 | KFX35429.1 ethanolamine utilization protein EutJ, partial [Geobacillus icigianus] | 351 |
      | lcl|Query\_31352 | KFX35322.1 hypothetical protein EP10\_04155 [Geobacillus icigianus] | 61 |
      | lcl|Query\_31353 | KFX35334.1 PucR family transcriptional regulator [Geobacillus icigianus] | 296 |
      | lcl|Query\_31354 | KFX35356.1 DUF1232 domain-containing protein [Geobacillus icigianus] | 90 |
      | lcl|Query\_31355 | KFX35373.1 C4-dicarboxylate ABC transporter [Geobacillus icigianus] | 347 |
      | lcl|Query\_31356 | KFX35387.1 superoxide dismutase family protein [Geobacillus icigianus] | 164 |
      | lcl|Query\_31357 | KFX35388.2 hypothetical protein EP10\_04535 [Geobacillus icigianus] | 71 |
      | lcl|Query\_31358 | KFX35397.2 hypothetical protein EP10\_04600 [Geobacillus icigianus] | 200 |
      | lcl|Query\_31359 | KFX35402.1 NAD(P)/FAD-dependent oxidoreductase [Geobacillus icigianus] | 407 |
      | lcl|Query\_31360 | KFX35406.1 diaminopimelate epimerase [Geobacillus icigianus] | 290 |
      | lcl|Query\_31361 | KFX35410.1 NifU family protein [Geobacillus icigianus] | 78 |
      | lcl|Query\_31362 | KFX35418.1 TIGR01457 family HAD-type hydrolase [Geobacillus icigianus] | 256 |
      | lcl|Query\_31363 | KFX35241.1 transposase [Geobacillus icigianus] | 302 |
      | lcl|Query\_31364 | KFX35242.1 CRISPR-associated endonuclease Cas2 [Geobacillus icigianus] | 102 |
      | lcl|Query\_31365 | KFX35243.1 type II CRISPR-associated endonuclease Cas1 [Geobacillus icigianus] | 299 |
      | lcl|Query\_31366 | KFX35244.1 type II CRISPR RNA-guided endonuclease Cas9 [Geobacillus icigianus] | 1087 |
      | lcl|Query\_31367 | KFX35246.1 MFS transporter [Geobacillus icigianus] | 412 |
      | lcl|Query\_31368 | PUA93314.1 PspA/IM30 family protein [Geobacillus icigianus] | 253 |
      | lcl|Query\_31369 | KFX35248.1 IS701 family transposase, partial [Geobacillus icigianus] | 115 |
      | lcl|Query\_31370 | KFX35061.1 hypothetical protein EP10\_04825 [Geobacillus icigianus] | 115 |
      | lcl|Query\_31371 | KFX35062.1 cystathionine beta-lyase MetC [Geobacillus icigianus] | 392 |
      | lcl|Query\_31372 | KFX35063.1 methionine biosynthesis PLP-dependent protein [Geobacillus icigianus] | 367 |
      | lcl|Query\_31373 | KFX35064.1 esterase family protein [Geobacillus icigianus] | 242 |
      | lcl|Query\_31374 | KFX35065.1 hypothetical protein EP10\_04850 [Geobacillus icigianus] | 173 |
      | lcl|Query\_31375 | KFX35066.1 GNAT family N-acetyltransferase [Geobacillus icigianus] | 144 |
      | lcl|Query\_31376 | KFX35067.1 sporulation protein [Geobacillus icigianus] | 85 |
      | lcl|Query\_31377 | PUA93312.1 YjcZ family sporulation protein [Geobacillus icigianus] | 43 |
      | lcl|Query\_31378 | KFX35068.1 hypothetical protein EP10\_04870 [Geobacillus icigianus] | 78 |
      | lcl|Query\_31379 | KFX35069.1 thioredoxin [Geobacillus icigianus] | 106 |
      | lcl|Query\_31380 | KFX35070.1 DUF421 domain-containing protein [Geobacillus icigianus] | 286 |
      | lcl|Query\_31381 | KFX35071.1 DUF1657 domain-containing protein [Geobacillus icigianus] | 68 |
      | lcl|Query\_31382 | KFX35072.1 stage V sporulation protein AE [Geobacillus icigianus] | 118 |
      | lcl|Query\_31383 | KFX35073.1 stage V sporulation protein AD [Geobacillus icigianus] | 337 |
      | lcl|Query\_31384 | KFX35074.1 stage V sporulation protein AC [Geobacillus icigianus] | 159 |
      | lcl|Query\_31385 | KFX35075.1 sporulation protein [Geobacillus icigianus] | 156 |
      | lcl|Query\_31386 | KFX35076.1 DUF1657 domain-containing protein [Geobacillus icigianus] | 68 |
      | lcl|Query\_31387 | KFX35077.1 holin [Geobacillus icigianus] | 67 |
      | lcl|Query\_31388 | KFX35078.1 hypothetical protein EP10\_04925 [Geobacillus icigianus] | 73 |
      | lcl|Query\_31389 | KFX35080.1 hypothetical protein EP10\_04935 [Geobacillus icigianus] | 240 |
      | lcl|Query\_31390 | KFX35081.1 hypothetical protein EP10\_04945 [Geobacillus icigianus] | 243 |
      | lcl|Query\_31391 | KFX35082.1 hypothetical protein EP10\_04950 [Geobacillus icigianus] | 88 |
      | lcl|Query\_31392 | KFX35084.2 DUF1360 domain-containing protein [Geobacillus icigianus] | 119 |
      | lcl|Query\_31393 | PUA93313.1 hypothetical protein EP10\_19540 [Geobacillus icigianus] | 135 |
      | lcl|Query\_31394 | KFX35085.1 enoyl-[acyl-carrier-protein] reductase FabI [Geobacillus icigianus] | 258 |
      | lcl|Query\_31395 | KFX35086.1 FtsW/RodA/SpoVE family cell cycle protein [Geobacillus icigianus] | 391 |
      | lcl|Query\_31396 | KFX35087.1 hypothetical protein EP10\_04990 [Geobacillus icigianus] | 71 |
      | lcl|Query\_31397 | KFX35088.1 bis(5'-nucleosyl)-tetraphosphatase PrpE [Geobacillus icigianus] | 245 |
      | lcl|Query\_31398 | KFX35089.1 RluA family pseudouridine synthase [Geobacillus icigianus] | 299 |
      | lcl|Query\_31399 | KFX35090.1 NAD kinase [Geobacillus icigianus] | 271 |
      | lcl|Query\_31400 | KFX35092.1 hypothetical protein EP10\_05015 [Geobacillus icigianus] | 129 |
      | lcl|Query\_31401 | KFX35093.1 CYTH domain-containing protein [Geobacillus icigianus] | 196 |
      | lcl|Query\_31402 | KFX35094.1 lytic transglycosylase domain-containing protein [Geobacillus icigianus] | 198 |
      | lcl|Query\_31403 | KFX35095.1 globin [Geobacillus icigianus] | 137 |
      | lcl|Query\_31404 | KFX35096.1 DsbA family protein [Geobacillus icigianus] | 296 |
      | lcl|Query\_31405 | KFX35097.1 hypothetical protein EP10\_05045 [Geobacillus icigianus] | 65 |
      | lcl|Query\_31406 | KFX35098.1 oligoendopeptidase F [Geobacillus icigianus] | 606 |
      | lcl|Query\_31407 | KFX35100.1 cardiolipin synthase [Geobacillus icigianus] | 502 |
      | lcl|Query\_31408 | KFX35101.1 adaptor protein MecA [Geobacillus icigianus] | 226 |
      | lcl|Query\_31409 | KFX35102.1 TerC family protein [Geobacillus icigianus] | 219 |
      | lcl|Query\_31410 | KFX35103.1 transcriptional regulator Spx [Geobacillus icigianus] | 132 |
      | lcl|Query\_31411 | KFX35104.1 hypothetical protein EP10\_05080 [Geobacillus icigianus] | 60 |
      | lcl|Query\_31412 | KFX35105.1 ABC transporter ATP-binding protein [Geobacillus icigianus] | 311 |
      | lcl|Query\_31413 | KFX35106.1 ABC transporter ATP-binding protein [Geobacillus icigianus] | 353 |
      | lcl|Query\_31414 | KFX35107.1 ABC transporter permease [Geobacillus icigianus] | 339 |
      | lcl|Query\_31415 | KFX35108.1 ABC transporter permease [Geobacillus icigianus] | 309 |
      | lcl|Query\_31416 | KFX35109.1 peptide ABC transporter substrate-binding protein [Geobacillus icigianus] | 549 |
      | lcl|Query\_31417 | KFX35079.2 spore coat protein regulator protein YlbO [Geobacillus icigianus] | 86 |
      | lcl|Query\_31418 | KFX35083.1 spore coat protein [Geobacillus icigianus] | 150 |
      | lcl|Query\_31419 | KFX35091.1 GTP pyrophosphokinase family protein [Geobacillus icigianus] | 212 |
      | lcl|Query\_31420 | KFX35099.1 competence protein CoiA [Geobacillus icigianus] | 429 |
      | lcl|Query\_31421 | KFX35042.2 hypothetical protein EP10\_05120 [Geobacillus icigianus] | 67 |
      | lcl|Query\_31422 | KFX35041.2 IS1634 family transposase [Geobacillus icigianus] | 460 |
      | lcl|Query\_31423 | PUA93309.1 hypothetical protein EP10\_19545, partial [Geobacillus icigianus] | 101 |
      | lcl|Query\_31424 | KFX35006.1 glycosyltransferase family 8 protein [Geobacillus icigianus] | 276 |
      | lcl|Query\_31425 | KFX35007.1 YfhD family protein [Geobacillus icigianus] | 61 |
      | lcl|Query\_31426 | PUA93310.1 YfhE family protein [Geobacillus icigianus] | 42 |
      | lcl|Query\_31427 | KFX35008.1 N-acetyltransferase [Geobacillus icigianus] | 184 |
      | lcl|Query\_31428 | KFX35009.1 TIGR01777 family protein [Geobacillus icigianus] | 306 |
      | lcl|Query\_31429 | KFX35010.1 recombination regulator RecX [Geobacillus icigianus] | 266 |
      | lcl|Query\_31430 | KFX35011.1 DUF1811 domain-containing protein [Geobacillus icigianus] | 113 |
      | lcl|Query\_31431 | PUA93311.1 YpzG family protein [Geobacillus icigianus] | 53 |
      | lcl|Query\_31432 | KFX35012.1 small, acid-soluble spore protein K [Geobacillus icigianus] | 53 |
      | lcl|Query\_31433 | KFX35013.1 DUF1641 domain-containing protein [Geobacillus icigianus] | 163 |
      | lcl|Query\_31434 | KFX35014.1 formate dehydrogenase subunit alpha [Geobacillus icigianus] | 987 |
      | lcl|Query\_31435 | KFX35015.1 DUF2294 domain-containing protein [Geobacillus icigianus] | 115 |
      | lcl|Query\_31436 | KFX35016.1 formate dehydrogenase accessory sulfurtransferase FdhD [Geobacillus icigianus] | 263 |
      | lcl|Query\_31437 | KFX35017.1 A/G-specific adenine glycosylase [Geobacillus icigianus] | 366 |
      | lcl|Query\_31438 | KFX35018.1 cytosolic protein [Geobacillus icigianus] | 74 |
      | lcl|Query\_31439 | KFX35019.1 enoyl-[acyl-carrier-protein] reductase FabL [Geobacillus icigianus] | 249 |
      | lcl|Query\_31440 | KFX35020.1 gamma-type small acid-soluble spore protein [Geobacillus icigianus] | 63 |
      | lcl|Query\_31441 | KFX35021.1 hypothetical protein EP10\_05230 [Geobacillus icigianus] | 76 |
      | lcl|Query\_31442 | KFX35022.1 DUF402 domain-containing protein [Geobacillus icigianus] | 177 |
      | lcl|Query\_31443 | KFX35023.1 aromatic acid exporter family protein [Geobacillus icigianus] | 359 |
      | lcl|Query\_31444 | KFX35024.2 glutamate synthase [Geobacillus icigianus] | 352 |
      | lcl|Query\_31445 | KFX35025.1 hypothetical protein EP10\_05250 [Geobacillus icigianus] | 136 |
      | lcl|Query\_31446 | KFX35026.1 glutamate-1-semialdehyde 2,1-aminomutase [Geobacillus icigianus] | 428 |
      | lcl|Query\_31447 | KFX35027.1 daunorubicin ABC transporter ATP-binding protein [Geobacillus icigianus] | 334 |
      | lcl|Query\_31448 | KFX35028.1 daunorubicin ABC transporter permease [Geobacillus icigianus] | 263 |
      | lcl|Query\_31449 | KFX35029.1 ABC transporter permease [Geobacillus icigianus] | 261 |
      | lcl|Query\_31450 | KFX35030.1 L-lactate dehydrogenase [Geobacillus icigianus] | 317 |
      | lcl|Query\_31451 | KFX35031.1 two pore domain potassium channel family protein [Geobacillus icigianus] | 134 |
      | lcl|Query\_31452 | KFX35032.1 thioredoxin-dependent thiol peroxidase [Geobacillus icigianus] | 158 |
      | lcl|Query\_31453 | KFX34852.1 late competence protein ComER [Geobacillus icigianus] | 273 |
      | lcl|Query\_31454 | KFX34854.1 ComE operon protein 2 [Geobacillus icigianus] | 156 |
      | lcl|Query\_31455 | KFX34855.1 DNA internalization-related competence protein ComEC/Rec2 [Geobacillus icigianus] | 749 |
      | lcl|Query\_31456 | KFX34856.1 YqzM family protein [Geobacillus icigianus] | 44 |
      | lcl|Query\_31457 | KFX34857.1 DNA polymerase III subunit delta [Geobacillus icigianus] | 346 |
      | lcl|Query\_31458 | KFX34859.1 GPR endopeptidase [Geobacillus icigianus] | 372 |
      | lcl|Query\_31459 | KFX34860.1 stage II sporulation protein P [Geobacillus icigianus] | 399 |
      | lcl|Query\_31460 | KFX34861.1 DUF3679 domain-containing protein [Geobacillus icigianus] | 112 |
      | lcl|Query\_31461 | KFX34862.1 elongation factor 4 [Geobacillus icigianus] | 609 |
      | lcl|Query\_31462 | KFX34863.1 oxygen-independent coproporphyrinogen III oxidase [Geobacillus icigianus] | 379 |
      | lcl|Query\_31463 | KFX34864.1 heat-inducible transcriptional repressor HrcA [Geobacillus icigianus] | 344 |
      | lcl|Query\_31464 | KFX34865.1 nucleotide exchange factor GrpE [Geobacillus icigianus] | 217 |
      | lcl|Query\_31465 | KFX34866.1 molecular chaperone DnaK [Geobacillus icigianus] | 601 |
      | lcl|Query\_31466 | KFX34867.1 molecular chaperone DnaJ [Geobacillus icigianus] | 387 |
      | lcl|Query\_31467 | KFX34868.1 50S ribosomal protein L11 methyltransferase [Geobacillus icigianus] | 312 |
      | lcl|Query\_31468 | KFX34869.1 16S rRNA (uracil(1498)-N(3))-methyltransferase [Geobacillus icigianus] | 250 |
      | lcl|Query\_31469 | KFX34870.1 tRNA (N(6)-L-threonylcarbamoyladenosine(37)-C(2))-methylthiotransferase MtaB [Geobacillus icigianus] | 449 |
      | lcl|Query\_31470 | KFX34871.1 deoxyribose-phosphate aldolase [Geobacillus icigianus] | 223 |
      | lcl|Query\_31471 | KFX34872.1 Na/Pi cotransporter family protein [Geobacillus icigianus] | 309 |
      | lcl|Query\_31472 | KFX34873.1 30S ribosomal protein S21 [Geobacillus icigianus] | 57 |
      | lcl|Query\_31473 | KFX34874.1 GatB/YqeY domain-containing protein [Geobacillus icigianus] | 148 |
      | lcl|Query\_31474 | KFX34875.1 sporulation protein YqfC [Geobacillus icigianus] | 94 |
      | lcl|Query\_31475 | KFX34876.1 sporulation protein YqfD [Geobacillus icigianus] | 395 |
      | lcl|Query\_31476 | KFX34877.1 PhoH family protein [Geobacillus icigianus] | 320 |
      | lcl|Query\_31477 | KFX34878.1 HDIG domain-containing protein [Geobacillus icigianus] | 700 |
      | lcl|Query\_31478 | KFX34879.1 rRNA maturation RNase YbeY [Geobacillus icigianus] | 156 |
      | lcl|Query\_31479 | KFX34880.1 diacylglycerol kinase family protein [Geobacillus icigianus] | 117 |
      | lcl|Query\_31480 | KFX34881.1 cytidine deaminase [Geobacillus icigianus] | 132 |
      | lcl|Query\_31481 | KFX34882.1 GTPase Era [Geobacillus icigianus] | 302 |
      | lcl|Query\_31482 | PUA93307.1 YqzL family protein [Geobacillus icigianus] | 47 |
      | lcl|Query\_31483 | KFX34883.1 DNA repair protein RecO [Geobacillus icigianus] | 262 |
      | lcl|Query\_31484 | KFX34884.1 transcriptional regulator [Geobacillus icigianus] | 198 |
      | lcl|Query\_31485 | KFX34885.1 kinase/pyrophosphorylase [Geobacillus icigianus] | 266 |
      | lcl|Query\_31486 | KFX34886.1 DNA primase [Geobacillus icigianus] | 599 |
      | lcl|Query\_31487 | KFX34887.1 RNA polymerase sigma factor RpoD [Geobacillus icigianus] | 375 |
      | lcl|Query\_31488 | KFX34888.1 hypothetical protein EP10\_05480 [Geobacillus icigianus] | 171 |
      | lcl|Query\_31489 | KFX34889.1 cytochrome c [Geobacillus icigianus] | 120 |
      | lcl|Query\_31490 | KFX34890.1 tRNA (adenine-N(1))-methyltransferase [Geobacillus icigianus] | 234 |
      | lcl|Query\_31491 | KFX34891.1 Nif3-like dinuclear metal center hexameric protein [Geobacillus icigianus] | 373 |
      | lcl|Query\_31492 | KFX34892.1 4-hydroxy-3-methylbut-2-enyl diphosphate reductase [Geobacillus icigianus] | 316 |
      | lcl|Query\_31493 | KFX34894.1 ATP-dependent helicase [Geobacillus icigianus] | 436 |
      | lcl|Query\_31494 | KFX34895.1 deoxyribonuclease IV [Geobacillus icigianus] | 299 |
      | lcl|Query\_31495 | KFX34896.1 DUF2624 domain-containing protein [Geobacillus icigianus] | 86 |
      | lcl|Query\_31496 | KFX34898.1 metal ABC transporter ATP-binding protein [Geobacillus icigianus] | 251 |
      | lcl|Query\_31497 | KFX34899.1 metal ABC transporter permease [Geobacillus icigianus] | 276 |
      | lcl|Query\_31498 | KFX34900.1 transcriptional repressor [Geobacillus icigianus] | 139 |
      | lcl|Query\_31499 | KFX34901.1 hypothetical protein EP10\_05545 [Geobacillus icigianus] | 190 |
      | lcl|Query\_31500 | KFX34902.1 hypothetical protein EP10\_05550 [Geobacillus icigianus] | 115 |
      | lcl|Query\_31501 | KFX34903.1 flavodoxin-dependent (E)-4-hydroxy-3-methylbut-2-enyl-diphosphate synthase [Geobacillus icigianus] | 376 |
      | lcl|Query\_31502 | KFX34904.1 hypothetical protein EP10\_05560 [Geobacillus icigianus] | 104 |
      | lcl|Query\_31503 | KFX34905.1 phosphate ABC transporter substrate-binding protein PstS family protein [Geobacillus icigianus] | 299 |
      | lcl|Query\_31504 | KFX34906.1 phosphate ABC transporter permease subunit PstC [Geobacillus icigianus] | 298 |
      | lcl|Query\_31505 | KFX34907.1 phosphate ABC transporter permease PtsA [Geobacillus icigianus] | 307 |
      | lcl|Query\_31506 | KFX34910.1 superoxide dismutase [Geobacillus icigianus] | 204 |
      | lcl|Query\_31507 | KFX34911.1 MFS transporter [Geobacillus icigianus] | 430 |
      | lcl|Query\_31508 | PUA93308.1 penicillin-binding protein 2 [Geobacillus icigianus] | 697 |
      | lcl|Query\_31509 | KFX34912.1 phosphate ABC transporter ATP-binding protein [Geobacillus icigianus] | 272 |
      | lcl|Query\_31510 | KFX34913.1 aminodeoxychorismate lyase [Geobacillus icigianus] | 145 |
      | lcl|Query\_31511 | KFX34914.1 hypothetical protein EP10\_05620 [Geobacillus icigianus] | 116 |
      | lcl|Query\_31512 | KFX34915.1 50S ribosomal protein L33 [Geobacillus icigianus] | 49 |
      | lcl|Query\_31513 | KFX34916.1 5-formyltetrahydrofolate cyclo-ligase [Geobacillus icigianus] | 189 |
      | lcl|Query\_31514 | KFX34917.1 hypothetical protein EP10\_05640 [Geobacillus icigianus] | 59 |
      | lcl|Query\_31515 | KFX34921.1 DUF910 domain-containing protein [Geobacillus icigianus] | 70 |
      | lcl|Query\_31516 | KFX34922.1 glucokinase [Geobacillus icigianus] | 317 |
      | lcl|Query\_31517 | KFX34923.1 peptidase M14 [Geobacillus icigianus] | 387 |
      | lcl|Query\_31518 | KFX34924.1 DUF2759 domain-containing protein [Geobacillus icigianus] | 58 |
      | lcl|Query\_31519 | KFX34925.1 MBL fold metallo-hydrolase [Geobacillus icigianus] | 210 |
      | lcl|Query\_31520 | KFX34926.1 cytosolic protein [Geobacillus icigianus] | 375 |
      | lcl|Query\_31521 | KFX34927.1 DUF2626 domain-containing protein [Geobacillus icigianus] | 80 |
      | lcl|Query\_31522 | KFX34928.1 transcriptional regulator [Geobacillus icigianus] | 229 |
      | lcl|Query\_31523 | KFX34929.2 type II/IV secretion system protein [Geobacillus icigianus] | 401 |
      | lcl|Query\_31524 | KFX34930.1 type II secretion system F family protein [Geobacillus icigianus] | 342 |
      | lcl|Query\_31525 | KFX34931.1 prepilin-type N-terminal cleavage/methylation domain-containing protein [Geobacillus icigianus] | 98 |
      | lcl|Query\_31526 | KFX34932.1 prepilin-type N-terminal cleavage/methylation domain-containing protein [Geobacillus icigianus] | 147 |
      | lcl|Query\_31527 | KFX34933.1 competence protein ComG [Geobacillus icigianus] | 108 |
      | lcl|Query\_31528 | KFX34934.2 competence protein ComGF [Geobacillus icigianus] | 160 |
      | lcl|Query\_31529 | KFX34935.1 hypothetical protein EP10\_05730 [Geobacillus icigianus] | 129 |
      | lcl|Query\_31530 | KFX34936.1 YqzE family protein [Geobacillus icigianus] | 60 |
      | lcl|Query\_31531 | KFX34937.1 hypothetical protein EP10\_05740 [Geobacillus icigianus] | 264 |
      | lcl|Query\_31532 | KFX34938.1 ATP-dependent helicase [Geobacillus icigianus] | 554 |
      | lcl|Query\_31533 | KFX34939.1 glycine cleavage system aminomethyltransferase GcvT [Geobacillus icigianus] | 364 |
      | lcl|Query\_31534 | KFX34940.1 aminomethyl-transferring glycine dehydrogenase subunit GcvPA [Geobacillus icigianus] | 448 |
      | lcl|Query\_31535 | KFX34942.1 hypothetical protein EP10\_05765 [Geobacillus icigianus] | 62 |
      | lcl|Query\_31536 | KFX34943.1 ROK family transcriptional regulator [Geobacillus icigianus] | 404 |
      | lcl|Query\_31537 | KFX34944.1 rhodanese-like domain-containing protein [Geobacillus icigianus] | 124 |
      | lcl|Query\_31538 | KFX34945.1 lipoate--protein ligase family protein [Geobacillus icigianus] | 278 |
      | lcl|Query\_31539 | KFX34853.1 competence protein ComEA [Geobacillus icigianus] | 209 |
      | lcl|Query\_31540 | KFX34858.1 30S ribosomal protein S20 [Geobacillus icigianus] | 89 |
      | lcl|Query\_31541 | KFX34893.1 hypothetical protein EP10\_05505 [Geobacillus icigianus] | 176 |
      | lcl|Query\_31542 | KFX34897.1 YitT family protein [Geobacillus icigianus] | 293 |
      | lcl|Query\_31543 | KFX34908.1 DUF1189 domain-containing protein [Geobacillus icigianus] | 257 |
      | lcl|Query\_31544 | KFX34918.1 rhomboid family intramembrane serine protease [Geobacillus icigianus] | 386 |
      | lcl|Query\_31545 | KFX34919.2 hypothetical protein EP10\_05650 [Geobacillus icigianus] | 281 |
      | lcl|Query\_31546 | KFX34941.1 glycine dehydrogenase (aminomethyl-transferring) [Geobacillus icigianus] | 490 |
      | lcl|Query\_31547 | KFX34829.1 hypothetical protein EP10\_05790, partial [Geobacillus icigianus] | 281 |
      | lcl|Query\_31548 | PUA93306.1 hypothetical protein EP10\_19580, partial [Geobacillus icigianus] | 84 |
      | lcl|Query\_31549 | KFX34795.1 glycosyl transferase [Geobacillus icigianus] | 366 |
      | lcl|Query\_31550 | KFX34796.1 DUF4004 domain-containing protein [Geobacillus icigianus] | 209 |
      | lcl|Query\_31551 | KFX34797.1 bactofilin [Geobacillus icigianus] | 234 |
      | lcl|Query\_31552 | KFX34798.1 bactofilin [Geobacillus icigianus] | 240 |
      | lcl|Query\_31553 | KFX34799.1 MFS transporter [Geobacillus icigianus] | 503 |
      | lcl|Query\_31554 | KFX34800.1 acyl-CoA desaturase [Geobacillus icigianus] | 372 |
      | lcl|Query\_31555 | KFX34802.1 Cof-type HAD-IIB family hydrolase [Geobacillus icigianus] | 286 |
      | lcl|Query\_31556 | KFX34803.1 histidine phosphatase family protein [Geobacillus icigianus] | 136 |
      | lcl|Query\_31557 | KFX34804.1 TIGR02221 family CRISPR-associated protein [Geobacillus icigianus] | 441 |
      | lcl|Query\_31558 | KFX34806.1 type III-B CRISPR-associated protein Cas10/Cmr2 [Geobacillus icigianus] | 598 |
      | lcl|Query\_31559 | KFX34807.1 type III-B CRISPR module-associated protein Cmr3 [Geobacillus icigianus] | 389 |
      | lcl|Query\_31560 | KFX34808.1 hypothetical protein EP10\_05885 [Geobacillus icigianus] | 168 |
      | lcl|Query\_31561 | KFX34809.1 type III-B CRISPR module RAMP protein Cmr4 [Geobacillus icigianus] | 329 |
      | lcl|Query\_31562 | KFX34810.2 type III-B CRISPR module-associated protein Cmr5 [Geobacillus icigianus] | 171 |
      | lcl|Query\_31563 | KFX34811.1 type III-B CRISPR module RAMP protein Cmr6 [Geobacillus icigianus] | 294 |
      | lcl|Query\_31564 | KFX34812.1 type I 3-dehydroquinate dehydratase [Geobacillus icigianus] | 257 |
      | lcl|Query\_31565 | KFX34813.1 shikimate kinase [Geobacillus icigianus] | 188 |
      | lcl|Query\_31566 | KFX34814.1 hypothetical protein EP10\_05930 [Geobacillus icigianus] | 110 |
      | lcl|Query\_31567 | KFX34816.1 hypothetical protein EP10\_05940 [Geobacillus icigianus] | 136 |
      | lcl|Query\_31568 | KFX34817.1 glycosyltransferase family 39 protein [Geobacillus icigianus] | 680 |
      | lcl|Query\_31569 | KFX34818.1 glycosyltransferase [Geobacillus icigianus] | 320 |
      | lcl|Query\_31570 | KFX34819.1 GtrA family protein [Geobacillus icigianus] | 136 |
      | lcl|Query\_31571 | KFX34820.1 glutamate-5-semialdehyde dehydrogenase [Geobacillus icigianus] | 414 |
      | lcl|Query\_31572 | KFX34821.1 glutamate 5-kinase [Geobacillus icigianus] | 374 |
      | lcl|Query\_31573 | PUA93305.1 hypothetical protein EP10\_19595 [Geobacillus icigianus] | 72 |
      | lcl|Query\_31574 | KFX34822.1 hypothetical protein EP10\_05975 [Geobacillus icigianus] | 71 |
      | lcl|Query\_31575 | KFX34823.1 dihydroxy-acid dehydratase [Geobacillus icigianus] | 559 |
      | lcl|Query\_31576 | KFX34801.1 EamA family transporter [Geobacillus icigianus] | 137 |
      | lcl|Query\_31577 | KFX34805.2 type III-B CRISPR module RAMP protein Cmr1 [Geobacillus icigianus] | 297 |
      | lcl|Query\_31578 | KFX34794.1 SMI1/KNR4 family protein [Geobacillus icigianus] | 147 |
      | lcl|Query\_31579 | KFX34793.1 cytoplasmic protein [Geobacillus icigianus] | 152 |
      | lcl|Query\_31580 | PUA93304.1 hypothetical protein EP10\_19600, partial [Geobacillus icigianus] | 146 |
      | lcl|Query\_31581 | KFX34791.1 hypothetical protein EP10\_06005 [Geobacillus icigianus] | 136 |
      | lcl|Query\_31582 | KFX34792.1 hypothetical protein EP10\_06010 [Geobacillus icigianus] | 110 |
      | lcl|Query\_31583 | KFX34689.1 cytosolic protein [Geobacillus icigianus] | 133 |
      | lcl|Query\_31584 | KFX34690.1 acyl-CoA thioesterase [Geobacillus icigianus] | 133 |
      | lcl|Query\_31585 | KFX34691.1 alcohol dehydrogenase [Geobacillus icigianus] | 395 |
      | lcl|Query\_31586 | KFX34692.1 2-nitropropane dioxygenase [Geobacillus icigianus] | 344 |
      | lcl|Query\_31587 | KFX34693.1 LacI family transcriptional regulator [Geobacillus icigianus] | 338 |
      | lcl|Query\_31588 | KFX34694.1 enoyl-CoA hydratase [Geobacillus icigianus] | 254 |
      | lcl|Query\_31589 | KFX34695.1 glycosyltransferase [Geobacillus icigianus] | 486 |
      | lcl|Query\_31590 | KFX34696.1 aldehyde dehydrogenase [Geobacillus icigianus] | 478 |
      | lcl|Query\_31591 | KFX34697.1 sodium:solute symporter [Geobacillus icigianus] | 469 |
      | lcl|Query\_31592 | KFX34698.1 aspartate aminotransferase family protein [Geobacillus icigianus] | 445 |
      | lcl|Query\_31593 | KFX34699.1 4-aminobutyrate--2-oxoglutarate transaminase [Geobacillus icigianus] | 448 |
      | lcl|Query\_31594 | KFX34700.1 PAS domain-containing protein [Geobacillus icigianus] | 464 |
      | lcl|Query\_31595 | KFX34701.1 agmatinase [Geobacillus icigianus] | 324 |
      | lcl|Query\_31596 | KFX34702.1 aldehyde dehydrogenase [Geobacillus icigianus] | 486 |
      | lcl|Query\_31597 | KFX34703.1 cytosine permease [Geobacillus icigianus] | 458 |
      | lcl|Query\_31598 | KFX34704.1 carbon-nitrogen hydrolase family protein [Geobacillus icigianus] | 273 |
      | lcl|Query\_31599 | KFX34705.1 cyclase family protein [Geobacillus icigianus] | 216 |
      | lcl|Query\_31600 | KFX34706.1 D-serine ammonia-lyase [Geobacillus icigianus] | 453 |
      | lcl|Query\_31601 | KFX34707.1 GNAT family N-acetyltransferase [Geobacillus icigianus] | 159 |
      | lcl|Query\_31602 | KFX34708.1 urease accessory protein UreH [Geobacillus icigianus] | 207 |
      | lcl|Query\_31603 | KFX34709.1 urease accessory protein UreD [Geobacillus icigianus] | 271 |
      | lcl|Query\_31604 | KFX34711.1 urease accessory protein UreF [Geobacillus icigianus] | 225 |
      | lcl|Query\_31605 | KFX34712.1 urease accessory protein UreE [Geobacillus icigianus] | 148 |
      | lcl|Query\_31606 | KFX34713.1 urease subunit alpha [Geobacillus icigianus] | 569 |
      | lcl|Query\_31607 | KFX34714.1 urease subunit beta [Geobacillus icigianus] | 108 |
      | lcl|Query\_31608 | KFX34715.1 urease subunit gamma [Geobacillus icigianus] | 101 |
      | lcl|Query\_31609 | KFX34716.1 urea ABC transporter ATP-binding subunit UrtE [Geobacillus icigianus] | 233 |
      | lcl|Query\_31610 | KFX34717.1 urea ABC transporter ATP-binding protein UrtD [Geobacillus icigianus] | 254 |
      | lcl|Query\_31611 | KFX34718.1 urea ABC transporter permease subunit UrtC [Geobacillus icigianus] | 352 |
      | lcl|Query\_31612 | KFX34720.1 urea ABC transporter substrate-binding protein [Geobacillus icigianus] | 418 |
      | lcl|Query\_31613 | KFX34721.1 chemotaxis protein [Geobacillus icigianus] | 465 |
      | lcl|Query\_31614 | KFX34722.1 permease DsdX [Geobacillus icigianus] | 449 |
      | lcl|Query\_31615 | KFX34723.1 gluconate kinase [Geobacillus icigianus] | 514 |
      | lcl|Query\_31616 | KFX34724.1 LacI family transcriptional regulator [Geobacillus icigianus] | 338 |
      | lcl|Query\_31617 | KFX34725.1 hypothetical protein EP10\_06220 [Geobacillus icigianus] | 74 |
      | lcl|Query\_31618 | KFX34726.1 mannitol-1-phosphate 5-dehydrogenase [Geobacillus icigianus] | 387 |
      | lcl|Query\_31619 | KFX34727.1 PTS mannitol transporter subunit IIA [Geobacillus icigianus] | 147 |
      | lcl|Query\_31620 | KFX34728.1 PRD domain-containing protein [Geobacillus icigianus] | 697 |
      | lcl|Query\_31621 | KFX34729.1 PTS mannitol transporter subunit IICBA [Geobacillus icigianus] | 482 |
      | lcl|Query\_31622 | KFX34730.1 PTS system, cellobiose-specific IIC component [Geobacillus icigianus] | 434 |
      | lcl|Query\_31623 | KFX34732.1 methylmalonate-semialdehyde dehydrogenase (CoA acylating) [Geobacillus icigianus] | 487 |
      | lcl|Query\_31624 | KFX34733.1 alcohol dehydrogenase [Geobacillus icigianus] | 395 |
      | lcl|Query\_31625 | KFX34734.1 sigma-54-dependent Fis family transcriptional regulator [Geobacillus icigianus] | 571 |
      | lcl|Query\_31626 | KFX34735.1 amino acid ABC transporter ATP-binding protein [Geobacillus icigianus] | 242 |
      | lcl|Query\_31627 | KFX34736.1 amino acid ABC transporter substrate-binding protein [Geobacillus icigianus] | 281 |
      | lcl|Query\_31628 | KFX34737.1 amino acid ABC transporter permease [Geobacillus icigianus] | 217 |
      | lcl|Query\_31629 | KFX34738.1 amino acid ABC transporter permease [Geobacillus icigianus] | 216 |
      | lcl|Query\_31630 | KFX34710.1 urease accessory protein UreG [Geobacillus icigianus] | 204 |
      | lcl|Query\_31631 | KFX34719.1 urea ABC transporter permease subunit UrtB [Geobacillus icigianus] | 299 |
      | lcl|Query\_31632 | KFX34731.2 IS630 family transposase [Geobacillus icigianus] | 166 |
      | lcl|Query\_31633 | KFX34739.2 sensor histidine kinase [Geobacillus icigianus] | 418 |
      | lcl|Query\_31634 | KFX34740.1 DNA-binding protein [Geobacillus icigianus] | 301 |
      | lcl|Query\_31635 | KFX34742.1 N-acetylmuramic acid 6-phosphate etherase, partial [Geobacillus icigianus] | 210 |
      | lcl|Query\_31636 | PUA93303.1 hypothetical protein EP10\_19620 [Geobacillus icigianus] | 75 |
      | lcl|Query\_31637 | KFX34684.1 DUF4277 domain-containing protein, partial [Geobacillus icigianus] | 150 |
      | lcl|Query\_31638 | KFX34486.1 ion transporter, partial [Geobacillus icigianus] | 208 |
      | lcl|Query\_31639 | KFX34487.1 hypothetical protein EP10\_06345 [Geobacillus icigianus] | 134 |
      | lcl|Query\_31640 | KFX34488.1 hypothetical protein EP10\_06350 [Geobacillus icigianus] | 87 |
      | lcl|Query\_31641 | KFX34489.1 hypothetical protein EP10\_06355 [Geobacillus icigianus] | 912 |
      | lcl|Query\_31642 | KFX34485.2 AAA family ATPase, partial [Geobacillus icigianus] | 64 |
      | lcl|Query\_31643 | KFX34481.2 glutathione S-transferase, partial [Geobacillus icigianus] | 172 |
      | lcl|Query\_31644 | KFX34482.1 radical SAM/SPASM domain-containing protein, partial [Geobacillus icigianus] | 422 |
      | lcl|Query\_31645 | KFX34468.1 IS701 family transposase, partial [Geobacillus icigianus] | 211 |
      | lcl|Query\_31646 | KFX34469.1 2-oxo acid dehydrogenase subunit E2 [Geobacillus icigianus] | 449 |
      | lcl|Query\_31647 | KFX34470.1 alpha-ketoacid dehydrogenase subunit beta [Geobacillus icigianus] | 327 |
      | lcl|Query\_31648 | KFX34471.1 thiamine pyrophosphate-dependent dehydrogenase E1 component subunit alpha [Geobacillus icigianus] | 331 |
      | lcl|Query\_31649 | KFX34472.1 dihydrolipoyl dehydrogenase [Geobacillus icigianus] | 473 |
      | lcl|Query\_31650 | KFX34473.1 butyrate kinase [Geobacillus icigianus] | 370 |
      | lcl|Query\_31651 | KFX34474.1 Glu/Leu/Phe/Val dehydrogenase [Geobacillus icigianus] | 367 |
      | lcl|Query\_31652 | KFX34475.1 phosphate butyryltransferase [Geobacillus icigianus] | 299 |
      | lcl|Query\_31653 | KFX34476.1 sigma-54-dependent Fis family transcriptional regulator [Geobacillus icigianus] | 687 |
      | lcl|Query\_31654 | KFX34477.1 DUF2627 domain-containing protein [Geobacillus icigianus] | 82 |
      | lcl|Query\_31655 | KFX34478.1 glycerophosphodiester phosphodiesterase [Geobacillus icigianus] | 242 |
      | lcl|Query\_31656 | KFX34479.2 hypothetical protein EP10\_06440 [Geobacillus icigianus] | 222 |
      | lcl|Query\_31657 | KFX34447.1 IS701 family transposase, partial [Geobacillus icigianus] | 286 |
      | lcl|Query\_31658 | PUA93302.1 TetR/AcrR family transcriptional regulator [Geobacillus icigianus] | 193 |
      | lcl|Query\_31659 | PUA93299.1 multidrug ABC transporter ATP-binding protein, partial [Geobacillus icigianus] | 44 |
      | lcl|Query\_31660 | KFX34420.1 hypothetical protein EP10\_06460 [Geobacillus icigianus] | 240 |
      | lcl|Query\_31661 | KFX34421.1 nitric-oxide reductase large subunit [Geobacillus icigianus] | 787 |
      | lcl|Query\_31662 | KFX34422.1 Crp/Fnr family transcriptional regulator [Geobacillus icigianus] | 230 |
      | lcl|Query\_31663 | KFX34423.1 DUF2249 domain-containing protein [Geobacillus icigianus] | 77 |
      | lcl|Query\_31664 | KFX34424.1 DUF2249 domain-containing protein [Geobacillus icigianus] | 69 |
      | lcl|Query\_31665 | KFX34425.2 hypothetical protein EP10\_06485 [Geobacillus icigianus] | 416 |
      | lcl|Query\_31666 | KFX34426.1 metal-sulfur cluster assembly factor [Geobacillus icigianus] | 100 |
      | lcl|Query\_31667 | KFX34427.1 hypothetical protein EP10\_06495 [Geobacillus icigianus] | 69 |
      | lcl|Query\_31668 | KFX34428.1 cadmium-translocating P-type ATPase [Geobacillus icigianus] | 713 |
      | lcl|Query\_31669 | KFX34429.1 transcriptional regulator [Geobacillus icigianus] | 123 |
      | lcl|Query\_31670 | KFX34430.1 esterase [Geobacillus icigianus] | 255 |
      | lcl|Query\_31671 | KFX34431.1 Cof-type HAD-IIB family hydrolase [Geobacillus icigianus] | 268 |
      | lcl|Query\_31672 | KFX34432.1 DUF3813 domain-containing protein [Geobacillus icigianus] | 66 |
      | lcl|Query\_31673 | KFX34433.1 DegV family protein [Geobacillus icigianus] | 287 |
      | lcl|Query\_31674 | PUA93300.1 DUF3941 domain-containing protein [Geobacillus icigianus] | 43 |
      | lcl|Query\_31675 | PUA93301.1 methyl-accepting chemotaxis protein [Geobacillus icigianus] | 560 |
      | lcl|Query\_31676 | KFX34434.1 BMP family ABC transporter substrate-binding protein [Geobacillus icigianus] | 325 |
      | lcl|Query\_31677 | KFX34435.1 YajQ family cyclic di-GMP-binding protein [Geobacillus icigianus] | 163 |
      | lcl|Query\_31678 | KFX34436.2 L-lactate permease [Geobacillus icigianus] | 492 |
      | lcl|Query\_31679 | KFX34437.1 ATP-binding protein [Geobacillus icigianus] | 250 |
      | lcl|Query\_31680 | KFX34438.1 IS21 family transposase [Geobacillus icigianus] | 501 |
      | lcl|Query\_31681 | KFX34439.1 spermidine/putrescine ABC transporter substrate-binding protein [Geobacillus icigianus] | 357 |
      | lcl|Query\_31682 | KFX34440.1 ABC transporter permease [Geobacillus icigianus] | 267 |
      | lcl|Query\_31683 | KFX34441.1 ABC transporter permease [Geobacillus icigianus] | 268 |
      | lcl|Query\_31684 | KFX34442.1 ABC transporter ATP-binding protein [Geobacillus icigianus] | 363 |
      | lcl|Query\_31685 | KFX34443.1 ABC transporter ATP-binding protein [Geobacillus icigianus] | 218 |
      | lcl|Query\_31686 | KFX34444.1 IS701 family transposase, partial [Geobacillus icigianus] | 115 |
      | lcl|Query\_31687 | KFX34389.1 hypothetical protein EP10\_06605 [Geobacillus icigianus] | 84 |
      | lcl|Query\_31688 | KFX34390.1 hypothetical protein EP10\_06610 [Geobacillus icigianus] | 136 |
      | lcl|Query\_31689 | KFX34391.1 ABC transporter ATP-binding protein [Geobacillus icigianus] | 229 |
      | lcl|Query\_31690 | KFX34392.1 ABC transporter permease [Geobacillus icigianus] | 432 |
      | lcl|Query\_31691 | KFX34393.1 ABC transporter permease [Geobacillus icigianus] | 372 |
      | lcl|Query\_31692 | KFX34394.1 class I SAM-dependent methyltransferase [Geobacillus icigianus] | 247 |
      | lcl|Query\_31693 | KFX34396.1 HD domain-containing protein [Geobacillus icigianus] | 189 |
      | lcl|Query\_31694 | KFX34397.1 nicotinate-nucleotide adenylyltransferase [Geobacillus icigianus] | 216 |
      | lcl|Query\_31695 | KFX34398.1 ribosome assembly RNA-binding protein YhbY [Geobacillus icigianus] | 97 |
      | lcl|Query\_31696 | KFX34399.1 shikimate dehydrogenase [Geobacillus icigianus] | 276 |
      | lcl|Query\_31697 | KFX34401.1 YqeG family HAD IIIA-type phosphatase [Geobacillus icigianus] | 171 |
      | lcl|Query\_31698 | KFX34402.1 sporulation histidine kinase inhibitor Sda [Geobacillus icigianus] | 46 |
      | lcl|Query\_31699 | KFX34403.1 phosphatidylserine decarboxylase [Geobacillus icigianus] | 269 |
      | lcl|Query\_31700 | KFX34404.1 CDP-diacylglycerol--serine O-phosphatidyltransferase [Geobacillus icigianus] | 179 |
      | lcl|Query\_31701 | KFX34405.1 flagellar motor protein MotA [Geobacillus icigianus] | 266 |
      | lcl|Query\_31702 | KFX34406.1 flagellar motor protein MotB [Geobacillus icigianus] | 258 |
      | lcl|Query\_31703 | KFX34407.1 hypothetical protein EP10\_06695 [Geobacillus icigianus] | 79 |
      | lcl|Query\_31704 | KFX34408.1 sporulation integral membrane protein YtvI [Geobacillus icigianus] | 341 |
      | lcl|Query\_31705 | KFX34409.1 DedA family protein [Geobacillus icigianus] | 197 |
      | lcl|Query\_31706 | KFX34410.1 glutamate/aspartate:proton symporter GltP [Geobacillus icigianus] | 422 |
      | lcl|Query\_31707 | KFX34395.1 ribosome silencing factor [Geobacillus icigianus] | 118 |
      | lcl|Query\_31708 | KFX34400.1 ribosome biogenesis GTPase YqeH [Geobacillus icigianus] | 369 |
      | lcl|Query\_31709 | KFX34386.1 hypothetical protein EP10\_06720 [Geobacillus icigianus] | 117 |
      | lcl|Query\_31710 | KFX34387.1 transcriptional repressor [Geobacillus icigianus] | 150 |
      | lcl|Query\_31711 | KFX34349.1 small, acid-soluble spore protein, H family [Geobacillus icigianus] | 65 |
      | lcl|Query\_31712 | KFX34350.1 AcrB/AcrD/AcrF family protein [Geobacillus icigianus] | 1073 |
      | lcl|Query\_31713 | KFX34352.2 N-acetyltransferase [Geobacillus icigianus] | 166 |
      | lcl|Query\_31714 | KFX34353.1 hypothetical protein EP10\_06760 [Geobacillus icigianus] | 84 |
      | lcl|Query\_31715 | PUA93298.1 ion transporter, partial [Geobacillus icigianus] | 111 |
      | lcl|Query\_31716 | KFX34354.2 nuclease [Geobacillus icigianus] | 292 |
      | lcl|Query\_31717 | KFX34302.1 vitamin B12-dependent ribonucleotide reductase [Geobacillus icigianus] | 857 |
      | lcl|Query\_31718 | KFX34303.1 LysM peptidoglycan-binding domain-containing protein [Geobacillus icigianus] | 470 |
      | lcl|Query\_31719 | KFX34304.2 DUF2332 domain-containing protein [Geobacillus icigianus] | 74 |
      | lcl|Query\_31720 | KFX34305.1 spore photoproduct lyase [Geobacillus icigianus] | 341 |
      | lcl|Query\_31721 | KFX34306.1 transcriptional regulator MntR [Geobacillus icigianus] | 141 |
      | lcl|Query\_31722 | KFX34307.1 hypothetical protein EP10\_06815 [Geobacillus icigianus] | 294 |
      | lcl|Query\_31723 | KFX34308.1 hypothetical protein EP10\_06820 [Geobacillus icigianus] | 118 |
      | lcl|Query\_31724 | KFX34309.1 DUF1385 domain-containing protein [Geobacillus icigianus] | 310 |
      | lcl|Query\_31725 | KFX34310.1 hypothetical protein EP10\_06830 [Geobacillus icigianus] | 176 |
      | lcl|Query\_31726 | KFX34311.1 aminopeptidase P family protein [Geobacillus icigianus] | 353 |
      | lcl|Query\_31727 | KFX34312.1 elongation factor P [Geobacillus icigianus] | 185 |
      | lcl|Query\_31728 | KFX34313.1 DUF2619 domain-containing protein [Geobacillus icigianus] | 93 |
      | lcl|Query\_31729 | KFX34314.1 stage III sporulation protein AA [Geobacillus icigianus] | 306 |
      | lcl|Query\_31730 | KFX34315.1 stage III sporulation protein SpoAB [Geobacillus icigianus] | 170 |
      | lcl|Query\_31731 | KFX34316.1 stage III sporulation protein AC [Geobacillus icigianus] | 68 |
      | lcl|Query\_31732 | KFX34317.1 stage III sporulation protein AD [Geobacillus icigianus] | 128 |
      | lcl|Query\_31733 | KFX34318.1 stage III sporulation protein AE [Geobacillus icigianus] | 387 |
      | lcl|Query\_31734 | KFX34319.1 stage III sporulation protein AF [Geobacillus icigianus] | 204 |
      | lcl|Query\_31735 | KFX34320.1 stage III sporulation protein AG [Geobacillus icigianus] | 212 |
      | lcl|Query\_31736 | KFX34321.1 SpoIIIAH-like family protein [Geobacillus icigianus] | 182 |
      | lcl|Query\_31737 | KFX34322.1 acetyl-CoA carboxylase biotin carboxyl carrier protein [Geobacillus icigianus] | 177 |
      | lcl|Query\_31738 | KFX34323.1 acetyl-CoA carboxylase biotin carboxylase subunit [Geobacillus icigianus] | 451 |
      | lcl|Query\_31739 | KFX34324.1 Asp23/Gls24 family envelope stress response protein [Geobacillus icigianus] | 133 |
      | lcl|Query\_31740 | KFX34325.1 transcription antitermination factor NusB [Geobacillus icigianus] | 130 |
      | lcl|Query\_31741 | KFX34326.1 bifunctional methylenetetrahydrofolate dehydrogenase/methenyltetrahydrofolate cyclohydrolase FolD [Geobacillus icigianus] | 284 |
      | lcl|Query\_31742 | KFX34327.1 exodeoxyribonuclease VII large subunit [Geobacillus icigianus] | 449 |
      | lcl|Query\_31743 | KFX34328.1 exodeoxyribonuclease VII small subunit [Geobacillus icigianus] | 76 |
      | lcl|Query\_31744 | KFX34329.1 polyprenyl synthetase family protein [Geobacillus icigianus] | 297 |
      | lcl|Query\_31745 | KFX34330.1 1-deoxy-D-xylulose-5-phosphate synthase [Geobacillus icigianus] | 630 |
      | lcl|Query\_31746 | KFX34331.1 TlyA family rRNA (cytidine-2'-O)-methyltransferase [Geobacillus icigianus] | 282 |
      | lcl|Query\_31747 | KFX34332.1 transcriptional regulator ArgR [Geobacillus icigianus] | 149 |
      | lcl|Query\_31748 | KFX34333.1 DNA repair protein RecN [Geobacillus icigianus] | 573 |
      | lcl|Query\_31749 | KFX34334.2 SpoIVB peptidase [Geobacillus icigianus] | 431 |
      | lcl|Query\_31750 | PUA93297.1 hypothetical protein EP10\_19675 [Geobacillus icigianus] | 71 |
      | lcl|Query\_31751 | KFX34287.1 polyisoprenoid-binding protein [Geobacillus icigianus] | 175 |
      | lcl|Query\_31752 | KFX34288.1 3-hydroxybutyrate dehydrogenase [Geobacillus icigianus] | 258 |
      | lcl|Query\_31753 | KFX34289.1 4a-hydroxytetrahydrobiopterin dehydratase [Geobacillus icigianus] | 101 |
      | lcl|Query\_31754 | KFX34290.1 lipase [Geobacillus icigianus] | 416 |
      | lcl|Query\_31755 | KFX34291.1 bile acid:sodium symporter family protein [Geobacillus icigianus] | 325 |
      | lcl|Query\_31756 | KFX34292.1 MBL fold metallo-hydrolase [Geobacillus icigianus] | 327 |
      | lcl|Query\_31757 | KFX34293.1 hypothetical protein EP10\_07005 [Geobacillus icigianus] | 89 |
      | lcl|Query\_31758 | KFX34295.1 hypothetical protein EP10\_07015 [Geobacillus icigianus] | 72 |
      | lcl|Query\_31759 | KFX34296.1 ABC transporter substrate-binding protein [Geobacillus icigianus] | 438 |
      | lcl|Query\_31760 | KFX34297.1 carbohydrate ABC transporter permease [Geobacillus icigianus] | 270 |
      | lcl|Query\_31761 | KFX34298.1 sugar ABC transporter permease [Geobacillus icigianus] | 313 |
      | lcl|Query\_31762 | KFX34299.1 sn-glycerol-3-phosphate ABC transporter ATP-binding protein UgpC [Geobacillus icigianus] | 366 |
      | lcl|Query\_31763 | KFX34300.1 peptide MFS transporter [Geobacillus icigianus] | 496 |
      | lcl|Query\_31764 | KFX34286.1 MurR/RpiR family transcriptional regulator [Geobacillus icigianus] | 285 |
      | lcl|Query\_31765 | KFX34294.2 hypothetical protein EP10\_07010 [Geobacillus icigianus] | 133 |
      | lcl|Query\_31766 | KFX34180.1 hypothetical protein EP10\_07045 [Geobacillus icigianus] | 63 |
      | lcl|Query\_31767 | KFX34181.2 VanZ family protein [Geobacillus icigianus] | 202 |
      | lcl|Query\_31768 | KFX34182.1 hypothetical protein EP10\_07060 [Geobacillus icigianus] | 68 |
      | lcl|Query\_31769 | KFX34183.1 type 1 glutamine amidotransferase domain-containing protein [Geobacillus icigianus] | 221 |
      | lcl|Query\_31770 | KFX34184.1 MMPL family transporter [Geobacillus icigianus] | 1054 |
      | lcl|Query\_31771 | KFX34186.1 alkaline phosphatase [Geobacillus icigianus] | 429 |
      | lcl|Query\_31772 | KFX34187.1 fluoride efflux transporter CrcB [Geobacillus icigianus] | 116 |
      | lcl|Query\_31773 | KFX34188.1 CrcB family protein [Geobacillus icigianus] | 128 |
      | lcl|Query\_31774 | KFX34189.1 molybdate ABC transporter substrate-binding protein [Geobacillus icigianus] | 263 |
      | lcl|Query\_31775 | KFX34190.1 phenylalanine--tRNA ligase subunit beta [Geobacillus icigianus] | 804 |
      | lcl|Query\_31776 | KFX34191.1 phenylalanine--tRNA ligase subunit alpha [Geobacillus icigianus] | 344 |
      | lcl|Query\_31777 | KFX34192.1 hypothetical protein EP10\_07120 [Geobacillus icigianus] | 144 |
      | lcl|Query\_31778 | KFX34193.1 RNA methyltransferase [Geobacillus icigianus] | 251 |
      | lcl|Query\_31779 | KFX34195.1 nucleotidyltransferase [Geobacillus icigianus] | 247 |
      | lcl|Query\_31780 | KFX34196.1 small acid-soluble spore protein SspI [Geobacillus icigianus] | 69 |
      | lcl|Query\_31781 | KFX34197.2 hypothetical protein EP10\_07145 [Geobacillus icigianus] | 194 |
      | lcl|Query\_31782 | KFX34198.1 hypothetical protein EP10\_07150 [Geobacillus icigianus] | 94 |
      | lcl|Query\_31783 | KFX34200.1 M42 family peptidase [Geobacillus icigianus] | 361 |
      | lcl|Query\_31784 | KFX34201.1 dUTPase [Geobacillus icigianus] | 161 |
      | lcl|Query\_31785 | PUA93296.1 DUF1294 domain-containing protein [Geobacillus icigianus] | 68 |
      | lcl|Query\_31786 | KFX34202.1 50S ribosomal protein L20 [Geobacillus icigianus] | 119 |
      | lcl|Query\_31787 | KFX34203.1 50S ribosomal protein L35 [Geobacillus icigianus] | 66 |
      | lcl|Query\_31788 | KFX34204.2 translation initiation factor IF-3 [Geobacillus icigianus] | 173 |
      | lcl|Query\_31789 | KFX34205.1 threonine--tRNA ligase [Geobacillus icigianus] | 649 |
      | lcl|Query\_31790 | KFX34206.1 putative sporulation protein YtxC [Geobacillus icigianus] | 291 |
      | lcl|Query\_31791 | KFX34207.1 primosomal protein DnaI [Geobacillus icigianus] | 313 |
      | lcl|Query\_31792 | KFX34209.1 transcriptional regulator NrdR [Geobacillus icigianus] | 153 |
      | lcl|Query\_31793 | KFX34210.1 cytosolic protein [Geobacillus icigianus] | 132 |
      | lcl|Query\_31794 | KFX34211.1 S-adenosylmethionine decarboxylase proenzyme [Geobacillus icigianus] | 124 |
      | lcl|Query\_31795 | KFX34212.1 glyceraldehyde-3-phosphate dehydrogenase [Geobacillus icigianus] | 342 |
      | lcl|Query\_31796 | KFX34213.1 dephospho-CoA kinase [Geobacillus icigianus] | 201 |
      | lcl|Query\_31797 | KFX34214.1 DNA-formamidopyrimidine glycosylase [Geobacillus icigianus] | 274 |
      | lcl|Query\_31798 | KFX34215.1 DNA polymerase I [Geobacillus icigianus] | 878 |
      | lcl|Query\_31799 | KFX34216.1 PAS domain-containing sensor histidine kinase [Geobacillus icigianus] | 582 |
      | lcl|Query\_31800 | KFX34217.1 DNA-binding response regulator [Geobacillus icigianus] | 235 |
      | lcl|Query\_31801 | KFX34218.1 enoyl-CoA hydratase [Geobacillus icigianus] | 155 |
      | lcl|Query\_31802 | KFX34219.1 malate dehydrogenase [Geobacillus icigianus] | 312 |
      | lcl|Query\_31803 | KFX34220.1 NADP-dependent isocitrate dehydrogenase [Geobacillus icigianus] | 423 |
      | lcl|Query\_31804 | KFX34221.1 citrate synthase [Geobacillus icigianus] | 372 |
      | lcl|Query\_31805 | KFX34222.1 DUF441 domain-containing protein [Geobacillus icigianus] | 151 |
      | lcl|Query\_31806 | KFX34223.1 sporulation integral membrane protein YtvI [Geobacillus icigianus] | 372 |
      | lcl|Query\_31807 | KFX34224.1 pyruvate kinase [Geobacillus icigianus] | 587 |
      | lcl|Query\_31808 | KFX34225.1 6-phosphofructokinase [Geobacillus icigianus] | 319 |
      | lcl|Query\_31809 | KFX34226.1 acetyl-CoA carboxylase carboxyl transferase subunit alpha [Geobacillus icigianus] | 325 |
      | lcl|Query\_31810 | KFX34227.1 acetyl-CoA carboxylase carboxyltransferase subunit beta [Geobacillus icigianus] | 290 |
      | lcl|Query\_31811 | KFX34228.1 DNA polymerase III subunit alpha [Geobacillus icigianus] | 1093 |
      | lcl|Query\_31812 | KFX34229.1 sporulation protein [Geobacillus icigianus] | 113 |
      | lcl|Query\_31813 | KFX34230.1 sporulation protein [Geobacillus icigianus] | 167 |
      | lcl|Query\_31814 | KFX34231.1 bifunctional oligoribonuclease/PAP phosphatase NrnA [Geobacillus icigianus] | 315 |
      | lcl|Query\_31815 | KFX34232.1 hypothetical protein EP10\_07340 [Geobacillus icigianus] | 101 |
      | lcl|Query\_31816 | KFX34233.1 CBS domain-containing protein [Geobacillus icigianus] | 435 |
      | lcl|Query\_31817 | KFX34234.1 metal-dependent hydrolase [Geobacillus icigianus] | 226 |
      | lcl|Query\_31818 | KFX34235.1 aminopeptidase P family protein [Geobacillus icigianus] | 364 |
      | lcl|Query\_31819 | KFX34236.1 alanine dehydrogenase [Geobacillus icigianus] | 372 |
      | lcl|Query\_31820 | KFX34237.1 SDR family NAD(P)-dependent oxidoreductase [Geobacillus icigianus] | 254 |
      | lcl|Query\_31821 | KFX34238.1 universal stress protein [Geobacillus icigianus] | 148 |
      | lcl|Query\_31822 | KFX34239.1 argininosuccinate lyase [Geobacillus icigianus] | 458 |
      | lcl|Query\_31823 | KFX34240.1 argininosuccinate synthase [Geobacillus icigianus] | 406 |
      | lcl|Query\_31824 | KFX34185.1 MarR family transcriptional regulator [Geobacillus icigianus] | 150 |
      | lcl|Query\_31825 | KFX34194.1 peptide-methionine (S)-S-oxide reductase [Geobacillus icigianus] | 174 |
      | lcl|Query\_31826 | KFX34199.1 hypothetical protein EP10\_07155 [Geobacillus icigianus] | 115 |
      | lcl|Query\_31827 | KFX34208.1 Replication initiation and membrane attachment protein [Geobacillus icigianus] | 460 |
      | lcl|Query\_31828 | KFX34176.1 hypothetical protein EP10\_07395 [Geobacillus icigianus] | 253 |
      | lcl|Query\_31829 | KFX34171.1 hypothetical protein EP10\_07400 [Geobacillus icigianus] | 107 |
      | lcl|Query\_31830 | PUA93295.1 hypothetical protein EP10\_19690 [Geobacillus icigianus] | 206 |
      | lcl|Query\_31831 | KFX34172.1 MFS transporter, partial [Geobacillus icigianus] | 198 |
      | lcl|Query\_31832 | PUA93294.1 SMI1/KNR4 family protein, partial [Geobacillus icigianus] | 115 |
      | lcl|Query\_31833 | KFX34166.1 sensor histidine kinase, partial [Geobacillus icigianus] | 133 |
      | lcl|Query\_31834 | KFX34167.1 DNA-binding response regulator [Geobacillus icigianus] | 236 |
      | lcl|Query\_31835 | KFX34148.1 YitT family protein [Geobacillus icigianus] | 289 |
      | lcl|Query\_31836 | KFX34150.1 4-hydroxy-tetrahydrodipicolinate reductase [Geobacillus icigianus] | 264 |
      | lcl|Query\_31837 | KFX34151.1 methylglyoxal synthase [Geobacillus icigianus] | 144 |
      | lcl|Query\_31838 | KFX34152.1 bacillithiol biosynthesis deacetylase BshB1 [Geobacillus icigianus] | 237 |
      | lcl|Query\_31839 | KFX34153.1 N-acetyl-alpha-D-glucosaminyl L-malate synthase BshA [Geobacillus icigianus] | 378 |
      | lcl|Query\_31840 | KFX34154.1 CCA tRNA nucleotidyltransferase [Geobacillus icigianus] | 404 |
      | lcl|Query\_31841 | KFX34155.1 bifunctional biotin--[acetyl-CoA-carboxylase] synthetase/biotin operon repressor [Geobacillus icigianus] | 329 |
      | lcl|Query\_31842 | KFX34156.1 3-methyl-2-oxobutanoate hydroxymethyltransferase [Geobacillus icigianus] | 279 |
      | lcl|Query\_31843 | KFX34157.1 pantoate--beta-alanine ligase [Geobacillus icigianus] | 289 |
      | lcl|Query\_31844 | KFX34158.1 aspartate 1-decarboxylase [Geobacillus icigianus] | 127 |
      | lcl|Query\_31845 | PUA93293.1 DUF4264 domain-containing protein [Geobacillus icigianus] | 56 |
      | lcl|Query\_31846 | KFX34160.1 peptidase M4 [Geobacillus icigianus] | 158 |
      | lcl|Query\_31847 | KFX34161.1 pyridoxal phosphate-dependent aminotransferase [Geobacillus icigianus] | 393 |
      | lcl|Query\_31848 | KFX34162.1 asparagine--tRNA ligase [Geobacillus icigianus] | 431 |
      | lcl|Query\_31849 | KFX34163.1 DnaD domain protein [Geobacillus icigianus] | 235 |
      | lcl|Query\_31850 | KFX34164.1 endonuclease III [Geobacillus icigianus] | 217 |
      | lcl|Query\_31851 | KFX34149.1 nucleotide pyrophosphohydrolase [Geobacillus icigianus] | 111 |
      | lcl|Query\_31852 | KFX34159.1 ATP-dependent helicase DinG [Geobacillus icigianus] | 909 |
      | lcl|Query\_31853 | KFX34133.1 IS66-like element ISGst1 family transposase [Geobacillus icigianus] | 482 |
      | lcl|Query\_31854 | PUA93292.1 MMPL family transporter [Geobacillus icigianus] | 694 |
      | lcl|Query\_31855 | KFX34100.1 hypothetical protein EP10\_07540 [Geobacillus icigianus] | 120 |
      | lcl|Query\_31856 | KFX34101.1 IS701 family transposase, partial [Geobacillus icigianus] | 52 |
      | lcl|Query\_31857 | PUA93291.1 right-handed parallel beta-helix repeat-containing protein, partial [Geobacillus icigianus] | 494 |
      | lcl|Query\_31858 | KFX34040.2 hypothetical protein EP10\_07555 [Geobacillus icigianus] | 105 |
      | lcl|Query\_31859 | KFX34041.1 DUF115 domain-containing protein [Geobacillus icigianus] | 594 |
      | lcl|Query\_31860 | KFX34042.1 hypothetical protein EP10\_07565 [Geobacillus icigianus] | 119 |
      | lcl|Query\_31861 | KFX34043.1 UDP-N-acetylglucosamine 4,6-dehydratase (inverting) [Geobacillus icigianus] | 324 |
      | lcl|Query\_31862 | KFX34044.1 gfo/Idh/MocA family oxidoreductase, partial [Geobacillus icigianus] | 152 |
      | lcl|Query\_31863 | PUA93290.1 DUF115 domain-containing protein [Geobacillus icigianus] | 414 |
      | lcl|Query\_31864 | KFX33960.1 HTH-type transcriptional regulator Hpr [Geobacillus icigianus] | 205 |
      | lcl|Query\_31865 | KFX33961.1 DUF1878 domain-containing protein [Geobacillus icigianus] | 113 |
      | lcl|Query\_31866 | KFX33962.1 hypothetical protein EP10\_07605 [Geobacillus icigianus] | 61 |
      | lcl|Query\_31867 | PUA93283.1 YjcZ family sporulation protein [Geobacillus icigianus] | 31 |
      | lcl|Query\_31868 | KFX33964.1 HIT family protein [Geobacillus icigianus] | 140 |
      | lcl|Query\_31869 | KFX33965.1 ABC transporter ATP-binding protein [Geobacillus icigianus] | 245 |
      | lcl|Query\_31870 | KFX33966.1 ABC transporter [Geobacillus icigianus] | 399 |
      | lcl|Query\_31871 | KFX33969.1 ferrochelatase [Geobacillus icigianus] | 316 |
      | lcl|Query\_31872 | KFX33970.1 protoporphyrinogen oxidase [Geobacillus icigianus] | 480 |
      | lcl|Query\_31873 | KFX33971.1 hypothetical protein EP10\_07660 [Geobacillus icigianus] | 67 |
      | lcl|Query\_31874 | KFX33972.1 TetR/AcrR family transcriptional regulator [Geobacillus icigianus] | 190 |
      | lcl|Query\_31875 | KFX33973.1 YhgE/Pip domain-containing protein [Geobacillus icigianus] | 791 |
      | lcl|Query\_31876 | PUA93284.1 hypothetical protein EP10\_19725 [Geobacillus icigianus] | 83 |
      | lcl|Query\_31877 | PUA93285.1 YhfH family protein [Geobacillus icigianus] | 45 |
      | lcl|Query\_31878 | KFX33974.1 hypothetical protein EP10\_07680 [Geobacillus icigianus] | 244 |
      | lcl|Query\_31879 | KFX33975.1 fatty acid--CoA ligase family protein [Geobacillus icigianus] | 514 |
      | lcl|Query\_31880 | KFX33976.1 BMP family ABC transporter substrate-binding protein [Geobacillus icigianus] | 336 |
      | lcl|Query\_31881 | KFX33978.1 D-amino-acid transaminase [Geobacillus icigianus] | 287 |
      | lcl|Query\_31882 | KFX33979.1 branched-chain amino acid ABC transporter permease [Geobacillus icigianus] | 238 |
      | lcl|Query\_31883 | KFX33980.1 AzlD domain-containing protein [Geobacillus icigianus] | 101 |
      | lcl|Query\_31884 | KFX33981.1 SCO family protein [Geobacillus icigianus] | 197 |
      | lcl|Query\_31885 | KFX33982.1 isocitrate lyase [Geobacillus icigianus] | 428 |
      | lcl|Query\_31886 | KFX33983.1 hypothetical protein EP10\_07730 [Geobacillus icigianus] | 64 |
      | lcl|Query\_31887 | KFX33984.1 competence protein ComK [Geobacillus icigianus] | 177 |
      | lcl|Query\_31888 | KFX33985.1 TVP38/TMEM64 family protein [Geobacillus icigianus] | 207 |
      | lcl|Query\_31889 | KFX33986.1 signal peptidase I [Geobacillus icigianus] | 182 |
      | lcl|Query\_31890 | KFX33987.1 helicase-exonuclease AddAB subunit AddB [Geobacillus icigianus] | 1173 |
      | lcl|Query\_31891 | KFX33988.1 helicase-exonuclease AddAB subunit AddA [Geobacillus icigianus] | 1242 |
      | lcl|Query\_31892 | KFX33989.1 exonuclease SbcCD subunit D [Geobacillus icigianus] | 394 |
      | lcl|Query\_31893 | KFX33990.1 SMC family ATPase [Geobacillus icigianus] | 1113 |
      | lcl|Query\_31894 | KFX33991.1 spore germination protein [Geobacillus icigianus] | 73 |
      | lcl|Query\_31895 | KFX33992.1 spore germination protein GerPE [Geobacillus icigianus] | 125 |
      | lcl|Query\_31896 | KFX33993.1 spore gernimation protein GerPD [Geobacillus icigianus] | 63 |
      | lcl|Query\_31897 | KFX33994.1 spore gernimation protein GerPC [Geobacillus icigianus] | 200 |
      | lcl|Query\_31898 | KFX33995.1 spore gernimation protein [Geobacillus icigianus] | 74 |
      | lcl|Query\_31899 | KFX33996.1 spore germination protein [Geobacillus icigianus] | 73 |
      | lcl|Query\_31900 | KFX33997.1 spore germination protein [Geobacillus icigianus] | 74 |
      | lcl|Query\_31901 | KFX33998.1 spore germination protein [Geobacillus icigianus] | 74 |
      | lcl|Query\_31902 | PUA93286.1 aspartyl-phosphate phosphatase Spo0E family protein [Geobacillus icigianus] | 56 |
      | lcl|Query\_31903 | KFX34001.1 FAA hydrolase family protein [Geobacillus icigianus] | 300 |
      | lcl|Query\_31904 | KFX34003.1 GGDEF domain-containing protein [Geobacillus icigianus] | 946 |
      | lcl|Query\_31905 | KFX34004.1 DUF2777 domain-containing protein [Geobacillus icigianus] | 182 |
      | lcl|Query\_31906 | KFX34006.1 alpha-glycosidase [Geobacillus icigianus] | 587 |
      | lcl|Query\_31907 | KFX34007.1 ABC transporter substrate-binding protein [Geobacillus icigianus] | 423 |
      | lcl|Query\_31908 | KFX34008.1 sugar ABC transporter permease [Geobacillus icigianus] | 426 |
      | lcl|Query\_31909 | KFX34009.1 sugar ABC transporter permease [Geobacillus icigianus] | 280 |
      | lcl|Query\_31910 | KFX34010.1 alpha-amlyase [Geobacillus icigianus] | 511 |
      | lcl|Query\_31911 | KFX34011.1 LacI family transcriptional regulator [Geobacillus icigianus] | 339 |
      | lcl|Query\_31912 | KFX34012.1 hypothetical protein EP10\_07890 [Geobacillus icigianus] | 75 |
      | lcl|Query\_31913 | KFX34013.1 hypothetical protein EP10\_07895 [Geobacillus icigianus] | 139 |
      | lcl|Query\_31914 | KFX34014.1 hypothetical protein EP10\_07900 [Geobacillus icigianus] | 92 |
      | lcl|Query\_31915 | KFX34015.1 hypothetical protein EP10\_07905 [Geobacillus icigianus] | 123 |
      | lcl|Query\_31916 | KFX34016.1 antibiotic biosynthesis monooxygenase [Geobacillus icigianus] | 112 |
      | lcl|Query\_31917 | KFX34017.1 NAD(P)-dependent oxidoreductase [Geobacillus icigianus] | 287 |
      | lcl|Query\_31918 | PUA93287.1 type II toxin-antitoxin system death-on-curing family toxin [Geobacillus icigianus] | 131 |
      | lcl|Query\_31919 | KFX34019.1 methionine synthase [Geobacillus icigianus] | 1136 |
      | lcl|Query\_31920 | KFX34020.1 bifunctional homocysteine S-methyltransferase/methylenetetrahydrofolate reductase [Geobacillus icigianus] | 616 |
      | lcl|Query\_31921 | PUA93288.1 hypothetical protein EP10\_19760 [Geobacillus icigianus] | 67 |
      | lcl|Query\_31922 | KFX34022.1 circular bacteriocin, circularin A/uberolysin family [Geobacillus icigianus] | 64 |
      | lcl|Query\_31923 | KFX34023.1 hypothetical protein EP10\_07955 [Geobacillus icigianus] | 539 |
      | lcl|Query\_31924 | KFX34024.1 stage II sporulation protein M [Geobacillus icigianus] | 170 |
      | lcl|Query\_31925 | KFX33963.1 peptidylprolyl isomerase [Geobacillus icigianus] | 280 |
      | lcl|Query\_31926 | KFX33968.1 uroporphyrinogen decarboxylase [Geobacillus icigianus] | 345 |
      | lcl|Query\_31927 | KFX34025.1 ABC transporter ATP-binding protein [Geobacillus icigianus] | 265 |
      | lcl|Query\_31928 | KFX33977.1 ABC transporter permease [Geobacillus icigianus] | 330 |
      | lcl|Query\_31929 | KFX34000.1 DUF418 domain-containing protein [Geobacillus icigianus] | 389 |
      | lcl|Query\_31930 | KFX34002.1 hypothetical protein EP10\_07835 [Geobacillus icigianus] | 117 |
      | lcl|Query\_31931 | KFX34005.1 asparagine synthase (glutamine-hydrolyzing) [Geobacillus icigianus] | 615 |
      | lcl|Query\_31932 | PUA93289.1 DUF1878 domain-containing protein [Geobacillus icigianus] | 114 |
      | lcl|Query\_31933 | KFX33935.1 hypothetical protein EP10\_07975 [Geobacillus icigianus] | 64 |
      | lcl|Query\_31934 | KFX33936.1 oligoribonuclease [Geobacillus icigianus] | 414 |
      | lcl|Query\_31935 | KFX33937.1 hypothetical protein EP10\_07985 [Geobacillus icigianus] | 636 |
      | lcl|Query\_31936 | KFX33938.1 MoxR family ATPase [Geobacillus icigianus] | 291 |
      | lcl|Query\_31937 | KFX33939.1 Replication termination protein [Geobacillus icigianus] | 124 |
      | lcl|Query\_31938 | KFX33940.1 formyltetrahydrofolate deformylase [Geobacillus icigianus] | 300 |
      | lcl|Query\_31939 | KFX33941.1 NO-inducible flavohemoprotein [Geobacillus icigianus] | 411 |
      | lcl|Query\_31940 | KFX33942.1 HTH-type transcriptional repressor NsrR [Geobacillus icigianus] | 148 |
      | lcl|Query\_31941 | KFX33943.1 polyamine aminopropyltransferase [Geobacillus icigianus] | 291 |
      | lcl|Query\_31942 | KFX33944.1 acyl-CoA dehydrogenase [Geobacillus icigianus] | 391 |
      | lcl|Query\_31943 | KFX33945.1 aminotransferase class V-fold PLP-dependent enzyme [Geobacillus icigianus] | 499 |
      | lcl|Query\_31944 | KFX33946.1 acyl-CoA thioesterase [Geobacillus icigianus] | 147 |
      | lcl|Query\_31945 | KFX33947.1 sodium:proton antiporter [Geobacillus icigianus] | 470 |
      | lcl|Query\_31946 | KFX33948.1 UTRA domain-containing protein, partial [Geobacillus icigianus] | 52 |
      | lcl|Query\_31947 | PUA93282.1 glutamyl endopeptidase, partial [Geobacillus icigianus] | 219 |
      | lcl|Query\_31948 | KFX33929.1 glycine--tRNA ligase [Geobacillus icigianus] | 460 |
      | lcl|Query\_31949 | KFX33931.1 MFS transporter [Geobacillus icigianus] | 398 |
      | lcl|Query\_31950 | KFX33932.1 hypothetical protein EP10\_08060 [Geobacillus icigianus] | 105 |
      | lcl|Query\_31951 | KFX33933.1 IS701 family transposase, partial [Geobacillus icigianus] | 286 |
      | lcl|Query\_31952 | KFX33930.1 glycosyltransferase family 2 protein [Geobacillus icigianus] | 276 |
      | lcl|Query\_31953 | KFX33828.2 DUF4277 domain-containing protein, partial [Geobacillus icigianus] | 207 |
      | lcl|Query\_31954 | KFX33829.1 HD-GYP domain-containing protein [Geobacillus icigianus] | 498 |
      | lcl|Query\_31955 | KFX33830.1 hypothetical protein EP10\_08085 [Geobacillus icigianus] | 123 |
      | lcl|Query\_31956 | KFX33831.1 GlsB/YeaQ/YmgE family stress response membrane protein [Geobacillus icigianus] | 84 |
      | lcl|Query\_31957 | KFX33832.1 hypothetical protein EP10\_08095 [Geobacillus icigianus] | 148 |
      | lcl|Query\_31958 | KFX33834.1 LysR family transcriptional regulator [Geobacillus icigianus] | 304 |
      | lcl|Query\_31959 | KFX33835.1 transcriptional regulator [Geobacillus icigianus] | 99 |
      | lcl|Query\_31960 | KFX33836.1 copper-translocating P-type ATPase [Geobacillus icigianus] | 798 |
      | lcl|Query\_31961 | KFX33837.1 copper chaperone [Geobacillus icigianus] | 67 |
      | lcl|Query\_31962 | KFX33839.1 DUF86 domain-containing protein [Geobacillus icigianus] | 112 |
      | lcl|Query\_31963 | KFX33840.2 peptidase C39 [Geobacillus icigianus] | 283 |
      | lcl|Query\_31964 | KFX33841.1 asparagine synthase (glutamine-hydrolyzing) [Geobacillus icigianus] | 635 |
      | lcl|Query\_31965 | KFX33842.1 ribonucleoside-diphosphate reductase subunit alpha [Geobacillus icigianus] | 760 |
      | lcl|Query\_31966 | KFX33843.1 ribonucleotide-diphosphate reductase subunit beta [Geobacillus icigianus] | 346 |
      | lcl|Query\_31967 | KFX33844.1 UDP-N-acetylmuramate dehydrogenase [Geobacillus icigianus] | 304 |
      | lcl|Query\_31968 | KFX33847.1 hypothetical protein EP10\_08180, partial [Geobacillus icigianus] | 62 |
      | lcl|Query\_31969 | KFX33833.1 putative sulfate exporter family transporter [Geobacillus icigianus] | 345 |
      | lcl|Query\_31970 | KFX33838.2 nucleotidyltransferase [Geobacillus icigianus] | 101 |
      | lcl|Query\_31971 | KFX33846.1 threonine/serine exporter [Geobacillus icigianus] | 247 |
      | lcl|Query\_31972 | KFX33824.1 flagellar protein FlgN, partial [Geobacillus icigianus] | 640 |
      | lcl|Query\_31973 | KFX33700.1 trehalose operon repressor, partial [Geobacillus icigianus] | 188 |
      | lcl|Query\_31974 | KFX33701.1 alpha,alpha-phosphotrehalase [Geobacillus icigianus] | 563 |
      | lcl|Query\_31975 | KFX33702.1 PTS trehalose transporter subunit IIBC [Geobacillus icigianus] | 471 |
      | lcl|Query\_31976 | KFX33703.1 MerR family transcriptional regulator [Geobacillus icigianus] | 241 |
      | lcl|Query\_31977 | KFX33704.1 hypothetical protein EP10\_08210 [Geobacillus icigianus] | 87 |
      | lcl|Query\_31978 | KFX33705.1 PilZ domain-containing protein [Geobacillus icigianus] | 114 |
      | lcl|Query\_31979 | KFX33706.1 DNA topoisomerase IV subunit A [Geobacillus icigianus] | 811 |
      | lcl|Query\_31980 | KFX33707.1 DNA topoisomerase IV subunit B [Geobacillus icigianus] | 645 |
      | lcl|Query\_31981 | KFX33708.1 CoA-binding protein [Geobacillus icigianus] | 139 |
      | lcl|Query\_31982 | KFX33709.1 AarF/ABC1/UbiB kinase family protein [Geobacillus icigianus] | 554 |
      | lcl|Query\_31983 | KFX33710.1 polyhydroxyalkanoate synthesis regulator [Geobacillus icigianus] | 99 |
      | lcl|Query\_31984 | KFX33698.1 hypothetical protein EP10\_08250, partial [Geobacillus icigianus] | 59 |
      | lcl|Query\_31985 | KFX33699.1 N-acetyltransferase [Geobacillus icigianus] | 144 |
      | lcl|Query\_31986 | PUA93281.1 hypothetical protein EP10\_19780 [Geobacillus icigianus] | 60 |
      | lcl|Query\_31987 | KFX33689.1 cell filamentation protein Fic [Geobacillus icigianus] | 212 |
      | lcl|Query\_31988 | KFX33690.1 integrase [Geobacillus icigianus] | 311 |
      | lcl|Query\_31989 | KFX33691.1 hypothetical protein EP10\_08280 [Geobacillus icigianus] | 60 |
      | lcl|Query\_31990 | KFX33692.1 anhydro-N-acetylmuramic acid kinase [Geobacillus icigianus] | 393 |
      | lcl|Query\_31991 | KFX33693.1 DUF871 domain-containing protein, partial [Geobacillus icigianus] | 244 |
      | lcl|Query\_31992 | PUA93275.1 hypothetical protein EP10\_19785, partial [Geobacillus icigianus] | 95 |
      | lcl|Query\_31993 | KFX33657.1 MFS transporter [Geobacillus icigianus] | 569 |
      | lcl|Query\_31994 | KFX33658.1 HlyD family secretion protein [Geobacillus icigianus] | 212 |
      | lcl|Query\_31995 | KFX33659.1 MarR family transcriptional regulator [Geobacillus icigianus] | 146 |
      | lcl|Query\_31996 | KFX33661.1 acyl-CoA carboxylase subunit beta [Geobacillus icigianus] | 517 |
      | lcl|Query\_31997 | KFX33662.1 enoyl-CoA hydratase [Geobacillus icigianus] | 260 |
      | lcl|Query\_31998 | KFX33663.1 hydroxymethylglutaryl-CoA lyase [Geobacillus icigianus] | 299 |
      | lcl|Query\_31999 | KFX33664.1 acetyl-CoA carboxylase biotin carboxyl carrier protein subunit [Geobacillus icigianus] | 70 |
      | lcl|Query\_32000 | KFX33665.1 acetyl-CoA carboxylase biotin carboxylase subunit [Geobacillus icigianus] | 450 |
      | lcl|Query\_32001 | KFX33666.1 AMP-binding protein [Geobacillus icigianus] | 544 |
      | lcl|Query\_32002 | KFX33667.1 acyl-CoA dehydrogenase [Geobacillus icigianus] | 380 |
      | lcl|Query\_32003 | KFX33668.1 TetR/AcrR family transcriptional regulator [Geobacillus icigianus] | 208 |
      | lcl|Query\_32004 | KFX33669.1 DNA alkylation repair protein [Geobacillus icigianus] | 82 |
      | lcl|Query\_32005 | KFX33670.1 N-acetyltransferase [Geobacillus icigianus] | 263 |
      | lcl|Query\_32006 | KFX33671.1 cytosolic protein [Geobacillus icigianus] | 85 |
      | lcl|Query\_32007 | KFX33673.1 PAS domain-containing sensor histidine kinase [Geobacillus icigianus] | 420 |
      | lcl|Query\_32008 | KFX33674.1 MATE family efflux transporter [Geobacillus icigianus] | 455 |
      | lcl|Query\_32009 | PUA93276.1 hypothetical protein EP10\_19800 [Geobacillus icigianus] | 53 |
      | lcl|Query\_32010 | PUA93277.1 YjcZ family sporulation protein [Geobacillus icigianus] | 50 |
      | lcl|Query\_32011 | PUA93278.1 YjcZ family sporulation protein [Geobacillus icigianus] | 32 |
      | lcl|Query\_32012 | PUA93279.1 sporulation protein YjcZ [Geobacillus icigianus] | 29 |
      | lcl|Query\_32013 | PUA93280.1 hypothetical protein EP10\_19820 [Geobacillus icigianus] | 71 |
      | lcl|Query\_32014 | KFX33675.1 cation transporter [Geobacillus icigianus] | 296 |
      | lcl|Query\_32015 | KFX33676.1 class I SAM-dependent methyltransferase [Geobacillus icigianus] | 240 |
      | lcl|Query\_32016 | KFX33677.1 D-alanyl-D-alanine carboxypeptidase family protein [Geobacillus icigianus] | 296 |
      | lcl|Query\_32017 | KFX33678.1 purine-nucleoside phosphorylase [Geobacillus icigianus] | 236 |
      | lcl|Query\_32018 | KFX33679.1 hypothetical protein EP10\_08485 [Geobacillus icigianus] | 135 |
      | lcl|Query\_32019 | KFX33680.1 hypothetical protein EP10\_08500 [Geobacillus icigianus] | 105 |
      | lcl|Query\_32020 | KFX33681.1 DUF2515 domain-containing protein [Geobacillus icigianus] | 368 |
      | lcl|Query\_32021 | KFX33682.1 YozD family protein [Geobacillus icigianus] | 59 |
      | lcl|Query\_32022 | KFX33684.1 YozE family protein [Geobacillus icigianus] | 77 |
      | lcl|Query\_32023 | KFX33685.1 biotin synthase BioB [Geobacillus icigianus] | 337 |
      | lcl|Query\_32024 | KFX33672.1 threonine ammonia-lyase [Geobacillus icigianus] | 402 |
      | lcl|Query\_32025 | KFX33683.1 hypothetical protein EP10\_08520 [Geobacillus icigianus] | 215 |
      | lcl|Query\_32026 | KFX33642.1 PTS sugar transporter subunit IIB, partial [Geobacillus icigianus] | 46 |
      | lcl|Query\_32027 | KFX33643.1 PTS sugar transporter subunit IIC [Geobacillus icigianus] | 434 |
      | lcl|Query\_32028 | KFX33630.1 AraC family transcriptional regulator [Geobacillus icigianus] | 545 |
      | lcl|Query\_32029 | KFX33631.1 iron ABC transporter permease [Geobacillus icigianus] | 335 |
      | lcl|Query\_32030 | KFX33632.1 iron ABC transporter permease [Geobacillus icigianus] | 341 |
      | lcl|Query\_32031 | KFX33633.1 alpha/beta hydrolase [Geobacillus icigianus] | 287 |
      | lcl|Query\_32032 | KFX33636.1 DinB family protein [Geobacillus icigianus] | 161 |
      | lcl|Query\_32033 | KFX33637.1 TatD family deoxyribonuclease [Geobacillus icigianus] | 254 |
      | lcl|Query\_32034 | KFX33634.1 DUF2975 domain-containing protein [Geobacillus icigianus] | 159 |
      | lcl|Query\_32035 | KFX33635.1 transcriptional regulator [Geobacillus icigianus] | 74 |
      | lcl|Query\_32036 | KFX33638.1 sigma-54-dependent Fis family transcriptional regulator [Geobacillus icigianus] | 455 |
      | lcl|Query\_32037 | KFX33626.1 IS630 family transposase, partial [Geobacillus icigianus] | 71 |
      | lcl|Query\_32038 | PUA93274.1 IS630 family transposase [Geobacillus icigianus] | 154 |
      | lcl|Query\_32039 | KFX33571.1 hypothetical protein EP10\_08645, partial [Geobacillus icigianus] | 162 |
      | lcl|Query\_32040 | KFX33572.1 ABC transporter ATP-binding protein [Geobacillus icigianus] | 305 |
      | lcl|Query\_32041 | KFX33567.1 hypothetical protein EP10\_08655, partial [Geobacillus icigianus] | 314 |
      | lcl|Query\_32042 | PUA93273.1 hypothetical protein EP10\_19860 [Geobacillus icigianus] | 291 |
      | lcl|Query\_32043 | KFX33569.1 hypothetical protein EP10\_08665 [Geobacillus icigianus] | 189 |
      | lcl|Query\_32044 | PUA93272.1 MFS transporter, partial [Geobacillus icigianus] | 72 |
      | lcl|Query\_32045 | KFX33527.1 HIT domain-containing protein [Geobacillus icigianus] | 144 |
      | lcl|Query\_32046 | KFX33529.1 DUF1640 domain-containing protein [Geobacillus icigianus] | 111 |
      | lcl|Query\_32047 | KFX33530.1 YdiU family protein [Geobacillus icigianus] | 487 |
      | lcl|Query\_32048 | KFX33532.1 ATPase [Geobacillus icigianus] | 568 |
      | lcl|Query\_32049 | KFX33533.1 DUF2294 domain-containing protein [Geobacillus icigianus] | 122 |
      | lcl|Query\_32050 | KFX33534.1 NADH dehydrogenase subunit 5 [Geobacillus icigianus] | 500 |
      | lcl|Query\_32051 | KFX33535.1 DUF2309 domain-containing protein [Geobacillus icigianus] | 863 |
      | lcl|Query\_32052 | KFX33537.1 butyryl-CoA dehydrogenase [Geobacillus icigianus] | 399 |
      | lcl|Query\_32053 | KFX33531.1 MerR family transcriptional regulator [Geobacillus icigianus] | 262 |
      | lcl|Query\_32054 | KFX33536.1 DUF3870 domain-containing protein [Geobacillus icigianus] | 102 |
      | lcl|Query\_32055 | KFX33485.1 NCS2 family permease [Geobacillus icigianus] | 441 |
      | lcl|Query\_32056 | KFX33486.1 glutamine-hydrolyzing GMP synthase [Geobacillus icigianus] | 510 |
      | lcl|Query\_32057 | KFX33487.1 DUF4129 domain-containing protein [Geobacillus icigianus] | 721 |
      | lcl|Query\_32058 | KFX33490.1 class II fumarate hydratase [Geobacillus icigianus] | 456 |
      | lcl|Query\_32059 | KFX33491.1 hypothetical protein EP10\_08770 [Geobacillus icigianus] | 140 |
      | lcl|Query\_32060 | KFX33492.1 hypothetical protein EP10\_08780 [Geobacillus icigianus] | 153 |
      | lcl|Query\_32061 | PUA93269.1 peptidoglycan-binding protein [Geobacillus icigianus] | 386 |
      | lcl|Query\_32062 | KFX33493.1 DNA-binding protein [Geobacillus icigianus] | 82 |
      | lcl|Query\_32063 | KFX33494.1 hypothetical protein EP10\_08800 [Geobacillus icigianus] | 61 |
      | lcl|Query\_32064 | KFX33495.1 hypothetical protein EP10\_08810 [Geobacillus icigianus] | 179 |
      | lcl|Query\_32065 | KFX33496.1 hypothetical protein EP10\_08815 [Geobacillus icigianus] | 256 |
      | lcl|Query\_32066 | KFX33497.1 N-acetylmuramoyl-L-alanine amidase [Geobacillus icigianus] | 238 |
      | lcl|Query\_32067 | KFX33498.1 hypothetical protein EP10\_08825 [Geobacillus icigianus] | 272 |
      | lcl|Query\_32068 | KFX33499.1 hypothetical protein EP10\_08830 [Geobacillus icigianus] | 126 |
      | lcl|Query\_32069 | KFX33500.1 hypothetical protein EP10\_08835 [Geobacillus icigianus] | 64 |
      | lcl|Query\_32070 | KFX33501.1 hypothetical protein EP10\_08840 [Geobacillus icigianus] | 116 |
      | lcl|Query\_32071 | KFX33502.1 glycoside hydrolase [Geobacillus icigianus] | 963 |
      | lcl|Query\_32072 | KFX33503.1 hypothetical protein EP10\_08850 [Geobacillus icigianus] | 265 |
      | lcl|Query\_32073 | KFX33504.1 hypothetical protein EP10\_08855 [Geobacillus icigianus] | 65 |
      | lcl|Query\_32074 | KFX33505.1 hypothetical protein EP10\_08860 [Geobacillus icigianus] | 495 |
      | lcl|Query\_32075 | KFX33506.1 hypothetical protein EP10\_08865 [Geobacillus icigianus] | 354 |
      | lcl|Query\_32076 | KFX33507.1 phage tail protein [Geobacillus icigianus] | 249 |
      | lcl|Query\_32077 | KFX33509.1 hypothetical protein EP10\_08880 [Geobacillus icigianus] | 267 |
      | lcl|Query\_32078 | PUA93270.1 ribbon-helix-helix domain-containing protein [Geobacillus icigianus] | 47 |
      | lcl|Query\_32079 | PUA93271.1 hypothetical protein EP10\_19900 [Geobacillus icigianus] | 70 |
      | lcl|Query\_32080 | KFX33510.1 hypothetical protein EP10\_08895 [Geobacillus icigianus] | 142 |
      | lcl|Query\_32081 | KFX33511.1 hypothetical protein EP10\_08900 [Geobacillus icigianus] | 316 |
      | lcl|Query\_32082 | KFX33512.1 hypothetical protein EP10\_08905 [Geobacillus icigianus] | 70 |
      | lcl|Query\_32083 | KFX33513.1 hypothetical protein EP10\_08910 [Geobacillus icigianus] | 147 |
      | lcl|Query\_32084 | KFX33514.1 hypothetical protein EP10\_08915 [Geobacillus icigianus] | 141 |
      | lcl|Query\_32085 | KFX33515.1 hypothetical protein EP10\_08920 [Geobacillus icigianus] | 141 |
      | lcl|Query\_32086 | KFX33516.1 hypothetical protein EP10\_08925 [Geobacillus icigianus] | 119 |
      | lcl|Query\_32087 | KFX33517.1 phage major capsid protein [Geobacillus icigianus] | 305 |
      | lcl|Query\_32088 | KFX33518.1 structural protein [Geobacillus icigianus] | 476 |
      | lcl|Query\_32089 | KFX33519.1 phage head morphogenesis protein [Geobacillus icigianus] | 288 |
      | lcl|Query\_32090 | KFX33520.1 phage portal protein [Geobacillus icigianus] | 481 |
      | lcl|Query\_32091 | KFX33521.1 hypothetical protein EP10\_08950 [Geobacillus icigianus] | 463 |
      | lcl|Query\_32092 | KFX33522.1 hypothetical protein EP10\_08955 [Geobacillus icigianus] | 177 |
      | lcl|Query\_32093 | KFX33488.1 DUF58 domain-containing protein [Geobacillus icigianus] | 389 |
      | lcl|Query\_32094 | KFX33489.2 MoxR family ATPase [Geobacillus icigianus] | 316 |
      | lcl|Query\_32095 | KFX33508.1 hypothetical protein EP10\_08875 [Geobacillus icigianus] | 476 |
      | lcl|Query\_32096 | KFX33479.1 flotillin family protein [Geobacillus icigianus] | 505 |
      | lcl|Query\_32097 | KFX33480.1 hypothetical protein EP10\_08980 [Geobacillus icigianus] | 109 |
      | lcl|Query\_32098 | KFX33481.1 diacylglycerol kinase [Geobacillus icigianus] | 306 |
      | lcl|Query\_32099 | KFX33482.1 23S rRNA (uracil(1939)-C(5))-methyltransferase RlmD [Geobacillus icigianus] | 459 |
      | lcl|Query\_32100 | PUA93268.1 hypothetical protein EP10\_19910 [Geobacillus icigianus] | 205 |
      | lcl|Query\_32101 | KFX33478.1 hypothetical protein EP10\_08970 [Geobacillus icigianus] | 178 |
      | lcl|Query\_32102 | PUA93267.1 hypothetical protein EP10\_19920, partial [Geobacillus icigianus] | 32 |
      | lcl|Query\_32103 | KFX33455.1 phosphotransferase [Geobacillus icigianus] | 264 |
      | lcl|Query\_32104 | KFX33456.1 NERD domain-containing protein [Geobacillus icigianus] | 305 |
      | lcl|Query\_32105 | KFX33459.1 dipeptidase PepV [Geobacillus icigianus] | 471 |
      | lcl|Query\_32106 | KFX33460.1 HTH domain-containing protein [Geobacillus icigianus] | 73 |
      | lcl|Query\_32107 | KFX33461.1 rRNA pseudouridine synthase [Geobacillus icigianus] | 248 |
      | lcl|Query\_32108 | KFX33462.1 hypothetical protein EP10\_09055 [Geobacillus icigianus] | 69 |
      | lcl|Query\_32109 | KFX33463.1 polysaccharide biosynthesis protein [Geobacillus icigianus] | 544 |
      | lcl|Query\_32110 | KFX33464.1 NAD(P)/FAD-dependent oxidoreductase [Geobacillus icigianus] | 435 |
      | lcl|Query\_32111 | KFX33465.1 DUF2758 domain-containing protein [Geobacillus icigianus] | 59 |
      | lcl|Query\_32112 | KFX33466.1 peptidase M4 family protein [Geobacillus icigianus] | 544 |
      | lcl|Query\_32113 | KFX33467.1 hypothetical protein EP10\_09080 [Geobacillus icigianus] | 166 |
      | lcl|Query\_32114 | KFX33468.1 helix-turn-helix domain-containing protein [Geobacillus icigianus] | 424 |
      | lcl|Query\_32115 | KFX33469.1 rhodanese-like domain-containing protein [Geobacillus icigianus] | 104 |
      | lcl|Query\_32116 | KFX33470.1 leucine--tRNA ligase [Geobacillus icigianus] | 805 |
      | lcl|Query\_32117 | KFX33457.1 RNA 2',3'-cyclic phosphodiesterase [Geobacillus icigianus] | 192 |
      | lcl|Query\_32118 | KFX33458.2 MFS transporter [Geobacillus icigianus] | 402 |
      | lcl|Query\_32119 | KFX33435.1 copper amine oxidase N-terminal domain-containing protein [Geobacillus icigianus] | 487 |
      | lcl|Query\_32120 | KFX33436.1 hypothetical protein EP10\_09130 [Geobacillus icigianus] | 1532 |
      | lcl|Query\_32121 | KFX33425.1 tryptophan transporter [Geobacillus icigianus] | 174 |
      | lcl|Query\_32122 | KFX33427.1 SigE-dependent sporulation protein [Geobacillus icigianus] | 61 |
      | lcl|Query\_32123 | KFX33428.1 phosphohydrolase [Geobacillus icigianus] | 41 |
      | lcl|Query\_32124 | KFX33429.1 3'-5' exoribonuclease YhaM [Geobacillus icigianus] | 325 |
      | lcl|Query\_32125 | KFX33430.1 ABC transporter permease [Geobacillus icigianus] | 409 |
      | lcl|Query\_32126 | KFX33431.1 DUF4162 domain-containing protein [Geobacillus icigianus] | 299 |
      | lcl|Query\_32127 | KFX33424.1 hypothetical protein EP10\_09135 [Geobacillus icigianus] | 62 |
      | lcl|Query\_32128 | KFX33426.1 3-phosphoserine/phosphohydroxythreonine transaminase [Geobacillus icigianus] | 360 |
      | lcl|Query\_32129 | KFX33410.1 IS701 family transposase, partial [Geobacillus icigianus] | 115 |
      | lcl|Query\_32130 | KFX33411.1 hypothetical protein EP10\_09180 [Geobacillus icigianus] | 585 |
      | lcl|Query\_32131 | KFX33412.1 phosphate ABC transporter substrate-binding protein [Geobacillus icigianus] | 286 |
      | lcl|Query\_32132 | KFX33413.1 phosphate ABC transporter permease subunit PstC [Geobacillus icigianus] | 311 |
      | lcl|Query\_32133 | KFX33414.1 phosphate ABC transporter permease PtsA [Geobacillus icigianus] | 296 |
      | lcl|Query\_32134 | KFX33416.1 PucR family transcriptional regulator [Geobacillus icigianus] | 411 |
      | lcl|Query\_32135 | KFX33417.1 alanine dehydrogenase [Geobacillus icigianus] | 377 |
      | lcl|Query\_32136 | KFX33418.1 hypothetical protein EP10\_09215 [Geobacillus icigianus] | 79 |
      | lcl|Query\_32137 | KFX33419.1 pyridoxal kinase [Geobacillus icigianus] | 271 |
      | lcl|Query\_32138 | KFX33420.1 sodium/proline symporter PutP [Geobacillus icigianus] | 485 |
      | lcl|Query\_32139 | KFX33374.1 Rrf2 family transcriptional regulator [Geobacillus icigianus] | 138 |
      | lcl|Query\_32140 | KFX33375.1 SDR family NAD(P)-dependent oxidoreductase [Geobacillus icigianus] | 291 |
      | lcl|Query\_32141 | KFX33377.1 L-cystine transporter [Geobacillus icigianus] | 461 |
      | lcl|Query\_32142 | KFX33378.1 MFS transporter [Geobacillus icigianus] | 264 |
      | lcl|Query\_32143 | KFX33376.2 alanine:cation symporter family protein [Geobacillus icigianus] | 489 |
      | lcl|Query\_32144 | KFX33237.1 sporulation protein [Geobacillus icigianus] | 244 |
      | lcl|Query\_32145 | KFX33238.1 NAD(+) synthase [Geobacillus icigianus] | 246 |
      | lcl|Query\_32146 | KFX33239.1 aminoglycoside phosphotransferase [Geobacillus icigianus] | 313 |
      | lcl|Query\_32147 | KFX33240.1 LysM peptidoglycan-binding domain-containing protein [Geobacillus icigianus] | 501 |
      | lcl|Query\_32148 | KFX33241.1 quinolinate synthase NadA [Geobacillus icigianus] | 367 |
      | lcl|Query\_32149 | KFX33242.1 carboxylating nicotinate-nucleotide diphosphorylase [Geobacillus icigianus] | 276 |
      | lcl|Query\_32150 | KFX33243.1 L-aspartate oxidase [Geobacillus icigianus] | 517 |
      | lcl|Query\_32151 | KFX33244.1 IscS subfamily cysteine desulfurase [Geobacillus icigianus] | 381 |
      | lcl|Query\_32152 | KFX33245.1 transcription repressor NadR [Geobacillus icigianus] | 179 |
      | lcl|Query\_32153 | KFX33247.1 ACT domain-containing protein [Geobacillus icigianus] | 147 |
      | lcl|Query\_32154 | KFX33248.1 GTPase ObgE [Geobacillus icigianus] | 433 |
      | lcl|Query\_32155 | KFX33249.1 sporulation protein [Geobacillus icigianus] | 181 |
      | lcl|Query\_32156 | KFX33250.1 50S ribosomal protein L27 [Geobacillus icigianus] | 96 |
      | lcl|Query\_32157 | KFX33251.1 ribosomal-processing cysteine protease Prp [Geobacillus icigianus] | 110 |
      | lcl|Query\_32158 | KFX33252.1 50S ribosomal protein L21 [Geobacillus icigianus] | 102 |
      | lcl|Query\_32159 | KFX33253.1 stage IV sporulation protein FB [Geobacillus icigianus] | 287 |
      | lcl|Query\_32160 | KFX33254.1 M23 family peptidase [Geobacillus icigianus] | 255 |
      | lcl|Query\_32161 | KFX33255.1 septum site-determining protein MinD [Geobacillus icigianus] | 267 |
      | lcl|Query\_32162 | KFX33256.2 septum site-determining protein MinC [Geobacillus icigianus] | 278 |
      | lcl|Query\_32163 | KFX33257.1 rod shape-determining protein MreD [Geobacillus icigianus] | 172 |
      | lcl|Query\_32164 | KFX33258.1 rod shape-determining protein MreC [Geobacillus icigianus] | 288 |
      | lcl|Query\_32165 | KFX33259.1 rod shape-determining protein [Geobacillus icigianus] | 340 |
      | lcl|Query\_32166 | KFX33260.1 JAB domain-containing protein [Geobacillus icigianus] | 226 |
      | lcl|Query\_32167 | KFX33261.1 septum formation inhibitor Maf [Geobacillus icigianus] | 193 |
      | lcl|Query\_32168 | KFX33262.1 stage II sporulation protein D [Geobacillus icigianus] | 371 |
      | lcl|Query\_32169 | KFX33263.1 type II secretion system protein [Geobacillus icigianus] | 153 |
      | lcl|Query\_32170 | KFX33264.1 pilus assembly protein PilO [Geobacillus icigianus] | 235 |
      | lcl|Query\_32171 | KFX33265.1 DUF2681 domain-containing protein [Geobacillus icigianus] | 191 |
      | lcl|Query\_32172 | KFX33266.1 pilus assembly protein PilM [Geobacillus icigianus] | 310 |
      | lcl|Query\_32173 | KFX33268.1 prepilin-type cleavage/methylation domain-containing protein [Geobacillus icigianus] | 158 |
      | lcl|Query\_32174 | KFX33269.1 type II secretion system F family protein [Geobacillus icigianus] | 403 |
      | lcl|Query\_32175 | KFX33270.1 type IV pilus twitching motility protein PilT [Geobacillus icigianus] | 351 |
      | lcl|Query\_32176 | KFX33271.1 type II secretion system protein GspE [Geobacillus icigianus] | 554 |
      | lcl|Query\_32177 | KFX33273.1 photosystem reaction center subunit H [Geobacillus icigianus] | 256 |
      | lcl|Query\_32178 | KFX33274.1 hypothetical protein EP10\_09450 [Geobacillus icigianus] | 483 |
      | lcl|Query\_32179 | KFX33275.1 general secretion pathway protein J [Geobacillus icigianus] | 210 |
      | lcl|Query\_32180 | KFX33276.1 Tfp pilus assembly protein [Geobacillus icigianus] | 189 |
      | lcl|Query\_32181 | KFX33277.1 hypothetical protein EP10\_09465 [Geobacillus icigianus] | 583 |
      | lcl|Query\_32182 | KFX33278.1 VWA domain-containing protein [Geobacillus icigianus] | 960 |
      | lcl|Query\_32183 | KFX33279.1 bifunctional folylpolyglutamate synthase/dihydrofolate synthase [Geobacillus icigianus] | 449 |
      | lcl|Query\_32184 | KFX33280.1 valine--tRNA ligase [Geobacillus icigianus] | 880 |
      | lcl|Query\_32185 | KFX33281.1 hypothetical protein EP10\_09490 [Geobacillus icigianus] | 62 |
      | lcl|Query\_32186 | KFX33282.1 spore coat protein YsxE [Geobacillus icigianus] | 343 |
      | lcl|Query\_32187 | KFX33283.1 stage VI sporulation protein D [Geobacillus icigianus] | 348 |
      | lcl|Query\_32188 | KFX33284.1 glutamate-1-semialdehyde 2,1-aminomutase [Geobacillus icigianus] | 429 |
      | lcl|Query\_32189 | KFX33285.1 porphobilinogen synthase [Geobacillus icigianus] | 324 |
      | lcl|Query\_32190 | KFX33286.1 uroporphyrinogen-III synthase [Geobacillus icigianus] | 256 |
      | lcl|Query\_32191 | KFX33287.1 hydroxymethylbilane synthase [Geobacillus icigianus] | 310 |
      | lcl|Query\_32192 | KFX33288.1 cytochrome C assembly protein [Geobacillus icigianus] | 272 |
      | lcl|Query\_32193 | KFX33289.1 glutamyl-tRNA reductase [Geobacillus icigianus] | 454 |
      | lcl|Query\_32194 | KFX33290.1 hypothetical protein EP10\_09535 [Geobacillus icigianus] | 161 |
      | lcl|Query\_32195 | KFX33291.1 YihA family ribosome biogenesis GTP-binding protein [Geobacillus icigianus] | 195 |
      | lcl|Query\_32196 | KFX33292.1 endopeptidase La [Geobacillus icigianus] | 780 |
      | lcl|Query\_32197 | KFX33293.1 ATP-dependent protease LonB [Geobacillus icigianus] | 557 |
      | lcl|Query\_32198 | KFX33294.1 ATP-dependent Clp protease ATP-binding subunit ClpX [Geobacillus icigianus] | 421 |
      | lcl|Query\_32199 | KFX33295.1 trigger factor [Geobacillus icigianus] | 428 |
      | lcl|Query\_32200 | KFX33296.1 hydrolase [Geobacillus icigianus] | 326 |
      | lcl|Query\_32201 | KFX33297.1 3-isopropylmalate dehydratase small subunit [Geobacillus icigianus] | 197 |
      | lcl|Query\_32202 | KFX33298.1 3-isopropylmalate dehydratase large subunit [Geobacillus icigianus] | 471 |
      | lcl|Query\_32203 | KFX33299.1 3-isopropylmalate dehydrogenase [Geobacillus icigianus] | 371 |
      | lcl|Query\_32204 | KFX33300.1 2-isopropylmalate synthase [Geobacillus icigianus] | 515 |
      | lcl|Query\_32205 | KFX33301.1 ketol-acid reductoisomerase [Geobacillus icigianus] | 341 |
      | lcl|Query\_32206 | KFX33302.1 acetolactate synthase small subunit [Geobacillus icigianus] | 172 |
      | lcl|Query\_32207 | KFX33303.1 acetolactate synthase large subunit [Geobacillus icigianus] | 579 |
      | lcl|Query\_32208 | KFX33304.1 hypothetical protein EP10\_09605 [Geobacillus icigianus] | 90 |
      | lcl|Query\_32209 | KFX33305.1 branched-chain-amino-acid transaminase [Geobacillus icigianus] | 299 |
      | lcl|Query\_32210 | KFX33306.1 metallophosphoesterase [Geobacillus icigianus] | 171 |
      | lcl|Query\_32211 | KFX33307.1 XTP/dITP diphosphatase [Geobacillus icigianus] | 204 |
      | lcl|Query\_32212 | KFX33309.1 sporulation protein [Geobacillus icigianus] | 357 |
      | lcl|Query\_32213 | KFX33310.1 glutamate racemase [Geobacillus icigianus] | 264 |
      | lcl|Query\_32214 | KFX33311.1 MarR family transcriptional regulator [Geobacillus icigianus] | 147 |
      | lcl|Query\_32215 | KFX33312.1 hypothetical protein EP10\_09645 [Geobacillus icigianus] | 75 |
      | lcl|Query\_32216 | KFX33313.1 DNA-binding response regulator [Geobacillus icigianus] | 74 |
      | lcl|Query\_32217 | KFX33314.1 succinate dehydrogenase iron-sulfur subunit [Geobacillus icigianus] | 253 |
      | lcl|Query\_32218 | KFX33315.1 succinate dehydrogenase flavoprotein subunit [Geobacillus icigianus] | 585 |
      | lcl|Query\_32219 | KFX33316.1 succinate dehydrogenase [Geobacillus icigianus] | 202 |
      | lcl|Query\_32220 | KFX33317.1 DUF2507 domain-containing protein [Geobacillus icigianus] | 160 |
      | lcl|Query\_32221 | KFX33320.1 iron permease [Geobacillus icigianus] | 308 |
      | lcl|Query\_32222 | KFX33321.1 hypothetical protein EP10\_09695 [Geobacillus icigianus] | 253 |
      | lcl|Query\_32223 | KFX33322.1 glycosyltransferase family 2 protein [Geobacillus icigianus] | 253 |
      | lcl|Query\_32224 | KFX33323.1 alkaline phosphatase family protein [Geobacillus icigianus] | 499 |
      | lcl|Query\_32225 | KFX33324.1 UPF0104 family protein [Geobacillus icigianus] | 310 |
      | lcl|Query\_32226 | KFX33325.1 UDP-glucose 4-epimerase [Geobacillus icigianus] | 314 |
      | lcl|Query\_32227 | KFX33326.1 hypothetical protein EP10\_09725 [Geobacillus icigianus] | 399 |
      | lcl|Query\_32228 | KFX33327.1 alkanesulfonate monooxygenase, FMNH(2)-dependent [Geobacillus icigianus] | 372 |
      | lcl|Query\_32229 | KFX33328.1 FMN reductase (NADPH) [Geobacillus icigianus] | 180 |
      | lcl|Query\_32230 | KFX33329.1 aliphatic sulfonate ABC transporter ATP-binding protein [Geobacillus icigianus] | 260 |
      | lcl|Query\_32231 | KFX33330.1 aliphatic sulfonate ABC transporter permease SsuC [Geobacillus icigianus] | 262 |
      | lcl|Query\_32232 | KFX33331.1 sulfonate ABC transporter substrate-binding protein [Geobacillus icigianus] | 331 |
      | lcl|Query\_32233 | KFX33332.1 thioredoxin [Geobacillus icigianus] | 105 |
      | lcl|Query\_32234 | KFX33333.1 electron transfer flavoprotein subunit alpha/FixB family protein [Geobacillus icigianus] | 325 |
      | lcl|Query\_32235 | KFX33334.1 electron transfer flavoprotein subunit beta/FixA family protein [Geobacillus icigianus] | 258 |
      | lcl|Query\_32236 | KFX33335.1 enoyl-CoA hydratase [Geobacillus icigianus] | 257 |
      | lcl|Query\_32237 | KFX33336.1 TetR family transcriptional regulator [Geobacillus icigianus] | 195 |
      | lcl|Query\_32238 | KFX33337.1 long-chain fatty acid--CoA ligase [Geobacillus icigianus] | 566 |
      | lcl|Query\_32239 | KFX33339.1 endonuclease MutS2 [Geobacillus icigianus] | 792 |
      | lcl|Query\_32240 | KFX33340.1 DNA polymerase/3'-5' exonuclease PolX [Geobacillus icigianus] | 574 |
      | lcl|Query\_32241 | KFX33341.1 CvpA family protein [Geobacillus icigianus] | 179 |
      | lcl|Query\_32242 | KFX33342.1 cell division protein ZapA [Geobacillus icigianus] | 91 |
      | lcl|Query\_32243 | KFX33343.1 ribonuclease HIII [Geobacillus icigianus] | 311 |
      | lcl|Query\_32244 | KFX33246.1 prephenate dehydratase [Geobacillus icigianus] | 283 |
      | lcl|Query\_32245 | KFX33267.1 prepilin peptidase [Geobacillus icigianus] | 248 |
      | lcl|Query\_32246 | KFX33272.2 hypothetical protein EP10\_09440 [Geobacillus icigianus] | 392 |
      | lcl|Query\_32247 | KFX33308.1 ribonuclease PH [Geobacillus icigianus] | 256 |
      | lcl|Query\_32248 | KFX33318.1 excinuclease ABC subunit UvrC [Geobacillus icigianus] | 590 |
      | lcl|Query\_32249 | KFX33319.1 nucleoporin-interacting protein [Geobacillus icigianus] | 478 |
      | lcl|Query\_32250 | KFX33338.1 DUF350 domain-containing protein [Geobacillus icigianus] | 135 |
      | lcl|Query\_32251 | PUA93266.1 hypothetical protein EP10\_19925, partial [Geobacillus icigianus] | 141 |
      | lcl|Query\_32252 | KFX33217.1 hypothetical protein EP10\_09830 [Geobacillus icigianus] | 244 |
      | lcl|Query\_32253 | KFX33218.1 hypothetical protein EP10\_09835 [Geobacillus icigianus] | 195 |
      | lcl|Query\_32254 | KFX33219.1 hypothetical protein EP10\_09840 [Geobacillus icigianus] | 69 |
      | lcl|Query\_32255 | KFX33220.1 recombinase family protein [Geobacillus icigianus] | 603 |
      | lcl|Query\_32256 | KFX33221.1 MgtC/SapB family protein [Geobacillus icigianus] | 225 |
      | lcl|Query\_32257 | KFX33222.1 EamA/RhaT family transporter [Geobacillus icigianus] | 302 |
      | lcl|Query\_32258 | KFX33223.1 protein-glutamine gamma-glutamyltransferase [Geobacillus icigianus] | 272 |
      | lcl|Query\_32259 | KFX33202.1 MFS transporter, partial [Geobacillus icigianus] | 158 |
      | lcl|Query\_32260 | KFX33203.1 glycogen biosynthesis protein GlgD [Geobacillus icigianus] | 54 |
      | lcl|Query\_32261 | KFX33204.1 DUF2524 domain-containing protein [Geobacillus icigianus] | 87 |
      | lcl|Query\_32262 | KFX33205.1 methyltransferase domain-containing protein [Geobacillus icigianus] | 201 |
      | lcl|Query\_32263 | KFX33206.1 DUF2600 domain-containing protein [Geobacillus icigianus] | 360 |
      | lcl|Query\_32264 | KFX33207.1 gamma carbonic anhydrase family protein [Geobacillus icigianus] | 176 |
      | lcl|Query\_32265 | KFX33208.1 methionine adenosyltransferase [Geobacillus icigianus] | 404 |
      | lcl|Query\_32266 | KFX33209.1 phosphoenolpyruvate carboxykinase (ATP) [Geobacillus icigianus] | 528 |
      | lcl|Query\_32267 | KFX33210.1 hypothetical protein EP10\_09920 [Geobacillus icigianus] | 127 |
      | lcl|Query\_32268 | KFX33196.1 tRNA (adenosine(37)-N6)-threonylcarbamoyltransferase complex transferase subunit TsaD [Geobacillus icigianus] | 338 |
      | lcl|Query\_32269 | KFX33197.1 ribosomal-protein-alanine N-acetyltransferase [Geobacillus icigianus] | 151 |
      | lcl|Query\_32270 | KFX33198.1 tRNA (adenosine(37)-N6)-threonylcarbamoyltransferase complex dimerization subunit type 1 TsaB [Geobacillus icigianus] | 246 |
      | lcl|Query\_32271 | KFX33199.1 tRNA (adenosine(37)-N6)-threonylcarbamoyltransferase complex ATPase subunit type 1 TsaE [Geobacillus icigianus] | 152 |
      | lcl|Query\_32272 | KFX33193.2 IS701 family transposase, partial [Geobacillus icigianus] | 206 |
      | lcl|Query\_32273 | KFX33194.2 O-methyltransferase [Geobacillus icigianus] | 190 |
      | lcl|Query\_32274 | KFX33178.1 undecaprenyl-phosphate glucose phosphotransferase, partial [Geobacillus icigianus] | 364 |
      | lcl|Query\_32275 | PUA93265.1 hypothetical protein EP10\_19930, partial [Geobacillus icigianus] | 154 |
      | lcl|Query\_32276 | KFX33172.1 hypothetical protein EP10\_09975 [Geobacillus icigianus] | 222 |
      | lcl|Query\_32277 | KFX33143.1 FAD-binding protein [Geobacillus icigianus] | 187 |
      | lcl|Query\_32278 | KFX33144.1 flavin reductase [Geobacillus icigianus] | 155 |
      | lcl|Query\_32279 | KFX33145.1 ABC transporter ATP-binding protein [Geobacillus icigianus] | 241 |
      | lcl|Query\_32280 | KFX33146.1 ABC transporter ATP-binding protein [Geobacillus icigianus] | 256 |
      | lcl|Query\_32281 | KFX33147.1 branched-chain amino acid ABC transporter permease [Geobacillus icigianus] | 354 |
      | lcl|Query\_32282 | KFX33148.1 branched-chain amino acid ABC transporter permease [Geobacillus icigianus] | 293 |
      | lcl|Query\_32283 | KFX33149.1 ABC transporter substrate-binding protein [Geobacillus icigianus] | 390 |
      | lcl|Query\_32284 | KFX33150.1 GntR family transcriptional regulator [Geobacillus icigianus] | 215 |
      | lcl|Query\_32285 | KFX33151.1 5-carboxymethyl-2-hydroxymuconate semialdehyde dehydrogenase [Geobacillus icigianus] | 503 |
      | lcl|Query\_32286 | KFX33152.1 5-carboxymethyl-2-hydroxymuconate isomerase [Geobacillus icigianus] | 130 |
      | lcl|Query\_32287 | KFX33153.1 2-hydroxyhepta-2,4-diene-1,7-dioate isomerase [Geobacillus icigianus] | 245 |
      | lcl|Query\_32288 | KFX33155.1 2,4-dihydroxyhept-2-ene-1,7-dioic acid aldolase [Geobacillus icigianus] | 305 |
      | lcl|Query\_32289 | KFX33156.1 3,4-dihydroxyphenylacetate 2,3-dioxygenase [Geobacillus icigianus] | 327 |
      | lcl|Query\_32290 | KFX33157.1 4-hydroxyphenylacetate 3-monooxygenase, oxygenase component [Geobacillus icigianus] | 492 |
      | lcl|Query\_32291 | KFX33158.1 hypothetical protein EP10\_10060 [Geobacillus icigianus] | 59 |
      | lcl|Query\_32292 | KFX33159.1 LysR family transcriptional regulator [Geobacillus icigianus] | 307 |
      | lcl|Query\_32293 | KFX33160.1 manganese catalase [Geobacillus icigianus] | 299 |
      | lcl|Query\_32294 | KFX33161.1 DUF2642 domain-containing protein [Geobacillus icigianus] | 75 |
      | lcl|Query\_32295 | KFX33162.1 PepSY domain-containing protein [Geobacillus icigianus] | 454 |
      | lcl|Query\_32296 | KFX33154.2 4-hydroxyphenylacetate isomerase [Geobacillus icigianus] | 260 |
      | lcl|Query\_32297 | KFX33163.1 DNA-binding response regulator [Geobacillus icigianus] | 391 |
      | lcl|Query\_32298 | KFX33066.1 M23 family peptidase [Geobacillus icigianus] | 291 |
      | lcl|Query\_32299 | KFX33067.1 stage II sporulation protein D [Geobacillus icigianus] | 342 |
      | lcl|Query\_32300 | KFX33068.1 UDP-N-acetylglucosamine 1-carboxyvinyltransferase [Geobacillus icigianus] | 434 |
      | lcl|Query\_32301 | KFX33069.2 hypothetical protein EP10\_10105 [Geobacillus icigianus] | 272 |
      | lcl|Query\_32302 | KFX33070.1 DUF1146 domain-containing protein [Geobacillus icigianus] | 79 |
      | lcl|Query\_32303 | KFX33071.1 NADH-quinone oxidoreductase subunit NuoN [Geobacillus icigianus] | 498 |
      | lcl|Query\_32304 | KFX33072.1 NADH-quinone oxidoreductase subunit M [Geobacillus icigianus] | 514 |
      | lcl|Query\_32305 | KFX33073.1 NADH-quinone oxidoreductase subunit L [Geobacillus icigianus] | 619 |
      | lcl|Query\_32306 | KFX33074.1 NADH-quinone oxidoreductase subunit NuoK [Geobacillus icigianus] | 101 |
      | lcl|Query\_32307 | KFX33075.1 NADH-quinone oxidoreductase subunit J [Geobacillus icigianus] | 173 |
      | lcl|Query\_32308 | KFX33076.1 NADH-quinone oxidoreductase subunit NuoI [Geobacillus icigianus] | 139 |
      | lcl|Query\_32309 | KFX33077.1 NADH-quinone oxidoreductase subunit NuoH [Geobacillus icigianus] | 333 |
      | lcl|Query\_32310 | KFX33078.1 NADH-quinone oxidoreductase subunit D [Geobacillus icigianus] | 366 |
      | lcl|Query\_32311 | KFX33079.1 NADH-quinone oxidoreductase subunit C [Geobacillus icigianus] | 475 |
      | lcl|Query\_32312 | KFX33080.1 NADH-quinone oxidoreductase subunit B [Geobacillus icigianus] | 170 |
      | lcl|Query\_32313 | KFX33081.1 NADH-quinone oxidoreductase subunit A [Geobacillus icigianus] | 122 |
      | lcl|Query\_32314 | KFX33082.1 F0F1 ATP synthase subunit epsilon [Geobacillus icigianus] | 133 |
      | lcl|Query\_32315 | KFX33083.1 ATP synthase subunit beta [Geobacillus icigianus] | 473 |
      | lcl|Query\_32316 | KFX33084.1 F0F1 ATP synthase subunit gamma [Geobacillus icigianus] | 285 |
      | lcl|Query\_32317 | KFX33085.1 ATP synthase subunit alpha [Geobacillus icigianus] | 502 |
      | lcl|Query\_32318 | KFX33086.1 F0F1 ATP synthase subunit delta [Geobacillus icigianus] | 178 |
      | lcl|Query\_32319 | KFX33087.1 ATP synthase F0 subunit B [Geobacillus icigianus] | 178 |
      | lcl|Query\_32320 | KFX33088.1 ATP synthase subunit C [Geobacillus icigianus] | 72 |
      | lcl|Query\_32321 | KFX33089.1 F0F1 ATP synthase subunit A [Geobacillus icigianus] | 237 |
      | lcl|Query\_32322 | PUA93264.1 ATP synthase subunit I [Geobacillus icigianus] | 135 |
      | lcl|Query\_32323 | KFX33090.1 hypothetical protein EP10\_10215 [Geobacillus icigianus] | 73 |
      | lcl|Query\_32324 | KFX33091.1 hypothetical protein EP10\_10220 [Geobacillus icigianus] | 70 |
      | lcl|Query\_32325 | KFX33092.1 uracil phosphoribosyltransferase [Geobacillus icigianus] | 209 |
      | lcl|Query\_32326 | KFX33093.1 serine hydroxymethyltransferase [Geobacillus icigianus] | 412 |
      | lcl|Query\_32327 | KFX33094.1 TIGR01440 family protein [Geobacillus icigianus] | 192 |
      | lcl|Query\_32328 | KFX33095.1 ribose 5-phosphate isomerase B [Geobacillus icigianus] | 150 |
      | lcl|Query\_32329 | KFX33096.1 methyl-accepting chemotaxis protein [Geobacillus icigianus] | 429 |
      | lcl|Query\_32330 | KFX33097.1 low molecular weight protein arginine phosphatase [Geobacillus icigianus] | 148 |
      | lcl|Query\_32331 | KFX33098.1 manganese efflux pump [Geobacillus icigianus] | 183 |
      | lcl|Query\_32332 | KFX33099.1 hypothetical protein EP10\_10260 [Geobacillus icigianus] | 71 |
      | lcl|Query\_32333 | KFX33100.1 threonylcarbamoyl-AMP synthase [Geobacillus icigianus] | 350 |
      | lcl|Query\_32334 | KFX33101.1 hypothetical protein EP10\_10270 [Geobacillus icigianus] | 71 |
      | lcl|Query\_32335 | KFX33102.2 stage II sporulation protein R [Geobacillus icigianus] | 274 |
      | lcl|Query\_32336 | KFX33103.1 peptide chain release factor N(5)-glutamine methyltransferase [Geobacillus icigianus] | 288 |
      | lcl|Query\_32337 | KFX33104.1 peptide chain release factor 1 [Geobacillus icigianus] | 358 |
      | lcl|Query\_32338 | KFX33105.1 hypothetical protein EP10\_10290 [Geobacillus icigianus] | 146 |
      | lcl|Query\_32339 | KFX33106.1 thymidine kinase [Geobacillus icigianus] | 206 |
      | lcl|Query\_32340 | KFX33107.1 50S ribosomal protein L31 [Geobacillus icigianus] | 66 |
      | lcl|Query\_32341 | KFX33108.1 transcription termination factor Rho [Geobacillus icigianus] | 424 |
      | lcl|Query\_32342 | KFX33109.1 fructose-bisphosphatase class II [Geobacillus icigianus] | 320 |
      | lcl|Query\_32343 | KFX33110.1 UDP-N-acetylglucosamine 1-carboxyvinyltransferase [Geobacillus icigianus] | 428 |
      | lcl|Query\_32344 | KFX33111.1 fructose-6-phosphate aldolase [Geobacillus icigianus] | 213 |
      | lcl|Query\_32345 | KFX33028.1 PBSX family phage terminase large subunit [Geobacillus icigianus] | 419 |
      | lcl|Query\_32346 | KFX33029.1 phage portal protein [Geobacillus icigianus] | 474 |
      | lcl|Query\_32347 | KFX33030.1 phage head morphogenesis protein [Geobacillus icigianus] | 485 |
      | lcl|Query\_32348 | KFX33031.1 hypothetical protein EP10\_10355 [Geobacillus icigianus] | 64 |
      | lcl|Query\_32349 | KFX33032.1 chemotaxis protein [Geobacillus icigianus] | 185 |
      | lcl|Query\_32350 | KFX33033.1 N4-gp56 family major capsid protein [Geobacillus icigianus] | 272 |
      | lcl|Query\_32351 | KFX33034.1 hypothetical protein EP10\_10375 [Geobacillus icigianus] | 113 |
      | lcl|Query\_32352 | KFX33035.1 hypothetical protein EP10\_10380 [Geobacillus icigianus] | 121 |
      | lcl|Query\_32353 | KFX33036.1 hypothetical protein EP10\_10385 [Geobacillus icigianus] | 132 |
      | lcl|Query\_32354 | KFX33037.1 hypothetical protein EP10\_10390 [Geobacillus icigianus] | 135 |
      | lcl|Query\_32355 | KFX33038.1 phage tail protein [Geobacillus icigianus] | 432 |
      | lcl|Query\_32356 | KFX33039.1 phage tail protein [Geobacillus icigianus] | 154 |
      | lcl|Query\_32357 | KFX33040.1 phage portal protein [Geobacillus icigianus] | 133 |
      | lcl|Query\_32358 | PUA93263.1 hypothetical protein EP10\_19940 [Geobacillus icigianus] | 59 |
      | lcl|Query\_32359 | KFX33041.1 phage tail tape measure protein [Geobacillus icigianus] | 746 |
      | lcl|Query\_32360 | KFX33042.1 LysM peptidoglycan-binding domain-containing protein [Geobacillus icigianus] | 224 |
      | lcl|Query\_32361 | KFX33043.1 hypothetical protein EP10\_10425 [Geobacillus icigianus] | 319 |
      | lcl|Query\_32362 | KFX33044.1 DUF2577 domain-containing protein [Geobacillus icigianus] | 111 |
      | lcl|Query\_32363 | KFX33045.1 DUF2634 domain-containing protein [Geobacillus icigianus] | 135 |
      | lcl|Query\_32364 | KFX33047.1 hypothetical protein EP10\_10445 [Geobacillus icigianus] | 128 |
      | lcl|Query\_32365 | KFX33048.1 DUF2313 domain-containing protein [Geobacillus icigianus] | 318 |
      | lcl|Query\_32366 | KFX33027.1 terminase small subunit [Geobacillus icigianus] | 169 |
      | lcl|Query\_32367 | KFX33046.1 baseplate J protein [Geobacillus icigianus] | 344 |
      | lcl|Query\_32368 | KFX33021.1 galactokinase, partial [Geobacillus icigianus] | 352 |
      | lcl|Query\_32369 | KFX33022.1 UDP-glucose 4-epimerase GalE [Geobacillus icigianus] | 328 |
      | lcl|Query\_32370 | KFX33023.1 UDP-glucose--hexose-1-phosphate uridylyltransferase [Geobacillus icigianus] | 508 |
      | lcl|Query\_32371 | KFX33024.1 LacI family DNA-binding transcriptional regulator [Geobacillus icigianus] | 339 |
      | lcl|Query\_32372 | KFX33025.1 Hsp20/alpha crystallin family protein [Geobacillus icigianus] | 147 |
      | lcl|Query\_32373 | KFX33026.1 hypothetical protein EP10\_10485 [Geobacillus icigianus] | 96 |
      | lcl|Query\_32374 | KFX32980.1 BH0509 family protein [Geobacillus icigianus] | 48 |
      | lcl|Query\_32375 | KFX32981.1 YihY/virulence factor BrkB family protein [Geobacillus icigianus] | 277 |
      | lcl|Query\_32376 | KFX32982.1 low molecular weight phosphotyrosine protein phosphatase [Geobacillus icigianus] | 160 |
      | lcl|Query\_32377 | KFX32983.1 DUF1128 domain-containing protein [Geobacillus icigianus] | 74 |
      | lcl|Query\_32378 | KFX32984.1 phosphoadenylyl-sulfate reductase [Geobacillus icigianus] | 235 |
      | lcl|Query\_32379 | KFX32985.1 sulfate adenylyltransferase [Geobacillus icigianus] | 386 |
      | lcl|Query\_32380 | KFX32986.1 adenylyl-sulfate kinase [Geobacillus icigianus] | 203 |
      | lcl|Query\_32381 | KFX32987.1 uroporphyrinogen-III C-methyltransferase [Geobacillus icigianus] | 258 |
      | lcl|Query\_32382 | KFX32988.1 sirohydrochlorin chelatase [Geobacillus icigianus] | 248 |
      | lcl|Query\_32383 | KFX32989.1 acylphosphatase [Geobacillus icigianus] | 94 |
      | lcl|Query\_32384 | KFX32990.1 arginase [Geobacillus icigianus] | 256 |
      | lcl|Query\_32385 | KFX32991.1 DUF1806 domain-containing protein [Geobacillus icigianus] | 115 |
      | lcl|Query\_32386 | KFX32992.1 bacillithiol biosynthesis deacetylase BshB2 [Geobacillus icigianus] | 217 |
      | lcl|Query\_32387 | KFX32993.1 bifunctional precorrin-2 dehydrogenase/sirohydrochlorin ferrochelatase [Geobacillus icigianus] | 221 |
      | lcl|Query\_32388 | KFX32994.1 DUF1992 domain-containing protein [Geobacillus icigianus] | 123 |
      | lcl|Query\_32389 | KFX32995.2 phosphodiesterase [Geobacillus icigianus] | 818 |
      | lcl|Query\_32390 | KFX32998.1 lactate permease [Geobacillus icigianus] | 557 |
      | lcl|Query\_32391 | KFX32999.1 FadR family transcriptional regulator [Geobacillus icigianus] | 241 |
      | lcl|Query\_32392 | KFX33000.1 NADPH:quinone oxidoreductase family protein [Geobacillus icigianus] | 327 |
      | lcl|Query\_32393 | PUA93262.1 hypothetical protein EP10\_19950 [Geobacillus icigianus] | 97 |
      | lcl|Query\_32394 | KFX33001.2 hypothetical protein EP10\_10615 [Geobacillus icigianus] | 77 |
      | lcl|Query\_32395 | KFX33004.1 ABC transporter ATP-binding protein [Geobacillus icigianus] | 307 |
      | lcl|Query\_32396 | KFX33005.1 hypothetical protein EP10\_10640 [Geobacillus icigianus] | 227 |
      | lcl|Query\_32397 | KFX33006.1 hypothetical protein EP10\_10645 [Geobacillus icigianus] | 197 |
      | lcl|Query\_32398 | KFX32996.1 NlpC/P60 family protein [Geobacillus icigianus] | 150 |
      | lcl|Query\_32399 | KFX32997.1 rod shape-determining protein [Geobacillus icigianus] | 335 |
      | lcl|Query\_32400 | PUA93261.1 pyruvate kinase [Geobacillus icigianus] | 596 |
      | lcl|Query\_32401 | KFX32936.1 toxin MazF [Geobacillus icigianus] | 128 |
      | lcl|Query\_32402 | KFX32938.1 hypothetical protein EP10\_10680 [Geobacillus icigianus] | 338 |
      | lcl|Query\_32403 | KFX32939.1 replicative DNA helicase [Geobacillus icigianus] | 500 |
      | lcl|Query\_32404 | KFX32941.1 hypothetical protein EP10\_10695 [Geobacillus icigianus] | 1470 |
      | lcl|Query\_32405 | PUA93259.1 DUF2639 domain-containing protein [Geobacillus icigianus] | 49 |
      | lcl|Query\_32406 | PUA93260.1 NETI motif-containing protein [Geobacillus icigianus] | 67 |
      | lcl|Query\_32407 | KFX32942.1 hypothetical protein EP10\_10710 [Geobacillus icigianus] | 68 |
      | lcl|Query\_32408 | KFX32943.1 sensor histidine kinase [Geobacillus icigianus] | 430 |
      | lcl|Query\_32409 | KFX32944.1 GntR family transcriptional regulator [Geobacillus icigianus] | 135 |
      | lcl|Query\_32410 | KFX32945.1 ABC transporter ATP-binding protein [Geobacillus icigianus] | 299 |
      | lcl|Query\_32411 | KFX32946.1 multidrug ABC transporter permease [Geobacillus icigianus] | 568 |
      | lcl|Query\_32412 | KFX32947.1 hypothetical protein EP10\_10745 [Geobacillus icigianus] | 75 |
      | lcl|Query\_32413 | KFX32948.1 spore germination protein [Geobacillus icigianus] | 541 |
      | lcl|Query\_32414 | KFX32949.1 Ger(x)C family spore germination protein [Geobacillus icigianus] | 401 |
      | lcl|Query\_32415 | KFX32950.1 spore gernimation protein KB [Geobacillus icigianus] | 367 |
      | lcl|Query\_32416 | KFX32951.1 PAS domain S-box protein [Geobacillus icigianus] | 479 |
      | lcl|Query\_32417 | KFX32952.2 ATP-binding protein [Geobacillus icigianus] | 147 |
      | lcl|Query\_32418 | KFX32953.1 anti-sigma factor antagonist [Geobacillus icigianus] | 148 |
      | lcl|Query\_32419 | KFX32954.1 ABC transporter ATP-binding protein [Geobacillus icigianus] | 578 |
      | lcl|Query\_32420 | KFX32955.1 ABC transporter ATP-binding protein [Geobacillus icigianus] | 599 |
      | lcl|Query\_32421 | KFX32956.1 MOSC domain-containing protein [Geobacillus icigianus] | 215 |
      | lcl|Query\_32422 | KFX32957.1 LLM class flavin-dependent oxidoreductase [Geobacillus icigianus] | 334 |
      | lcl|Query\_32423 | KFX32959.1 gamma-glutamylcyclotransferase [Geobacillus icigianus] | 129 |
      | lcl|Query\_32424 | KFX32960.1 hypothetical protein EP10\_10815, partial [Geobacillus icigianus] | 63 |
      | lcl|Query\_32425 | KFX32937.1 AbrB/MazE/SpoVT family DNA-binding domain-containing protein [Geobacillus icigianus] | 102 |
      | lcl|Query\_32426 | KFX32940.2 non-ribosomal peptide synthetase module [Geobacillus icigianus] | 239 |
      | lcl|Query\_32427 | KFX32958.1 thioredoxin family protein [Geobacillus icigianus] | 187 |
      | lcl|Query\_32428 | KFX32933.1 alpha-galactosidase [Geobacillus icigianus] | 728 |
      | lcl|Query\_32429 | KFX32934.1 EcsC family protein [Geobacillus icigianus] | 260 |
      | lcl|Query\_32430 | PUA93258.1 polyprenyl synthetase [Geobacillus icigianus] | 292 |
      | lcl|Query\_32431 | KFX32916.1 IS982 family transposase [Geobacillus icigianus] | 292 |
      | lcl|Query\_32432 | KFX32917.1 lantibiotic ABC transporter ATP-binding protein [Geobacillus icigianus] | 230 |
      | lcl|Query\_32433 | KFX32918.1 lantibiotic immunity ABC transporter MutE/EpiE family permease subunit [Geobacillus icigianus] | 248 |
      | lcl|Query\_32434 | KFX32919.1 lantibiotic immunity ABC transporter MutG family permease subunit [Geobacillus icigianus] | 262 |
      | lcl|Query\_32435 | KFX32920.1 NisI/SpaI family lantibiotic immunity lipoprotein [Geobacillus icigianus] | 144 |
      | lcl|Query\_32436 | KFX32921.1 sensor histidine kinase [Geobacillus icigianus] | 481 |
      | lcl|Query\_32437 | KFX32923.1 ABC transporter ATP-binding protein [Geobacillus icigianus] | 217 |
      | lcl|Query\_32438 | KFX32924.1 hypothetical protein EP10\_10885 [Geobacillus icigianus] | 233 |
      | lcl|Query\_32439 | KFX32925.1 hypothetical protein EP10\_10890 [Geobacillus icigianus] | 246 |
      | lcl|Query\_32440 | KFX32926.1 NADH dehydrogenase FAD-containing subunit [Geobacillus icigianus] | 293 |
      | lcl|Query\_32441 | KFX32927.1 hypothetical protein EP10\_10900 [Geobacillus icigianus] | 129 |
      | lcl|Query\_32442 | KFX32922.1 DNA-binding response regulator [Geobacillus icigianus] | 232 |
      | lcl|Query\_32443 | KFX32907.1 hypothetical protein EP10\_10905 [Geobacillus icigianus] | 308 |
      | lcl|Query\_32444 | KFX32908.1 hypothetical protein EP10\_10910 [Geobacillus icigianus] | 258 |
      | lcl|Query\_32445 | KFX32899.1 peptidase M48 Ste24p [Geobacillus icigianus] | 570 |
      | lcl|Query\_32446 | KFX32900.1 PDZ domain-containing protein [Geobacillus icigianus] | 402 |
      | lcl|Query\_32447 | KFX32901.1 DNA-binding response regulator [Geobacillus icigianus] | 223 |
      | lcl|Query\_32448 | KFX32902.1 GHKL domain-containing protein [Geobacillus icigianus] | 457 |
      | lcl|Query\_32449 | KFX32904.1 IS701 family transposase, partial [Geobacillus icigianus] | 286 |
      | lcl|Query\_32450 | KFX32821.1 diguanylate cyclase, partial [Geobacillus icigianus] | 432 |
      | lcl|Query\_32451 | KFX32822.1 DeoR/GlpR transcriptional regulator [Geobacillus icigianus] | 250 |
      | lcl|Query\_32452 | KFX32823.1 1-phosphofructokinase [Geobacillus icigianus] | 302 |
      | lcl|Query\_32453 | KFX32824.1 PTS fructose transporter subunit IIA [Geobacillus icigianus] | 623 |
      | lcl|Query\_32454 | KFX32825.1 permease [Geobacillus icigianus] | 288 |
      | lcl|Query\_32455 | KFX32826.1 TIGR03943 family protein [Geobacillus icigianus] | 282 |
      | lcl|Query\_32456 | KFX32827.1 MBL fold metallo-hydrolase [Geobacillus icigianus] | 327 |
      | lcl|Query\_32457 | KFX32828.1 hypothetical protein EP10\_11000 [Geobacillus icigianus] | 133 |
      | lcl|Query\_32458 | KFX32829.1 type I methionyl aminopeptidase [Geobacillus icigianus] | 249 |
      | lcl|Query\_32459 | KFX32830.1 sensor histidine kinase [Geobacillus icigianus] | 434 |
      | lcl|Query\_32460 | KFX32831.1 uroporphyrinogen-III synthase [Geobacillus icigianus] | 268 |
      | lcl|Query\_32461 | KFX32832.1 respiratory nitrate reductase subunit gamma [Geobacillus icigianus] | 234 |
      | lcl|Query\_32462 | KFX32833.1 nitrate reductase molybdenum cofactor assembly chaperone [Geobacillus icigianus] | 191 |
      | lcl|Query\_32463 | KFX32834.1 nitrate reductase subunit beta [Geobacillus icigianus] | 490 |
      | lcl|Query\_32464 | PUA93252.1 nitrate reductase subunit alpha [Geobacillus icigianus] | 1239 |
      | lcl|Query\_32465 | KFX32835.1 hypothetical protein EP10\_11040 [Geobacillus icigianus] | 167 |
      | lcl|Query\_32466 | KFX32837.1 tetratricopeptide repeat protein [Geobacillus icigianus] | 1386 |
      | lcl|Query\_32467 | KFX32838.1 hypothetical protein EP10\_11060 [Geobacillus icigianus] | 87 |
      | lcl|Query\_32468 | KFX32839.1 membrane protein insertase YidC [Geobacillus icigianus] | 249 |
      | lcl|Query\_32469 | KFX32841.1 nicotinate phosphoribosyltransferase [Geobacillus icigianus] | 490 |
      | lcl|Query\_32470 | PUA93253.1 oligoendopeptidase F [Geobacillus icigianus] | 630 |
      | lcl|Query\_32471 | KFX32842.1 oxidoreductase [Geobacillus icigianus] | 296 |
      | lcl|Query\_32472 | KFX32843.1 DNA topoisomerase III [Geobacillus icigianus] | 718 |
      | lcl|Query\_32473 | KFX32844.1 hypothetical protein EP10\_11100 [Geobacillus icigianus] | 72 |
      | lcl|Query\_32474 | KFX32845.1 ribonuclease E inhibitor RraA [Geobacillus icigianus] | 161 |
      | lcl|Query\_32475 | KFX32846.1 (2Fe-2S) ferredoxin domain-containing protein [Geobacillus icigianus] | 134 |
      | lcl|Query\_32476 | KFX32847.2 energy-coupling factor ABC transporter permease [Geobacillus icigianus] | 247 |
      | lcl|Query\_32477 | KFX32848.1 energy-coupling factor ABC transporter substrate-binding protein [Geobacillus icigianus] | 97 |
      | lcl|Query\_32478 | KFX32849.1 cobalt ABC transporter permease [Geobacillus icigianus] | 242 |
      | lcl|Query\_32479 | PUA93254.1 ABC transporter ATP-binding protein [Geobacillus icigianus] | 274 |
      | lcl|Query\_32480 | KFX32850.1 precorrin-3B C(17)-methyltransferase [Geobacillus icigianus] | 577 |
      | lcl|Query\_32481 | KFX32851.1 sirohydrochlorin chelatase [Geobacillus icigianus] | 277 |
      | lcl|Query\_32482 | KFX32852.1 precorrin-6A reductase [Geobacillus icigianus] | 258 |
      | lcl|Query\_32483 | KFX32853.1 precorrin-8X methylmutase [Geobacillus icigianus] | 215 |
      | lcl|Query\_32484 | KFX32854.1 bifunctional cobalt-precorrin-7 (C(5))-methyltransferase/cobalt-precorrin-6B (C(15))-methyltransferase [Geobacillus icigianus] | 401 |
      | lcl|Query\_32485 | KFX32855.1 precorrin-2 C(20)-methyltransferase [Geobacillus icigianus] | 233 |
      | lcl|Query\_32486 | KFX32856.1 precorrin-4 C(11)-methyltransferase [Geobacillus icigianus] | 262 |
      | lcl|Query\_32487 | KFX32857.2 cobalamin biosynthesis protein CbiG [Geobacillus icigianus] | 414 |
      | lcl|Query\_32488 | KFX32858.1 cobyrinate a,c-diamide synthase [Geobacillus icigianus] | 454 |
      | lcl|Query\_32489 | KFX32859.1 cobyric acid synthase [Geobacillus icigianus] | 500 |
      | lcl|Query\_32490 | KFX32861.1 uroporphyrinogen-III C-methyltransferase [Geobacillus icigianus] | 256 |
      | lcl|Query\_32491 | KFX32862.1 cob(I)yrinic acid a,c-diamide adenosyltransferase [Geobacillus icigianus] | 181 |
      | lcl|Query\_32492 | PUA93255.1 YjcZ family sporulation protein [Geobacillus icigianus] | 31 |
      | lcl|Query\_32493 | KFX32863.1 homoserine O-succinyltransferase [Geobacillus icigianus] | 302 |
      | lcl|Query\_32494 | KFX32864.1 hypothetical protein EP10\_11220 [Geobacillus icigianus] | 170 |
      | lcl|Query\_32495 | KFX32865.1 glutathione peroxidase [Geobacillus icigianus] | 163 |
      | lcl|Query\_32496 | KFX32866.1 virulence factor [Geobacillus icigianus] | 384 |
      | lcl|Query\_32497 | KFX32869.1 hypothetical protein EP10\_11250 [Geobacillus icigianus] | 194 |
      | lcl|Query\_32498 | KFX32871.1 thymidylate synthase [Geobacillus icigianus] | 264 |
      | lcl|Query\_32499 | KFX32872.1 dihydrofolate reductase [Geobacillus icigianus] | 164 |
      | lcl|Query\_32500 | KFX32873.1 DUF2512 domain-containing protein [Geobacillus icigianus] | 149 |
      | lcl|Query\_32501 | KFX32874.1 threonine dehydratase [Geobacillus icigianus] | 423 |
      | lcl|Query\_32502 | KFX32876.1 SCO family protein [Geobacillus icigianus] | 190 |
      | lcl|Query\_32503 | KFX32877.1 GDSL family lipase [Geobacillus icigianus] | 266 |
      | lcl|Query\_32504 | KFX32878.1 DUF2140 domain-containing protein [Geobacillus icigianus] | 200 |
      | lcl|Query\_32505 | KFX32879.1 NOL1/NOP2/sun family putative RNA methylase [Geobacillus icigianus] | 455 |
      | lcl|Query\_32506 | KFX32880.1 glycerol-3-phosphate 1-O-acyltransferase PlsY [Geobacillus icigianus] | 197 |
      | lcl|Query\_32507 | KFX32881.1 OsmC family peroxiredoxin [Geobacillus icigianus] | 141 |
      | lcl|Query\_32508 | KFX32882.1 bifunctional metallophosphatase/5'-nucleotidase [Geobacillus icigianus] | 540 |
      | lcl|Query\_32509 | KFX32883.1 peptidase T [Geobacillus icigianus] | 411 |
      | lcl|Query\_32510 | KFX32885.1 sulfite oxidase-like oxidoreductase [Geobacillus icigianus] | 205 |
      | lcl|Query\_32511 | KFX32886.1 DUF969 domain-containing protein [Geobacillus icigianus] | 228 |
      | lcl|Query\_32512 | KFX32887.1 DUF979 domain-containing protein [Geobacillus icigianus] | 319 |
      | lcl|Query\_32513 | KFX32888.1 pyroglutamyl-peptidase I [Geobacillus icigianus] | 215 |
      | lcl|Query\_32514 | PUA93256.1 peptidoglycan-binding protein [Geobacillus icigianus] | 49 |
      | lcl|Query\_32515 | KFX32836.1 lipase [Geobacillus icigianus] | 250 |
      | lcl|Query\_32516 | KFX32840.1 cysteine hydrolase [Geobacillus icigianus] | 183 |
      | lcl|Query\_32517 | KFX32889.1 cobalt-precorrin-5B (C(1))-methyltransferase [Geobacillus icigianus] | 372 |
      | lcl|Query\_32518 | KFX32860.1 nicotinate-nucleotide--dimethylbenzimidazole phosphoribosyltransferase [Geobacillus icigianus] | 351 |
      | lcl|Query\_32519 | KFX32867.1 BrxA/BrxB family bacilliredoxin [Geobacillus icigianus] | 145 |
      | lcl|Query\_32520 | PUA93257.1 YuzL family protein [Geobacillus icigianus] | 43 |
      | lcl|Query\_32521 | KFX32868.2 LL-diaminopimelate aminotransferase [Geobacillus icigianus] | 394 |
      | lcl|Query\_32522 | KFX32870.1 toxin [Geobacillus icigianus] | 233 |
      | lcl|Query\_32523 | KFX32875.1 DUF2535 domain-containing protein [Geobacillus icigianus] | 83 |
      | lcl|Query\_32524 | KFX32884.2 ECF transporter S component [Geobacillus icigianus] | 204 |
      | lcl|Query\_32525 | KFX32764.1 ABC transporter ATP-binding protein [Geobacillus icigianus] | 341 |
      | lcl|Query\_32526 | KFX32765.1 ABC transporter ATP-binding protein [Geobacillus icigianus] | 308 |
      | lcl|Query\_32527 | KFX32766.1 ABC transporter permease [Geobacillus icigianus] | 322 |
      | lcl|Query\_32528 | KFX32767.1 ABC transporter permease [Geobacillus icigianus] | 302 |
      | lcl|Query\_32529 | KFX32768.1 oligopeptide ABC transporter substrate-binding protein [Geobacillus icigianus] | 594 |
      | lcl|Query\_32530 | KFX32769.1 VOC family protein [Geobacillus icigianus] | 127 |
      | lcl|Query\_32531 | KFX32770.1 PaaI family thioesterase [Geobacillus icigianus] | 160 |
      | lcl|Query\_32532 | KFX32771.1 PaaI family thioesterase [Geobacillus icigianus] | 138 |
      | lcl|Query\_32533 | PUA93251.1 formimidoylglutamase [Geobacillus icigianus] | 332 |
      | lcl|Query\_32534 | KFX32772.1 AraC family transcriptional regulator [Geobacillus icigianus] | 495 |
      | lcl|Query\_32535 | KFX32773.1 urocanate hydratase [Geobacillus icigianus] | 553 |
      | lcl|Query\_32536 | KFX32774.1 imidazolonepropionase [Geobacillus icigianus] | 424 |
      | lcl|Query\_32537 | KFX32775.1 histidine transporter [Geobacillus icigianus] | 438 |
      | lcl|Query\_32538 | KFX32776.1 large conductance mechanosensitive channel protein MscL [Geobacillus icigianus] | 131 |
      | lcl|Query\_32539 | KFX32777.1 YeeE/YedE family protein [Geobacillus icigianus] | 406 |
      | lcl|Query\_32540 | KFX32778.1 hypothetical protein EP10\_11455 [Geobacillus icigianus] | 145 |
      | lcl|Query\_32541 | KFX32779.1 hypothetical protein EP10\_11460 [Geobacillus icigianus] | 388 |
      | lcl|Query\_32542 | KFX32780.1 Uma2 family endonuclease [Geobacillus icigianus] | 188 |
      | lcl|Query\_32543 | KFX32782.1 FadR family transcriptional regulator [Geobacillus icigianus] | 239 |
      | lcl|Query\_32544 | KFX32783.1 FAD-binding oxidoreductase [Geobacillus icigianus] | 470 |
      | lcl|Query\_32545 | KFX32784.2 (Fe-S)-binding protein [Geobacillus icigianus] | 437 |
      | lcl|Query\_32546 | KFX32785.1 ABC transporter substrate-binding protein [Geobacillus icigianus] | 368 |
      | lcl|Query\_32547 | KFX32786.1 ABC transporter ATP-binding protein [Geobacillus icigianus] | 368 |
      | lcl|Query\_32548 | KFX32787.1 ABC transporter permease [Geobacillus icigianus] | 310 |
      | lcl|Query\_32549 | KFX32788.2 ABC transporter permease [Geobacillus icigianus] | 264 |
      | lcl|Query\_32550 | KFX32789.1 L-2-hydroxyglutarate oxidase [Geobacillus icigianus] | 398 |
      | lcl|Query\_32551 | KFX32791.1 SAM-dependent DNA methyltransferase [Geobacillus icigianus] | 515 |
      | lcl|Query\_32552 | KFX32692.1 polysaccharide deacetylase family sporulation protein PdaB [Geobacillus icigianus] | 251 |
      | lcl|Query\_32553 | KFX32694.1 spore gernimation protein GerD [Geobacillus icigianus] | 209 |
      | lcl|Query\_32554 | KFX32696.1 N-acetylmuramoyl-L-alanine amidase CwlD [Geobacillus icigianus] | 238 |
      | lcl|Query\_32555 | KFX32697.1 DUF2521 domain-containing protein [Geobacillus icigianus] | 146 |
      | lcl|Query\_32556 | KFX32699.1 50S ribosomal protein L13 [Geobacillus icigianus] | 145 |
      | lcl|Query\_32557 | KFX32700.1 tRNA pseudouridine(38-40) synthase TruA [Geobacillus icigianus] | 255 |
      | lcl|Query\_32558 | KFX32701.1 energy-coupling factor transporter transmembrane protein EcfT [Geobacillus icigianus] | 265 |
      | lcl|Query\_32559 | KFX32702.1 energy-coupling factor transporter ATPase [Geobacillus icigianus] | 290 |
      | lcl|Query\_32560 | KFX32703.1 energy-coupling factor ABC transporter ATP-binding protein [Geobacillus icigianus] | 279 |
      | lcl|Query\_32561 | KFX32704.1 50S ribosomal protein L17 [Geobacillus icigianus] | 120 |
      | lcl|Query\_32562 | KFX32705.1 DNA-directed RNA polymerase subunit alpha [Geobacillus icigianus] | 314 |
      | lcl|Query\_32563 | KFX32706.1 30S ribosomal protein S11 [Geobacillus icigianus] | 129 |
      | lcl|Query\_32564 | KFX32707.1 30S ribosomal protein S13 [Geobacillus icigianus] | 121 |
      | lcl|Query\_32565 | KFX32708.1 50S ribosomal protein L36 [Geobacillus icigianus] | 37 |
      | lcl|Query\_32566 | KFX32709.1 translation initiation factor IF-1 [Geobacillus icigianus] | 72 |
      | lcl|Query\_32567 | KFX32710.1 type I methionyl aminopeptidase [Geobacillus icigianus] | 248 |
      | lcl|Query\_32568 | KFX32711.1 adenylate kinase [Geobacillus icigianus] | 217 |
      | lcl|Query\_32569 | KFX32712.1 preprotein translocase subunit SecY [Geobacillus icigianus] | 430 |
      | lcl|Query\_32570 | KFX32713.1 50S ribosomal protein L15 [Geobacillus icigianus] | 146 |
      | lcl|Query\_32571 | KFX32714.1 50S ribosomal protein L30 [Geobacillus icigianus] | 62 |
      | lcl|Query\_32572 | KFX32715.1 30S ribosomal protein S5 [Geobacillus icigianus] | 166 |
      | lcl|Query\_32573 | KFX32716.1 50S ribosomal protein L18 [Geobacillus icigianus] | 120 |
      | lcl|Query\_32574 | KFX32717.1 50S ribosomal protein L6 [Geobacillus icigianus] | 178 |
      | lcl|Query\_32575 | KFX32718.1 30S ribosomal protein S8 [Geobacillus icigianus] | 132 |
      | lcl|Query\_32576 | KFX32719.1 30S ribosomal protein S14 type Z [Geobacillus icigianus] | 61 |
      | lcl|Query\_32577 | KFX32720.1 50S ribosomal protein L5 [Geobacillus icigianus] | 179 |
      | lcl|Query\_32578 | KFX32721.1 50S ribosomal protein L24 [Geobacillus icigianus] | 103 |
      | lcl|Query\_32579 | KFX32722.1 50S ribosomal protein L14 [Geobacillus icigianus] | 122 |
      | lcl|Query\_32580 | KFX32723.1 30S ribosomal protein S17 [Geobacillus icigianus] | 87 |
      | lcl|Query\_32581 | KFX32724.1 50S ribosomal protein L29 [Geobacillus icigianus] | 66 |
      | lcl|Query\_32582 | KFX32725.1 50S ribosomal protein L16 [Geobacillus icigianus] | 141 |
      | lcl|Query\_32583 | KFX32726.1 30S ribosomal protein S3 [Geobacillus icigianus] | 218 |
      | lcl|Query\_32584 | KFX32727.1 50S ribosomal protein L22 [Geobacillus icigianus] | 113 |
      | lcl|Query\_32585 | KFX32728.1 30S ribosomal protein S19 [Geobacillus icigianus] | 92 |
      | lcl|Query\_32586 | KFX32729.1 50S ribosomal protein L2 [Geobacillus icigianus] | 276 |
      | lcl|Query\_32587 | KFX32730.1 50S ribosomal protein L23 [Geobacillus icigianus] | 95 |
      | lcl|Query\_32588 | KFX32731.1 50S ribosomal protein L4 [Geobacillus icigianus] | 207 |
      | lcl|Query\_32589 | KFX32732.1 50S ribosomal protein L3 [Geobacillus icigianus] | 213 |
      | lcl|Query\_32590 | KFX32733.1 30S ribosomal protein S10 [Geobacillus icigianus] | 102 |
      | lcl|Query\_32591 | KFX32734.1 elongation factor Tu [Geobacillus icigianus] | 395 |
      | lcl|Query\_32592 | KFX32735.1 elongation factor G [Geobacillus icigianus] | 692 |
      | lcl|Query\_32593 | KFX32736.1 30S ribosomal protein S7 [Geobacillus icigianus] | 156 |
      | lcl|Query\_32594 | KFX32737.1 30S ribosomal protein S12 [Geobacillus icigianus] | 140 |
      | lcl|Query\_32595 | KFX32738.1 50S ribosomal protein L7ae-like protein [Geobacillus icigianus] | 82 |
      | lcl|Query\_32596 | KFX32739.1 DNA-directed RNA polymerase subunit beta' [Geobacillus icigianus] | 1199 |
      | lcl|Query\_32597 | KFX32740.1 DNA-directed RNA polymerase subunit beta [Geobacillus icigianus] | 1190 |
      | lcl|Query\_32598 | KFX32741.1 class I SAM-dependent methyltransferase [Geobacillus icigianus] | 200 |
      | lcl|Query\_32599 | KFX32742.1 50S ribosomal protein L7/L12 [Geobacillus icigianus] | 122 |
      | lcl|Query\_32600 | KFX32743.1 50S ribosomal protein L10 [Geobacillus icigianus] | 166 |
      | lcl|Query\_32601 | KFX32744.1 50S ribosomal protein L1 [Geobacillus icigianus] | 233 |
      | lcl|Query\_32602 | KFX32745.2 50S ribosomal protein L11 [Geobacillus icigianus] | 152 |
      | lcl|Query\_32603 | KFX32746.1 transcription termination/antitermination protein NusG [Geobacillus icigianus] | 177 |
      | lcl|Query\_32604 | KFX32747.1 preprotein translocase subunit SecE [Geobacillus icigianus] | 60 |
      | lcl|Query\_32605 | KFX32748.1 RNA polymerase sporulation sigma factor SigH [Geobacillus icigianus] | 216 |
      | lcl|Query\_32606 | KFX32749.1 NYN domain-containing protein [Geobacillus icigianus] | 170 |
      | lcl|Query\_32607 | KFX32750.1 23S rRNA (guanosine(2251)-2'-O)-methyltransferase RlmB [Geobacillus icigianus] | 245 |
      | lcl|Query\_32608 | KFX32751.1 ribonuclease III [Geobacillus icigianus] | 140 |
      | lcl|Query\_32609 | KFX32752.1 cysteine--tRNA ligase [Geobacillus icigianus] | 467 |
      | lcl|Query\_32610 | KFX32753.1 serine O-acetyltransferase [Geobacillus icigianus] | 224 |
      | lcl|Query\_32611 | KFX32754.1 glutamate--tRNA ligase [Geobacillus icigianus] | 490 |
      | lcl|Query\_32612 | KFX32755.1 2-C-methyl-D-erythritol 2,4-cyclodiphosphate synthase [Geobacillus icigianus] | 160 |
      | lcl|Query\_32613 | KFX32756.1 2-C-methyl-D-erythritol 4-phosphate cytidylyltransferase [Geobacillus icigianus] | 228 |
      | lcl|Query\_32614 | KFX32757.1 PIN/TRAM domain-containing protein [Geobacillus icigianus] | 364 |
      | lcl|Query\_32615 | KFX32758.1 DNA repair protein RadA [Geobacillus icigianus] | 456 |
      | lcl|Query\_32616 | KFX32759.1 ATP-dependent Clp protease ATP-binding subunit [Geobacillus icigianus] | 811 |
      | lcl|Query\_32617 | KFX32760.1 protein arginine kinase [Geobacillus icigianus] | 363 |
      | lcl|Query\_32618 | KFX32761.1 hypothetical protein EP10\_11930 [Geobacillus icigianus] | 182 |
      | lcl|Query\_32619 | KFX32762.1 CtsR family transcriptional regulator [Geobacillus icigianus] | 153 |
      | lcl|Query\_32620 | KFX32693.1 KinB-signaling pathway activation protein [Geobacillus icigianus] | 199 |
      | lcl|Query\_32621 | KFX32695.1 ATP-binding protein [Geobacillus icigianus] | 339 |
      | lcl|Query\_32622 | KFX32698.1 30S ribosomal protein S9 [Geobacillus icigianus] | 130 |
      | lcl|Query\_32623 | KFX32603.1 spore maturation protein [Geobacillus icigianus] | 198 |
      | lcl|Query\_32624 | KFX32604.1 spore maturation protein [Geobacillus icigianus] | 177 |
      | lcl|Query\_32625 | KFX32605.1 rRNA pseudouridine synthase [Geobacillus icigianus] | 243 |
      | lcl|Query\_32626 | KFX32606.1 thiol-disulfide oxidoreductase [Geobacillus icigianus] | 174 |
      | lcl|Query\_32627 | KFX32607.1 cytochrome c biogenesis protein [Geobacillus icigianus] | 552 |
      | lcl|Query\_32628 | KFX32608.1 c-type cytochrome biogenesis protein CcsB [Geobacillus icigianus] | 395 |
      | lcl|Query\_32629 | KFX32609.1 DNA-binding response regulator [Geobacillus icigianus] | 241 |
      | lcl|Query\_32630 | KFX32610.1 HAMP domain-containing protein [Geobacillus icigianus] | 597 |
      | lcl|Query\_32631 | KFX32611.1 N-acetylglucosamine-6-phosphate deacetylase [Geobacillus icigianus] | 406 |
      | lcl|Query\_32632 | KFX32612.1 glucosamine-6-phosphate deaminase [Geobacillus icigianus] | 253 |
      | lcl|Query\_32633 | KFX32613.1 GntR family transcriptional regulator [Geobacillus icigianus] | 243 |
      | lcl|Query\_32634 | KFX32615.1 hypothetical protein EP10\_12025 [Geobacillus icigianus] | 62 |
      | lcl|Query\_32635 | KFX32616.1 ABC transporter substrate-binding protein [Geobacillus icigianus] | 319 |
      | lcl|Query\_32636 | KFX32617.2 ABC transporter permease [Geobacillus icigianus] | 337 |
      | lcl|Query\_32637 | KFX32618.1 ABC transporter ATP-binding protein [Geobacillus icigianus] | 491 |
      | lcl|Query\_32638 | KFX32619.1 cobalamin biosynthesis protein [Geobacillus icigianus] | 323 |
      | lcl|Query\_32639 | KFX32620.1 threonine-phosphate decarboxylase [Geobacillus icigianus] | 356 |
      | lcl|Query\_32640 | KFX32621.1 cobalamin biosynthesis protein [Geobacillus icigianus] | 185 |
      | lcl|Query\_32641 | KFX32622.1 adenosylcobinamide-GDP ribazoletransferase [Geobacillus icigianus] | 262 |
      | lcl|Query\_32642 | KFX32623.1 phosphoglycerate mutase [Geobacillus icigianus] | 216 |
      | lcl|Query\_32643 | KFX32624.1 cobalamin biosynthesis protein [Geobacillus icigianus] | 138 |
      | lcl|Query\_32644 | KFX32625.1 cob(I)yrinic acid a,c-diamide adenosyltransferase [Geobacillus icigianus] | 192 |
      | lcl|Query\_32645 | KFX32626.1 ECF transporter S component [Geobacillus icigianus] | 162 |
      | lcl|Query\_32646 | KFX32627.1 ATPase [Geobacillus icigianus] | 238 |
      | lcl|Query\_32647 | KFX32629.1 UDP-glucose 6-dehydrogenase [Geobacillus icigianus] | 370 |
      | lcl|Query\_32648 | KFX32630.1 histidinol phosphatase [Geobacillus icigianus] | 270 |
      | lcl|Query\_32649 | KFX32631.1 phosphoglycerate dehydrogenase [Geobacillus icigianus] | 524 |
      | lcl|Query\_32650 | KFX32632.1 inorganic diphosphatase [Geobacillus icigianus] | 167 |
      | lcl|Query\_32651 | KFX32634.1 Rrf2 family transcriptional regulator [Geobacillus icigianus] | 349 |
      | lcl|Query\_32652 | KFX32635.1 ATP-dependent DNA helicase RecQ [Geobacillus icigianus] | 502 |
      | lcl|Query\_32653 | KFX32636.1 CPBP family intramembrane metalloprotease [Geobacillus icigianus] | 194 |
      | lcl|Query\_32654 | KFX32637.1 LysM peptidoglycan-binding domain-containing protein [Geobacillus icigianus] | 179 |
      | lcl|Query\_32655 | KFX32638.1 DUF2663 domain-containing protein [Geobacillus icigianus] | 148 |
      | lcl|Query\_32656 | KFX32639.1 hypothetical protein EP10\_12155 [Geobacillus icigianus] | 126 |
      | lcl|Query\_32657 | KFX32640.1 genetic competence negative regulator [Geobacillus icigianus] | 196 |
      | lcl|Query\_32658 | KFX32641.1 Glu/Leu/Phe/Val dehydrogenase [Geobacillus icigianus] | 423 |
      | lcl|Query\_32659 | KFX32642.1 YpdA family putative bacillithiol disulfide reductase [Geobacillus icigianus] | 326 |
      | lcl|Query\_32660 | KFX32643.1 asparaginase [Geobacillus icigianus] | 322 |
      | lcl|Query\_32661 | KFX32644.1 PrsW family intramembrane metalloprotease [Geobacillus icigianus] | 225 |
      | lcl|Query\_32662 | KFX32645.1 spore cortex-lytic enzyme [Geobacillus icigianus] | 264 |
      | lcl|Query\_32663 | KFX32646.1 germination protein YpeB [Geobacillus icigianus] | 447 |
      | lcl|Query\_32664 | KFX32647.1 pilus assembly protein PilZ [Geobacillus icigianus] | 218 |
      | lcl|Query\_32665 | KFX32649.1 (d)CMP kinase [Geobacillus icigianus] | 224 |
      | lcl|Query\_32666 | KFX32650.1 1-acyl-sn-glycerol-3-phosphate acyltransferase [Geobacillus icigianus] | 195 |
      | lcl|Query\_32667 | KFX32651.1 30S ribosomal protein S1 [Geobacillus icigianus] | 387 |
      | lcl|Query\_32668 | KFX32652.1 hypothetical protein EP10\_12225 [Geobacillus icigianus] | 115 |
      | lcl|Query\_32669 | PUA93250.1 YpzI family protein [Geobacillus icigianus] | 44 |
      | lcl|Query\_32670 | KFX32653.1 hypothetical protein EP10\_12235 [Geobacillus icigianus] | 147 |
      | lcl|Query\_32671 | KFX32654.1 hypothetical protein EP10\_12240 [Geobacillus icigianus] | 104 |
      | lcl|Query\_32672 | KFX32655.1 hypothetical protein EP10\_12245 [Geobacillus icigianus] | 198 |
      | lcl|Query\_32673 | KFX32656.1 hypothetical protein EP10\_12250 [Geobacillus icigianus] | 300 |
      | lcl|Query\_32674 | KFX32657.1 hypothetical protein EP10\_12255 [Geobacillus icigianus] | 61 |
      | lcl|Query\_32675 | KFX32658.1 ribosome biogenesis GTPase Der [Geobacillus icigianus] | 436 |
      | lcl|Query\_32676 | KFX32659.1 NAD(P)H-dependent glycerol-3-phosphate dehydrogenase [Geobacillus icigianus] | 345 |
      | lcl|Query\_32677 | KFX32660.1 DUF2768 domain-containing protein [Geobacillus icigianus] | 67 |
      | lcl|Query\_32678 | KFX32661.1 hypothetical protein EP10\_12280 [Geobacillus icigianus] | 240 |
      | lcl|Query\_32679 | KFX32662.1 stage IV sporulation protein A [Geobacillus icigianus] | 492 |
      | lcl|Query\_32680 | KFX32663.1 hypothetical protein EP10\_12290 [Geobacillus icigianus] | 93 |
      | lcl|Query\_32681 | KFX32664.2 DNA-binding protein [Geobacillus icigianus] | 107 |
      | lcl|Query\_32682 | KFX32665.1 GTP cyclohydrolase I FolE [Geobacillus icigianus] | 188 |
      | lcl|Query\_32683 | KFX32666.1 trp RNA-binding attenuation protein MtrB [Geobacillus icigianus] | 74 |
      | lcl|Query\_32684 | KFX32668.1 demethylmenaquinone methyltransferase [Geobacillus icigianus] | 234 |
      | lcl|Query\_32685 | KFX32669.1 heptaprenyl diphosphate synthase component II [Geobacillus icigianus] | 320 |
      | lcl|Query\_32686 | KFX32670.1 nucleoside-diphosphate kinase [Geobacillus icigianus] | 149 |
      | lcl|Query\_32687 | KFX32671.1 protein-glutamate O-methyltransferase CheR [Geobacillus icigianus] | 256 |
      | lcl|Query\_32688 | KFX32672.1 chorismate synthase [Geobacillus icigianus] | 388 |
      | lcl|Query\_32689 | KFX32673.1 3-dehydroquinate synthase [Geobacillus icigianus] | 366 |
      | lcl|Query\_32690 | KFX32674.1 chorismate mutase [Geobacillus icigianus] | 130 |
      | lcl|Query\_32691 | KFX32675.1 anthranilate synthase component I [Geobacillus icigianus] | 508 |
      | lcl|Query\_32692 | KFX32676.1 anthranilate phosphoribosyltransferase [Geobacillus icigianus] | 339 |
      | lcl|Query\_32693 | KFX32677.1 indole-3-glycerol phosphate synthase TrpC [Geobacillus icigianus] | 266 |
      | lcl|Query\_32694 | KFX32678.1 phosphoribosylanthranilate isomerase [Geobacillus icigianus] | 217 |
      | lcl|Query\_32695 | KFX32679.1 tryptophan synthase subunit beta [Geobacillus icigianus] | 404 |
      | lcl|Query\_32696 | KFX32680.1 tryptophan synthase subunit alpha [Geobacillus icigianus] | 274 |
      | lcl|Query\_32697 | KFX32681.1 histidinol-phosphate transaminase [Geobacillus icigianus] | 365 |
      | lcl|Query\_32698 | KFX32682.1 prephenate dehydrogenase [Geobacillus icigianus] | 367 |
      | lcl|Query\_32699 | KFX32601.2 superoxide dismutase [Geobacillus icigianus] | 378 |
      | lcl|Query\_32700 | KFX32602.1 D-alanyl-D-alanine carboxypeptidase [Geobacillus icigianus] | 371 |
      | lcl|Query\_32701 | KFX32614.1 hypothetical protein EP10\_12020 [Geobacillus icigianus] | 204 |
      | lcl|Query\_32702 | KFX32628.1 RNA polymerase sigma factor SigX [Geobacillus icigianus] | 181 |
      | lcl|Query\_32703 | KFX32633.1 ferredoxin [Geobacillus icigianus] | 82 |
      | lcl|Query\_32704 | KFX32648.1 hypothetical protein EP10\_12205 [Geobacillus icigianus] | 59 |
      | lcl|Query\_32705 | KFX32667.2 trans-hexaprenyltranstransferase [Geobacillus icigianus] | 258 |
      | lcl|Query\_32706 | KFX32683.1 3-phosphoshikimate 1-carboxyvinyltransferase, partial [Geobacillus icigianus] | 421 |
      | lcl|Query\_32707 | PUA93249.1 hypothetical protein EP10\_20080, partial [Geobacillus icigianus] | 255 |
      | lcl|Query\_32708 | KFX32567.1 LysR family transcriptional regulator [Geobacillus icigianus] | 292 |
      | lcl|Query\_32709 | KFX32568.1 cation transporter [Geobacillus icigianus] | 307 |
      | lcl|Query\_32710 | KFX32569.1 ArsR family transcriptional regulator [Geobacillus icigianus] | 102 |
      | lcl|Query\_32711 | KFX32570.1 YbjQ family protein [Geobacillus icigianus] | 105 |
      | lcl|Query\_32712 | KFX32571.1 MBL fold metallo-hydrolase [Geobacillus icigianus] | 280 |
      | lcl|Query\_32713 | KFX32572.1 FAD-binding oxidoreductase [Geobacillus icigianus] | 375 |
      | lcl|Query\_32714 | KFX32573.1 IDEAL domain-containing protein [Geobacillus icigianus] | 184 |
      | lcl|Query\_32715 | KFX32574.1 glycosyltransferase family 2 protein [Geobacillus icigianus] | 448 |
      | lcl|Query\_32716 | KFX32575.1 malate:quinone oxidoreductase [Geobacillus icigianus] | 501 |
      | lcl|Query\_32717 | KFX32576.1 hypothetical protein EP10\_12455 [Geobacillus icigianus] | 163 |
      | lcl|Query\_32718 | KFX32578.1 hypothetical protein EP10\_12470 [Geobacillus icigianus] | 172 |
      | lcl|Query\_32719 | KFX32579.1 hypothetical protein EP10\_12475 [Geobacillus icigianus] | 178 |
      | lcl|Query\_32720 | KFX32582.1 ATP-dependent helicase [Geobacillus icigianus] | 802 |
      | lcl|Query\_32721 | KFX32583.1 type I restriction endonuclease subunit R [Geobacillus icigianus] | 1020 |
      | lcl|Query\_32722 | PUA93248.1 restriction endonuclease subunit S, partial [Geobacillus icigianus] | 54 |
      | lcl|Query\_32723 | KFX32580.1 DUF2075 domain-containing protein [Geobacillus icigianus] | 556 |
      | lcl|Query\_32724 | KFX32581.1 hypothetical protein EP10\_12485 [Geobacillus icigianus] | 714 |
      | lcl|Query\_32725 | PUA93247.1 DUF2487 domain-containing protein, partial [Geobacillus icigianus] | 46 |
      | lcl|Query\_32726 | KFX32551.1 hypothetical protein EP10\_12505 [Geobacillus icigianus] | 177 |
      | lcl|Query\_32727 | KFX32552.1 tetratricopeptide repeat protein [Geobacillus icigianus] | 418 |
      | lcl|Query\_32728 | KFX32527.1 phenylacetate--CoA ligase [Geobacillus icigianus] | 441 |
      | lcl|Query\_32729 | KFX32528.1 1,2-phenylacetyl-CoA epoxidase subunit A [Geobacillus icigianus] | 322 |
      | lcl|Query\_32730 | KFX32529.1 1,2-phenylacetyl-CoA epoxidase subunit B [Geobacillus icigianus] | 117 |
      | lcl|Query\_32731 | KFX32530.1 phenylacetate-CoA oxygenase subunit PaaI [Geobacillus icigianus] | 275 |
      | lcl|Query\_32732 | KFX32531.1 phenylacetate-CoA oxygenase subunit PaaJ [Geobacillus icigianus] | 158 |
      | lcl|Query\_32733 | KFX32532.1 EthD family reductase [Geobacillus icigianus] | 103 |
      | lcl|Query\_32734 | KFX32533.1 enoyl-CoA hydratase [Geobacillus icigianus] | 258 |
      | lcl|Query\_32735 | KFX32534.1 2-(1,2-epoxy-1,2-dihydrophenyl)acetyl-CoA isomerase [Geobacillus icigianus] | 257 |
      | lcl|Query\_32736 | KFX32535.1 aldehyde dehydrogenase [Geobacillus icigianus] | 505 |
      | lcl|Query\_32737 | KFX32536.1 3-hydroxyacyl-CoA dehydrogenase [Geobacillus icigianus] | 287 |
      | lcl|Query\_32738 | KFX32537.1 acetyl-CoA C-acyltransferase [Geobacillus icigianus] | 403 |
      | lcl|Query\_32739 | KFX32538.1 phenylacetic acid degradation operon negative regulatory protein PaaX [Geobacillus icigianus] | 285 |
      | lcl|Query\_32740 | KFX32539.1 gamma carbonic anhydrase family protein [Geobacillus icigianus] | 184 |
      | lcl|Query\_32741 | KFX32540.1 DUF561 domain-containing protein [Geobacillus icigianus] | 316 |
      | lcl|Query\_32742 | KFX32541.1 Glu/Leu/Phe/Val dehydrogenase [Geobacillus icigianus] | 380 |
      | lcl|Query\_32743 | KFX32542.1 ABC transporter substrate-binding protein [Geobacillus icigianus] | 337 |
      | lcl|Query\_32744 | KFX32543.1 ABC transporter permease [Geobacillus icigianus] | 330 |
      | lcl|Query\_32745 | KFX32544.1 ABC transporter ATP-binding protein [Geobacillus icigianus] | 264 |
      | lcl|Query\_32746 | KFX32545.1 pyruvate dehydrogenase (acetyl-transferring) E1 component subunit alpha [Geobacillus icigianus] | 356 |
      | lcl|Query\_32747 | KFX32546.1 alpha-ketoacid dehydrogenase subunit beta [Geobacillus icigianus] | 331 |
      | lcl|Query\_32748 | KFX32547.1 gamma carbonic anhydrase family protein [Geobacillus icigianus] | 174 |
      | lcl|Query\_32749 | KFX32453.1 glycosyltransferase family 1 protein, partial [Geobacillus icigianus] | 239 |
      | lcl|Query\_32750 | KFX32454.1 IS110 family transposase [Geobacillus icigianus] | 378 |
      | lcl|Query\_32751 | KFX32449.1 hypothetical protein EP10\_12635 [Geobacillus icigianus] | 156 |
      | lcl|Query\_32752 | PUA93242.1 RNA polymerase sigma factor, partial [Geobacillus icigianus] | 59 |
      | lcl|Query\_32753 | PUA93243.1 sigma-70 family RNA polymerase sigma factor [Geobacillus icigianus] | 85 |
      | lcl|Query\_32754 | KFX32413.1 zf-HC2 domain-containing protein [Geobacillus icigianus] | 159 |
      | lcl|Query\_32755 | PUA93244.1 hypothetical protein EP10\_20110 [Geobacillus icigianus] | 105 |
      | lcl|Query\_32756 | KFX32414.1 Lrp/AsnC family transcriptional regulator [Geobacillus icigianus] | 151 |
      | lcl|Query\_32757 | KFX32415.1 carboxymuconolactone decarboxylase family protein [Geobacillus icigianus] | 142 |
      | lcl|Query\_32758 | KFX32416.1 ATP-dependent DNA helicase [Geobacillus icigianus] | 645 |
      | lcl|Query\_32759 | KFX32417.1 carboxypeptidase M32 [Geobacillus icigianus] | 500 |
      | lcl|Query\_32760 | KFX32418.1 cytochrome C [Geobacillus icigianus] | 549 |
      | lcl|Query\_32761 | KFX32419.1 cytochrome B5 [Geobacillus icigianus] | 156 |
      | lcl|Query\_32762 | KFX32420.1 cytochrome c oxidase subunit 2A [Geobacillus icigianus] | 51 |
      | lcl|Query\_32763 | KFX32421.1 xanthine phosphoribosyltransferase [Geobacillus icigianus] | 195 |
      | lcl|Query\_32764 | KFX32422.1 purine permease [Geobacillus icigianus] | 435 |
      | lcl|Query\_32765 | KFX32423.1 hypothetical protein EP10\_12710 [Geobacillus icigianus] | 232 |
      | lcl|Query\_32766 | KFX32424.1 type III polyketide synthase [Geobacillus icigianus] | 355 |
      | lcl|Query\_32767 | KFX32426.1 dynamin [Geobacillus icigianus] | 1251 |
      | lcl|Query\_32768 | KFX32427.1 hypothetical protein EP10\_12735 [Geobacillus icigianus] | 61 |
      | lcl|Query\_32769 | PUA93245.1 DUF2533 domain-containing protein [Geobacillus icigianus] | 101 |
      | lcl|Query\_32770 | KFX32428.1 sulfurtransferase [Geobacillus icigianus] | 282 |
      | lcl|Query\_32771 | KFX32429.1 TlpA family protein disulfide reductase [Geobacillus icigianus] | 173 |
      | lcl|Query\_32772 | PUA93246.1 FbpB family small basic protein [Geobacillus icigianus] | 43 |
      | lcl|Query\_32773 | KFX32430.1 small, acid-soluble spore protein N [Geobacillus icigianus] | 47 |
      | lcl|Query\_32774 | KFX32431.1 small acid-soluble spore protein Tlp [Geobacillus icigianus] | 75 |
      | lcl|Query\_32775 | KFX32432.1 acyl-CoA thioesterase [Geobacillus icigianus] | 138 |
      | lcl|Query\_32776 | KFX32433.1 hypothetical protein EP10\_12780 [Geobacillus icigianus] | 104 |
      | lcl|Query\_32777 | KFX32434.1 hotdog fold thioesterase [Geobacillus icigianus] | 135 |
      | lcl|Query\_32778 | KFX32435.1 hypothetical protein EP10\_12790 [Geobacillus icigianus] | 96 |
      | lcl|Query\_32779 | KFX32436.1 magnesium transporter CorA family protein [Geobacillus icigianus] | 312 |
      | lcl|Query\_32780 | KFX32437.1 DUF502 domain-containing protein [Geobacillus icigianus] | 197 |
      | lcl|Query\_32781 | KFX32438.1 NAD(P)/FAD-dependent oxidoreductase [Geobacillus icigianus] | 479 |
      | lcl|Query\_32782 | KFX32425.1 hypothetical protein EP10\_12720 [Geobacillus icigianus] | 192 |
      | lcl|Query\_32783 | KFX32439.1 IS4 family transposase, partial [Geobacillus icigianus] | 313 |
      | lcl|Query\_32784 | KFX32387.1 hypothetical protein EP10\_12825 [Geobacillus icigianus] | 110 |
      | lcl|Query\_32785 | KFX32380.1 spore coat protein, partial [Geobacillus icigianus] | 29 |
      | lcl|Query\_32786 | KFX32381.1 hypothetical protein EP10\_12835 [Geobacillus icigianus] | 415 |
      | lcl|Query\_32787 | KFX32382.1 DUF5082 domain-containing protein [Geobacillus icigianus] | 96 |
      | lcl|Query\_32788 | KFX32383.1 hypothetical protein EP10\_12845 [Geobacillus icigianus] | 79 |
      | lcl|Query\_32789 | KFX32384.1 IS110 family transposase [Geobacillus icigianus] | 427 |
      | lcl|Query\_32790 | KFX32385.2 hypothetical protein EP10\_12855 [Geobacillus icigianus] | 64 |
      | lcl|Query\_32791 | KFX32339.1 hypothetical protein EP10\_12860, partial [Geobacillus icigianus] | 122 |
      | lcl|Query\_32792 | KFX32340.1 replicative DNA helicase [Geobacillus icigianus] | 419 |
      | lcl|Query\_32793 | PUA93238.1 hypothetical protein EP10\_20125 [Geobacillus icigianus] | 161 |
      | lcl|Query\_32794 | KFX32341.1 hypothetical protein EP10\_12880 [Geobacillus icigianus] | 65 |
      | lcl|Query\_32795 | KFX32342.1 hypothetical protein EP10\_12885 [Geobacillus icigianus] | 61 |
      | lcl|Query\_32796 | PUA93239.1 hypothetical protein EP10\_20130 [Geobacillus icigianus] | 84 |
      | lcl|Query\_32797 | KFX32343.1 RusA family crossover junction endodeoxyribonuclease [Geobacillus icigianus] | 139 |
      | lcl|Query\_32798 | KFX32344.1 hypothetical protein EP10\_12895 [Geobacillus icigianus] | 152 |
      | lcl|Query\_32799 | PUA93240.1 hypothetical protein EP10\_20135 [Geobacillus icigianus] | 93 |
      | lcl|Query\_32800 | KFX32345.1 Fis family transcriptional regulator [Geobacillus icigianus] | 166 |
      | lcl|Query\_32801 | KFX32346.1 hypothetical protein EP10\_12910 [Geobacillus icigianus] | 350 |
      | lcl|Query\_32802 | KFX32347.1 hypothetical protein EP10\_12915 [Geobacillus icigianus] | 73 |
      | lcl|Query\_32803 | PUA93241.1 hypothetical protein EP10\_20140 [Geobacillus icigianus] | 81 |
      | lcl|Query\_32804 | KFX32348.1 hypothetical protein EP10\_12925, partial [Geobacillus icigianus] | 114 |
      | lcl|Query\_32805 | KFX32335.1 ABC transporter ATP-binding protein [Geobacillus icigianus] | 372 |
      | lcl|Query\_32806 | KFX32336.2 sugar ABC transporter permease, partial [Geobacillus icigianus] | 248 |
      | lcl|Query\_32807 | KFX32300.1 phosphopyruvate hydratase [Geobacillus icigianus] | 430 |
      | lcl|Query\_32808 | KFX32301.1 2,3-bisphosphoglycerate-independent phosphoglycerate mutase [Geobacillus icigianus] | 511 |
      | lcl|Query\_32809 | KFX32302.1 triose-phosphate isomerase [Geobacillus icigianus] | 253 |
      | lcl|Query\_32810 | KFX32303.1 phosphoglycerate kinase [Geobacillus icigianus] | 394 |
      | lcl|Query\_32811 | KFX32304.1 type I glyceraldehyde-3-phosphate dehydrogenase [Geobacillus icigianus] | 335 |
      | lcl|Query\_32812 | KFX32305.1 hypothetical protein EP10\_12965 [Geobacillus icigianus] | 346 |
      | lcl|Query\_32813 | KFX32306.1 glutaredoxin family protein [Geobacillus icigianus] | 81 |
      | lcl|Query\_32814 | KFX32307.1 RNA polymerase sigma-54 factor [Geobacillus icigianus] | 435 |
      | lcl|Query\_32815 | KFX32308.1 ATP-dependent Clp endopeptidase proteolytic subunit ClpP [Geobacillus icigianus] | 196 |
      | lcl|Query\_32816 | KFX32309.1 HPr family phosphocarrier protein [Geobacillus icigianus] | 85 |
      | lcl|Query\_32817 | KFX32310.1 DNA-binding protein WhiA [Geobacillus icigianus] | 320 |
      | lcl|Query\_32818 | KFX32311.1 YvcK family protein [Geobacillus icigianus] | 317 |
      | lcl|Query\_32819 | KFX32312.1 RNase adapter RapZ [Geobacillus icigianus] | 298 |
      | lcl|Query\_32820 | KFX32314.1 thioredoxin-disulfide reductase [Geobacillus icigianus] | 318 |
      | lcl|Query\_32821 | KFX32315.1 tetratricopeptide repeat protein [Geobacillus icigianus] | 490 |
      | lcl|Query\_32822 | KFX32316.1 bifunctional phosphoribosyl-AMP cyclohydrolase/phosphoribosyl-ATP diphosphatase HisIE [Geobacillus icigianus] | 216 |
      | lcl|Query\_32823 | KFX32317.1 imidazole glycerol phosphate synthase subunit HisF [Geobacillus icigianus] | 252 |
      | lcl|Query\_32824 | KFX32318.1 1-(5-phosphoribosyl)-5-[(5-phosphoribosylamino)methylideneamino]imidazole-4-carboxamide isomerase [Geobacillus icigianus] | 245 |
      | lcl|Query\_32825 | KFX32319.1 imidazole glycerol phosphate synthase subunit HisH [Geobacillus icigianus] | 213 |
      | lcl|Query\_32826 | KFX32321.1 imidazoleglycerol-phosphate dehydratase HisB [Geobacillus icigianus] | 195 |
      | lcl|Query\_32827 | KFX32322.1 histidinol dehydrogenase [Geobacillus icigianus] | 427 |
      | lcl|Query\_32828 | KFX32324.1 ATP phosphoribosyltransferase regulatory subunit [Geobacillus icigianus] | 394 |
      | lcl|Query\_32829 | KFX32325.1 hypothetical protein EP10\_13070 [Geobacillus icigianus] | 82 |
      | lcl|Query\_32830 | PUA93235.1 hypothetical protein EP10\_20155 [Geobacillus icigianus] | 71 |
      | lcl|Query\_32831 | KFX32313.1 8-oxo-dGTP diphosphatase [Geobacillus icigianus] | 152 |
      | lcl|Query\_32832 | KFX32323.1 ATP phosphoribosyltransferase [Geobacillus icigianus] | 207 |
      | lcl|Query\_32833 | PUA93236.1 hypothetical protein EP10\_20150 [Geobacillus icigianus] | 19 |
      | lcl|Query\_32834 | PUA93237.1 teicoplanin resistance protein VanZ, partial [Geobacillus icigianus] | 73 |
      | lcl|Query\_32835 | KFX32276.1 ferrous iron transport protein A [Geobacillus icigianus] | 73 |
      | lcl|Query\_32836 | KFX32277.1 ferrous iron transport protein B [Geobacillus icigianus] | 664 |
      | lcl|Query\_32837 | PUA93234.1 FeoB-associated Cys-rich membrane protein [Geobacillus icigianus] | 49 |
      | lcl|Query\_32838 | KFX32278.1 hypothetical protein EP10\_13090 [Geobacillus icigianus] | 199 |
      | lcl|Query\_32839 | PUA93233.1 hypothetical protein EP10\_20175, partial [Geobacillus icigianus] | 241 |
      | lcl|Query\_32840 | KFX32275.1 recombinase family protein, partial [Geobacillus icigianus] | 175 |
      | lcl|Query\_32841 | PUA93232.1 hypothetical protein EP10\_20180, partial [Geobacillus icigianus] | 144 |
      | lcl|Query\_32842 | KFX32247.1 hypothetical protein EP10\_13115 [Geobacillus icigianus] | 219 |
      | lcl|Query\_32843 | KFX32248.1 hypothetical protein EP10\_13120 [Geobacillus icigianus] | 176 |
      | lcl|Query\_32844 | KFX32249.1 AbrB/MazE/SpoVT family DNA-binding domain-containing protein [Geobacillus icigianus] | 93 |
      | lcl|Query\_32845 | KFX32226.1 transposase [Geobacillus icigianus] | 183 |
      | lcl|Query\_32846 | KFX32204.1 hypothetical protein EP10\_13145, partial [Geobacillus icigianus] | 380 |
      | lcl|Query\_32847 | KFX32206.1 FMN-dependent NADH-azoreductase [Geobacillus icigianus] | 211 |
      | lcl|Query\_32848 | KFX32207.1 PAS domain S-box protein [Geobacillus icigianus] | 636 |
      | lcl|Query\_32849 | KFX32208.1 RNA polymerase subunit sigma-70 [Geobacillus icigianus] | 256 |
      | lcl|Query\_32850 | KFX32209.1 anti-sigma B factor RsbW [Geobacillus icigianus] | 162 |
      | lcl|Query\_32851 | KFX32210.1 anti-sigma factor antagonist [Geobacillus icigianus] | 116 |
      | lcl|Query\_32852 | KFX32211.1 DUF1232 domain-containing protein [Geobacillus icigianus] | 127 |
      | lcl|Query\_32853 | KFX32212.1 uracil-DNA glycosylase [Geobacillus icigianus] | 229 |
      | lcl|Query\_32854 | KFX32213.1 hypothetical protein EP10\_13190 [Geobacillus icigianus] | 99 |
      | lcl|Query\_32855 | KFX32214.1 DUF423 domain-containing protein [Geobacillus icigianus] | 123 |
      | lcl|Query\_32856 | KFX32215.1 spore coat protein GerQ [Geobacillus icigianus] | 182 |
      | lcl|Query\_32857 | KFX32216.1 cell wall hydrolase [Geobacillus icigianus] | 142 |
      | lcl|Query\_32858 | KFX32217.1 heme-dependent peroxidase [Geobacillus icigianus] | 248 |
      | lcl|Query\_32859 | KFX32218.1 phosphate acetyltransferase [Geobacillus icigianus] | 326 |
      | lcl|Query\_32860 | KFX32219.1 hypothetical protein EP10\_13220 [Geobacillus icigianus] | 87 |
      | lcl|Query\_32861 | KFX32220.1 lipoate--protein ligase family protein [Geobacillus icigianus] | 289 |
      | lcl|Query\_32862 | KFX32221.1 RsfA family transcriptional regulator [Geobacillus icigianus] | 217 |
      | lcl|Query\_32863 | KFX32222.1 DUF1450 domain-containing protein [Geobacillus icigianus] | 75 |
      | lcl|Query\_32864 | KFX32223.1 HD domain-containing protein [Geobacillus icigianus] | 432 |
      | lcl|Query\_32865 | KFX32224.1 hypothetical protein EP10\_13245 [Geobacillus icigianus] | 172 |
      | lcl|Query\_32866 | KFX32225.1 NCS2 family permease [Geobacillus icigianus] | 433 |
      | lcl|Query\_32867 | KFX32199.1 lactate utilization protein C [Geobacillus icigianus] | 240 |
      | lcl|Query\_32868 | KFX32200.1 iron-sulfur cluster-binding protein [Geobacillus icigianus] | 476 |
      | lcl|Query\_32869 | PUA93231.1 (Fe-S)-binding protein [Geobacillus icigianus] | 265 |
      | lcl|Query\_32870 | KFX32201.2 DNA-binding response regulator [Geobacillus icigianus] | 199 |
      | lcl|Query\_32871 | KFX32184.1 PBP1A family penicillin-binding protein [Geobacillus icigianus] | 900 |
      | lcl|Query\_32872 | KFX32185.1 Holliday junction resolvase RecU [Geobacillus icigianus] | 205 |
      | lcl|Query\_32873 | KFX32186.1 hypothetical protein EP10\_13310 [Geobacillus icigianus] | 80 |
      | lcl|Query\_32874 | KFX32187.1 hypothetical protein EP10\_13320 [Geobacillus icigianus] | 66 |
      | lcl|Query\_32875 | KFX32188.1 spore coat protein [Geobacillus icigianus] | 115 |
      | lcl|Query\_32876 | KFX32189.1 sporulation protein [Geobacillus icigianus] | 84 |
      | lcl|Query\_32877 | KFX32190.1 hypothetical protein EP10\_13335 [Geobacillus icigianus] | 61 |
      | lcl|Query\_32878 | KFX32191.1 heat-shock protein Hsp20 [Geobacillus icigianus] | 154 |
      | lcl|Query\_32879 | KFX32192.2 DNA-binding response regulator [Geobacillus icigianus] | 224 |
      | lcl|Query\_32880 | KFX32193.1 HAMP domain-containing protein [Geobacillus icigianus] | 461 |
      | lcl|Query\_32881 | KFX32194.1 glycerol-3-phosphate responsive antiterminator [Geobacillus icigianus] | 184 |
      | lcl|Query\_32882 | KFX32195.1 glycerol-3-phosphate dehydrogenase/oxidase, partial [Geobacillus icigianus] | 548 |
      | lcl|Query\_32883 | KFX32082.1 glycerol kinase [Geobacillus icigianus] | 496 |
      | lcl|Query\_32884 | KFX32083.1 aquaporin family protein [Geobacillus icigianus] | 272 |
      | lcl|Query\_32885 | KFX32085.1 carbohydrate ABC transporter permease [Geobacillus icigianus] | 270 |
      | lcl|Query\_32886 | PUA93230.1 glycerol-3-phosphate ABC transporter permease, partial [Geobacillus icigianus] | 57 |
      | lcl|Query\_32887 | KFX32084.2 ABC transporter substrate-binding protein [Geobacillus icigianus] | 427 |
      | lcl|Query\_32888 | KFX32078.1 type VII secretion protein EssB [Geobacillus icigianus] | 428 |
      | lcl|Query\_32889 | KFX32080.1 WXG100 family type VII secretion target [Geobacillus icigianus] | 97 |
      | lcl|Query\_32890 | KFX32081.1 hypothetical protein EP10\_13430 [Geobacillus icigianus] | 110 |
      | lcl|Query\_32891 | KFX32079.1 ubiquitin [Geobacillus icigianus] | 79 |
      | lcl|Query\_32892 | KFX32027.1 tRNA (N6-threonylcarbamoyladenosine(37)-N6)-methyltransferase TrmO [Geobacillus icigianus] | 153 |
      | lcl|Query\_32893 | KFX32026.2 hypothetical protein EP10\_13440 [Geobacillus icigianus] | 85 |
      | lcl|Query\_32894 | PUA93229.1 hypothetical protein EP10\_20210, partial [Geobacillus icigianus] | 129 |
      | lcl|Query\_32895 | KFX32022.1 ABC transporter permease [Geobacillus icigianus] | 258 |
      | lcl|Query\_32896 | KFX32024.1 hypothetical protein EP10\_13470 [Geobacillus icigianus] | 321 |
      | lcl|Query\_32897 | KFX32025.1 hypothetical protein EP10\_13475 [Geobacillus icigianus] | 372 |
      | lcl|Query\_32898 | KFX31978.1 Zn-dependent hydrolase, partial [Geobacillus icigianus] | 365 |
      | lcl|Query\_32899 | KFX31979.1 amidohydrolase [Geobacillus icigianus] | 394 |
      | lcl|Query\_32900 | KFX31980.1 DUF3311 domain-containing protein [Geobacillus icigianus] | 66 |
      | lcl|Query\_32901 | KFX31981.1 sodium:solute symporter [Geobacillus icigianus] | 490 |
      | lcl|Query\_32902 | KFX31982.1 hypothetical protein EP10\_13500 [Geobacillus icigianus] | 115 |
      | lcl|Query\_32903 | PUA93227.1 K(+)-transporting ATPase subunit F [Geobacillus icigianus] | 26 |
      | lcl|Query\_32904 | KFX31983.2 potassium-transporting ATPase subunit KdpA [Geobacillus icigianus] | 560 |
      | lcl|Query\_32905 | KFX31984.1 K(+)-transporting ATPase subunit B [Geobacillus icigianus] | 677 |
      | lcl|Query\_32906 | KFX31985.1 potassium-transporting ATPase subunit KdpC [Geobacillus icigianus] | 192 |
      | lcl|Query\_32907 | KFX31986.1 histidine kinase [Geobacillus icigianus] | 374 |
      | lcl|Query\_32908 | KFX31987.1 LacI family transcriptional regulator [Geobacillus icigianus] | 329 |
      | lcl|Query\_32909 | KFX31988.1 ribokinase [Geobacillus icigianus] | 298 |
      | lcl|Query\_32910 | KFX31989.1 D-ribose pyranase [Geobacillus icigianus] | 132 |
      | lcl|Query\_32911 | KFX31990.1 sugar ABC transporter ATP-binding protein [Geobacillus icigianus] | 497 |
      | lcl|Query\_32912 | KFX31991.1 ribose ABC transporter permease [Geobacillus icigianus] | 314 |
      | lcl|Query\_32913 | KFX31992.1 ribose ABC transporter substrate-binding protein RbsB [Geobacillus icigianus] | 311 |
      | lcl|Query\_32914 | KFX31993.1 hypothetical protein EP10\_13560 [Geobacillus icigianus] | 89 |
      | lcl|Query\_32915 | KFX31995.1 arylformamidase [Geobacillus icigianus] | 208 |
      | lcl|Query\_32916 | KFX31996.1 tryptophan 2,3-dioxygenase [Geobacillus icigianus] | 280 |
      | lcl|Query\_32917 | KFX31997.1 amino acid permease [Geobacillus icigianus] | 479 |
      | lcl|Query\_32918 | KFX31998.1 kynureninase [Geobacillus icigianus] | 428 |
      | lcl|Query\_32919 | PUA93228.1 APC family permease [Geobacillus icigianus] | 578 |
      | lcl|Query\_32920 | KFX31916.1 hypothetical protein EP10\_13600, partial [Geobacillus icigianus] | 154 |
      | lcl|Query\_32921 | KFX31917.1 IS701 family transposase, partial [Geobacillus icigianus] | 151 |
      | lcl|Query\_32922 | KFX31911.1 sporulation protein YpjB [Geobacillus icigianus] | 262 |
      | lcl|Query\_32923 | KFX31913.1 cytochrome C oxidase Cbb3 [Geobacillus icigianus] | 254 |
      | lcl|Query\_32924 | KFX31914.1 cytochrome b6 [Geobacillus icigianus] | 224 |
      | lcl|Query\_32925 | KFX31915.1 ubiquinol-cytochrome c reductase iron-sulfur subunit [Geobacillus icigianus] | 169 |
      | lcl|Query\_32926 | PUA93226.1 DUF2487 domain-containing protein, partial [Geobacillus icigianus] | 133 |
      | lcl|Query\_32927 | KFX31912.1 DUF1405 domain-containing protein [Geobacillus icigianus] | 201 |
      | lcl|Query\_32928 | KFX31842.1 restriction endonuclease, partial [Geobacillus icigianus] | 141 |
      | lcl|Query\_32929 | KFX31843.2 DNA (cytosine-5-)-methyltransferase [Geobacillus icigianus] | 364 |
      | lcl|Query\_32930 | KFX31844.1 ABC transporter ATP-binding protein [Geobacillus icigianus] | 672 |
      | lcl|Query\_32931 | KFX31845.1 multidrug ABC transporter permease/ATP-binding protein [Geobacillus icigianus] | 586 |
      | lcl|Query\_32932 | KFX31846.1 Uma2 family endonuclease [Geobacillus icigianus] | 190 |
      | lcl|Query\_32933 | KFX31847.1 NUDIX domain-containing protein [Geobacillus icigianus] | 140 |
      | lcl|Query\_32934 | KFX31848.1 ABC transporter ATP-binding protein [Geobacillus icigianus] | 246 |
      | lcl|Query\_32935 | KFX31849.1 ABC transporter permease [Geobacillus icigianus] | 256 |
      | lcl|Query\_32936 | KFX31850.1 NUDIX domain-containing protein [Geobacillus icigianus] | 141 |
      | lcl|Query\_32937 | KFX31851.1 catalase/peroxidase HPI [Geobacillus icigianus] | 736 |
      | lcl|Query\_32938 | KFX31811.1 amino acid transporter [Geobacillus icigianus] | 205 |
      | lcl|Query\_32939 | KFX31812.1 PBP1A family penicillin-binding protein [Geobacillus icigianus] | 681 |
      | lcl|Query\_32940 | KFX31813.1 polyamine aminopropyltransferase [Geobacillus icigianus] | 275 |
      | lcl|Query\_32941 | KFX31814.1 agmatinase [Geobacillus icigianus] | 291 |
      | lcl|Query\_32942 | KFX31815.1 DUF1934 domain-containing protein [Geobacillus icigianus] | 143 |
      | lcl|Query\_32943 | KFX31816.1 arginine--tRNA ligase [Geobacillus icigianus] | 557 |
      | lcl|Query\_32944 | KFX31817.1 XapX domain-containing protein [Geobacillus icigianus] | 56 |
      | lcl|Query\_32945 | KFX31818.1 cardiolipin synthase [Geobacillus icigianus] | 400 |
      | lcl|Query\_32946 | KFX31819.1 (Fe-S)-binding protein [Geobacillus icigianus] | 698 |
      | lcl|Query\_32947 | KFX31820.1 acetyl-CoA C-acetyltransferase [Geobacillus icigianus] | 392 |
      | lcl|Query\_32948 | KFX31821.1 hypothetical protein EP10\_13760 [Geobacillus icigianus] | 86 |
      | lcl|Query\_32949 | KFX31822.1 3-hydroxybutyryl-CoA dehydrogenase [Geobacillus icigianus] | 283 |
      | lcl|Query\_32950 | KFX31823.1 acyl-CoA dehydrogenase [Geobacillus icigianus] | 380 |
      | lcl|Query\_32951 | KFX31824.1 acyl-CoA dehydrogenase [Geobacillus icigianus] | 380 |
      | lcl|Query\_32952 | KFX31825.1 TetR/AcrR family transcriptional regulator [Geobacillus icigianus] | 210 |
      | lcl|Query\_32953 | KFX31826.1 methylmalonyl-CoA mutase [Geobacillus icigianus] | 1086 |
      | lcl|Query\_32954 | KFX31827.1 hypothetical protein EP10\_13790 [Geobacillus icigianus] | 65 |
      | lcl|Query\_32955 | KFX31828.1 DNA-directed RNA polymerase subunit delta [Geobacillus icigianus] | 185 |
      | lcl|Query\_32956 | KFX31829.1 CTP synthase [Geobacillus icigianus] | 531 |
      | lcl|Query\_32957 | KFX31830.1 DUF2529 domain-containing protein [Geobacillus icigianus] | 173 |
      | lcl|Query\_32958 | KFX31831.1 two-component system response regulator [Geobacillus icigianus] | 120 |
      | lcl|Query\_32959 | KFX31832.1 fructose-1,6-bisphosphate aldolase, class II [Geobacillus icigianus] | 287 |
      | lcl|Query\_32960 | PUA93225.1 SMI1/KNR4 family protein, partial [Geobacillus icigianus] | 145 |
      | lcl|Query\_32961 | KFX31806.2 hypothetical protein EP10\_13830 [Geobacillus icigianus] | 168 |
      | lcl|Query\_32962 | KFX31778.1 small acid-soluble spore protein [Geobacillus icigianus] | 67 |
      | lcl|Query\_32963 | KFX31781.1 hypothetical protein EP10\_13850 [Geobacillus icigianus] | 74 |
      | lcl|Query\_32964 | KFX31782.1 septation ring formation regulator EzrA [Geobacillus icigianus] | 567 |
      | lcl|Query\_32965 | KFX31783.1 histidinol-phosphatase [Geobacillus icigianus] | 279 |
      | lcl|Query\_32966 | KFX31784.1 GAF domain-containing protein [Geobacillus icigianus] | 158 |
      | lcl|Query\_32967 | KFX31785.1 GGDEF domain-containing protein [Geobacillus icigianus] | 610 |
      | lcl|Query\_32968 | PUA93220.1 hypothetical protein EP10\_20260 [Geobacillus icigianus] | 97 |
      | lcl|Query\_32969 | KFX31786.1 30S ribosomal protein S4 [Geobacillus icigianus] | 200 |
      | lcl|Query\_32970 | KFX31787.1 tyrosine--tRNA ligase [Geobacillus icigianus] | 419 |
      | lcl|Query\_32971 | PUA93221.1 peptidoglycan glycosyltransferase [Geobacillus icigianus] | 923 |
      | lcl|Query\_32972 | KFX31788.1 acetate--CoA ligase [Geobacillus icigianus] | 571 |
      | lcl|Query\_32973 | KFX31789.1 N-acetyltransferase [Geobacillus icigianus] | 210 |
      | lcl|Query\_32974 | KFX31790.1 CBS domain-containing protein [Geobacillus icigianus] | 214 |
      | lcl|Query\_32975 | KFX31791.1 acetoin utilization protein AcuC [Geobacillus icigianus] | 389 |
      | lcl|Query\_32976 | PUA93222.1 hypothetical protein EP10\_20275 [Geobacillus icigianus] | 100 |
      | lcl|Query\_32977 | KFX31792.1 catabolite control protein A [Geobacillus icigianus] | 330 |
      | lcl|Query\_32978 | KFX31793.1 3-deoxy-7-phosphoheptulonate synthase [Geobacillus icigianus] | 360 |
      | lcl|Query\_32979 | KFX31794.1 bacillithiol system redox-active protein YtxJ [Geobacillus icigianus] | 109 |
      | lcl|Query\_32980 | PUA93223.1 hypothetical protein EP10\_20280 [Geobacillus icigianus] | 211 |
      | lcl|Query\_32981 | KFX31795.1 DUF948 domain-containing protein [Geobacillus icigianus] | 148 |
      | lcl|Query\_32982 | KFX31796.1 UDP-N-acetylmuramate--L-alanine ligase [Geobacillus icigianus] | 434 |
      | lcl|Query\_32983 | KFX31797.1 DNA translocase FtsK [Geobacillus icigianus] | 853 |
      | lcl|Query\_32984 | KFX31798.1 DUF4479 domain-containing protein [Geobacillus icigianus] | 201 |
      | lcl|Query\_32985 | KFX31799.1 DUF1444 domain-containing protein [Geobacillus icigianus] | 265 |
      | lcl|Query\_32986 | KFX31800.1 DUF84 domain-containing protein [Geobacillus icigianus] | 176 |
      | lcl|Query\_32987 | KFX31801.1 M42 family peptidase [Geobacillus icigianus] | 358 |
      | lcl|Query\_32988 | KFX31802.1 peptidase M4 [Geobacillus icigianus] | 98 |
      | lcl|Query\_32989 | KFX31803.1 MBL fold metallo-hydrolase [Geobacillus icigianus] | 283 |
      | lcl|Query\_32990 | KFX31804.1 tRNA (guanosine(46)-N7)-methyltransferase TrmB [Geobacillus icigianus] | 216 |
      | lcl|Query\_32991 | PUA93224.1 hypothetical protein EP10\_20285, partial [Geobacillus icigianus] | 53 |
      | lcl|Query\_32992 | KFX31779.1 tRNA 4-thiouridine(8) synthase ThiI [Geobacillus icigianus] | 401 |
      | lcl|Query\_32993 | KFX31780.1 cysteine desulfurase [Geobacillus icigianus] | 387 |
      | lcl|Query\_32994 | KFX31756.1 sporulation transcription factor Spo0A [Geobacillus icigianus] | 266 |
      | lcl|Query\_32995 | KFX31744.1 MFS transporter, partial [Geobacillus icigianus] | 311 |
      | lcl|Query\_32996 | KFX31745.2 acyl-CoA dehydrogenase [Geobacillus icigianus] | 366 |
      | lcl|Query\_32997 | PUA93219.1 hypothetical protein EP10\_20290 [Geobacillus icigianus] | 159 |
      | lcl|Query\_32998 | KFX31746.1 aspartate aminotransferase family protein [Geobacillus icigianus] | 418 |
      | lcl|Query\_32999 | KFX31739.1 IS66 family transposase, partial [Geobacillus icigianus] | 334 |
      | lcl|Query\_33000 | KFX31740.2 metallothiol transferase FosB [Geobacillus icigianus] | 153 |
      | lcl|Query\_33001 | KFX31721.1 barnase inhibitor [Geobacillus icigianus] | 101 |
      | lcl|Query\_33002 | KFX31724.2 hypothetical protein EP10\_14085 [Geobacillus icigianus] | 101 |
      | lcl|Query\_33003 | KFX31725.1 DUF2564 domain-containing protein [Geobacillus icigianus] | 89 |
      | lcl|Query\_33004 | KFX31726.1 cold-shock protein CspB [Geobacillus icigianus] | 66 |
      | lcl|Query\_33005 | KFX31727.1 hypothetical protein EP10\_14100 [Geobacillus icigianus] | 83 |
      | lcl|Query\_33006 | KFX31728.1 sporulation protein SpoOM [Geobacillus icigianus] | 133 |
      | lcl|Query\_33007 | KFX31587.1 SDR family NAD(P)-dependent oxidoreductase, partial [Geobacillus icigianus] | 248 |
      | lcl|Query\_33008 | KFX31588.1 NAD(P)-dependent oxidoreductase [Geobacillus icigianus] | 288 |
      | lcl|Query\_33009 | KFX31589.1 acyl-CoA dehydrogenase [Geobacillus icigianus] | 403 |
      | lcl|Query\_33010 | KFX31591.1 TetR/AcrR family transcriptional regulator [Geobacillus icigianus] | 189 |
      | lcl|Query\_33011 | KFX31592.1 phosphotransferase family protein [Geobacillus icigianus] | 352 |
      | lcl|Query\_33012 | KFX31593.1 2-phosphosulfolactate phosphatase [Geobacillus icigianus] | 260 |
      | lcl|Query\_33013 | KFX31594.1 NADPH:quinone oxidoreductase family protein [Geobacillus icigianus] | 324 |
      | lcl|Query\_33014 | KFX31595.1 aminotransferase A [Geobacillus icigianus] | 385 |
      | lcl|Query\_33015 | KFX31596.1 IDEAL domain-containing protein [Geobacillus icigianus] | 75 |
      | lcl|Query\_33016 | KFX31597.1 magnesium and cobalt transport protein CorA [Geobacillus icigianus] | 332 |
      | lcl|Query\_33017 | KFX31598.1 DUF1906 domain-containing protein [Geobacillus icigianus] | 216 |
      | lcl|Query\_33018 | KFX31599.1 GGDEF domain-containing protein [Geobacillus icigianus] | 543 |
      | lcl|Query\_33019 | KFX31600.1 HEAT repeat domain-containing protein [Geobacillus icigianus] | 341 |
      | lcl|Query\_33020 | KFX31602.1 hypothetical protein EP10\_14195 [Geobacillus icigianus] | 144 |
      | lcl|Query\_33021 | KFX31603.1 hypothetical protein EP10\_14200 [Geobacillus icigianus] | 94 |
      | lcl|Query\_33022 | KFX31604.1 2,4-dienoyl-CoA reductase [Geobacillus icigianus] | 255 |
      | lcl|Query\_33023 | KFX31605.1 diguanylate phosphodiesterase [Geobacillus icigianus] | 405 |
      | lcl|Query\_33024 | KFX31606.1 hypothetical protein EP10\_14220 [Geobacillus icigianus] | 77 |
      | lcl|Query\_33025 | KFX31607.1 antirepressor AbbA [Geobacillus icigianus] | 65 |
      | lcl|Query\_33026 | KFX31608.1 CBS domain-containing protein [Geobacillus icigianus] | 158 |
      | lcl|Query\_33027 | KFX31609.1 LysR family transcriptional regulator [Geobacillus icigianus] | 290 |
      | lcl|Query\_33028 | KFX31610.1 2,3,4,5-tetrahydropyridine-2,6-dicarboxylate N-acetyltransferase [Geobacillus icigianus] | 236 |
      | lcl|Query\_33029 | KFX31611.1 N-acetyldiaminopimelate deacetylase [Geobacillus icigianus] | 378 |
      | lcl|Query\_33030 | KFX31612.1 hypothetical protein EP10\_14255 [Geobacillus icigianus] | 80 |
      | lcl|Query\_33031 | KFX31613.1 TrkA family potassium uptake protein [Geobacillus icigianus] | 220 |
      | lcl|Query\_33032 | KFX31614.1 ribonuclease J [Geobacillus icigianus] | 555 |
      | lcl|Query\_33033 | KFX31615.1 hypothetical protein EP10\_14270 [Geobacillus icigianus] | 71 |
      | lcl|Query\_33034 | KFX31616.1 Cof-type HAD-IIB family hydrolase [Geobacillus icigianus] | 258 |
      | lcl|Query\_33035 | KFX31617.1 peptide deformylase [Geobacillus icigianus] | 184 |
      | lcl|Query\_33036 | KFX31618.1 pyruvate dehydrogenase (acetyl-transferring) E1 component subunit alpha [Geobacillus icigianus] | 369 |
      | lcl|Query\_33037 | KFX31619.1 alpha-ketoacid dehydrogenase subunit beta [Geobacillus icigianus] | 325 |
      | lcl|Query\_33038 | KFX31620.1 2-oxo acid dehydrogenase subunit E2 [Geobacillus icigianus] | 431 |
      | lcl|Query\_33039 | KFX31621.1 dihydrolipoyl dehydrogenase [Geobacillus icigianus] | 470 |
      | lcl|Query\_33040 | KFX31622.1 hypothetical protein EP10\_14315 [Geobacillus icigianus] | 80 |
      | lcl|Query\_33041 | KFX31623.1 DUF1885 domain-containing protein [Geobacillus icigianus] | 125 |
      | lcl|Query\_33042 | KFX31624.1 GapA-binding peptide SR1P [Geobacillus icigianus] | 41 |
      | lcl|Query\_33043 | KFX31625.1 aminotransferase class V-fold PLP-dependent enzyme [Geobacillus icigianus] | 490 |
      | lcl|Query\_33044 | KFX31626.1 nitronate monooxygenase [Geobacillus icigianus] | 319 |
      | lcl|Query\_33045 | KFX31627.1 hypothetical protein EP10\_14345 [Geobacillus icigianus] | 98 |
      | lcl|Query\_33046 | KFX31629.1 inositol monophosphatase family protein [Geobacillus icigianus] | 264 |
      | lcl|Query\_33047 | KFX31630.2 translational GTPase TypA [Geobacillus icigianus] | 609 |
      | lcl|Query\_33048 | KFX31631.1 hypothetical protein EP10\_14375 [Geobacillus icigianus] | 102 |
      | lcl|Query\_33049 | KFX31632.1 hypothetical protein EP10\_14380 [Geobacillus icigianus] | 94 |
      | lcl|Query\_33050 | KFX31633.1 DUF2197 domain-containing protein [Geobacillus icigianus] | 66 |
      | lcl|Query\_33051 | KFX31634.1 YhcN/YlaJ family sporulation lipoprotein [Geobacillus icigianus] | 196 |
      | lcl|Query\_33052 | KFX31635.1 PhoH family protein [Geobacillus icigianus] | 443 |
      | lcl|Query\_33053 | KFX31636.1 hypothetical protein EP10\_14400 [Geobacillus icigianus] | 168 |
      | lcl|Query\_33054 | KFX31638.1 putative lipid II flippase FtsW [Geobacillus icigianus] | 403 |
      | lcl|Query\_33055 | KFX31639.1 pyruvate carboxylase [Geobacillus icigianus] | 1147 |
      | lcl|Query\_33056 | KFX31640.2 heme A synthase [Geobacillus icigianus] | 331 |
      | lcl|Query\_33057 | KFX31641.1 protoheme IX farnesyltransferase [Geobacillus icigianus] | 309 |
      | lcl|Query\_33058 | KFX31642.1 cytochrome c oxidase subunit II [Geobacillus icigianus] | 356 |
      | lcl|Query\_33059 | KFX31643.1 cytochrome c oxidase subunit I [Geobacillus icigianus] | 623 |
      | lcl|Query\_33060 | KFX31644.1 cytochrome (ubi)quinol oxidase subunit III [Geobacillus icigianus] | 206 |
      | lcl|Query\_33061 | KFX31645.1 cytochrome c oxidase subunit 4B [Geobacillus icigianus] | 110 |
      | lcl|Query\_33062 | KFX31646.1 cytochrome c oxidase assembly factor CtaG [Geobacillus icigianus] | 299 |
      | lcl|Query\_33063 | KFX31647.1 DUF420 domain-containing protein [Geobacillus icigianus] | 151 |
      | lcl|Query\_33064 | KFX31648.1 hypothetical protein EP10\_14460 [Geobacillus icigianus] | 97 |
      | lcl|Query\_33065 | KFX31649.1 hypothetical protein EP10\_14465 [Geobacillus icigianus] | 119 |
      | lcl|Query\_33066 | KFX31650.1 CBS domain-containing protein [Geobacillus icigianus] | 141 |
      | lcl|Query\_33067 | KFX31651.1 hypothetical protein EP10\_14480 [Geobacillus icigianus] | 335 |
      | lcl|Query\_33068 | KFX31652.1 cytosolic protein [Geobacillus icigianus] | 127 |
      | lcl|Query\_33069 | KFX31656.1 hypothetical protein EP10\_14505 [Geobacillus icigianus] | 89 |
      | lcl|Query\_33070 | KFX31657.1 hypothetical protein EP10\_14510 [Geobacillus icigianus] | 72 |
      | lcl|Query\_33071 | KFX31658.1 methylthioribose kinase [Geobacillus icigianus] | 129 |
      | lcl|Query\_33072 | KFX31659.1 hypothetical protein EP10\_14520 [Geobacillus icigianus] | 62 |
      | lcl|Query\_33073 | KFX31660.1 16S rRNA (guanine(966)-N(2))-methyltransferase RsmD [Geobacillus icigianus] | 198 |
      | lcl|Query\_33074 | KFX31661.1 pantetheine-phosphate adenylyltransferase [Geobacillus icigianus] | 166 |
      | lcl|Query\_33075 | KFX31662.1 sporulation integral membrane protein YlbJ [Geobacillus icigianus] | 410 |
      | lcl|Query\_33076 | KFX31663.1 esterase [Geobacillus icigianus] | 262 |
      | lcl|Query\_33077 | KFX31664.1 PDZ domain-containing protein [Geobacillus icigianus] | 339 |
      | lcl|Query\_33078 | KFX31665.1 nucleotidyltransferase [Geobacillus icigianus] | 406 |
      | lcl|Query\_33079 | KFX31666.1 hypothetical protein EP10\_14555 [Geobacillus icigianus] | 180 |
      | lcl|Query\_33080 | PUA93217.1 50S ribosomal protein L32 [Geobacillus icigianus] | 57 |
      | lcl|Query\_33081 | KFX31668.1 enoyl-CoA hydratase/isomerase family protein [Geobacillus icigianus] | 251 |
      | lcl|Query\_33082 | KFX31669.1 RsfA family transcriptional regulator [Geobacillus icigianus] | 191 |
      | lcl|Query\_33083 | KFX31670.2 2-dehydropantoate 2-reductase [Geobacillus icigianus] | 306 |
      | lcl|Query\_33084 | KFX31671.1 DUF3397 domain-containing protein [Geobacillus icigianus] | 128 |
      | lcl|Query\_33085 | KFX31672.1 bacillithiol biosynthesis cysteine-adding enzyme BshC [Geobacillus icigianus] | 542 |
      | lcl|Query\_33086 | KFX31673.1 16S rRNA (cytosine(1402)-N(4))-methyltransferase RsmH [Geobacillus icigianus] | 310 |
      | lcl|Query\_33087 | KFX31675.1 PASTA domain-containing protein [Geobacillus icigianus] | 735 |
      | lcl|Query\_33088 | KFX31676.1 stage V sporulation protein D [Geobacillus icigianus] | 639 |
      | lcl|Query\_33089 | KFX31678.1 hypothetical protein EP10\_14615 [Geobacillus icigianus] | 151 |
      | lcl|Query\_33090 | KFX31679.1 phospho-N-acetylmuramoyl-pentapeptide-transferase [Geobacillus icigianus] | 324 |
      | lcl|Query\_33091 | KFX31680.1 UDP-N-acetylmuramoyl-L-alanine--D-glutamate ligase [Geobacillus icigianus] | 451 |
      | lcl|Query\_33092 | KFX31681.1 stage V sporulation protein E [Geobacillus icigianus] | 366 |
      | lcl|Query\_33093 | KFX31682.1 cell division protein FtsQ/DivIB [Geobacillus icigianus] | 261 |
      | lcl|Query\_33094 | KFX31683.1 DUF881 domain-containing protein [Geobacillus icigianus] | 234 |
      | lcl|Query\_33095 | KFX31685.1 DUF1290 domain-containing protein [Geobacillus icigianus] | 120 |
      | lcl|Query\_33096 | KFX31687.1 cell division protein FtsZ [Geobacillus icigianus] | 375 |
      | lcl|Query\_33097 | KFX31688.1 hypothetical protein EP10\_14675 [Geobacillus icigianus] | 86 |
      | lcl|Query\_33098 | KFX31689.1 sigma-E processing peptidase SpoIIGA [Geobacillus icigianus] | 304 |
      | lcl|Query\_33099 | KFX31690.1 RNA polymerase sporulation sigma factor SigE [Geobacillus icigianus] | 239 |
      | lcl|Query\_33100 | KFX31692.1 YlmC/YmxH family sporulation protein [Geobacillus icigianus] | 82 |
      | lcl|Query\_33101 | KFX31693.1 hypothetical protein EP10\_14700 [Geobacillus icigianus] | 68 |
      | lcl|Query\_33102 | KFX31695.1 YggS family pyridoxal phosphate-dependent enzyme [Geobacillus icigianus] | 224 |
      | lcl|Query\_33103 | KFX31696.1 cell division protein SepF [Geobacillus icigianus] | 143 |
      | lcl|Query\_33104 | KFX31697.1 YggT family protein [Geobacillus icigianus] | 90 |
      | lcl|Query\_33105 | KFX31698.1 RNA-binding protein [Geobacillus icigianus] | 257 |
      | lcl|Query\_33106 | KFX31699.1 DivIVA domain-containing protein [Geobacillus icigianus] | 177 |
      | lcl|Query\_33107 | KFX31700.1 isoleucine--tRNA ligase [Geobacillus icigianus] | 924 |
      | lcl|Query\_33108 | KFX31701.1 hypothetical protein EP10\_14740 [Geobacillus icigianus] | 622 |
      | lcl|Query\_33109 | KFX31702.1 ABC transporter permease [Geobacillus icigianus] | 683 |
      | lcl|Query\_33110 | KFX31703.1 ABC transporter ATP-binding protein [Geobacillus icigianus] | 289 |
      | lcl|Query\_33111 | KFX31704.1 hypothetical protein EP10\_14755 [Geobacillus icigianus] | 219 |
      | lcl|Query\_33112 | KFX31705.1 ABC transporter permease [Geobacillus icigianus] | 672 |
      | lcl|Query\_33113 | KFX31706.1 ABC transporter ATP-binding protein [Geobacillus icigianus] | 288 |
      | lcl|Query\_33114 | KFX31707.1 hypothetical protein EP10\_14770 [Geobacillus icigianus] | 210 |
      | lcl|Query\_33115 | KFX31708.2 lipoprotein signal peptidase [Geobacillus icigianus] | 163 |
      | lcl|Query\_33116 | KFX31709.1 RluA family pseudouridine synthase [Geobacillus icigianus] | 304 |
      | lcl|Query\_33117 | KFX31710.1 bifunctional pyr operon transcriptional regulator/uracil phosphoribosyltransferase PyrR [Geobacillus icigianus] | 179 |
      | lcl|Query\_33118 | KFX31711.1 uracil permease [Geobacillus icigianus] | 432 |
      | lcl|Query\_33119 | KFX31712.1 aspartate carbamoyltransferase catalytic subunit [Geobacillus icigianus] | 308 |
      | lcl|Query\_33120 | KFX31713.1 dihydroorotase [Geobacillus icigianus] | 427 |
      | lcl|Query\_33121 | KFX31714.1 carbamoyl-phosphate synthase small subunit [Geobacillus icigianus] | 364 |
      | lcl|Query\_33122 | KFX31715.1 carbamoyl-phosphate synthase large subunit [Geobacillus icigianus] | 1065 |
      | lcl|Query\_33123 | KFX31716.1 dihydroorotate dehydrogenase electron transfer subunit [Geobacillus icigianus] | 257 |
      | lcl|Query\_33124 | KFX31717.1 dihydroorotate dehydrogenase [Geobacillus icigianus] | 313 |
      | lcl|Query\_33125 | KFX31718.1 orotidine-5'-phosphate decarboxylase [Geobacillus icigianus] | 239 |
      | lcl|Query\_33126 | KFX31719.1 orotate phosphoribosyltransferase [Geobacillus icigianus] | 207 |
      | lcl|Query\_33127 | KFX31720.1 DUF3888 domain-containing protein [Geobacillus icigianus] | 142 |
      | lcl|Query\_33128 | PUA93218.1 integrase [Geobacillus icigianus] | 47 |
      | lcl|Query\_33129 | KFX31590.1 3-oxoacyl-ACP reductase [Geobacillus icigianus] | 260 |
      | lcl|Query\_33130 | KFX31601.1 glycosyltransferase family 2 protein [Geobacillus icigianus] | 467 |
      | lcl|Query\_33131 | KFX31628.1 hypothetical protein EP10\_14350 [Geobacillus icigianus] | 62 |
      | lcl|Query\_33132 | KFX31637.1 hypothetical protein EP10\_14405 [Geobacillus icigianus] | 95 |
      | lcl|Query\_33133 | KFX31653.1 hypothetical protein EP10\_14490 [Geobacillus icigianus] | 77 |
      | lcl|Query\_33134 | KFX31655.1 YlbF family regulator [Geobacillus icigianus] | 148 |
      | lcl|Query\_33135 | KFX31674.1 cell division protein FtsL [Geobacillus icigianus] | 118 |
      | lcl|Query\_33136 | KFX31677.1 UDP-N-acetylmuramoyl-L-alanyl-D-glutamate--2,6-diaminopimelate ligase [Geobacillus icigianus] | 489 |
      | lcl|Query\_33137 | KFX31684.2 DUF881 domain-containing protein [Geobacillus icigianus] | 219 |
      | lcl|Query\_33138 | KFX31686.1 cell division protein FtsA [Geobacillus icigianus] | 422 |
      | lcl|Query\_33139 | KFX31691.1 RNA polymerase sporulation sigma factor SigG [Geobacillus icigianus] | 259 |
      | lcl|Query\_33140 | KFX31694.1 peptidoglycan editing factor PgeF [Geobacillus icigianus] | 272 |
      | lcl|Query\_33141 | KFX31533.1 lysine--tRNA ligase [Geobacillus icigianus] | 494 |
      | lcl|Query\_33142 | KFX31534.1 tRNA dihydrouridine synthase DusB [Geobacillus icigianus] | 333 |
      | lcl|Query\_33143 | KFX31535.1 XRE family transcriptional regulator [Geobacillus icigianus] | 73 |
      | lcl|Query\_33144 | KFX31536.1 2-amino-4-hydroxy-6-hydroxymethyldihydropteridine diphosphokinase [Geobacillus icigianus] | 175 |
      | lcl|Query\_33145 | KFX31537.2 dihydroneopterin aldolase [Geobacillus icigianus] | 129 |
      | lcl|Query\_33146 | KFX31538.1 dihydropteroate synthase [Geobacillus icigianus] | 283 |
      | lcl|Query\_33147 | KFX31539.1 4-amino-4-deoxychorismate lyase [Geobacillus icigianus] | 291 |
      | lcl|Query\_33148 | KFX31540.1 aminodeoxychorismate/anthranilate synthase component II [Geobacillus icigianus] | 191 |
      | lcl|Query\_33149 | KFX31541.1 aminodeoxychorismate synthase component I [Geobacillus icigianus] | 473 |
      | lcl|Query\_33150 | KFX31542.1 cysteine synthase A [Geobacillus icigianus] | 308 |
      | lcl|Query\_33151 | KFX31544.1 type III pantothenate kinase [Geobacillus icigianus] | 258 |
      | lcl|Query\_33152 | KFX31545.1 ATP-dependent metallopeptidase FtsH/Yme1/Tma family protein [Geobacillus icigianus] | 628 |
      | lcl|Query\_33153 | KFX31546.1 hypoxanthine phosphoribosyltransferase [Geobacillus icigianus] | 181 |
      | lcl|Query\_33154 | KFX31547.1 tRNA lysidine(34) synthetase TilS [Geobacillus icigianus] | 464 |
      | lcl|Query\_33155 | KFX31548.1 serine/threonine protein kinase [Geobacillus icigianus] | 325 |
      | lcl|Query\_33156 | KFX31549.1 VWA domain-containing protein [Geobacillus icigianus] | 246 |
      | lcl|Query\_33157 | KFX31550.1 stage II sporulation protein E [Geobacillus icigianus] | 825 |
      | lcl|Query\_33158 | KFX31551.1 RNA-binding protein S1 [Geobacillus icigianus] | 132 |
      | lcl|Query\_33159 | KFX31552.1 septum formation initiator family protein [Geobacillus icigianus] | 123 |
      | lcl|Query\_33160 | KFX31553.1 spore cortex biosynthesis protein YabQ [Geobacillus icigianus] | 210 |
      | lcl|Query\_33161 | KFX31555.1 RNA-binding S4 domain-containing protein [Geobacillus icigianus] | 93 |
      | lcl|Query\_33162 | KFX31557.1 polysaccharide biosynthesis protein [Geobacillus icigianus] | 516 |
      | lcl|Query\_33163 | KFX31559.1 transcription-repair coupling factor [Geobacillus icigianus] | 1177 |
      | lcl|Query\_33164 | KFX31560.1 DUF2757 domain-containing protein [Geobacillus icigianus] | 76 |
      | lcl|Query\_33165 | KFX31561.1 aminoacyl-tRNA hydrolase [Geobacillus icigianus] | 186 |
      | lcl|Query\_33166 | KFX31562.1 50S ribosomal protein L25 [Geobacillus icigianus] | 210 |
      | lcl|Query\_33167 | KFX31563.1 ribose-phosphate diphosphokinase [Geobacillus icigianus] | 315 |
      | lcl|Query\_33168 | KFX31564.1 bifunctional UDP-N-acetylglucosamine diphosphorylase/glucosamine-1-phosphate N-acetyltransferase GlmU [Geobacillus icigianus] | 458 |
      | lcl|Query\_33169 | KFX31565.1 septation protein spoVG [Geobacillus icigianus] | 96 |
      | lcl|Query\_33170 | KFX31566.1 RidA family protein [Geobacillus icigianus] | 124 |
      | lcl|Query\_33171 | KFX31567.1 pur operon repressor [Geobacillus icigianus] | 273 |
      | lcl|Query\_33172 | KFX31568.1 4-(cytidine 5'-diphospho)-2-C-methyl-D-erythritol kinase [Geobacillus icigianus] | 290 |
      | lcl|Query\_33173 | KFX31569.1 small, acid-soluble spore protein, alpha/beta type [Geobacillus icigianus] | 52 |
      | lcl|Query\_33174 | KFX31570.1 ABC transporter permease [Geobacillus icigianus] | 85 |
      | lcl|Query\_33175 | KFX31571.1 sporulation peptidase YabG [Geobacillus icigianus] | 299 |
      | lcl|Query\_33176 | KFX31572.1 16S rRNA (adenine(1518)-N(6)/adenine(1519)-N(6))-dimethyltransferase RsmA [Geobacillus icigianus] | 292 |
      | lcl|Query\_33177 | KFX31574.1 DUF348 domain-containing protein [Geobacillus icigianus] | 403 |
      | lcl|Query\_33178 | KFX31575.1 TatD family deoxyribonuclease [Geobacillus icigianus] | 256 |
      | lcl|Query\_33179 | KFX31576.1 methionine--tRNA ligase [Geobacillus icigianus] | 650 |
      | lcl|Query\_33180 | KFX31543.2 Hsp33 family molecular chaperone HslO [Geobacillus icigianus] | 296 |
      | lcl|Query\_33181 | KFX31554.1 sporulation protein YabP [Geobacillus icigianus] | 101 |
      | lcl|Query\_33182 | KFX31556.1 MazG family protein [Geobacillus icigianus] | 489 |
      | lcl|Query\_33183 | KFX31558.1 stage V sporulation protein T [Geobacillus icigianus] | 178 |
      | lcl|Query\_33184 | KFX31573.1 ribonuclease M5 [Geobacillus icigianus] | 187 |
      | lcl|Query\_33185 | KFX31506.1 IS4 family transposase, partial [Geobacillus icigianus] | 83 |
      | lcl|Query\_33186 | KFX31507.1 zinc-finger domain-containing protein [Geobacillus icigianus] | 71 |
      | lcl|Query\_33187 | KFX31508.1 hypothetical protein EP10\_15175 [Geobacillus icigianus] | 221 |
      | lcl|Query\_33188 | KFX31509.1 EamA/RhaT family transporter [Geobacillus icigianus] | 308 |
      | lcl|Query\_33189 | KFX31510.1 small, acid-soluble spore protein L [Geobacillus icigianus] | 45 |
      | lcl|Query\_33190 | KFX31511.1 aconitate hydratase AcnA [Geobacillus icigianus] | 906 |
      | lcl|Query\_33191 | KFX31512.1 small acid-soluble spore protein O [Geobacillus icigianus] | 49 |
      | lcl|Query\_33192 | KFX31513.1 hypothetical protein EP10\_15200 [Geobacillus icigianus] | 86 |
      | lcl|Query\_33193 | KFX31514.1 small, acid-soluble spore protein P [Geobacillus icigianus] | 48 |
      | lcl|Query\_33194 | KFX31483.1 two-component sensor histidine kinase, partial [Geobacillus icigianus] | 437 |
      | lcl|Query\_33195 | KFX31477.1 IS66 family transposase [Geobacillus icigianus] | 485 |
      | lcl|Query\_33196 | KFX31458.1 putative CRISPR-associated protein [Geobacillus icigianus] | 400 |
      | lcl|Query\_33197 | KFX31459.1 TIGR02221 family CRISPR-associated protein [Geobacillus icigianus] | 441 |
      | lcl|Query\_33198 | KFX31460.2 type III-B CRISPR module RAMP protein Cmr1 [Geobacillus icigianus] | 302 |
      | lcl|Query\_33199 | KFX31461.1 type III-B CRISPR-associated protein Cas10/Cmr2 [Geobacillus icigianus] | 547 |
      | lcl|Query\_33200 | KFX31462.1 type III-B CRISPR module-associated protein Cmr3 [Geobacillus icigianus] | 376 |
      | lcl|Query\_33201 | KFX31463.1 type III-B CRISPR module RAMP protein Cmr4 [Geobacillus icigianus] | 297 |
      | lcl|Query\_33202 | KFX31464.1 type III-B CRISPR module-associated protein Cmr5 [Geobacillus icigianus] | 136 |
      | lcl|Query\_33203 | KFX31465.1 type III-B CRISPR module RAMP protein Cmr6 [Geobacillus icigianus] | 381 |
      | lcl|Query\_33204 | KFX31466.1 hypothetical protein EP10\_15265 [Geobacillus icigianus] | 72 |
      | lcl|Query\_33205 | KFX31467.1 TIGR02556 family CRISPR-associated protein [Geobacillus icigianus] | 571 |
      | lcl|Query\_33206 | KFX31468.1 type I-B CRISPR-associated protein Cas7/Csh2 [Geobacillus icigianus] | 318 |
      | lcl|Query\_33207 | KFX31469.1 type I-B CRISPR-associated protein Cas5 [Geobacillus icigianus] | 247 |
      | lcl|Query\_33208 | KFX31470.1 CRISPR-associated helicase/endonuclease Cas3 [Geobacillus icigianus] | 777 |
      | lcl|Query\_33209 | KFX31471.1 CRISPR-associated protein Cas4 [Geobacillus icigianus] | 169 |
      | lcl|Query\_33210 | KFX31472.1 type I-B CRISPR-associated endonuclease Cas1 [Geobacillus icigianus] | 333 |
      | lcl|Query\_33211 | KFX31473.1 CRISPR-associated endonuclease Cas2 [Geobacillus icigianus] | 87 |
      | lcl|Query\_33212 | KFX31474.1 CRISPR-associated endoribonuclease Cas6 [Geobacillus icigianus] | 248 |
      | lcl|Query\_33213 | KFX31456.1 PAS domain-containing protein [Geobacillus icigianus] | 137 |
      | lcl|Query\_33214 | KFX31457.1 hypothetical protein EP10\_15320 [Geobacillus icigianus] | 101 |
      | lcl|Query\_33215 | KFX31455.2 TetR/AcrR family transcriptional regulator [Geobacillus icigianus] | 218 |
      | lcl|Query\_33216 | KFX31413.1 flagellin [Geobacillus icigianus] | 270 |
      | lcl|Query\_33217 | PUA93216.1 carbon storage regulator [Geobacillus icigianus] | 92 |
      | lcl|Query\_33218 | KFX31414.1 flagellar assembly protein FliW [Geobacillus icigianus] | 147 |
      | lcl|Query\_33219 | KFX31415.1 hypothetical protein EP10\_15350 [Geobacillus icigianus] | 184 |
      | lcl|Query\_33220 | KFX31416.1 flagellar hook-associated protein FlgL [Geobacillus icigianus] | 295 |
      | lcl|Query\_33221 | KFX31417.1 flagellar hook-associated protein FlgK [Geobacillus icigianus] | 533 |
      | lcl|Query\_33222 | KFX31418.2 flagellar protein FlgN [Geobacillus icigianus] | 175 |
      | lcl|Query\_33223 | KFX31419.1 flagellar biosynthesis anti-sigma factor FlgM [Geobacillus icigianus] | 89 |
      | lcl|Query\_33224 | KFX31420.1 hypothetical protein EP10\_15375 [Geobacillus icigianus] | 136 |
      | lcl|Query\_33225 | KFX31421.1 DUF327 domain-containing protein [Geobacillus icigianus] | 146 |
      | lcl|Query\_33226 | KFX31422.1 hypothetical protein EP10\_15385 [Geobacillus icigianus] | 90 |
      | lcl|Query\_33227 | KFX31423.1 hypothetical protein EP10\_15390 [Geobacillus icigianus] | 544 |
      | lcl|Query\_33228 | KFX31424.1 ComF family protein [Geobacillus icigianus] | 230 |
      | lcl|Query\_33229 | KFX31425.1 DNA/RNA helicase [Geobacillus icigianus] | 463 |
      | lcl|Query\_33230 | KFX31426.1 DegV family protein [Geobacillus icigianus] | 281 |
      | lcl|Query\_33231 | KFX31427.1 DNA-binding response regulator [Geobacillus icigianus] | 224 |
      | lcl|Query\_33232 | KFX31428.1 histidine kinase [Geobacillus icigianus] | 387 |
      | lcl|Query\_33233 | KFX31430.1 transcriptional regulator [Geobacillus icigianus] | 334 |
      | lcl|Query\_33234 | KFX31431.1 N-acetylmuramoyl-L-alanine amidase [Geobacillus icigianus] | 741 |
      | lcl|Query\_33235 | KFX31432.1 SH3 domain-containing protein [Geobacillus icigianus] | 225 |
      | lcl|Query\_33236 | KFX31433.1 SpoIID/LytB domain-containing protein [Geobacillus icigianus] | 444 |
      | lcl|Query\_33237 | KFX31434.1 glycosyltransferase WbuB [Geobacillus icigianus] | 413 |
      | lcl|Query\_33238 | KFX31435.1 hypothetical protein EP10\_15450 [Geobacillus icigianus] | 88 |
      | lcl|Query\_33239 | KFX31436.1 glycosyltransferase family 4 protein [Geobacillus icigianus] | 360 |
      | lcl|Query\_33240 | KFX31437.1 glycosyl transferase [Geobacillus icigianus] | 371 |
      | lcl|Query\_33241 | KFX31438.1 oligosaccharide repeat unit polymerase [Geobacillus icigianus] | 462 |
      | lcl|Query\_33242 | KFX31439.1 UDP-N-acetylglucosamine 2-epimerase (non-hydrolyzing) [Geobacillus icigianus] | 352 |
      | lcl|Query\_33243 | KFX31440.1 polysaccharide biosynthesis protein [Geobacillus icigianus] | 482 |
      | lcl|Query\_33244 | KFX31441.1 UDP-3-O-(3-hydroxymyristoyl)glucosamine N-acyltransferase [Geobacillus icigianus] | 243 |
      | lcl|Query\_33245 | KFX31442.1 nucleotide sugar dehydrogenase [Geobacillus icigianus] | 437 |
      | lcl|Query\_33246 | KFX31443.1 gfo/Idh/MocA family oxidoreductase [Geobacillus icigianus] | 337 |
      | lcl|Query\_33247 | KFX31445.1 aminotransferase class V-fold PLP-dependent enzyme [Geobacillus icigianus] | 372 |
      | lcl|Query\_33248 | KFX31446.1 undecaprenyl/decaprenyl-phosphate alpha-N-acetylglucosaminyl 1-phosphate transferase [Geobacillus icigianus] | 360 |
      | lcl|Query\_33249 | KFX31447.1 accessory Sec system translocase SecA2 [Geobacillus icigianus] | 797 |
      | lcl|Query\_33250 | KFX31448.1 accessory Sec system S-layer assembly protein [Geobacillus icigianus] | 297 |
      | lcl|Query\_33251 | KFX31449.1 PDZ domain-containing protein [Geobacillus icigianus] | 465 |
      | lcl|Query\_33252 | KFX31450.1 hypothetical protein EP10\_15530 [Geobacillus icigianus] | 246 |
      | lcl|Query\_33253 | KFX31451.1 hypothetical protein EP10\_15535 [Geobacillus icigianus] | 133 |
      | lcl|Query\_33254 | KFX31452.1 phosphodiester glycosidase family protein [Geobacillus icigianus] | 652 |
      | lcl|Query\_33255 | KFX31453.1 serine protease [Geobacillus icigianus] | 410 |
      | lcl|Query\_33256 | KFX31454.1 hypothetical protein EP10\_15550 [Geobacillus icigianus] | 248 |
      | lcl|Query\_33257 | KFX31429.1 YigZ family protein [Geobacillus icigianus] | 216 |
      | lcl|Query\_33258 | KFX31444.1 lipopolysaccharide biosynthesis protein [Geobacillus icigianus] | 245 |
      | lcl|Query\_33259 | KFX31373.1 UV DNA damage repair endonuclease UvsE [Geobacillus icigianus] | 321 |
      | lcl|Query\_33260 | KFX31374.1 hypothetical protein EP10\_15570 [Geobacillus icigianus] | 160 |
      | lcl|Query\_33261 | KFX31375.1 hypothetical protein EP10\_15575 [Geobacillus icigianus] | 62 |
      | lcl|Query\_33262 | KFX31376.1 hypothetical protein EP10\_15580 [Geobacillus icigianus] | 191 |
      | lcl|Query\_33263 | KFX31377.1 hypothetical protein EP10\_15585 [Geobacillus icigianus] | 212 |
      | lcl|Query\_33264 | KFX31378.1 hypothetical protein EP10\_15590 [Geobacillus icigianus] | 618 |
      | lcl|Query\_33265 | KFX31380.1 hypothetical protein EP10\_15605 [Geobacillus icigianus] | 482 |
      | lcl|Query\_33266 | KFX31381.1 hypothetical protein EP10\_15610 [Geobacillus icigianus] | 160 |
      | lcl|Query\_33267 | KFX31382.1 amino acid permease [Geobacillus icigianus] | 457 |
      | lcl|Query\_33268 | KFX31384.1 DNA topoisomerase III [Geobacillus icigianus] | 696 |
      | lcl|Query\_33269 | KFX31385.1 hypothetical protein EP10\_15630 [Geobacillus icigianus] | 71 |
      | lcl|Query\_33270 | KFX31386.1 hypothetical protein EP10\_15635 [Geobacillus icigianus] | 65 |
      | lcl|Query\_33271 | KFX31387.1 serine/threonine protein kinase [Geobacillus icigianus] | 281 |
      | lcl|Query\_33272 | KFX31388.1 ABC transporter ATP-binding protein [Geobacillus icigianus] | 261 |
      | lcl|Query\_33273 | KFX31390.1 YhgE/Pip domain-containing protein [Geobacillus icigianus] | 709 |
      | lcl|Query\_33274 | KFX31391.1 EamA-like transporter family protein [Geobacillus icigianus] | 144 |
      | lcl|Query\_33275 | KFX31393.1 hypothetical protein EP10\_15680 [Geobacillus icigianus] | 85 |
      | lcl|Query\_33276 | KFX31394.1 multidrug resistance efflux transporter family protein [Geobacillus icigianus] | 333 |
      | lcl|Query\_33277 | PUA93215.1 hypothetical protein EP10\_20335 [Geobacillus icigianus] | 87 |
      | lcl|Query\_33278 | KFX31396.1 dehydratase [Geobacillus icigianus] | 134 |
      | lcl|Query\_33279 | KFX31397.1 MaoC family dehydratase [Geobacillus icigianus] | 149 |
      | lcl|Query\_33280 | KFX31398.1 beta-ketoacyl-ACP reductase [Geobacillus icigianus] | 254 |
      | lcl|Query\_33281 | KFX31399.1 acyl-CoA dehydrogenase [Geobacillus icigianus] | 392 |
      | lcl|Query\_33282 | KFX31400.1 hypothetical protein EP10\_15715 [Geobacillus icigianus] | 187 |
      | lcl|Query\_33283 | KFX31401.1 TetR family transcriptional regulator [Geobacillus icigianus] | 196 |
      | lcl|Query\_33284 | KFX31402.1 oxygen-insensitive NADPH nitroreductase [Geobacillus icigianus] | 249 |
      | lcl|Query\_33285 | KFX31403.1 pirin family protein [Geobacillus icigianus] | 277 |
      | lcl|Query\_33286 | KFX31404.1 hypothetical protein EP10\_15750 [Geobacillus icigianus] | 254 |
      | lcl|Query\_33287 | KFX31405.1 hypothetical protein EP10\_15755 [Geobacillus icigianus] | 156 |
      | lcl|Query\_33288 | KFX31406.1 ABC transporter permease [Geobacillus icigianus] | 326 |
      | lcl|Query\_33289 | KFX31407.1 ABC transporter ATP-binding protein [Geobacillus icigianus] | 304 |
      | lcl|Query\_33290 | KFX31408.1 GDSL family lipase [Geobacillus icigianus] | 263 |
      | lcl|Query\_33291 | KFX31411.1 sulfite exporter TauE/SafE family protein [Geobacillus icigianus] | 294 |
      | lcl|Query\_33292 | KFX31412.1 type I glutamate--ammonia ligase [Geobacillus icigianus] | 452 |
      | lcl|Query\_33293 | KFX31389.1 nitric oxide synthase oxygenase [Geobacillus icigianus] | 359 |
      | lcl|Query\_33294 | KFX31392.1 QueT transporter family protein [Geobacillus icigianus] | 167 |
      | lcl|Query\_33295 | KFX31409.2 hypothetical protein EP10\_15775 [Geobacillus icigianus] | 91 |
      | lcl|Query\_33296 | KFX31338.1 DUF4025 domain-containing protein [Geobacillus icigianus] | 62 |
      | lcl|Query\_33297 | KFX31339.1 DUF2512 domain-containing protein [Geobacillus icigianus] | 171 |
      | lcl|Query\_33298 | KFX31342.1 hypothetical protein EP10\_15840 [Geobacillus icigianus] | 244 |
      | lcl|Query\_33299 | KFX31343.1 MFS transporter [Geobacillus icigianus] | 417 |
      | lcl|Query\_33300 | KFX31344.1 sporulation protein [Geobacillus icigianus] | 251 |
      | lcl|Query\_33301 | KFX31345.1 NAD-dependent malic enzyme [Geobacillus icigianus] | 478 |
      | lcl|Query\_33302 | KFX31346.1 DJ-1/PfpI/YhbO family deglycase/protease [Geobacillus icigianus] | 183 |
      | lcl|Query\_33303 | KFX31348.1 fructosamine kinase [Geobacillus icigianus] | 290 |
      | lcl|Query\_33304 | KFX31349.1 DUF2157 domain-containing protein [Geobacillus icigianus] | 696 |
      | lcl|Query\_33305 | KFX31350.1 hypothetical protein EP10\_15900 [Geobacillus icigianus] | 234 |
      | lcl|Query\_33306 | KFX31351.2 PAP2 family protein [Geobacillus icigianus] | 200 |
      | lcl|Query\_33307 | KFX31352.1 glutamate synthase subunit beta [Geobacillus icigianus] | 494 |
      | lcl|Query\_33308 | KFX31353.1 glutamate synthase large subunit [Geobacillus icigianus] | 1519 |
      | lcl|Query\_33309 | KFX31354.1 LysR family transcriptional regulator [Geobacillus icigianus] | 302 |
      | lcl|Query\_33310 | KFX31355.1 PTS-dependent dihydroxyacetone kinase phosphotransferase subunit DhaM [Geobacillus icigianus] | 130 |
      | lcl|Query\_33311 | KFX31356.1 dihydroxyacetone kinase subunit L [Geobacillus icigianus] | 204 |
      | lcl|Query\_33312 | KFX31357.1 dihydroxyacetone kinase subunit DhaK [Geobacillus icigianus] | 332 |
      | lcl|Query\_33313 | KFX31359.2 Uma2 family endonuclease [Geobacillus icigianus] | 189 |
      | lcl|Query\_33314 | KFX31360.1 aspartate aminotransferase family protein [Geobacillus icigianus] | 450 |
      | lcl|Query\_33315 | KFX31361.1 PucR family transcriptional regulator [Geobacillus icigianus] | 538 |
      | lcl|Query\_33316 | KFX31362.1 GABA permease [Geobacillus icigianus] | 463 |
      | lcl|Query\_33317 | KFX31363.1 hypothetical protein EP10\_15965 [Geobacillus icigianus] | 103 |
      | lcl|Query\_33318 | KFX31364.1 site-specific DNA-methyltransferase [Geobacillus icigianus] | 477 |
      | lcl|Query\_33319 | KFX31365.1 ABC transporter [Geobacillus icigianus] | 321 |
      | lcl|Query\_33320 | KFX31366.1 hypothetical protein EP10\_15985 [Geobacillus icigianus] | 103 |
      | lcl|Query\_33321 | KFX31367.1 iron ABC transporter ATP-binding protein [Geobacillus icigianus] | 251 |
      | lcl|Query\_33322 | KFX31368.1 iron ABC transporter permease [Geobacillus icigianus] | 316 |
      | lcl|Query\_33323 | KFX31369.1 ABC transporter permease [Geobacillus icigianus] | 315 |
      | lcl|Query\_33324 | KFX31370.1 aldehyde dehydrogenase family protein [Geobacillus icigianus] | 488 |
      | lcl|Query\_33325 | KFX31371.1 assimilatory sulfite reductase (NADPH) hemoprotein subunit [Geobacillus icigianus] | 573 |
      | lcl|Query\_33326 | KFX31372.1 assimilatory sulfite reductase (NADPH) flavoprotein subunit [Geobacillus icigianus] | 609 |
      | lcl|Query\_33327 | KFX31337.1 hypothetical protein EP10\_15805 [Geobacillus icigianus] | 135 |
      | lcl|Query\_33328 | KFX31340.2 toxic anion resistance protein [Geobacillus icigianus] | 396 |
      | lcl|Query\_33329 | KFX31341.1 MFS transporter [Geobacillus icigianus] | 217 |
      | lcl|Query\_33330 | PUA93214.1 DUF541 domain-containing protein [Geobacillus icigianus] | 191 |
      | lcl|Query\_33331 | KFX31347.1 NAD-dependent deacylase [Geobacillus icigianus] | 242 |
      | lcl|Query\_33332 | KFX31358.1 oxidoreductase [Geobacillus icigianus] | 328 |
      | lcl|Query\_33333 | KFX31315.1 ABC transporter ATP-binding protein [Geobacillus icigianus] | 641 |
      | lcl|Query\_33334 | KFX31317.1 redox-sensing transcriptional repressor Rex [Geobacillus icigianus] | 220 |
      | lcl|Query\_33335 | KFX31318.1 twin-arginine translocase TatA/TatE family subunit [Geobacillus icigianus] | 50 |
      | lcl|Query\_33336 | KFX31319.1 twin-arginine translocase subunit TatC [Geobacillus icigianus] | 247 |
      | lcl|Query\_33337 | KFX31320.1 DUF4305 domain-containing protein [Geobacillus icigianus] | 59 |
      | lcl|Query\_33338 | KFX31321.1 CPBP family intramembrane metalloprotease [Geobacillus icigianus] | 244 |
      | lcl|Query\_33339 | KFX31322.1 co-chaperone GroES [Geobacillus icigianus] | 94 |
      | lcl|Query\_33340 | KFX31323.1 chaperonin GroEL [Geobacillus icigianus] | 539 |
      | lcl|Query\_33341 | KFX31324.1 hypothetical protein EP10\_16085 [Geobacillus icigianus] | 59 |
      | lcl|Query\_33342 | KFX31325.1 ImmA/IrrE family metallo-endopeptidase [Geobacillus icigianus] | 146 |
      | lcl|Query\_33343 | KFX31327.1 XRE family transcriptional regulator [Geobacillus icigianus] | 70 |
      | lcl|Query\_33344 | KFX31328.1 phage antirepressor Ant [Geobacillus icigianus] | 245 |
      | lcl|Query\_33345 | KFX31330.1 hypothetical protein EP10\_16125 [Geobacillus icigianus] | 94 |
      | lcl|Query\_33346 | KFX31331.1 hypothetical protein EP10\_16130 [Geobacillus icigianus] | 87 |
      | lcl|Query\_33347 | KFX31332.2 hypothetical protein EP10\_16140 [Geobacillus icigianus] | 117 |
      | lcl|Query\_33348 | KFX31333.1 hypothetical protein EP10\_16145 [Geobacillus icigianus] | 59 |
      | lcl|Query\_33349 | KFX31335.1 recombinase RecT [Geobacillus icigianus] | 280 |
      | lcl|Query\_33350 | KFX31336.1 replication protein, partial [Geobacillus icigianus] | 138 |
      | lcl|Query\_33351 | KFX31316.1 cyclic pyranopterin monophosphate synthase MoaC [Geobacillus icigianus] | 162 |
      | lcl|Query\_33352 | KFX31326.2 XRE family transcriptional regulator [Geobacillus icigianus] | 152 |
      | lcl|Query\_33353 | KFX31329.1 DUF771 domain-containing protein [Geobacillus icigianus] | 81 |
      | lcl|Query\_33354 | KFX31334.1 hypothetical protein EP10\_16150 [Geobacillus icigianus] | 321 |
      | lcl|Query\_33355 | KFX31296.1 hypothetical protein EP10\_16180 [Geobacillus icigianus] | 242 |
      | lcl|Query\_33356 | KFX31297.1 molybdopterin converting factor subunit 1 [Geobacillus icigianus] | 76 |
      | lcl|Query\_33357 | KFX31298.1 molybdenum cofactor biosynthesis protein MoaE [Geobacillus icigianus] | 154 |
      | lcl|Query\_33358 | KFX31299.1 molybdopterin-guanine dinucleotide biosynthesis protein B [Geobacillus icigianus] | 166 |
      | lcl|Query\_33359 | KFX31300.1 molybdopterin molybdenumtransferase MoeA [Geobacillus icigianus] | 419 |
      | lcl|Query\_33360 | KFX31301.1 iron-sulfur cluster repair di-iron protein [Geobacillus icigianus] | 235 |
      | lcl|Query\_33361 | KFX31302.1 GTP 3',8-cyclase MoaA [Geobacillus icigianus] | 341 |
      | lcl|Query\_33362 | KFX31303.1 molybdate ABC transporter permease subunit [Geobacillus icigianus] | 220 |
      | lcl|Query\_33363 | KFX31304.1 Crp/Fnr family transcriptional regulator [Geobacillus icigianus] | 236 |
      | lcl|Query\_33364 | KFX31305.1 nitrite reductase [Geobacillus icigianus] | 352 |
      | lcl|Query\_33365 | KFX31306.1 NarK/NasA family nitrate transporter [Geobacillus icigianus] | 391 |
      | lcl|Query\_33366 | KFX31307.1 ABC transporter permease [Geobacillus icigianus] | 359 |
      | lcl|Query\_33367 | KFX31310.2 permease [Geobacillus icigianus] | 498 |
      | lcl|Query\_33368 | KFX31311.1 ABC transporter permease [Geobacillus icigianus] | 259 |
      | lcl|Query\_33369 | KFX31312.1 hypothetical protein EP10\_16270 [Geobacillus icigianus] | 100 |
      | lcl|Query\_33370 | KFX31313.1 hypothetical protein EP10\_16275 [Geobacillus icigianus] | 389 |
      | lcl|Query\_33371 | KFX31314.1 radical SAM/SPASM domain-containing protein [Geobacillus icigianus] | 372 |
      | lcl|Query\_33372 | KFX31309.1 ABC transporter ATP-binding protein [Geobacillus icigianus] | 212 |
      | lcl|Query\_33373 | KFX31294.1 ATP-dependent helicase, partial [Geobacillus icigianus] | 147 |
      | lcl|Query\_33374 | KFX31295.1 ATP-dependent endonuclease [Geobacillus icigianus] | 657 |
      | lcl|Query\_33375 | KFX31290.2 HNH endonuclease [Geobacillus icigianus] | 258 |
      | lcl|Query\_33376 | KFX31291.1 restriction endonuclease [Geobacillus icigianus] | 475 |
      | lcl|Query\_33377 | KFX31292.1 SAM-dependent methyltransferase [Geobacillus icigianus] | 491 |
      | lcl|Query\_33378 | KFX31293.1 type I restriction-modification system endonuclease [Geobacillus icigianus] | 1080 |
      | lcl|Query\_33379 | PUA93213.1 hypothetical protein EP10\_20385 [Geobacillus icigianus] | 207 |
      | lcl|Query\_33380 | KFX31286.1 DUF600 domain-containing protein [Geobacillus icigianus] | 155 |
      | lcl|Query\_33381 | KFX31287.2 hypothetical protein EP10\_16360 [Geobacillus icigianus] | 212 |
      | lcl|Query\_33382 | PUA93210.1 hypothetical protein EP10\_20390 [Geobacillus icigianus] | 195 |
      | lcl|Query\_33383 | PUA93211.1 hypothetical protein EP10\_20395 [Geobacillus icigianus] | 142 |
      | lcl|Query\_33384 | PUA93212.1 hypothetical protein EP10\_20400 [Geobacillus icigianus] | 90 |
      | lcl|Query\_33385 | KFX31285.2 hypothetical protein EP10\_16345 [Geobacillus icigianus] | 182 |
      | lcl|Query\_33386 | KFX31284.1 Abortive infection protein AbiEi, partial [Geobacillus icigianus] | 175 |
      | lcl|Query\_33387 | KFX31281.1 cupin domain-containing protein [Geobacillus icigianus] | 456 |
      | lcl|Query\_33388 | KFX31282.1 glycosyltransferase family 4 protein [Geobacillus icigianus] | 377 |
      | lcl|Query\_33389 | KFX31283.2 glycosyltransferase family 1 protein [Geobacillus icigianus] | 316 |
      | lcl|Query\_33390 | PUA93209.1 recombinase XerD, partial [Geobacillus icigianus] | 244 |
      | lcl|Query\_33391 | PUA93206.1 transposase family protein [Geobacillus icigianus] | 115 |
      | lcl|Query\_33392 | PUA93207.1 hypothetical protein EP10\_20415 [Geobacillus icigianus] | 150 |
      | lcl|Query\_33393 | PUA93208.1 hypothetical protein EP10\_20420, partial [Geobacillus icigianus] | 72 |
      | lcl|Query\_33394 | KFX31280.1 AAA family ATPase, partial [Geobacillus icigianus] | 244 |
      | lcl|Query\_33395 | KFX31250.1 ammonium transporter [Geobacillus icigianus] | 433 |
      | lcl|Query\_33396 | KFX31251.1 nucleotidyltransferase [Geobacillus icigianus] | 350 |
      | lcl|Query\_33397 | KFX31252.1 3'-5' exonuclease [Geobacillus icigianus] | 242 |
      | lcl|Query\_33398 | KFX31253.1 hypothetical protein EP10\_16525 [Geobacillus icigianus] | 162 |
      | lcl|Query\_33399 | KFX31255.1 ABC transporter ATP-binding protein [Geobacillus icigianus] | 270 |
      | lcl|Query\_33400 | KFX31256.1 iron reductase [Geobacillus icigianus] | 245 |
      | lcl|Query\_33401 | KFX31257.1 GGDEF domain-containing protein [Geobacillus icigianus] | 654 |
      | lcl|Query\_33402 | KFX31258.1 histidine phosphatase family protein [Geobacillus icigianus] | 208 |
      | lcl|Query\_33403 | KFX31259.1 ferrous iron transporter B [Geobacillus icigianus] | 464 |
      | lcl|Query\_33404 | KFX31260.1 iron transporter FeoB [Geobacillus icigianus] | 244 |
      | lcl|Query\_33405 | KFX31261.1 ferrous iron transport protein A [Geobacillus icigianus] | 84 |
      | lcl|Query\_33406 | KFX31262.2 acetoacetate--CoA ligase [Geobacillus icigianus] | 671 |
      | lcl|Query\_33407 | KFX31263.1 hypothetical protein EP10\_16585 [Geobacillus icigianus] | 128 |
      | lcl|Query\_33408 | KFX31265.1 multidrug ABC transporter permease [Geobacillus icigianus] | 257 |
      | lcl|Query\_33409 | KFX31267.1 MerR family DNA-binding transcriptional regulator [Geobacillus icigianus] | 129 |
      | lcl|Query\_33410 | KFX31270.1 isovaleryl-CoA dehydrogenase [Geobacillus icigianus] | 560 |
      | lcl|Query\_33411 | KFX31271.1 SOS response-associated peptidase [Geobacillus icigianus] | 222 |
      | lcl|Query\_33412 | KFX31272.1 DUF393 domain-containing protein [Geobacillus icigianus] | 134 |
      | lcl|Query\_33413 | KFX31273.1 hypothetical protein EP10\_16640 [Geobacillus icigianus] | 131 |
      | lcl|Query\_33414 | PUA93205.1 bifunctional ADP-dependent NAD(P)H-hydrate dehydratase/NAD(P)H-hydrate epimerase [Geobacillus icigianus] | 518 |
      | lcl|Query\_33415 | KFX31274.1 N-acetyltransferase [Geobacillus icigianus] | 145 |
      | lcl|Query\_33416 | KFX31275.1 IclR family transcriptional regulator [Geobacillus icigianus] | 249 |
      | lcl|Query\_33417 | KFX31276.1 allophanate hydrolase subunit 1 [Geobacillus icigianus] | 228 |
      | lcl|Query\_33418 | KFX31277.1 KipI antagonist [Geobacillus icigianus] | 323 |
      | lcl|Query\_33419 | KFX31278.1 LamB/YcsF family protein [Geobacillus icigianus] | 260 |
      | lcl|Query\_33420 | KFX31279.1 DUF1722 domain-containing protein [Geobacillus icigianus] | 322 |
      | lcl|Query\_33421 | KFX31254.1 iron-siderophore ABC transporter substrate-binding protein [Geobacillus icigianus] | 320 |
      | lcl|Query\_33422 | KFX31264.1 ABC transporter [Geobacillus icigianus] | 323 |
      | lcl|Query\_33423 | KFX31266.1 multidrug transporter [Geobacillus icigianus] | 262 |
      | lcl|Query\_33424 | PUA93204.1 hypothetical protein EP10\_20445, partial [Geobacillus icigianus] | 39 |
      | lcl|Query\_33425 | KFX31244.1 UDP-4-amino-4,6-dideoxy-N-acetyl-beta-L-altrosamine transaminase [Geobacillus icigianus] | 381 |
      | lcl|Query\_33426 | KFX31245.2 pseudaminic acid synthase [Geobacillus icigianus] | 351 |
      | lcl|Query\_33427 | KFX31246.1 UDP-2,4-diacetamido-2,4,6-trideoxy-beta-L-altropyranose hydrolase [Geobacillus icigianus] | 353 |
      | lcl|Query\_33428 | KFX31248.1 acylneuraminate cytidylyltransferase [Geobacillus icigianus] | 241 |
      | lcl|Query\_33429 | KFX31249.2 carbamoyl-phosphate synthase [Geobacillus icigianus] | 307 |
      | lcl|Query\_33430 | PUA93202.1 MFS transporter [Geobacillus icigianus] | 339 |
      | lcl|Query\_33431 | PUA93203.1 general secretion pathway protein A [Geobacillus icigianus] | 48 |
      | lcl|Query\_33432 | KFX31068.1 16S rRNA (cytosine(967)-C(5))-methyltransferase RsmB, partial [Geobacillus icigianus] | 280 |
      | lcl|Query\_33433 | KFX31069.1 23S rRNA (adenine(2503)-C(2))-methyltransferase RlmN [Geobacillus icigianus] | 366 |
      | lcl|Query\_33434 | KFX31070.1 Stp1/IreP family PP2C-type Ser/Thr phosphatase [Geobacillus icigianus] | 249 |
      | lcl|Query\_33435 | KFX31072.1 ribosome small subunit-dependent GTPase A [Geobacillus icigianus] | 293 |
      | lcl|Query\_33436 | KFX31073.1 ribulose-phosphate 3-epimerase [Geobacillus icigianus] | 218 |
      | lcl|Query\_33437 | KFX31074.1 thiamine diphosphokinase [Geobacillus icigianus] | 216 |
      | lcl|Query\_33438 | KFX31075.1 stage V sporulation protein SpoVM [Geobacillus icigianus] | 26 |
      | lcl|Query\_33439 | KFX31076.1 50S ribosomal protein L28 [Geobacillus icigianus] | 61 |
      | lcl|Query\_33440 | KFX31077.1 Asp23/Gls24 family envelope stress response protein [Geobacillus icigianus] | 120 |
      | lcl|Query\_33441 | KFX31078.1 DAK2 domain-containing protein [Geobacillus icigianus] | 555 |
      | lcl|Query\_33442 | KFX31079.1 L-serine ammonia-lyase, iron-sulfur-dependent, subunit beta [Geobacillus icigianus] | 220 |
      | lcl|Query\_33443 | KFX31080.1 L-serine ammonia-lyase, iron-sulfur-dependent, subunit alpha [Geobacillus icigianus] | 294 |
      | lcl|Query\_33444 | KFX31081.1 DNA helicase RecG [Geobacillus icigianus] | 682 |
      | lcl|Query\_33445 | KFX31082.1 transcription factor FapR [Geobacillus icigianus] | 198 |
      | lcl|Query\_33446 | KFX31083.1 phosphate acyltransferase PlsX [Geobacillus icigianus] | 333 |
      | lcl|Query\_33447 | KFX31084.1 [acyl-carrier-protein] S-malonyltransferase [Geobacillus icigianus] | 313 |
      | lcl|Query\_33448 | KFX31085.1 3-oxoacyl-[acyl-carrier-protein] reductase [Geobacillus icigianus] | 247 |
      | lcl|Query\_33449 | KFX31086.1 acyl carrier protein [Geobacillus icigianus] | 77 |
      | lcl|Query\_33450 | KFX31087.1 ribonuclease III [Geobacillus icigianus] | 244 |
      | lcl|Query\_33451 | KFX31088.1 chromosome segregation protein SMC [Geobacillus icigianus] | 1187 |
      | lcl|Query\_33452 | KFX31089.1 signal recognition particle-docking protein FtsY [Geobacillus icigianus] | 328 |
      | lcl|Query\_33453 | KFX31090.1 putative DNA-binding protein [Geobacillus icigianus] | 110 |
      | lcl|Query\_33454 | KFX31091.1 signal recognition particle protein [Geobacillus icigianus] | 446 |
      | lcl|Query\_33455 | KFX31092.1 30S ribosomal protein S16 [Geobacillus icigianus] | 90 |
      | lcl|Query\_33456 | KFX31094.1 hypothetical protein EP10\_16855 [Geobacillus icigianus] | 128 |
      | lcl|Query\_33457 | KFX31095.1 ribosome maturation factor RimM [Geobacillus icigianus] | 172 |
      | lcl|Query\_33458 | KFX31096.1 tRNA (guanosine(37)-N1)-methyltransferase TrmD [Geobacillus icigianus] | 244 |
      | lcl|Query\_33459 | KFX31097.1 50S ribosomal protein L19 [Geobacillus icigianus] | 116 |
      | lcl|Query\_33460 | KFX31098.1 signal peptidase I [Geobacillus icigianus] | 183 |
      | lcl|Query\_33461 | KFX31100.1 ribonuclease HII [Geobacillus icigianus] | 258 |
      | lcl|Query\_33462 | KFX31101.1 hypothetical protein EP10\_16890 [Geobacillus icigianus] | 81 |
      | lcl|Query\_33463 | KFX31102.1 flagellar hook-length control protein FliK [Geobacillus icigianus] | 512 |
      | lcl|Query\_33464 | KFX31103.1 type III secretion system protein [Geobacillus icigianus] | 95 |
      | lcl|Query\_33465 | KFX31104.1 ADP-forming succinate--CoA ligase subunit beta [Geobacillus icigianus] | 386 |
      | lcl|Query\_33466 | KFX31105.1 succinate--CoA ligase subunit alpha [Geobacillus icigianus] | 300 |
      | lcl|Query\_33467 | KFX31106.1 DNA-protecting protein DprA [Geobacillus icigianus] | 293 |
      | lcl|Query\_33468 | KFX31107.1 type I DNA topoisomerase [Geobacillus icigianus] | 691 |
      | lcl|Query\_33469 | KFX31108.1 tyrosine recombinase XerC [Geobacillus icigianus] | 300 |
      | lcl|Query\_33470 | KFX31109.1 ATP-dependent protease subunit HslV [Geobacillus icigianus] | 180 |
      | lcl|Query\_33471 | KFX31111.1 GTP-sensing pleiotropic transcriptional regulator CodY [Geobacillus icigianus] | 259 |
      | lcl|Query\_33472 | KFX31112.1 flagellar basal body rod protein FlgB [Geobacillus icigianus] | 129 |
      | lcl|Query\_33473 | KFX31113.1 flagellar basal body rod protein FlgC [Geobacillus icigianus] | 150 |
      | lcl|Query\_33474 | KFX31114.1 flagellar hook-basal body complex protein FliE [Geobacillus icigianus] | 98 |
      | lcl|Query\_33475 | KFX31115.1 flagellar basal body M-ring protein FliF [Geobacillus icigianus] | 527 |
      | lcl|Query\_33476 | KFX31116.1 flagellar motor switch protein FliG [Geobacillus icigianus] | 339 |
      | lcl|Query\_33477 | KFX31117.1 flagellar assembly protein FliH [Geobacillus icigianus] | 259 |
      | lcl|Query\_33478 | KFX31118.2 flagellar protein export ATPase FliI [Geobacillus icigianus] | 436 |
      | lcl|Query\_33479 | KFX31119.2 flagellar export protein FliJ [Geobacillus icigianus] | 148 |
      | lcl|Query\_33480 | KFX31120.1 MgtE protein [Geobacillus icigianus] | 203 |
      | lcl|Query\_33481 | KFX31122.1 flagellar hook assembly protein FlgD [Geobacillus icigianus] | 142 |
      | lcl|Query\_33482 | KFX31123.1 flagellar basal-body rod protein FlgF [Geobacillus icigianus] | 265 |
      | lcl|Query\_33483 | KFX31125.1 flagellar basal body-associated protein FliL [Geobacillus icigianus] | 142 |
      | lcl|Query\_33484 | KFX31126.1 flagellar motor switch protein FliM [Geobacillus icigianus] | 334 |
      | lcl|Query\_33485 | KFX31127.1 flagellar motor switch phosphatase FliY [Geobacillus icigianus] | 387 |
      | lcl|Query\_33486 | KFX31128.1 response regulator [Geobacillus icigianus] | 119 |
      | lcl|Query\_33487 | KFX31130.1 flagellar biosynthetic protein FliP [Geobacillus icigianus] | 222 |
      | lcl|Query\_33488 | KFX31131.1 flagellar biosynthetic protein FliQ [Geobacillus icigianus] | 89 |
      | lcl|Query\_33489 | KFX31132.1 flagellar type III secretion system protein FliR [Geobacillus icigianus] | 257 |
      | lcl|Query\_33490 | KFX31133.1 flagellar biosynthesis protein FlhB [Geobacillus icigianus] | 359 |
      | lcl|Query\_33491 | KFX31134.1 flagellar biosynthesis protein FlhA [Geobacillus icigianus] | 681 |
      | lcl|Query\_33492 | KFX31135.1 flagellar biosynthesis protein FlhF [Geobacillus icigianus] | 370 |
      | lcl|Query\_33493 | KFX31136.1 cobyrinic acid a,c-diamide synthase [Geobacillus icigianus] | 289 |
      | lcl|Query\_33494 | KFX31137.1 chemotaxis response regulator protein-glutamate methylesterase [Geobacillus icigianus] | 348 |
      | lcl|Query\_33495 | KFX31138.1 chemotaxis protein CheA [Geobacillus icigianus] | 667 |
      | lcl|Query\_33496 | KFX31139.1 chemotaxis protein CheW [Geobacillus icigianus] | 145 |
      | lcl|Query\_33497 | KFX31140.1 chemotaxis protein CheC [Geobacillus icigianus] | 210 |
      | lcl|Query\_33498 | KFX31141.1 chemotaxis protein CheD [Geobacillus icigianus] | 165 |
      | lcl|Query\_33499 | KFX31142.1 FliA/WhiG family RNA polymerase sigma factor [Geobacillus icigianus] | 251 |
      | lcl|Query\_33500 | KFX31143.1 hypothetical protein EP10\_17100 [Geobacillus icigianus] | 81 |
      | lcl|Query\_33501 | KFX31146.1 elongation factor Ts [Geobacillus icigianus] | 294 |
      | lcl|Query\_33502 | KFX31147.1 UMP kinase [Geobacillus icigianus] | 240 |
      | lcl|Query\_33503 | KFX31148.1 ribosome recycling factor [Geobacillus icigianus] | 185 |
      | lcl|Query\_33504 | KFX31149.1 isoprenyl transferase [Geobacillus icigianus] | 257 |
      | lcl|Query\_33505 | KFX31150.1 phosphatidate cytidylyltransferase [Geobacillus icigianus] | 264 |
      | lcl|Query\_33506 | KFX31151.1 1-deoxy-D-xylulose-5-phosphate reductoisomerase [Geobacillus icigianus] | 382 |
      | lcl|Query\_33507 | KFX31152.1 RIP metalloprotease RseP [Geobacillus icigianus] | 417 |
      | lcl|Query\_33508 | KFX31153.1 proline--tRNA ligase [Geobacillus icigianus] | 567 |
      | lcl|Query\_33509 | KFX31154.1 PolC-type DNA polymerase III [Geobacillus icigianus] | 1433 |
      | lcl|Query\_33510 | KFX31155.1 ribosome assembly cofactor RimP [Geobacillus icigianus] | 157 |
      | lcl|Query\_33511 | KFX31156.1 transcription termination/antitermination protein NusA [Geobacillus icigianus] | 383 |
      | lcl|Query\_33512 | KFX31157.1 DUF448 domain-containing protein [Geobacillus icigianus] | 96 |
      | lcl|Query\_33513 | KFX31158.1 hypothetical protein EP10\_17175 [Geobacillus icigianus] | 102 |
      | lcl|Query\_33514 | KFX31159.1 translation initiation factor IF-2 [Geobacillus icigianus] | 745 |
      | lcl|Query\_33515 | KFX31160.1 DUF503 domain-containing protein [Geobacillus icigianus] | 92 |
      | lcl|Query\_33516 | KFX31161.1 30S ribosome-binding factor RbfA [Geobacillus icigianus] | 123 |
      | lcl|Query\_33517 | KFX31162.1 tRNA pseudouridine(55) synthase TruB [Geobacillus icigianus] | 303 |
      | lcl|Query\_33518 | KFX31164.1 30S ribosomal protein S15 [Geobacillus icigianus] | 89 |
      | lcl|Query\_33519 | KFX31165.1 polyribonucleotide nucleotidyltransferase [Geobacillus icigianus] | 722 |
      | lcl|Query\_33520 | KFX31166.1 hypothetical protein EP10\_17215 [Geobacillus icigianus] | 327 |
      | lcl|Query\_33521 | KFX31167.1 insulinase family protein [Geobacillus icigianus] | 415 |
      | lcl|Query\_33522 | KFX31168.1 YlmC/YmxH family sporulation protein [Geobacillus icigianus] | 79 |
      | lcl|Query\_33523 | KFX31169.1 dipicolinic acid synthetase subunit A [Geobacillus icigianus] | 301 |
      | lcl|Query\_33524 | KFX31170.1 dipicolinate synthase subunit B [Geobacillus icigianus] | 201 |
      | lcl|Query\_33525 | KFX31171.1 aspartate-semialdehyde dehydrogenase [Geobacillus icigianus] | 347 |
      | lcl|Query\_33526 | KFX31172.1 aspartate kinase [Geobacillus icigianus] | 415 |
      | lcl|Query\_33527 | KFX31173.1 4-hydroxy-tetrahydrodipicolinate synthase [Geobacillus icigianus] | 291 |
      | lcl|Query\_33528 | KFX31174.1 RNase J family beta-CASP ribonuclease [Geobacillus icigianus] | 556 |
      | lcl|Query\_33529 | KFX31175.1 peptidoglycan endopeptidase [Geobacillus icigianus] | 237 |
      | lcl|Query\_33530 | KFX31176.1 hypothetical protein EP10\_17275 [Geobacillus icigianus] | 200 |
      | lcl|Query\_33531 | KFX31177.1 Clp protease ClpP [Geobacillus icigianus] | 246 |
      | lcl|Query\_33532 | KFX31178.1 ribonuclease [Geobacillus icigianus] | 74 |
      | lcl|Query\_33533 | KFX31179.1 DNA translocase FtsK [Geobacillus icigianus] | 770 |
      | lcl|Query\_33534 | KFX31180.1 GntR family transcriptional regulator [Geobacillus icigianus] | 243 |
      | lcl|Query\_33535 | KFX31183.1 ABC transporter permease [Geobacillus icigianus] | 348 |
      | lcl|Query\_33536 | KFX31184.1 ABC transporter permease [Geobacillus icigianus] | 319 |
      | lcl|Query\_33537 | KFX31185.1 insulinase family protein [Geobacillus icigianus] | 432 |
      | lcl|Query\_33538 | KFX31186.1 insulinase family protein [Geobacillus icigianus] | 429 |
      | lcl|Query\_33539 | KFX31187.1 SDR family NAD(P)-dependent oxidoreductase [Geobacillus icigianus] | 238 |
      | lcl|Query\_33540 | KFX31188.1 DUF3243 domain-containing protein [Geobacillus icigianus] | 84 |
      | lcl|Query\_33541 | KFX31189.1 DUF3388 domain-containing protein [Geobacillus icigianus] | 263 |
      | lcl|Query\_33542 | KFX31190.1 DUF4115 domain-containing protein [Geobacillus icigianus] | 293 |
      | lcl|Query\_33543 | KFX31191.1 CDP-diacylglycerol--glycerol-3-phosphate 3-phosphatidyltransferase [Geobacillus icigianus] | 192 |
      | lcl|Query\_33544 | KFX31192.1 competence/damage-inducible protein A [Geobacillus icigianus] | 414 |
      | lcl|Query\_33545 | KFX31193.1 recombinase RecA [Geobacillus icigianus] | 347 |
      | lcl|Query\_33546 | KFX31194.1 ribonuclease Y [Geobacillus icigianus] | 518 |
      | lcl|Query\_33547 | KFX31195.1 TIGR00282 family metallophosphoesterase [Geobacillus icigianus] | 264 |
      | lcl|Query\_33548 | KFX31196.1 stage V sporulation protein S [Geobacillus icigianus] | 86 |
      | lcl|Query\_33549 | KFX31197.1 membrane dipeptidase [Geobacillus icigianus] | 307 |
      | lcl|Query\_33550 | KFX31198.1 2-oxoacid:acceptor oxidoreductase subunit alpha [Geobacillus icigianus] | 590 |
      | lcl|Query\_33551 | KFX31199.1 2-oxoacid:ferredoxin oxidoreductase subunit beta [Geobacillus icigianus] | 288 |
      | lcl|Query\_33552 | KFX31200.1 tRNA (N6-isopentenyl adenosine(37)-C2)-methylthiotransferase MiaB [Geobacillus icigianus] | 531 |
      | lcl|Query\_33553 | KFX31201.1 hypothetical protein EP10\_17410 [Geobacillus icigianus] | 143 |
      | lcl|Query\_33554 | KFX31202.1 outer spore coat protein CotE [Geobacillus icigianus] | 182 |
      | lcl|Query\_33555 | KFX31203.1 DNA mismatch repair protein MutS [Geobacillus icigianus] | 895 |
      | lcl|Query\_33556 | KFX31204.1 DNA mismatch repair endonuclease MutL [Geobacillus icigianus] | 649 |
      | lcl|Query\_33557 | KFX31205.1 peptidoglycan endopeptidase [Geobacillus icigianus] | 339 |
      | lcl|Query\_33558 | KFX31206.1 BCCT family transporter [Geobacillus icigianus] | 506 |
      | lcl|Query\_33559 | KFX31207.1 MFS transporter [Geobacillus icigianus] | 449 |
      | lcl|Query\_33560 | KFX31209.1 tRNA (adenosine(37)-N6)-dimethylallyltransferase MiaA [Geobacillus icigianus] | 314 |
      | lcl|Query\_33561 | KFX31210.1 RNA chaperone Hfq [Geobacillus icigianus] | 75 |
      | lcl|Query\_33562 | KFX31211.1 stage V sporulation protein K [Geobacillus icigianus] | 310 |
      | lcl|Query\_33563 | KFX31212.1 fatty acid--CoA ligase [Geobacillus icigianus] | 524 |
      | lcl|Query\_33564 | KFX31213.1 acyl-CoA dehydrogenase [Geobacillus icigianus] | 381 |
      | lcl|Query\_33565 | KFX31214.1 CoA transferase [Geobacillus icigianus] | 357 |
      | lcl|Query\_33566 | KFX31215.1 fatty-acid--CoA ligase [Geobacillus icigianus] | 539 |
      | lcl|Query\_33567 | KFX31217.1 acetyl-CoA C-acyltransferase [Geobacillus icigianus] | 382 |
      | lcl|Query\_33568 | KFX31218.1 EamA/RhaT family transporter [Geobacillus icigianus] | 292 |
      | lcl|Query\_33569 | KFX31219.1 trimeric intracellular cation channel family protein [Geobacillus icigianus] | 204 |
      | lcl|Query\_33570 | KFX31220.1 GTPase HflX [Geobacillus icigianus] | 414 |
      | lcl|Query\_33571 | KFX31221.1 hypothetical protein EP10\_17515 [Geobacillus icigianus] | 423 |
      | lcl|Query\_33572 | KFX31222.1 MerR family transcriptional regulator [Geobacillus icigianus] | 133 |
      | lcl|Query\_33573 | KFX31223.1 type I glutamate--ammonia ligase [Geobacillus icigianus] | 444 |
      | lcl|Query\_33574 | KFX31224.1 transcriptional repressor LexA [Geobacillus icigianus] | 207 |
      | lcl|Query\_33575 | KFX31226.1 DUF896 family protein [Geobacillus icigianus] | 76 |
      | lcl|Query\_33576 | KFX31227.1 transketolase [Geobacillus icigianus] | 668 |
      | lcl|Query\_33577 | KFX31228.1 hypothetical protein EP10\_17555 [Geobacillus icigianus] | 92 |
      | lcl|Query\_33578 | KFX31229.1 sporulation inhibitor of replication protein SirA [Geobacillus icigianus] | 145 |
      | lcl|Query\_33579 | KFX31230.1 hypothetical protein EP10\_17565 [Geobacillus icigianus] | 70 |
      | lcl|Query\_33580 | KFX31232.1 ABC transporter ATP-binding protein [Geobacillus icigianus] | 592 |
      | lcl|Query\_33581 | KFX31233.1 aspartyl-phosphate phosphatase Spo0E family protein [Geobacillus icigianus] | 59 |
      | lcl|Query\_33582 | KFX31234.1 cytochrome c biogenesis protein CcdA [Geobacillus icigianus] | 235 |
      | lcl|Query\_33583 | KFX31235.1 response regulator [Geobacillus icigianus] | 118 |
      | lcl|Query\_33584 | KFX31237.1 DUF2621 domain-containing protein [Geobacillus icigianus] | 144 |
      | lcl|Query\_33585 | KFX31238.1 LysM peptidoglycan-binding domain-containing protein [Geobacillus icigianus] | 216 |
      | lcl|Query\_33586 | KFX31239.1 hypothetical protein EP10\_17615 [Geobacillus icigianus] | 98 |
      | lcl|Query\_33587 | KFX31240.1 sigma-54-dependent transcriptional regulator [Geobacillus icigianus] | 895 |
      | lcl|Query\_33588 | KFX31241.1 PTS sugar transporter subunit IIB [Geobacillus icigianus] | 100 |
      | lcl|Query\_33589 | KFX31242.1 chitin disaccharide deacetylase [Geobacillus icigianus] | 245 |
      | lcl|Query\_33590 | KFX31243.1 PTS lactose/cellobiose transporter subunit IIA [Geobacillus icigianus] | 106 |
      | lcl|Query\_33591 | KFX31071.1 Stk1 family PASTA domain-containing Ser/Thr kinase [Geobacillus icigianus] | 658 |
      | lcl|Query\_33592 | KFX31093.1 KH domain-containing protein [Geobacillus icigianus] | 78 |
      | lcl|Query\_33593 | KFX31099.1 ribosome biogenesis GTPase YlqF [Geobacillus icigianus] | 286 |
      | lcl|Query\_33594 | KFX31110.1 HslU--HslV peptidase ATPase subunit [Geobacillus icigianus] | 464 |
      | lcl|Query\_33595 | KFX31124.1 hypothetical protein EP10\_17005 [Geobacillus icigianus] | 74 |
      | lcl|Query\_33596 | KFX31129.2 flagellar protein [Geobacillus icigianus] | 217 |
      | lcl|Query\_33597 | KFX31144.1 hypothetical protein EP10\_17105 [Geobacillus icigianus] | 222 |
      | lcl|Query\_33598 | KFX31145.1 30S ribosomal protein S2 [Geobacillus icigianus] | 235 |
      | lcl|Query\_33599 | KFX31163.2 bifunctional riboflavin kinase/FAD synthetase [Geobacillus icigianus] | 328 |
      | lcl|Query\_33600 | PUA93200.1 hypothetical protein EP10\_20465 [Geobacillus icigianus] | 72 |
      | lcl|Query\_33601 | KFX31181.1 BMP family ABC transporter substrate-binding protein [Geobacillus icigianus] | 363 |
      | lcl|Query\_33602 | KFX31182.1 ABC transporter ATP-binding protein [Geobacillus icigianus] | 509 |
      | lcl|Query\_33603 | KFX31208.1 methyl-accepting chemotaxis protein [Geobacillus icigianus] | 565 |
      | lcl|Query\_33604 | PUA93201.1 hypothetical protein EP10\_20470 [Geobacillus icigianus] | 23 |
      | lcl|Query\_33605 | KFX31225.1 cell division suppressor protein YneA [Geobacillus icigianus] | 101 |
      | lcl|Query\_33606 | KFX31231.1 multidrug ABC transporter permease/ATP-binding protein [Geobacillus icigianus] | 587 |
      | lcl|Query\_33607 | KFX31236.2 hypothetical protein EP10\_17595 [Geobacillus icigianus] | 157 |
      | lcl|Query\_33608 | PUA93199.1 IS1634 family transposase [Geobacillus icigianus] | 461 |
      | lcl|Query\_33609 | KFX31066.2 ABC transporter ATP-binding protein [Geobacillus icigianus] | 270 |
      | lcl|Query\_33610 | PUA93197.1 hypothetical protein EP10\_20495, partial [Geobacillus icigianus] | 1419 |
      | lcl|Query\_33611 | PUA93198.1 hypothetical protein EP10\_20500 [Geobacillus icigianus] | 70 |
      | lcl|Query\_33612 | PUA93194.1 hypothetical protein EP10\_20505 [Geobacillus icigianus] | 182 |
      | lcl|Query\_33613 | PUA93195.1 general secretion pathway protein A [Geobacillus icigianus] | 57 |
      | lcl|Query\_33614 | PUA93196.1 hypothetical protein EP10\_20525 [Geobacillus icigianus] | 91 |
      | lcl|Query\_33615 | KFX31055.1 FAD-binding protein [Geobacillus icigianus] | 455 |
      | lcl|Query\_33616 | KFX31056.1 alpha-ketoacid dehydrogenase subunit beta [Geobacillus icigianus] | 325 |
      | lcl|Query\_33617 | KFX31057.1 pyruvate dehydrogenase (acetyl-transferring) E1 component subunit alpha [Geobacillus icigianus] | 359 |
      | lcl|Query\_33618 | KFX31058.1 2-oxo acid dehydrogenase subunit E2 [Geobacillus icigianus] | 430 |
      | lcl|Query\_33619 | KFX31059.1 hypothetical protein EP10\_17720 [Geobacillus icigianus] | 64 |
      | lcl|Query\_33620 | KFX31060.1 hypothetical protein EP10\_17725 [Geobacillus icigianus] | 131 |
      | lcl|Query\_33621 | KFX31061.1 hypothetical protein EP10\_17730 [Geobacillus icigianus] | 121 |
      | lcl|Query\_33622 | KFX31062.1 gfo/Idh/MocA family oxidoreductase [Geobacillus icigianus] | 328 |
      | lcl|Query\_33623 | KFX31063.1 DUF4275 domain-containing protein [Geobacillus icigianus] | 145 |
      | lcl|Query\_33624 | PUA93193.1 hypothetical protein EP10\_20535 [Geobacillus icigianus] | 59 |
      | lcl|Query\_33625 | KFX31065.1 DUF1360 domain-containing protein [Geobacillus icigianus] | 127 |
      | lcl|Query\_33626 | KFX31064.1 arsenate reductase (thioredoxin) [Geobacillus icigianus] | 139 |
      | lcl|Query\_33627 | KFX31052.1 site-2 protease family protein [Geobacillus icigianus] | 222 |
      | lcl|Query\_33628 | KFX31053.1 hypothetical protein EP10\_17775 [Geobacillus icigianus] | 171 |
      | lcl|Query\_33629 | KFX31051.1 4-oxalocrotonate tautomerase [Geobacillus icigianus] | 62 |
      | lcl|Query\_33630 | KFX31050.1 hypothetical protein EP10\_17790 [Geobacillus icigianus] | 195 |
      | lcl|Query\_33631 | PUA93192.1 hypothetical protein EP10\_20550, partial [Geobacillus icigianus] | 210 |
      | lcl|Query\_33632 | KFX31041.1 glutamine--fructose-6-phosphate transaminase (isomerizing) [Geobacillus icigianus] | 600 |
      | lcl|Query\_33633 | KFX31042.1 phosphoglucosamine mutase [Geobacillus icigianus] | 449 |
      | lcl|Query\_33634 | KFX31043.1 YbbR-like domain-containing protein [Geobacillus icigianus] | 412 |
      | lcl|Query\_33635 | KFX31044.1 TIGR00159 family protein [Geobacillus icigianus] | 273 |
      | lcl|Query\_33636 | KFX31045.1 anti-sigma factor [Geobacillus icigianus] | 203 |
      | lcl|Query\_33637 | KFX31046.1 RNA polymerase sigma factor SigW [Geobacillus icigianus] | 187 |
      | lcl|Query\_33638 | KFX31047.1 arginase [Geobacillus icigianus] | 299 |
      | lcl|Query\_33639 | KFX31048.1 hypothetical protein EP10\_17845 [Geobacillus icigianus] | 65 |
      | lcl|Query\_33640 | KFX31036.1 hypothetical protein EP10\_17890 [Geobacillus icigianus] | 196 |
      | lcl|Query\_33641 | KFX31037.1 hypothetical protein EP10\_17895 [Geobacillus icigianus] | 141 |
      | lcl|Query\_33642 | KFX31038.1 hypothetical protein EP10\_17905 [Geobacillus icigianus] | 149 |
      | lcl|Query\_33643 | KFX31040.1 IS982 family transposase [Geobacillus icigianus] | 292 |
      | lcl|Query\_33644 | KFX31039.1 hypothetical protein EP10\_17910 [Geobacillus icigianus] | 64 |
      | lcl|Query\_33645 | KFX31034.1 DUF4145 domain-containing protein [Geobacillus icigianus] | 1113 |
      | lcl|Query\_33646 | KFX31035.1 hypothetical protein EP10\_17940 [Geobacillus icigianus] | 62 |
      | lcl|Query\_33647 | PUA93191.1 type I restriction endonuclease subunit R, partial [Geobacillus icigianus] | 92 |
      | lcl|Query\_33648 | KFX31025.1 SAM-dependent DNA methyltransferase [Geobacillus icigianus] | 485 |
      | lcl|Query\_33649 | KFX31026.1 23S rRNA (uracil(1939)-C(5))-methyltransferase RlmD [Geobacillus icigianus] | 468 |
      | lcl|Query\_33650 | KFX31027.1 DNA-3-methyladenine glycosylase 2 family protein [Geobacillus icigianus] | 288 |
      | lcl|Query\_33651 | KFX31028.1 delta-lactam-biosynthetic de-N-acetylase [Geobacillus icigianus] | 263 |
      | lcl|Query\_33652 | KFX31029.1 fumarate hydratase [Geobacillus icigianus] | 515 |
      | lcl|Query\_33653 | KFX31030.1 radical SAM/CxCxxxxC motif protein YfkAB [Geobacillus icigianus] | 374 |
      | lcl|Query\_33654 | KFX31031.1 hypothetical protein EP10\_17985 [Geobacillus icigianus] | 262 |
      | lcl|Query\_33655 | KFX31033.1 MFS transporter [Geobacillus icigianus] | 377 |
      | lcl|Query\_33656 | KFX31032.1 calcium/proton exchanger [Geobacillus icigianus] | 350 |
      | lcl|Query\_33657 | KFX31019.2 DUF5082 domain-containing protein [Geobacillus icigianus] | 100 |
      | lcl|Query\_33658 | KFX31022.1 hypothetical protein EP10\_18025 [Geobacillus icigianus] | 93 |
      | lcl|Query\_33659 | KFX31023.1 hypothetical protein EP10\_18040 [Geobacillus icigianus] | 62 |
      | lcl|Query\_33660 | KFX31024.1 TIGR01741 family protein [Geobacillus icigianus] | 160 |
      | lcl|Query\_33661 | KFX31018.2 hypothetical protein EP10\_18000 [Geobacillus icigianus] | 100 |
      | lcl|Query\_33662 | PUA93190.1 hypothetical protein EP10\_20600 [Geobacillus icigianus] | 422 |
      | lcl|Query\_33663 | KFX30984.1 acyl--CoA ligase [Geobacillus icigianus] | 531 |
      | lcl|Query\_33664 | KFX30985.1 NAD kinase [Geobacillus icigianus] | 267 |
      | lcl|Query\_33665 | KFX30986.1 signal peptide peptidase SppA [Geobacillus icigianus] | 335 |
      | lcl|Query\_33666 | KFX30987.1 RDD family protein [Geobacillus icigianus] | 156 |
      | lcl|Query\_33667 | KFX30989.1 sporulation protein YtfJ [Geobacillus icigianus] | 154 |
      | lcl|Query\_33668 | KFX30990.1 thiol peroxidase [Geobacillus icigianus] | 166 |
      | lcl|Query\_33669 | KFX30991.1 class I SAM-dependent methyltransferase [Geobacillus icigianus] | 329 |
      | lcl|Query\_33670 | KFX30994.2 MFS transporter [Geobacillus icigianus] | 516 |
      | lcl|Query\_33671 | KFX30996.1 sterol-binding protein [Geobacillus icigianus] | 114 |
      | lcl|Query\_33672 | KFX30997.1 long-chain fatty acid--CoA ligase [Geobacillus icigianus] | 514 |
      | lcl|Query\_33673 | KFX30998.1 acyl-CoA dehydrogenase [Geobacillus icigianus] | 374 |
      | lcl|Query\_33674 | KFX30999.1 acyl-CoA dehydrogenase [Geobacillus icigianus] | 404 |
      | lcl|Query\_33675 | KFX31000.1 enoyl-CoA hydratase/isomerase family protein [Geobacillus icigianus] | 263 |
      | lcl|Query\_33676 | KFX31001.1 3-oxoacyl-ACP reductase FabG [Geobacillus icigianus] | 280 |
      | lcl|Query\_33677 | KFX31002.1 acetyl-CoA C-acyltransferase [Geobacillus icigianus] | 380 |
      | lcl|Query\_33678 | KFX31003.1 AarF/ABC1/UbiB kinase family protein [Geobacillus icigianus] | 581 |
      | lcl|Query\_33679 | KFX31004.1 hypothetical protein EP10\_18155 [Geobacillus icigianus] | 110 |
      | lcl|Query\_33680 | KFX31005.2 alcohol dehydrogenase [Geobacillus icigianus] | 368 |
      | lcl|Query\_33681 | KFX31006.1 hypothetical protein EP10\_18165 [Geobacillus icigianus] | 142 |
      | lcl|Query\_33682 | KFX31007.1 aldehyde dehydrogenase [Geobacillus icigianus] | 495 |
      | lcl|Query\_33683 | KFX31008.1 R2-like ligand-binding oxidase [Geobacillus icigianus] | 302 |
      | lcl|Query\_33684 | KFX31009.1 hypothetical protein EP10\_18180 [Geobacillus icigianus] | 201 |
      | lcl|Query\_33685 | KFX31012.1 molybdenum cofactor biosynthesis protein MoaB [Geobacillus icigianus] | 170 |
      | lcl|Query\_33686 | KFX31013.1 MFS transporter [Geobacillus icigianus] | 400 |
      | lcl|Query\_33687 | KFX31014.1 ATP-grasp domain-containing protein [Geobacillus icigianus] | 398 |
      | lcl|Query\_33688 | KFX31015.1 transcriptional regulator [Geobacillus icigianus] | 368 |
      | lcl|Query\_33689 | KFX31016.1 acetate--CoA ligase [Geobacillus icigianus] | 552 |
      | lcl|Query\_33690 | KFX31017.1 hypothetical protein EP10\_18225 [Geobacillus icigianus] | 111 |
      | lcl|Query\_33691 | KFX30988.2 hypothetical protein EP10\_18075 [Geobacillus icigianus] | 223 |
      | lcl|Query\_33692 | KFX30992.1 acetate kinase [Geobacillus icigianus] | 396 |
      | lcl|Query\_33693 | KFX30993.1 TetR family transcriptional regulator [Geobacillus icigianus] | 201 |
      | lcl|Query\_33694 | KFX30995.1 hypothetical protein EP10\_18110 [Geobacillus icigianus] | 170 |
      | lcl|Query\_33695 | KFX31010.1 hypothetical protein EP10\_18185 [Geobacillus icigianus] | 380 |
      | lcl|Query\_33696 | PUA93187.1 hypothetical protein EP10\_20605 [Geobacillus icigianus] | 69 |
      | lcl|Query\_33697 | KFX30908.1 SMC-Scp complex subunit ScpB [Geobacillus icigianus] | 208 |
      | lcl|Query\_33698 | KFX30909.1 segregation/condensation protein A [Geobacillus icigianus] | 251 |
      | lcl|Query\_33699 | KFX30910.1 DUF309 domain-containing protein [Geobacillus icigianus] | 170 |
      | lcl|Query\_33700 | KFX30911.1 N-acetyltransferase [Geobacillus icigianus] | 118 |
      | lcl|Query\_33701 | KFX30912.1 6,7-dimethyl-8-ribityllumazine synthase [Geobacillus icigianus] | 154 |
      | lcl|Query\_33702 | KFX30913.1 bifunctional 3,4-dihydroxy-2-butanone-4-phosphate synthase/GTP cyclohydrolase II [Geobacillus icigianus] | 397 |
      | lcl|Query\_33703 | KFX30914.1 riboflavin synthase [Geobacillus icigianus] | 215 |
      | lcl|Query\_33704 | KFX30915.2 bifunctional diaminohydroxyphosphoribosylaminopyrimidine deaminase/5-amino-6-(5-phosphoribosylamino)uracil reductase RibD [Geobacillus icigianus] | 366 |
      | lcl|Query\_33705 | KFX30916.1 peptidylprolyl isomerase [Geobacillus icigianus] | 146 |
      | lcl|Query\_33706 | KFX30917.1 DUF1002 domain-containing protein [Geobacillus icigianus] | 296 |
      | lcl|Query\_33707 | KFX30919.1 spore germination protein [Geobacillus icigianus] | 491 |
      | lcl|Query\_33708 | KFX30920.1 stage V sporulation protein AE [Geobacillus icigianus] | 202 |
      | lcl|Query\_33709 | KFX30921.1 SpoVA/SpoVAEb family sporulation membrane protein [Geobacillus icigianus] | 116 |
      | lcl|Query\_33710 | KFX30922.1 stage V sporulation protein AD [Geobacillus icigianus] | 346 |
      | lcl|Query\_33711 | KFX30923.1 SpoVA/SpoVAEb family sporulation membrane protein [Geobacillus icigianus] | 142 |
      | lcl|Query\_33712 | KFX30924.1 stage V sporulation protein AB [Geobacillus icigianus] | 143 |
      | lcl|Query\_33713 | KFX30925.1 stage V sporulation protein AA [Geobacillus icigianus] | 207 |
      | lcl|Query\_33714 | KFX30926.1 RNA polymerase sporulation sigma factor SigF [Geobacillus icigianus] | 250 |
      | lcl|Query\_33715 | KFX30927.1 anti-sigma F factor [Geobacillus icigianus] | 146 |
      | lcl|Query\_33716 | KFX30928.1 anti-sigma F factor antagonist [Geobacillus icigianus] | 116 |
      | lcl|Query\_33717 | KFX30929.1 D-alanyl-D-alanine carboxypeptidase [Geobacillus icigianus] | 391 |
      | lcl|Query\_33718 | KFX30930.1 pyrimidine-nucleoside phosphorylase [Geobacillus icigianus] | 433 |
      | lcl|Query\_33719 | KFX30931.1 purine-nucleoside phosphorylase [Geobacillus icigianus] | 273 |
      | lcl|Query\_33720 | KFX30932.1 phosphopentomutase [Geobacillus icigianus] | 395 |
      | lcl|Query\_33721 | KFX30933.1 site-specific tyrosine recombinase XerD [Geobacillus icigianus] | 298 |
      | lcl|Query\_33722 | KFX30934.1 DUF4227 domain-containing protein [Geobacillus icigianus] | 70 |
      | lcl|Query\_33723 | KFX30935.1 transcriptional repressor [Geobacillus icigianus] | 152 |
      | lcl|Query\_33724 | KFX30937.1 TIGR00375 family protein [Geobacillus icigianus] | 396 |
      | lcl|Query\_33725 | KFX30938.1 NUDIX hydrolase [Geobacillus icigianus] | 187 |
      | lcl|Query\_33726 | KFX30939.1 aldo/keto reductase [Geobacillus icigianus] | 306 |
      | lcl|Query\_33727 | KFX30942.1 DUF2552 domain-containing protein [Geobacillus icigianus] | 79 |
      | lcl|Query\_33728 | KFX30943.1 iron-sulfur cluster biosynthesis family protein [Geobacillus icigianus] | 114 |
      | lcl|Query\_33729 | KFX30944.1 SDR family NAD(P)-dependent oxidoreductase [Geobacillus icigianus] | 262 |
      | lcl|Query\_33730 | KFX30945.1 MBL fold metallo-hydrolase [Geobacillus icigianus] | 323 |
      | lcl|Query\_33731 | KFX30946.1 pyrroline-5-carboxylate reductase [Geobacillus icigianus] | 279 |
      | lcl|Query\_33732 | KFX30947.1 NADPH dehydrogenase NamA [Geobacillus icigianus] | 340 |
      | lcl|Query\_33733 | KFX30948.1 ribonuclease Z [Geobacillus icigianus] | 307 |
      | lcl|Query\_33734 | KFX30949.1 glucose-6-phosphate dehydrogenase [Geobacillus icigianus] | 503 |
      | lcl|Query\_33735 | KFX30950.1 cyclase family protein [Geobacillus icigianus] | 205 |
      | lcl|Query\_33736 | KFX30951.2 AbrB/MazE/SpoVT family DNA-binding domain-containing protein [Geobacillus icigianus] | 103 |
      | lcl|Query\_33737 | KFX30952.1 type II toxin-antitoxin system VapC family toxin [Geobacillus icigianus] | 138 |
      | lcl|Query\_33738 | KFX30953.1 glycoside hydrolase family 1 protein [Geobacillus icigianus] | 455 |
      | lcl|Query\_33739 | KFX30954.1 hypothetical protein EP10\_18480 [Geobacillus icigianus] | 129 |
      | lcl|Query\_33740 | KFX30955.1 DNA-binding response regulator [Geobacillus icigianus] | 229 |
      | lcl|Query\_33741 | KFX30956.1 sensor histidine kinase [Geobacillus icigianus] | 334 |
      | lcl|Query\_33742 | KFX30957.1 ABC transporter ATP-binding protein [Geobacillus icigianus] | 251 |
      | lcl|Query\_33743 | KFX30958.1 ABC transporter permease [Geobacillus icigianus] | 641 |
      | lcl|Query\_33744 | PUA93188.1 hypothetical protein EP10\_20610 [Geobacillus icigianus] | 60 |
      | lcl|Query\_33745 | KFX30960.1 phosphogluconate dehydrogenase (NADP(+)-dependent, decarboxylating) [Geobacillus icigianus] | 469 |
      | lcl|Query\_33746 | KFX30961.1 hypothetical protein EP10\_18525 [Geobacillus icigianus] | 64 |
      | lcl|Query\_33747 | KFX30962.1 chemotaxis protein CheW [Geobacillus icigianus] | 157 |
      | lcl|Query\_33748 | KFX30963.1 hypothetical protein EP10\_18540 [Geobacillus icigianus] | 374 |
      | lcl|Query\_33749 | KFX30964.1 acyl-CoA carboxylase subunit beta [Geobacillus icigianus] | 516 |
      | lcl|Query\_33750 | KFX30965.1 methylmalonyl-CoA epimerase [Geobacillus icigianus] | 141 |
      | lcl|Query\_33751 | KFX30966.1 L,D-transpeptidase [Geobacillus icigianus] | 168 |
      | lcl|Query\_33752 | KFX30967.1 aromatic acid exporter family protein [Geobacillus icigianus] | 326 |
      | lcl|Query\_33753 | KFX30968.1 DUF1998 domain-containing protein [Geobacillus icigianus] | 1786 |
      | lcl|Query\_33754 | KFX30969.1 hypothetical protein EP10\_18575 [Geobacillus icigianus] | 317 |
      | lcl|Query\_33755 | KFX30970.1 hypothetical protein EP10\_18580 [Geobacillus icigianus] | 660 |
      | lcl|Query\_33756 | KFX30971.1 hypothetical protein EP10\_18585 [Geobacillus icigianus] | 432 |
      | lcl|Query\_33757 | KFX30972.1 hypothetical protein EP10\_18590 [Geobacillus icigianus] | 61 |
      | lcl|Query\_33758 | KFX30975.1 restriction endonuclease subunit M/S [Geobacillus icigianus] | 634 |
      | lcl|Query\_33759 | KFX30976.1 BrxA/BrxB family bacilliredoxin [Geobacillus icigianus] | 145 |
      | lcl|Query\_33760 | KFX30977.1 methylmalonyl Co-A mutase-associated GTPase MeaB [Geobacillus icigianus] | 382 |
      | lcl|Query\_33761 | KFX30978.1 methylmalonyl-CoA mutase [Geobacillus icigianus] | 731 |
      | lcl|Query\_33762 | KFX30979.1 methylmalonyl-CoA mutase [Geobacillus icigianus] | 682 |
      | lcl|Query\_33763 | KFX30980.1 Uma2 family endonuclease [Geobacillus icigianus] | 194 |
      | lcl|Query\_33764 | PUA93189.1 HAMP domain-containing protein [Geobacillus icigianus] | 483 |
      | lcl|Query\_33765 | KFX30981.1 DNA-binding response regulator [Geobacillus icigianus] | 224 |
      | lcl|Query\_33766 | KFX30982.1 hypothetical protein EP10\_18655 [Geobacillus icigianus] | 325 |
      | lcl|Query\_33767 | KFX30983.1 hypothetical protein EP10\_18660 [Geobacillus icigianus] | 80 |
      | lcl|Query\_33768 | KFX30918.1 diaminopimelate decarboxylase [Geobacillus icigianus] | 439 |
      | lcl|Query\_33769 | KFX30936.1 stage II sporulation protein M [Geobacillus icigianus] | 220 |
      | lcl|Query\_33770 | KFX30941.1 DUF3886 domain-containing protein [Geobacillus icigianus] | 81 |
      | lcl|Query\_33771 | KFX30959.1 hypothetical protein EP10\_18510 [Geobacillus icigianus] | 182 |
      | lcl|Query\_33772 | KFX30907.1 hypothetical protein EP10\_18675 [Geobacillus icigianus] | 76 |
      | lcl|Query\_33773 | KFX30869.1 amino acid permease [Geobacillus icigianus] | 470 |
      | lcl|Query\_33774 | KFX30870.1 L-glutamate gamma-semialdehyde dehydrogenase [Geobacillus icigianus] | 515 |
      | lcl|Query\_33775 | KFX30871.1 ornithine--oxo-acid transaminase [Geobacillus icigianus] | 405 |
      | lcl|Query\_33776 | KFX30872.1 DedA family protein [Geobacillus icigianus] | 199 |
      | lcl|Query\_33777 | KFX30873.1 iron ABC transporter permease [Geobacillus icigianus] | 332 |
      | lcl|Query\_33778 | KFX30874.1 iron ABC transporter permease [Geobacillus icigianus] | 335 |
      | lcl|Query\_33779 | KFX30875.1 ABC transporter substrate-binding protein [Geobacillus icigianus] | 307 |
      | lcl|Query\_33780 | KFX30876.1 ABC transporter ATP-binding protein [Geobacillus icigianus] | 266 |
      | lcl|Query\_33781 | KFX30878.1 two-component sensor histidine kinase [Geobacillus icigianus] | 368 |
      | lcl|Query\_33782 | KFX30879.1 ABC transporter ATP-binding protein [Geobacillus icigianus] | 312 |
      | lcl|Query\_33783 | KFX30880.1 ABC transporter permease [Geobacillus icigianus] | 367 |
      | lcl|Query\_33784 | KFX30881.1 ABC transporter permease [Geobacillus icigianus] | 381 |
      | lcl|Query\_33785 | KFX30882.1 N-acetylmuramoyl-L-alanine amidase [Geobacillus icigianus] | 242 |
      | lcl|Query\_33786 | KFX30883.1 serine protease [Geobacillus icigianus] | 442 |
      | lcl|Query\_33787 | KFX30885.2 Trk family potassium uptake protein [Geobacillus icigianus] | 445 |
      | lcl|Query\_33788 | KFX30886.1 hypothetical protein EP10\_18780 [Geobacillus icigianus] | 75 |
      | lcl|Query\_33789 | KFX30887.1 APC family permease [Geobacillus icigianus] | 611 |
      | lcl|Query\_33790 | KFX30888.1 sporulation membrane protein YtaF [Geobacillus icigianus] | 212 |
      | lcl|Query\_33791 | KFX30889.1 sporulation protein SpoOM [Geobacillus icigianus] | 331 |
      | lcl|Query\_33792 | KFX30890.1 ROK family protein [Geobacillus icigianus] | 307 |
      | lcl|Query\_33793 | KFX30891.1 undecaprenyl-diphosphate phosphatase [Geobacillus icigianus] | 273 |
      | lcl|Query\_33794 | KFX30892.1 undecaprenyldiphospho-muramoylpentapeptide beta-N-acetylglucosaminyltransferase [Geobacillus icigianus] | 356 |
      | lcl|Query\_33795 | KFX30893.1 hypothetical protein EP10\_18815 [Geobacillus icigianus] | 137 |
      | lcl|Query\_33796 | KFX30894.1 polysaccharide deacetylase family protein [Geobacillus icigianus] | 242 |
      | lcl|Query\_33797 | KFX30895.1 DedA family protein [Geobacillus icigianus] | 198 |
      | lcl|Query\_33798 | KFX30896.1 UDP-N-acetylglucosamine--LPS N-acetylglucosamine transferase [Geobacillus icigianus] | 380 |
      | lcl|Query\_33799 | KFX30897.1 D-alanine--D-alanine ligase [Geobacillus icigianus] | 365 |
      | lcl|Query\_33800 | KFX30898.1 UDP-N-acetylmuramoyl-tripeptide--D-alanyl-D-alanine ligase [Geobacillus icigianus] | 458 |
      | lcl|Query\_33801 | KFX30899.1 ATP-dependent helicase [Geobacillus icigianus] | 467 |
      | lcl|Query\_33802 | PUA93185.1 rhomboid family intramembrane serine protease [Geobacillus icigianus] | 212 |
      | lcl|Query\_33803 | KFX30900.1 holo-ACP synthase [Geobacillus icigianus] | 129 |
      | lcl|Query\_33804 | KFX30901.1 outer membrane lipoprotein carrier protein LolA [Geobacillus icigianus] | 337 |
      | lcl|Query\_33805 | KFX30902.1 alanine racemase [Geobacillus icigianus] | 388 |
      | lcl|Query\_33806 | KFX30903.1 antitoxin endoai [Geobacillus icigianus] | 93 |
      | lcl|Query\_33807 | KFX30904.1 type II toxin-antitoxin system PemK/MazF family toxin [Geobacillus icigianus] | 116 |
      | lcl|Query\_33808 | KFX30905.1 RNA-binding transcriptional accessory protein [Geobacillus icigianus] | 721 |
      | lcl|Query\_33809 | PUA93186.1 cortex morphogenetic protein CmpA [Geobacillus icigianus] | 37 |
      | lcl|Query\_33810 | KFX30906.1 SprT family protein [Geobacillus icigianus] | 154 |
      | lcl|Query\_33811 | KFX30877.2 DNA-binding response regulator [Geobacillus icigianus] | 212 |
      | lcl|Query\_33812 | KFX30884.1 LTA synthase family protein [Geobacillus icigianus] | 650 |
      | lcl|Query\_33813 | KFX30863.1 IS4/IS5 family transposase, partial [Geobacillus icigianus] | 160 |
      | lcl|Query\_33814 | KFX30864.1 IS4 family transposase [Geobacillus icigianus] | 453 |
      | lcl|Query\_33815 | PUA93182.1 hypothetical protein EP10\_20655 [Geobacillus icigianus] | 67 |
      | lcl|Query\_33816 | PUA93183.1 insulinase family protein [Geobacillus icigianus] | 64 |
      | lcl|Query\_33817 | KFX30868.1 insulinase family protein [Geobacillus icigianus] | 71 |
      | lcl|Query\_33818 | PUA93184.1 ABC transporter ATP-binding protein, partial [Geobacillus icigianus] | 37 |
      | lcl|Query\_33819 | KFX30861.1 DUF3899 domain-containing protein [Geobacillus icigianus] | 125 |
      | lcl|Query\_33820 | KFX30862.1 tryptophan--tRNA ligase, partial [Geobacillus icigianus] | 121 |
      | lcl|Query\_33821 | KFX30852.1 TIGR02679 family protein [Geobacillus icigianus] | 406 |
      | lcl|Query\_33822 | KFX30854.1 TIGR02678 family protein [Geobacillus icigianus] | 385 |
      | lcl|Query\_33823 | KFX30855.1 TIGR02677 family protein [Geobacillus icigianus] | 503 |
      | lcl|Query\_33824 | KFX30856.1 hypothetical protein EP10\_19015 [Geobacillus icigianus] | 243 |
      | lcl|Query\_33825 | KFX30857.2 glycine/betaine ABC transporter substrate-binding protein [Geobacillus icigianus] | 315 |
      | lcl|Query\_33826 | KFX30858.1 CBS domain-containing protein [Geobacillus icigianus] | 375 |
      | lcl|Query\_33827 | KFX30859.1 ABC transporter permease [Geobacillus icigianus] | 209 |
      | lcl|Query\_33828 | KFX30860.1 hypothetical protein EP10\_19040 [Geobacillus icigianus] | 78 |
      | lcl|Query\_33829 | KFX30853.1 TIGR02680 family protein [Geobacillus icigianus] | 1372 |
      | lcl|Query\_33830 | PUA93181.1 transporter, partial [Geobacillus icigianus] | 78 |
      | lcl|Query\_33831 | KFX30851.1 IS701 family transposase, partial [Geobacillus icigianus] | 220 |

Subject Length
:   921721

Other reports
:   Distance tree of results
    Multiple alignment
    MSA viewer
     Help

    Reports are generated on using all sequences producing significant alignments. To generate reports on a subset of sequences, use the report links in the Descriptions tab while selecting specific sequences.

## Filter Results

Organism only top 20 will appear


exclude

Add organism


---

Percent Identity from

Percent Identity to

E value from

E value to

Query Coverage from

Query Coverage to

Filter Reset

- Descriptions

  ### Sequences producing significant alignments

  - Download
    - FASTA (complete sequence)
    - FASTA (aligned sequences)
    - GenBank (complete sequence)
    - Hit Table (text)
    - Hit Table (CSV)
    - Text
    - Descriptions Table (CSV)
    - XML
    - ASN.1
  - Manage Columns
    - Description
    - Max Score
    - Total Score
    - Query Coverage
    - E value
    - Percent Identity
    - Accession
    - Restore defaults
  - Show

    10
    50
    100
  - Help

    Subject sequences can be removed or added from within the Descriptions tab and the selections will carry through to the other tabbed views.
    Use the formats in Download to save data for selected sequences. Manage Columns adds and subtracts data columns from the Descriptions table.
    Use the click outs to see the selected results in
    , Graphical Sequence Viewer
    , BLAST Tree View
    , COBALT multiple sequence alignment
    .

  - select all
  - 12 sequences selected

  - Graphics
  - Distance tree of results
  - Multiple alignment

  , Reading indexes 1-5, displaying indexes 1-5


  Load next setPrevious Match


  Sequences producing significant alignments:

  | Select for downloading or viewing reports | Description | Max Score | Total Score | Query Cover | E value | Per. Ident | Accession |
  | --- | --- | --- | --- | --- | --- | --- | --- |
  | 1Select seq lcl|Query\_33745 | KFX30960.1 phosphogluconate dehydrogenase (NADP(+)-dependent, decarboxylating) [Geobacillus icigianus] | 24.6 | 24.6 | 42% | 3.3 | 26.25% | Query\_33745 |
  | 2Select seq lcl|Query\_33645 | KFX31034.1 DUF4145 domain-containing protein [Geobacillus icigianus] | 24.3 | 24.3 | 13% | 5.9 | 32.00% | Query\_33645 |
  | 3Select seq lcl|Query\_33632 | KFX31041.1 glutamine--fructose-6-phosphate transaminase (isomerizing) [Geobacillus icigianus] | 38.5 | 38.5 | 57% | 1e-04 | 23.01% | Query\_33632 |
  | 4Select seq lcl|Query\_33474 | KFX31114.1 flagellar hook-basal body complex protein FliE [Geobacillus icigianus] | 23.5 | 23.5 | 28% | 4.8 | 33.93% | Query\_33474 |
  | 5Select seq lcl|Query\_32047 | KFX33530.1 YdiU family protein [Geobacillus icigianus] | 25.0 | 25.0 | 36% | 2.9 | 27.71% | Query\_32047 |
  | 6Select seq lcl|Query\_31881 | KFX33978.1 D-amino-acid transaminase [Geobacillus icigianus] | 25.8 | 25.8 | 32% | 1.5 | 28.57% | Query\_31881 |
  | 7Select seq lcl|Query\_31764 | KFX34286.1 MurR/RpiR family transcriptional regulator [Geobacillus icigianus] | 45.8 | 45.8 | 59% | 3e-07 | 29.66% | Query\_31764 |
  | 8Select seq lcl|Query\_31742 | KFX34327.1 exodeoxyribonuclease VII large subunit [Geobacillus icigianus] | 24.6 | 24.6 | 25% | 3.8 | 29.17% | Query\_31742 |
  | 9Select seq lcl|Query\_31597 | KFX34703.1 cytosine permease [Geobacillus icigianus] | 27.7 | 27.7 | 40% | 0.37 | 29.07% | Query\_31597 |
  | 10Select seq lcl|Query\_31635 | KFX34742.1 N-acetylmuramic acid 6-phosphate etherase, partial [Geobacillus icigianus] | 29.6 | 29.6 | 31% | 0.057 | 27.12% | Query\_31635 |
  | 11Select seq lcl|Query\_31039 | KFX35732.1 MurR/RpiR family transcriptional regulator [Geobacillus icigianus] | 24.3 | 24.3 | 50% | 4.2 | 25.53% | Query\_31039 |
  | 12Select seq lcl|Query\_30897 | KFX36173.1 mannose-1-phosphate guanylyltransferase [Geobacillus icigianus] | 25.0 | 25.0 | 9% | 2.5 | 41.18% | Query\_30897 |
- Graphic Summary

  - hover to see the title
  - click to show alignments
  - Show Conserved Domains
  - Alignment Scores
  - < 40
  - 40 - 50
  - 50 - 80
  - 80 - 200
  - >= 200
  - Help

    The graphic is an overview of the database sequences aligned to the query sequence. These are represented horizontal bars colored coded by score and showing the extent
    of the alignment on the query sequence. Separate aligned regions on the same database sequence are connected by a thin grey line.
    Mousing over an alignment shows the database sequence title. Clicking an alignment displays a box with more details about the alignment and
    link to the sequence alignment itself in the Alignments section of the report.

  - 12 sequences selected
  - Help

    To select sequences, go to the Descriptions tab

  ### Distribution of the top 12 Blast Hits on 12 subject sequences

  Query

  1

  30

  60

  90

  120

  150

  180

  KFX30960.1 phosphogluconate dehydrogenase (NADP(+)-depe..

  Score:24 Evalue:3.3

  Accession:Query\_33745

  Alignment

  KFX36173.1 mannose-1-phosphate guanylyltransferase [Geo..

  Score:25 Evalue:2.5

  Accession:Query\_30897

  Alignment

  KFX31114.1 flagellar hook-basal body complex protein Fl..

  Score:23 Evalue:4.8

  Accession:Query\_33474

  Alignment

  KFX31034.1 DUF4145 domain-containing protein [Geobacill..

  Score:24 Evalue:5.9

  Accession:Query\_33645

  Alignment

  KFX31041.1 glutamine--fructose-6-phosphate transaminase..

  Score:38 Evalue:9.8e-05

  Accession:Query\_33632

  Alignment

  KFX33530.1 YdiU family protein [Geobacillus icigianus]

  Score:25 Evalue:2.9

  Accession:Query\_32047

  Alignment

  KFX33978.1 D-amino-acid transaminase [Geobacillus icigi..

  Score:25 Evalue:1.5

  Accession:Query\_31881

  Alignment

  KFX34286.1 MurR/RpiR family transcriptional regulator [..

  Score:45 Evalue:2.7e-07

  Accession:Query\_31764

  Alignment

  KFX34327.1 exodeoxyribonuclease VII large subunit [Geob..

  Score:24 Evalue:3.8

  Accession:Query\_31742

  Alignment

  KFX34703.1 cytosine permease [Geobacillus icigianus]

  Score:27 Evalue:0.37

  Accession:Query\_31597

  Alignment

  KFX34742.1 N-acetylmuramic acid 6-phosphate etherase, p..

  Score:29 Evalue:0.057

  Accession:Query\_31635

  Alignment

  KFX35732.1 MurR/RpiR family transcriptional regulator [..

  Score:24 Evalue:4.2

  Accession:Query\_31039

  Alignment
- Alignments

  - Alignment view

    Pairwise
    Pairwise with dots for identities
    Query-anchored with dots for identities
    Query-anchored with letters for identities
    Flat query-anchored with dots for identities
    Flat query-anchored with letters for identities
  - CDS feature
  - Line length:

    60
    90
    120
    150
  - Help

    - Alignment view: Choose how to view alignments.
      The default "pairwise" view shows how each subject sequence aligns
      individually to the query sequence. The "query-anchored" view shows how
      all subject sequences align to the query sequence. For each view type,
      you can choose to show "identities" (matching residues) as letters or dots.
      more...
    - CDS feature: Show annotated coding region and translation.
      more...
    - Line length: Number of letters to show on one line in an alignment.
  - Restore defaults
  - Download
    - FASTA (complete sequence)
    - FASTA (aligned sequences)
    - GenBank (complete sequence)
    - Hit Table (text)
    - Hit Table (CSV)
    - Text
    - XML
    - ASN.1


  - 12 sequences selected
  - Help

    To select sequences, go to the Descriptions tab

  Loading alignment... for sequences lcl|Query\_33745,lcl|Query\_33645,lcl|Query\_33632,lcl|Query\_33474,lcl|Query\_32047 Reading indexes 1-5

  Download

  FASTA (complete sequence)

  FASTA (aligned sequences)

  GenBank (complete sequence)

  Text (aligned sequences)

  Continue
  Cancel

  Graphics

  Next
  Previous
  Descriptions

  KFX30960.1 phosphogluconate dehydrogenase (NADP(+)-dependent, decarboxylating) [Geobacillus icigianus]

  Sequence ID: Query\_33745Length: 469Number of Matches: 1

  Related Information

  Range 1: 41 to 103Graphics

  Next Match
  Previous Match
  First Match

  Alignment statistics for match #1

  | Score | Expect | Method | Identities | Positives | Gaps | Frame |
  | --- | --- | --- | --- | --- | --- | --- |
  | 24.6 bits(52) | 3.3() | Compositional matrix adjust. | 21/80(26%) | 33/80(41%) | 17/80(21%) |  |

  Features:

  ```
  Query  11   EILQELNRTADFIADEEAEKLVNGILQAKKIFVAGAGRSGFMSKSFAMRMMHMGLDAYVV  70
              E LQE   T + +     E+ VN + + +KI +                M+  G      
  Sbjct  41   EFLQEAKGT-NIVGTYSIEEFVNALEKPRKILL----------------MVKAGAPTDTT  83

  Query  71   GETITPNLEQDDILIIGSGS  90
               E + P+LE+ DILI G  +
  Sbjct  84   IEQLKPHLEKGDILIDGGNT  103
  ```

  Download

  FASTA (complete sequence)

  FASTA (aligned sequences)

  GenBank (complete sequence)

  Text (aligned sequences)

  Continue
  Cancel

  Graphics

  Next
  Previous
  Descriptions

  KFX31034.1 DUF4145 domain-containing protein [Geobacillus icigianus]

  Sequence ID: Query\_33645Length: 1113Number of Matches: 1

  Related Information

  Range 1: 361 to 385Graphics

  Next Match
  Previous Match
  First Match

  Alignment statistics for match #1

  | Score | Expect | Method | Identities | Positives | Gaps | Frame |
  | --- | --- | --- | --- | --- | --- | --- |
  | 24.3 bits(51) | 5.9() | Composition-based stats. | 8/25(32%) | 18/25(72%) | 0/25(0%) |  |

  Features:

  ```
  Query  77   NLEQDDILIIGSGSGETRSLVSMAE  101
              N  +  +L++ +GSG+TR+ +S+ +
  Sbjct  361  NKRRKALLVMATGSGKTRTAISIVD  385
  ```

  Download

  FASTA (complete sequence)

  FASTA (aligned sequences)

  GenBank (complete sequence)

  Text (aligned sequences)

  Continue
  Cancel

  Graphics

  Next
  Previous
  Descriptions

  KFX31041.1 glutamine--fructose-6-phosphate transaminase (isomerizing) [Geobacillus icigianus]

  Sequence ID: Query\_33632Length: 600Number of Matches: 1

  Related Information

  Range 1: 281 to 393Graphics

  Next Match
  Previous Match
  First Match

  Alignment statistics for match #1

  | Score | Expect | Method | Identities | Positives | Gaps | Frame |
  | --- | --- | --- | --- | --- | --- | --- |
  | 38.5 bits(88) | 1e-04() | Compositional matrix adjust. | 26/113(23%) | 51/113(45%) | 5/113(4%) |  |

  Features:

  ```
  Query  29   EKLVNGILQAKKIFVAGAGRS---GFMSKSFAMRMMHMGLDAYVVGE--TITPNLEQDDI  83
              + ++N +L A ++++   G S   G + K        + ++ ++  E     P L +  +
  Sbjct  281  QAIINEVLNADRLYIVACGTSYHAGLVGKQLIESWAKIPVEVHIASEFSYNMPLLSEKPL  340

  Query  84   LIIGSGSGETRSLVSMAEKAKSLGATIALVTIFPASTIGKLADITVKLPGSPK  136
               +  S SGET    ++  +   LG     +T  P ST+ + AD T+ L   P+
  Sbjct  341  FLFISQSGETADSRAVLVQTNKLGHKAITITNVPGSTLSREADYTLLLHAGPE  393
  ```

  Download

  FASTA (complete sequence)

  FASTA (aligned sequences)

  GenBank (complete sequence)

  Text (aligned sequences)

  Continue
  Cancel

  Graphics

  Next
  Previous
  Descriptions

  KFX31114.1 flagellar hook-basal body complex protein FliE [Geobacillus icigianus]

  Sequence ID: Query\_33474Length: 98Number of Matches: 1

  Related Information

  Range 1: 37 to 90Graphics

  Next Match
  Previous Match
  First Match

  Alignment statistics for match #1

  | Score | Expect | Method | Identities | Positives | Gaps | Frame |
  | --- | --- | --- | --- | --- | --- | --- |
  | 23.5 bits(49) | 4.8() | Compositional matrix adjust. | 19/56(34%) | 28/56(50%) | 5/56(8%) |  |

  Features:

  ```
  Query  16  LNRT--ADFIADEEAEKLVNG-ILQAKKIFVAGAGRSGFMSKSFAMRMMHMGLDAY  68
             LNR   A   ADE  EKLV G  +Q   + +AG   S  +  +  +R  +  ++AY
  Sbjct  37  LNRVNEAQLQADELTEKLVRGENVQLHDVMIAGQKASIALQLTLEVR--NKAIEAY  90
  ```

  Download

  FASTA (complete sequence)

  FASTA (aligned sequences)

  GenBank (complete sequence)

  Text (aligned sequences)

  Continue
  Cancel

  Graphics

  Next
  Previous
  Descriptions

  KFX33530.1 YdiU family protein [Geobacillus icigianus]

  Sequence ID: Query\_32047Length: 487Number of Matches: 1

  Related Information

  Range 1: 94 to 172Graphics

  Next Match
  Previous Match
  First Match

  Alignment statistics for match #1

  | Score | Expect | Method | Identities | Positives | Gaps | Frame |
  | --- | --- | --- | --- | --- | --- | --- |
  | 25.0 bits(53) | 2.9() | Compositional matrix adjust. | 23/83(28%) | 39/83(46%) | 19/83(22%) |  |

  Features:

  ```
  Query  67   AYVVGETITPNLEQDDILIIGSGS------GETRS---------LVSMAEKAKSLGATIA  111
              A ++GE +TP+ ++ DI + GSG       G+ R+         +VS A  A  +  T +
  Sbjct  94   AVLIGEHVTPSGKRVDIQLKGSGRTPYSRGGDGRAALGPMLREYIVSEAMHALGIPTTRS  153

  Query  112  LVTIFPASTIGKLADITVKLPGS  134
              L  +    TI +      +LPG+
  Sbjct  154  LAVVTTGETIRR----ETELPGA  172
  ```

  ```

  ```
- Taxonomy

  ### Reports

  - 12 sequences selected
  - Help

    To select sequences, go to the Descriptions tab
  - Lineage
  - Organism
  - Taxonomy
- Dot Plot

  ### Plot of lcl|Query\_30600 vs lcl|Query\_33831

   Help

  This dot matrix view shows regions of similarity based upon the BLAST results. The query sequence is represented on the X-axis and the numbers represent the bases/residues of the query. The subject is represented on the Y-axis and again the numbers represent the bases/residues of the subject. Alignments are shown in the plot as lines. Plus strand and protein matches are slanted from the bottom left to the upper right corner, minus strand matches are slanted from the upper left to the lower right. The number of lines shown in the plot is the same as the number of alignments found by BLAST.


Feedback
Top


### Connect

- Twitter
- Facebook
- YouTube
- LinkedIn
- GitHub

- Blog
- Support Center

### National Center for Biotechnology Information

 8600 Rockville Pike
Bethesda  MD, 20894 USA 

- About us
- Contact us
- Polices
- FOIA

#### Popular

- PubMed
- PubMed Central
- Bookshelf
- PubChem
- Gene
- BLAST
- Nucleotide
- Protein
- GEO

#### Resources

- Literature
- Health
- Genomes
- Genes
- Proteins
- Chemicals

#### Actions

- Submit
- Download
- Learn
- Develop
- Analyze
- Research

NLM
 | 
NIH
 | 
HHS
 | 
USA.gov


PreferencesTurn off

External link. Please review our privacy policy.
