# Supplementary material for: A Genome-Scale Metabolic Model of 2,3-Butanediol Production by Thermophilic Bacteria Geobacillus icigianus
Source: Microorganisms. 2020 Jul 4;8(7):1002. doi: 10.3390/microorganisms8071002 (PMC7409357; doi:10.3390/microorganisms8071002)
Supplement: Supplementary file 1 [file microorganisms-08-01002-s001.zip › Geobacillus_icigianus_supplementary/Blast_results/acetolactate_sythase/GStearothermophilus_vs_Gicigianus.html]

NCBI Blast:tr|A4IRI1|A4IRI1\_GEOTN Acetolactate synthase...


 


- NCBI Home
- Sign in to NCBI
- Skip to Main Content
- Skip to Navigation
- About NCBI Accesskeys

National Institutes of Health

U.S. National Library of Medicine

National Center for Biotechnology Information

NCBI homepage

Log in


Show account info

Close

#### Account

Logged in as:  
**username**

- Dashboard (My NCBI)
- Publications (My Bibliography)
- Account settings
- Log out

COVID-19 is an emerging, rapidly evolving situation.  
Get the latest public health information from CDC: https://www.coronavirus.gov .  
Get the latest research from NIH: https://www.nih.gov/coronavirus.  
Find NCBI SARS-CoV-2 literature, sequence, and clinical content: https://www.ncbi.nlm.nih.gov/sars-cov-2/.

BLAST ® » blastp suite »

# results for RID-F34K7TDB016

- Home
- Recent Results
- Saved Strategies
- Help


- Edit Search
- Save Search
- Search Summary

  Search Parameters

  | Search parameter name | Search parameter value |
  | --- | --- |
  | Program | blastp |
  | Word size | 6 |
  | Expect value | 10 |
  | Hitlist size | 100 |
  | Gapcosts | 11,1 |
  | Matrix | BLOSUM62 |
  | Filter string | F |
  | Genetic Code | 1 |
  | Window Size | 40 |
  | Threshold | 21 |
  | Composition-based stats | 2 |

  Database

  | Database parameter name | Database parameter value |
  | --- | --- |
  | Posted date | Jun 18, 2020 12:42 AM |
  | Number of letters | 958,461 |
  | Number of sequences | 3,408 |
  | Entrez query | Includes: Geobacillus icigianus (taxid:1430331)  Excludes:  None |

  Karlin-Altschul statistics

  | Params | Ungapped | Gapped |
  | --- | --- | --- |
  | Lambda | 0.318364 | 0.267 |
  | K | 0.135509 | 0.041 |
  | H | 0.400828 | 0.14 |
  | Alpha | 0.7916 | 1.9 |
  | Alpha\_v | 4.96466 | 42.6028 |
  | Sigma |  | 43.6362 |

  Results Statistics

  | Results Statistics parameter name | Results Statistics parameter value |
  | --- | --- |

- How to read this report?
- BLAST Help Videos
- Back to Traditional Results Page

Your search is limited to records that include: Geobacillus icigianus (taxid:1430331)

- Full Entrez Query

  txid1430331 [ORGN]


Job Title
:   tr|A4IRI1|A4IRI1\_GEOTN Acetolactate synthase...
    ...

    tr|A4IRI1|A4IRI1\_GEOTN Acetolactate synthase...

RID
:   F34K7TDB016
    Search expires on 06-24 13:25 pm

    - Download All
      - Text
      - XML
      - ASN.1
      - JSON Seq-align
      - Hit Table(text)
      - Hit Table(csv)
      - Multiple-file XML2
      - Single-file XML2
      - Multiple-file JSON
      - Single-file JSON
      - SAM

Results for
:   lcl|Query\_31880 tr|A4IRI1|A4IRI1\_GEOTN Acetolactate synthase OS=Geobacillus thermodenitrificans (strain NG80-2) OX=...(576aa)

Program
:   BLASTP
     Help

    Program version: BLASTP 2.10.1+

    - Citation

      Reference 

      Stephen F. Altschul, Thomas L. Madden, Alejandro A. Schäffer, Jinghui Zhang, Zheng Zhang, Webb Miller, and David J. Lipman (1997), "Gapped BLAST and PSI-BLAST: a new generation of protein database search programs", Nucleic Acids Res. 25:3389-3402.

      Reference - compositional score matrix adjustment

      Stephen F. Altschul, John C. Wootton, E. Michael Gertz, Richa Agarwala, Aleksandr Morgulis, Alejandro A. Schäffer, and Yi-Kuo Yu (2005) "Protein database searches using compositionally adjusted substitution matrices", FEBS J. 272:5101-5109.

Database
:   nr

    - See details

      Title:All non-redundant GenBank CDS translations+PDB+SwissProt+PIR+PRF excluding environmental samples from WGS projects  
      Molecule Type:Protein  
      Update date:2020/06/22  
      Number of sequences:291769650

Query ID
:   lcl|Query\_31880
    lcl|Query\_31880

Description
:   tr|A4IRI1|A4IRI1\_GEOTN Acetolactate synthase OS=Geobacillus thermodenitrificans (strain NG80-2) OX=420246 GN=GTNG\_2590 PE=3 SV=1
    ...

    tr|A4IRI1|A4IRI1\_GEOTN Acetolactate synthase OS=Geobacillus thermodenitrificans (strain NG80-2) OX=420246 GN=GTNG\_2590 PE=3 SV=1

Molecule type
:   amino acid

Query Length
:   576

Other reports
:   Distance tree of results
    Multiple alignment
    MSA viewer
     Help

    Reports are generated on using all sequences producing significant alignments. To generate reports on a subset of sequences, use the report links in the Descriptions tab while selecting specific sequences.

## Filter Results

Organism only top 20 will appear


exclude

Add organism


---

Percent Identity from

Percent Identity to

E value from

E value to

Query Coverage from

Query Coverage to

Filter Reset

- Descriptions

  ### Sequences producing significant alignments

  - Download
    - FASTA (complete sequence)
    - FASTA (aligned sequences)
    - GenBank (complete sequence)
    - Hit Table (text)
    - Hit Table (CSV)
    - Text
    - Descriptions Table (CSV)
    - XML
    - ASN.1
  - Manage Columns
    - Description
    - Max Score
    - Total Score
    - Query Coverage
    - E value
    - Percent Identity
    - Accession
    - Restore defaults
  - Show

    10
    50
    100
  - Help

    Subject sequences can be removed or added from within the Descriptions tab and the selections will carry through to the other tabbed views.
    Use the formats in Download to save data for selected sequences. Manage Columns adds and subtracts data columns from the Descriptions table.
    Use the click outs to see the selected results in
    GenPept
    , Graphical Sequence Viewer
    , BLAST Tree View
    , COBALT multiple sequence alignment
    .

  - select all
  - 3 sequences selected
  - GenPept
  - Graphics
  - Distance tree of results
  - Multiple alignment

  , Reading indexes 1-3, displaying indexes 1-3


  Load next setPrevious Match


  Sequences producing significant alignments:

  | Select for downloading or viewing reports | Description | Max Score | Total Score | Query Cover | E value | Per. Ident | Accession |
  | --- | --- | --- | --- | --- | --- | --- | --- |
  | 1Select seq ref|WP\_033020306.1| | MULTISPECIES: acetolactate synthase large subunit [Geobacillus] | 1105 | 1105 | 100% | 0.0 | 92.91% | WP\_033020306.1 |
  | 2Select seq ref|WP\_033018494.1| | MULTISPECIES: thiamine pyrophosphate-binding protein [Geobacillus] | 286 | 286 | 94% | 2e-90 | 33.21% | WP\_033018494.1 |
  | 3Select seq ref|WP\_033019344.1| | MULTISPECIES: acetyl-CoA carboxylase biotin carboxylase subunit [Geobacillus] | 25.8 | 25.8 | 4% | 6.3 | 40.74% | WP\_033019344.1 |
- Graphic Summary

  - hover to see the title
  - click to show alignments
  - Show Conserved Domains
  - Alignment Scores
  - < 40
  - 40 - 50
  - 50 - 80
  - 80 - 200
  - >= 200
  - Help

    The graphic is an overview of the database sequences aligned to the query sequence. These are represented horizontal bars colored coded by score and showing the extent
    of the alignment on the query sequence. Separate aligned regions on the same database sequence are connected by a thin grey line.
    Mousing over an alignment shows the database sequence title. Clicking an alignment displays a box with more details about the alignment and
    link to the sequence alignment itself in the Alignments section of the report.

  - 3 sequences selected
  - Help

    To select sequences, go to the Descriptions tab

  Putative conserved domains have been detected, click on the image below for detailed results.

  ### Distribution of the top 3 Blast Hits on 3 subject sequences

  Query

  1

  100

  200

  300

  400

  500

  MULTISPECIES: acetolactate synthase large subunit [Geob..

  Score:1105 Evalue:0

  Accession:WP\_033020306.1

  Alignment

  MULTISPECIES: thiamine pyrophosphate-binding protein [G..

  Score:286 Evalue:1.9e-90

  Accession:WP\_033018494.1

  Alignment

  MULTISPECIES: acetyl-CoA carboxylase biotin carboxylase..

  Score:25 Evalue:6.3

  Accession:WP\_033019344.1

  Alignment
- Alignments

  - Alignment view

    Pairwise
    Pairwise with dots for identities
    Query-anchored with dots for identities
    Query-anchored with letters for identities
    Flat query-anchored with dots for identities
    Flat query-anchored with letters for identities
  - CDS feature
  - Line length:

    60
    90
    120
    150
  - Help

    - Alignment view: Choose how to view alignments.
      The default "pairwise" view shows how each subject sequence aligns
      individually to the query sequence. The "query-anchored" view shows how
      all subject sequences align to the query sequence. For each view type,
      you can choose to show "identities" (matching residues) as letters or dots.
      more...
    - CDS feature: Show annotated coding region and translation.
      more...
    - Line length: Number of letters to show on one line in an alignment.
  - Restore defaults
  - Download
    - FASTA (complete sequence)
    - FASTA (aligned sequences)
    - GenBank (complete sequence)
    - Hit Table (text)
    - Hit Table (CSV)
    - Text
    - XML
    - ASN.1


  - 3 sequences selected
  - Help

    To select sequences, go to the Descriptions tab

  Loading alignment... for sequences ref|WP\_033020306.1|,ref|WP\_033018494.1|,ref|WP\_033019344.1| Reading indexes 1-3

  Download

  FASTA (complete sequence)

  FASTA (aligned sequences)

  GenBank (complete sequence)

  Text (aligned sequences)

  Continue
  Cancel

  GenPeptGraphics

  Next
  Previous
  Descriptions

  MULTISPECIES: acetolactate synthase large subunit [Geobacillus]

  Sequence ID: WP\_033020306.1Length: 579Number of Matches: 1

  - See 1 more title(s)
    Identical Proteins

    acetolactate synthase large subunit [Geobacillus icigianus]

    Sequence ID: KFX33303.1Length: 579Number of Matches:

  Related Information

  Identical Proteins-Identical proteins to WP\_033020306.1

  Range 1: 1 to 578GenPeptGraphics

  Next Match
  Previous Match
  First Match

  Alignment statistics for match #1

  | Score | Expect | Method | Identities | Positives | Gaps | Frame |
  | --- | --- | --- | --- | --- | --- | --- |
  | 1105 bits(2858) | 0.0() | Compositional matrix adjust. | 537/578(93%) | 557/578(96%) | 2/578(0%) |  |

  Features:

  ```
  Query  1    MAKMKVEEQ--AKTKARMSGSLMLIEALKAENVEVIFGYPGGAVLPLYDELYKAGVFHVL  58
              M +M VEEQ  AKTK +++GSLMLIEALKAE VEVIFGYPGGAVLPLYDELYKAGVFHVL
  Sbjct  1    MTRMNVEEQEKAKTKMKLNGSLMLIEALKAEGVEVIFGYPGGAVLPLYDELYKAGVFHVL  60

  Query  59   TRHEQGAIHAAEGYARISGKPGVVIATSGPGATNIVTGLTDAMMDSLPLVVFTGQVATNV  118
              TRHEQGAIHAAEGYARISGKPGVVIATSGPGATN+VTGLTDAMMDSLPLVVFTGQVAT+V
  Sbjct  61   TRHEQGAIHAAEGYARISGKPGVVIATSGPGATNLVTGLTDAMMDSLPLVVFTGQVATSV  120

  Query  119  IGSDAFQEADVVGITMPITKHNYQVRDISELPKIIKEAFHIATTGRPGPVLIDIPKDVTI  178
              IGSDAFQEADVVGITMPITKHNYQVRDISELP+IIKEAFHIATTGRPGPVLIDIPKD+T 
  Sbjct  121  IGSDAFQEADVVGITMPITKHNYQVRDISELPRIIKEAFHIATTGRPGPVLIDIPKDITT  180

  Query  179  AEGEFDYNQDVHLPGYQPTTQPNHWQIRRLVEAVSQSKRPVILAGAGVLHANAADELQQY  238
              AEGEFDY+QDVHLPGYQPTTQPNHWQIRRLVEAVSQSKRPVILAGAGVLHANAADEL+QY
  Sbjct  181  AEGEFDYDQDVHLPGYQPTTQPNHWQIRRLVEAVSQSKRPVILAGAGVLHANAADELRQY  240

  Query  239  AEQQNIPVAHTLLGLGGFPADHPLFLGMAGMHGTYAANMALYECDLLINIGARFDDRVTG  298
              AEQQ IPV HTLLGLGGFPADHPLFLGMAGMHGTY ANMALYECDLLINIGARFDDRVTG
  Sbjct  241  AEQQRIPVVHTLLGLGGFPADHPLFLGMAGMHGTYTANMALYECDLLINIGARFDDRVTG  300

  Query  299  NLKYFAPKATVAHIDIDPAEIGKNVPTKIPIVSDAKAALQELIEQQGKPADNAAWLAQLN  358
              NL  FAPKATVAHIDIDPAEIGKNVPTKIPIVSDAKAALQELI QQGKPAD AAWL QL+
  Sbjct  301  NLNDFAPKATVAHIDIDPAEIGKNVPTKIPIVSDAKAALQELIAQQGKPADTAAWLVQLD  360

  Query  359  EWKRRFPLHYEPEAGAIKPQKLIEMIYEVTGGEAIVTTDVGQHQMWAAQYYKFNRPNRWV  418
              EWKRRFPL+YEPEAG IKPQKLIEMIYE+T GEAIVTTDVGQHQMWAAQYYKFNRP+RWV
  Sbjct  361  EWKRRFPLYYEPEAGTIKPQKLIEMIYEMTNGEAIVTTDVGQHQMWAAQYYKFNRPHRWV  420

  Query  419  TSGGLGTMGFGLPAAIGAQLADQSATVVSIVGDGGFQMTCQELSVIQELQLPIKVVIVNN  478
              TSGGLGTMGFGLPAAIGAQLAD+SATVVSIVGDGGFQMT QELSVIQELQLPIK+VIVNN
  Sbjct  421  TSGGLGTMGFGLPAAIGAQLADRSATVVSIVGDGGFQMTFQELSVIQELQLPIKIVIVNN  480

  Query  479  QALGMVRQWQELFYDKRYSHSLIPNQPDFVKLAEAYGMPGLRAKTEAEAAEVLKQAFAID  538
              QALGMVRQWQELFYD+RYSHSLIPNQPDFVKLAEAYGM GLRAKTEAEAA+VLKQAFAI+
  Sbjct  481  QALGMVRQWQELFYDQRYSHSLIPNQPDFVKLAEAYGMLGLRAKTEAEAADVLKQAFAIN  540

  Query  539  GPVLLDFHVCADENVYPMVAPGKGLHEMVGVKACEELS  576
              GPVLLDFHV ADENVYPMVAPGKGLH+MVGVKACEE S
  Sbjct  541  GPVLLDFHVRADENVYPMVAPGKGLHQMVGVKACEESS  578
  ```

  Download

  FASTA (complete sequence)

  FASTA (aligned sequences)

  GenBank (complete sequence)

  Text (aligned sequences)

  Continue
  Cancel

  GenPeptGraphics

  Next
  Previous
  Descriptions

  MULTISPECIES: thiamine pyrophosphate-binding protein [Geobacillus]

  Sequence ID: WP\_033018494.1Length: 551Number of Matches: 1

  - See 1 more title(s)
    Identical Proteins

    acetolactate synthase [Geobacillus icigianus]

    Sequence ID: KFX35671.1Length: 551Number of Matches:

  Related Information

  Identical Proteins-Identical proteins to WP\_033018494.1

  Range 1: 3 to 535GenPeptGraphics

  Next Match
  Previous Match
  First Match

  Alignment statistics for match #1

  | Score | Expect | Method | Identities | Positives | Gaps | Frame |
  | --- | --- | --- | --- | --- | --- | --- |
  | 286 bits(731) | 2e-90() | Compositional matrix adjust. | 184/554(33%) | 280/554(50%) | 32/554(5%) |  |

  Features:

  ```
  Query  11   KTKARMSGSLMLIEALKAENVEVIFGYPGGAVLPLYDELYKAGVFHVLT-RHEQGAIHAA  69
              +T   ++ + +++E LK E +  +FG PG + LPL D +Y+      ++ RHE GA   A
  Sbjct  3    RTIRNVTVAKVIVECLKQEQIRHVFGVPGESYLPLLDAIYEEPSIEFISARHEGGASFMA  62

  Query  70   EGYARISGKPGVVIATSGPGATNIVTGLTDAMMDSLPLVVFTGQVATNVIGSDAFQEADV  129
              EGYA+ +   GVV+AT   GA N+  G+  A  DS P+VVF GQV +  +G + FQE D+
  Sbjct  63   EGYAKAARTCGVVLATRAVGAANLAIGVHTARQDSTPMVVFLGQVDSRFLGREGFQEVDL  122

  Query  130  VGITMPITKHNYQVRDISELPKIIKEAFHIATTGRPGPVLIDIPKDV---TIAEGEFDYN  186
                   P+ K   ++RD   +P++++ AF  A TGRPGPV++ +P+DV   T+ E      
  Sbjct  123  EAFFRPLAKWTVEIRDAERVPELVQRAFRTAKTGRPGPVVVSLPEDVLWQTVPEAVMAST  182

  Query  187  QDVHLPGYQPTTQPNHWQIRRLVEAVSQSKRPVILAGAGVLHANAADELQQYAEQQNIPV  246
              Q        P   P H  +R +   ++++KRP+++AG GV  A A   L+ +AE  ++PV
  Sbjct  183  Q-------VPKPAPRHEDVREVEAWLTRAKRPLVIAGGGVKWAGAEPLLRLWAETYSLPV  235

  Query  247  AHTLLGLGGFPADHPLFLGMAGMHGTYAANMALYECDLLINIGARFDDRVTGNLKYFAPK  306
                       FP DHP ++G  G+    A      + D++I +G R  +  T +  Y  P 
  Sbjct  236  MAAFRRHDVFPHDHPCYVGHLGLGAPEAVRETAEQADVVIALGTRLSEVTTQD--YCVPS  293

  Query  307  A--TVAHIDIDPAEIGKNVPTKIPIVSDAKAALQELIEQQGKPADNAAWLAQLNEWKRRF  364
              A  T+ HID+D    GK     + I +D + AL  L+    +P    +W     EW  + 
  Sbjct  294  ANQTLIHIDLDSDGFGKVRAPDVAIWADCREALSRLLTIAVRP----SW----QEWVAKR  345

  Query  365  PLHYEPEAG-AIKPQKLIEMIYEVTG----GEAIVTTDVGQHQMWAAQYYKFNRPNRWVT  419
                 YE  A    KP+ + E             A+ T D G    W   ++ F   + ++ 
  Sbjct  346  REQYEQTATLPEKPRNVYEAAMASFARHLPNNAVFTNDAGNFAGWLHTFFPFGEGHTYIG  405

  Query  420  SGGLGTMGFGLPAAIGAQLADQSATVVSIVGDGGFQMTCQELSVIQELQLPIKVVIVNNQ  479
                  G MG+G+PAAIGA+LA    TVVS+ GDGGF MT QEL       +PI  V+ NN+
  Sbjct  406  PTS-GAMGYGMPAAIGAKLALPDRTVVSLSGDGGFMMTMQELETAVRYDIPIISVVFNNR  464

  Query  480  ALGMVRQWQELFYDKRYSHSLIPNQPDFVKLAEAYGMPGLRAKTEAEAAEVLKQAFAIDG  539
                G +R  QEL +  R   + + + P F +LAE     G + +TE +  E L  A A   
  Sbjct  465  MYGTIRMHQELRFPGRVIGTELGSVP-FARLAECLNGLGFQVQTEQQFTEALCAALAAKR  523

  Query  540  PVLLDFHVCADENV  553
              P +++  V AD + 
  Sbjct  524  PTVIE--VLADPDC  535
  ```

  Download

  FASTA (complete sequence)

  FASTA (aligned sequences)

  GenBank (complete sequence)

  Text (aligned sequences)

  Continue
  Cancel

  GenPeptGraphics

  Next
  Previous
  Descriptions

  MULTISPECIES: acetyl-CoA carboxylase biotin carboxylase subunit [Geobacillus]

  Sequence ID: WP\_033019344.1Length: 451Number of Matches: 1

  - See 1 more title(s)
    Identical Proteins

    acetyl-CoA carboxylase biotin carboxylase subunit [Geobacillus icigianus]

    Sequence ID: KFX34323.1Length: 451Number of Matches:

  Related Information

  Identical Proteins-Identical proteins to WP\_033019344.1

  Range 1: 373 to 399GenPeptGraphics

  Next Match
  Previous Match
  First Match

  Alignment statistics for match #1

  | Score | Expect | Method | Identities | Positives | Gaps | Frame |
  | --- | --- | --- | --- | --- | --- | --- |
  | 25.8 bits(55) | 6.3() | Compositional matrix adjust. | 11/27(41%) | 14/27(51%) | 0/27(0%) |  |

  Features:

  ```
  Query  36   GYPGGAVLPLYDELYKAGVFHVLTRHE  62
               YPG A+ P YD +    + H  TR E
  Sbjct  373  AYPGYAIPPYYDSMIAKLIVHAPTRAE  399
  ```

  ```

  ```
- Taxonomy

  ### Reports

  - 3 sequences selected
  - Help

    To select sequences, go to the Descriptions tab
  - Lineage

    Lineage Report

    | Organism | Blast Name | Score | Number of Hits | Description |
    | --- | --- | --- | --- | --- |
    | Bacillaceae | firmicutes |  | 6 |  |
    | .Geobacillus | firmicutes | 1105 | 3 | Geobacillus hits |
    | .Geobacillus icigianus | firmicutes | 1105 | 3 | Geobacillus icigianus hits |
  - Organism

    Organism Report

    | Description | Score | E value | Accession |
    | --- | --- | --- | --- |
    | Geobacillus [firmicutes]  Next Previous First | | | |
    | --- | --- | --- | --- |
    | MULTISPECIES: acetolactate synthase large subunit [Geobacillus] | 1105 | 0.0 | WP\_033020306 |
    | MULTISPECIES: thiamine pyrophosphate-binding protein [Geobacillus] | 286 | 2e-90 | WP\_033018494 |
    | MULTISPECIES: acetyl-CoA carboxylase biotin carboxylase subunit [Geobacillus] | 25.8 | 6.3 | WP\_033019344 |
    | Geobacillus icigianus [firmicutes]  Next Previous First | | | |
    | acetolactate synthase large subunit [Geobacillus icigianus] | 1105 | 0.0 | KFX33303 |
    | acetolactate synthase [Geobacillus icigianus] | 286 | 2e-90 | KFX35671 |
    | acetyl-CoA carboxylase biotin carboxylase subunit [Geobacillus icigianus] | 25.8 | 6.3 | KFX34323 |
  - Taxonomy

    Taxonomy Report

    | Taxonomy | Number of hits | Number of Organisms | Description |
    | --- | --- | --- | --- |
    | Bacillaceae | 6 | 2 |  |
    | .  Geobacillus | 3 | 2 | Geobacillus hits |
    | ..  Geobacillus icigianus | 3 | 1 | Geobacillus icigianus hits |


Feedback
Top


### Connect

- Twitter
- Facebook
- YouTube
- LinkedIn
- GitHub

- Blog
- Support Center

### National Center for Biotechnology Information

 8600 Rockville Pike
Bethesda  MD, 20894 USA 

- About us
- Contact us
- Polices
- FOIA

#### Popular

- PubMed
- PubMed Central
- Bookshelf
- PubChem
- Gene
- BLAST
- Nucleotide
- Protein
- GEO

#### Resources

- Literature
- Health
- Genomes
- Genes
- Proteins
- Chemicals

#### Actions

- Submit
- Download
- Learn
- Develop
- Analyze
- Research

NLM
 | 
NIH
 | 
HHS
 | 
USA.gov


PreferencesTurn off

External link. Please review our privacy policy.

|  |  |  |  |  |  |  |  |  |  |
| --- | --- | --- | --- | --- | --- | --- | --- | --- | --- |
|  | Определить языкАзербайджанскийАлбанскийАмхарскийАнглийскийАрабскийАрмянскийАфрикаансБаскскийБелорусскийБенгальскийБирманскийБолгарскийБоснийскийВаллийскийВенгерскийВьетнамскийГавайскийГаитянскийГалисийскийГолландскийГреческийГрузинскийГуджаратиДатскийЗулуИвритИгбоИдишИндонезийскийИрландскийИсландскийИспанскийИтальянскийЙорубаКазахскийКаннадаКаталанскийКиргизскийКитайский ТрадКитайский УпрКорейскийКорсиканскийКурманджиКхмерскийКхосаЛаосскийЛатинскийЛатышскийЛитовскийЛюксембургскийМакедонскийМалагасийскийМалайскийМалаяламМальтийскийМаориМаратхиМонгольскийНемецкийНепальскийНорвежскийПанджабиПерсидскийПольскийПортугальскийПуштуРумынскийРусскийСамоанскийСебуанскийСербскийСесотоСингальскийСиндхиСловацкийСловенскийСомалийскийСуахилиСунданскийТаджикскийТайскийТамильскийТелугуТурецкийУзбекскийУкраинскийУрдуФилиппинскийФинскийФранцузскийФризскийХаусаХиндиХмонгХорватскийЧеваЧешскийШведскийШонаШотландский (гэльский)ЭсперантоЭстонскийЯванскийЯпонский |  | АзербайджанскийАлбанскийАмхарскийАнглийскийАрабскийАрмянскийАфрикаансБаскскийБелорусскийБенгальскийБирманскийБолгарскийБоснийскийВаллийскийВенгерскийВьетнамскийГавайскийГаитянскийГалисийскийГолландскийГреческийГрузинскийГуджаратиДатскийЗулуИвритИгбоИдишИндонезийскийИрландскийИсландскийИспанскийИтальянскийЙорубаКазахскийКаннадаКаталанскийКиргизскийКитайский ТрадКитайский УпрКорейскийКорсиканскийКурманджиКхмерскийКхосаЛаосскийЛатинскийЛатышскийЛитовскийЛюксембургскийМакедонскийМалагасийскийМалайскийМалаяламМальтийскийМаориМаратхиМонгольскийНемецкийНепальскийНорвежскийПанджабиПерсидскийПольскийПортугальскийПуштуРумынскийРусскийСамоанскийСебуанскийСербскийСесотоСингальскийСиндхиСловацкийСловенскийСомалийскийСуахилиСунданскийТаджикскийТайскийТамильскийТелугуТурецкийУзбекскийУкраинскийУрдуФилиппинскийФинскийФранцузскийФризскийХаусаХиндиХмонгХорватскийЧеваЧешскийШведскийШонаШотландский (гэльский)ЭсперантоЭстонскийЯванскийЯпонский |  |  |  |  |  |  |

Звуковая функция ограничена 200 символами

|  |  |  |  |
| --- | --- | --- | --- |
|  |  | Настройки : История : Обратная связь : Donate | Закрыть |
