# Supplementary material for: A Genome-Scale Metabolic Model of 2,3-Butanediol Production by Thermophilic Bacteria Geobacillus icigianus
Source: Microorganisms. 2020 Jul 4;8(7):1002. doi: 10.3390/microorganisms8071002 (PMC7409357; doi:10.3390/microorganisms8071002)
Supplement: Supplementary file 1 [file microorganisms-08-01002-s001.zip › Geobacillus_icigianus_supplementary/Blast_results/RR_butandeiol_dehydrohenase/BSubtilis_vs_Gicigianus.html]

NCBI Blast:sp|O34788|BDHA\_BACSU (R,R)-butanediol dehydrogenase...


 


- NCBI Home
- Sign in to NCBI
- Skip to Main Content
- Skip to Navigation
- About NCBI Accesskeys

National Institutes of Health

U.S. National Library of Medicine

National Center for Biotechnology Information

NCBI homepage

Log in


Show account info

Close

#### Account

Logged in as:  
**username**

- Dashboard (My NCBI)
- Publications (My Bibliography)
- Account settings
- Log out

COVID-19 is an emerging, rapidly evolving situation.  
Get the latest public health information from CDC: https://www.coronavirus.gov .  
Get the latest research from NIH: https://www.nih.gov/coronavirus.  
Find NCBI SARS-CoV-2 literature, sequence, and clinical content: https://www.ncbi.nlm.nih.gov/sars-cov-2/.

BLAST ^®^ » blastp suite »

# results for RID-F350MJBK014

- Home
- Recent Results
- Saved Strategies
- Help


- Edit Search
- Save Search
- Search Summary

  Search Parameters

  | Search parameter name | Search parameter value |
  | --- | --- |
  | Program | blastp |
  | Word size | 6 |
  | Expect value | 10 |
  | Hitlist size | 100 |
  | Gapcosts | 11,1 |
  | Matrix | BLOSUM62 |
  | Filter string | F |
  | Genetic Code | 1 |
  | Window Size | 40 |
  | Threshold | 21 |
  | Composition-based stats | 2 |

  Database

  | Database parameter name | Database parameter value |
  | --- | --- |
  | Posted date | Jun 18, 2020 12:42 AM |
  | Number of letters | 958,461 |
  | Number of sequences | 3,408 |
  | Entrez query | Includes: Geobacillus icigianus (taxid:1430331)  Excludes:  None |

  Karlin-Altschul statistics

  | Params | Ungapped | Gapped |
  | --- | --- | --- |
  | Lambda | 0.316043 | 0.267 |
  | K | 0.137705 | 0.041 |
  | H | 0.397313 | 0.14 |
  | Alpha | 0.7916 | 1.9 |
  | Alpha\_v | 4.96466 | 42.6028 |
  | Sigma |  | 43.6362 |

  Results Statistics

  | Results Statistics parameter name | Results Statistics parameter value |
  | --- | --- |

- How to read this report?
- BLAST Help Videos
- Back to Traditional Results Page

Your search is limited to records that include: Geobacillus icigianus (taxid:1430331)

- Full Entrez Query

  txid1430331 [ORGN]


Job Title
:   sp|O34788|BDHA\_BACSU (R,R)-butanediol dehydrogenase...
    ...

    sp|O34788|BDHA\_BACSU (R,R)-butanediol dehydrogenase...

RID
:   F350MJBK014
    Search expires on 06-24 13:33 pm

    - Download All
      - Text
      - XML
      - ASN.1
      - JSON Seq-align
      - Hit Table(text)
      - Hit Table(csv)
      - Multiple-file XML2
      - Single-file XML2
      - Multiple-file JSON
      - Single-file JSON
      - SAM

Results for
:   lcl|Query\_77375 sp|O34788|BDHA\_BACSU (R,R)-butanediol dehydrogenase OS=Bacillus subtilis (strain 168) OX=224308 GN=...(346aa)

Program
:   BLASTP
     Help

    Program version: BLASTP 2.10.1+

    - Citation

      Reference 

      Stephen F. Altschul, Thomas L. Madden, Alejandro A. Schäffer, Jinghui Zhang, Zheng Zhang, Webb Miller, and David J. Lipman (1997), "Gapped BLAST and PSI-BLAST: a new generation of protein database search programs", Nucleic Acids Res. 25:3389-3402.

      Reference - compositional score matrix adjustment

      Stephen F. Altschul, John C. Wootton, E. Michael Gertz, Richa Agarwala, Aleksandr Morgulis, Alejandro A. Schäffer, and Yi-Kuo Yu (2005) "Protein database searches using compositionally adjusted substitution matrices", FEBS J. 272:5101-5109.

Database
:   nr

    - See details

      Title:All non-redundant GenBank CDS translations+PDB+SwissProt+PIR+PRF excluding environmental samples from WGS projects  
      Molecule Type:Protein  
      Update date:2020/06/22  
      Number of sequences:291769650

Query ID
:   lcl|Query\_77375
    lcl|Query\_77375

Description
:   sp|O34788|BDHA\_BACSU (R,R)-butanediol dehydrogenase OS=Bacillus subtilis (strain 168) OX=224308 GN=bdhA PE=1 SV=1
    ...

    sp|O34788|BDHA\_BACSU (R,R)-butanediol dehydrogenase OS=Bacillus subtilis (strain 168) OX=224308 GN=bdhA PE=1 SV=1

Molecule type
:   amino acid

Query Length
:   346

Other reports
:   Distance tree of results
    Multiple alignment
    MSA viewer
     Help

    Reports are generated on using all sequences producing significant alignments. To generate reports on a subset of sequences, use the report links in the Descriptions tab while selecting specific sequences.

## Filter Results

Organism only top 20 will appear


exclude

Add organism


---

Percent Identity from

Percent Identity to

E value from

E value to

Query Coverage from

Query Coverage to

Filter Reset

- Descriptions

  ### Sequences producing significant alignments

  - Download
    - FASTA (complete sequence)
    - FASTA (aligned sequences)
    - GenBank (complete sequence)
    - Hit Table (text)
    - Hit Table (CSV)
    - Text
    - Descriptions Table (CSV)
    - XML
    - ASN.1
  - Manage Columns
    - Description
    - Max Score
    - Total Score
    - Query Coverage
    - E value
    - Percent Identity
    - Accession
    - Restore defaults
  - Show

    10
    50
    100
  - Help

    Subject sequences can be removed or added from within the Descriptions tab and the selections will carry through to the other tabbed views.
    Use the formats in Download to save data for selected sequences. Manage Columns adds and subtracts data columns from the Descriptions table.
    Use the click outs to see the selected results in
    GenPept
    , Graphical Sequence Viewer
    , BLAST Tree View
    , COBALT multiple sequence alignment
    .

  - select all
  - 2 sequences selected
  - GenPept
  - Graphics
  - Distance tree of results
  - Multiple alignment

  , Reading indexes 1-2, displaying indexes 1-2


  Load next setPrevious Match


  Sequences producing significant alignments:

  | Select for downloading or viewing reports | Description | Max Score | Total Score | Query Cover | E value | Per. Ident | Accession |
  | --- | --- | --- | --- | --- | --- | --- | --- |
  | 1Select seq ref\|WP\_051985134.1\| | Zn-dependent alcohol dehydrogenase [Geobacillus icigianus] | 99.8 | 99.8 | 99% | 1e-24 | 27.78% | WP\_051985134.1 |
  | 2Select seq ref\|WP\_033020614.1\| | MULTISPECIES: NADPH:quinone oxidoreductase family protein [Geobacillus] | 59.7 | 59.7 | 67% | 5e-11 | 25.52% | WP\_033020614.1 |
- Graphic Summary

  - hover to see the title
  - click to show alignments
  - Show Conserved Domains
  - Alignment Scores
  - < 40
  - 40 - 50
  - 50 - 80
  - 80 - 200
  - >= 200
  - Help

    The graphic is an overview of the database sequences aligned to the query sequence. These are represented horizontal bars colored coded by score and showing the extent
    of the alignment on the query sequence. Separate aligned regions on the same database sequence are connected by a thin grey line.
    Mousing over an alignment shows the database sequence title. Clicking an alignment displays a box with more details about the alignment and
    link to the sequence alignment itself in the Alignments section of the report.

  - 2 sequences selected
  - Help

    To select sequences, go to the Descriptions tab

  Putative conserved domains have been detected, click on the image below for detailed results.

  ### Distribution of the top 2 Blast Hits on 2 subject sequences

  Query

  1

  60

  120

  180

  240

  300

  Zn-dependent alcohol dehydrogenase [Geobacillus icigian..

  Score:99 Evalue:1.3e-24

  Accession:WP\_051985134.1

  Alignment

  MULTISPECIES: NADPH:quinone oxidoreductase family prote..

  Score:59 Evalue:4.8e-11

  Accession:WP\_033020614.1

  Alignment
- Alignments

  - Alignment view

    Pairwise
    Pairwise with dots for identities
    Query-anchored with dots for identities
    Query-anchored with letters for identities
    Flat query-anchored with dots for identities
    Flat query-anchored with letters for identities
  - CDS feature
  - Line length:

    60
    90
    120
    150
  - Help

    - Alignment view: Choose how to view alignments.
      The default "pairwise" view shows how each subject sequence aligns
      individually to the query sequence. The "query-anchored" view shows how
      all subject sequences align to the query sequence. For each view type,
      you can choose to show "identities" (matching residues) as letters or dots.
      more...
    - CDS feature: Show annotated coding region and translation.
      more...
    - Line length: Number of letters to show on one line in an alignment.
  - Restore defaults
  - Download
    - FASTA (complete sequence)
    - FASTA (aligned sequences)
    - GenBank (complete sequence)
    - Hit Table (text)
    - Hit Table (CSV)
    - Text
    - XML
    - ASN.1


  - 2 sequences selected
  - Help

    To select sequences, go to the Descriptions tab

  Loading alignment... for sequences ref|WP\_051985134.1|,ref|WP\_033020614.1| Reading indexes 1-2

  Download

  FASTA (complete sequence)

  FASTA (aligned sequences)

  GenBank (complete sequence)

  Text (aligned sequences)

  Continue
  Cancel

  GenPeptGraphics

  Next
  Previous
  Descriptions

  Zn-dependent alcohol dehydrogenase [Geobacillus icigianus]

  Sequence ID: WP\_051985134.1Length: 368Number of Matches: 1

  - See 1 more title(s)
    Identical Proteins

    alcohol dehydrogenase [Geobacillus icigianus]

    Sequence ID: KFX31005.2Length: 368Number of Matches:

  Related Information

  Identical Proteins-Identical proteins to WP\_051985134.1

  Range 1: 2 to 365GenPeptGraphics

  Next Match
  Previous Match
  First Match

  Alignment statistics for match #1

  | Score | Expect | Method | Identities | Positives | Gaps | Frame |
  | --- | --- | --- | --- | --- | --- | --- |
  | 99.8 bits(247) | 1e-24() | Compositional matrix adjust. | 105/378(28%) | 171/378(45%) | 48/378(12%) |  |

  Features:

  ```
  Query  1    MKAARWHNQKD-IRIEHIEEPKTEPGKVKIKVKWCGICGSDLHEYLGGPIFIPVDKPHPL  59
              +KAA  H+  + + IE ++      G+VK+K+K  G+C SD H   G             
  Sbjct  2    VKAAVLHHYGEPLAIEELDLAAPRFGEVKVKMKAAGLCHSDWHVVQG------------Q  49

  Query  60   TNETAPVTMGHEFSGEVVEVGEGVENYKVGDRVVVEPIFAT----HGHQGAYNLDEQMGF  115
                   P+ +GHE +G V EVGEGV + + GD VV+  + +     +   G  +L E    
  Sbjct  50   LPLPLPLVLGHEGAGIVEEVGEGVTSVQAGDHVVLNWVPSCGRCPYCRLGRPDLCEAAAR  109

  Query  116  LGLAG-----------GG---------GGFSEYVSVDEELLFKLPDELSYEQGALVEPSA  155
              L   G           GG         G FSE   V E+    +  ++ +E  AL+  S 
  Sbjct  110  LTATGTLPDGTTRFSLGGKPVYQFLTAGAFSERTIVPEQAAIPIRRDVPFELAALISCSV  169

  Query  156  VALYA--VRSSKLKAGDKAAVFGCGPIGLLVIEALKAAGATDIYAVELSPERQQKAEELG  213
              +      + +++++ G   AV G G +G  +I+    AGA  I A+++  E+    +  G
  Sbjct  170  MTGVGAVIHAARVRPGSTVAVIGAGGVGFNIIQGAALAGAKQIIAIDIVEEKLTMTKTFG  229

  Query  214  AIIVDPSKTDDVVAEIAERTGG-GVDVAFEVTGVPVVLRQAIQSTTIAGETVIVSIW--E  270
              A     +  +D V+ + + T G GVD AFE  G P  +  A   T  AG TVIV I    
  Sbjct  230  ATHTVNAGDEDAVSAVLDLTDGLGVDYAFEAIGRPETMADAYNMTRKAGMTVIVGIAAPH  289

  Query  271  KGAEIHPNDIVIKERTVKGI-IGYRDI---FPAVLSLMKEGYFSADKLVTKKIVLDDLIE  326
              +  EI+   +  + +T+ G+ +G  +    +P +L L   G    + L++    L+  I 
  Sbjct  290  ETVEINAFSLPSQAKTLTGVWLGQGNPPVDYPKLLDLYAAGKLKLEPLISAVYALEQ-IN  348

  Query  327  EGFGALIKEKSQVKILVR  344
              +GF AL K    ++ L+R
  Sbjct  349  DGFAAL-KSGQNIRGLIR  365
  ```

  Download

  FASTA (complete sequence)

  FASTA (aligned sequences)

  GenBank (complete sequence)

  Text (aligned sequences)

  Continue
  Cancel

  GenPeptGraphics

  Next
  Previous
  Descriptions

  MULTISPECIES: NADPH:quinone oxidoreductase family protein [Geobacillus]

  Sequence ID: WP\_033020614.1Length: 327Number of Matches: 1

  - See 1 more title(s)
    Identical Proteins

    NADPH:quinone oxidoreductase family protein [Geobacillus icigianus]

    Sequence ID: KFX33000.1Length: 327Number of Matches:

  Related Information

  Identical Proteins-Identical proteins to WP\_033020614.1

  Range 1: 15 to 215GenPeptGraphics

  Next Match
  Previous Match
  First Match

  Alignment statistics for match #1

  | Score | Expect | Method | Identities | Positives | Gaps | Frame |
  | --- | --- | --- | --- | --- | --- | --- |
  | 59.7 bits(143) | 5e-11() | Compositional matrix adjust. | 61/239(26%) | 105/239(43%) | 43/239(17%) |  |

  Features:

  ```
  Query  12   IRIEHIEEPKTEPGKVKIKVKWCGICGSDLHEYLGGPIFIPVDKPHPLTNETAPVTMGHE  71
              ++++ IEEPK +P +V I V    +  +D+    G                  P+ +G +
  Sbjct  15   LKLKDIEEPKPKPNEVIIAVSKTSLNFADIKNRTGKK-----------AKGEFPLILGLD  63

  Query  72   FSGEVVEVGEGVENYKVGDRVVVEPIFATHGHQGAYNLDEQMGFLGLAGGGGGFSEYVSV  131
               +G VVEVGE V++  VG RV+  P                          G ++EYV  
  Sbjct  64   AAGIVVEVGEQVKSISVGQRVIAFP------------------------SNGSYTEYVVA  99

  Query  132  DEELLFKLPDELSYEQGALVEPSAVALYAVRS--SKLKAGDKAAVF-GCGPIGLLVIEAL  188
              DE+L+F +P+ +     A     +   YA+    ++++ G+   +    G +G   I+  
  Sbjct  100  DEKLVFPIPNNMDMLTAAACPIVSFLSYALLKYLARIQEGESVLIHSASGGVGTTAIQLA  159

  Query  189  KAAGATDIYAVELSPERQQKAEELGAIIVDPSKT-DDVVAEIAERTGG-GVDVAFEVTG  245
              +  GA  I+    + E++  A + GA   D   T ++ V E+ ERT G GV+V  +  G
  Sbjct  160  RIFGAKQIFGSVGNIEKEFIAIDAGA---DEVFTYENFVREVKERTDGQGVNVILDSVG  215
  ```
- Taxonomy

  ### Reports

  - 2 sequences selected
  - Help

    To select sequences, go to the Descriptions tab
  - Lineage

    Lineage Report

    | Organism | Blast Name | Score | Number of Hits | Description |
    | --- | --- | --- | --- | --- |
    | Bacillaceae | firmicutes |  | 4 |  |
    | .Geobacillus | firmicutes |  | 1 |  |
    | ..Geobacillus icigianus | firmicutes | 99.8 | 3 | Geobacillus icigianus hits |
    | .Geobacillus | firmicutes | 59.7 | 1 | Geobacillus hits |
  - Organism

    Organism Report

    | Description | Score | E value | Accession |
    | --- | --- | --- | --- |
    | Geobacillus icigianus [firmicutes]  Next Previous First | | | |
    | --- | --- | --- | --- |
    | Zn-dependent alcohol dehydrogenase [Geobacillus icigianus] | 99.8 | 1e-24 | WP\_051985134 |
    | alcohol dehydrogenase [Geobacillus icigianus] | 99.8 | 1e-24 | KFX31005 |
    | NADPH:quinone oxidoreductase family protein [Geobacillus icigianus] | 59.7 | 5e-11 | KFX33000 |
    | Geobacillus [firmicutes]  Next Previous First | | | |
    | MULTISPECIES: NADPH:quinone oxidoreductase family protein [Geobacillus] | 59.7 | 5e-11 | WP\_033020614 |
  - Taxonomy

    Taxonomy Report

    | Taxonomy | Number of hits | Number of Organisms | Description |
    | --- | --- | --- | --- |
    | Bacillaceae | 4 | 2 |  |
    | .  Geobacillus | 1 | 2 | Geobacillus hits |
    | ..  Geobacillus icigianus | 3 | 1 | Geobacillus icigianus hits |


Feedback
Top


### Connect

- Twitter
- Facebook
- YouTube
- LinkedIn
- GitHub

- Blog
- Support Center

### National Center for Biotechnology Information

 8600 Rockville Pike
Bethesda  MD, 20894 USA 

- About us
- Contact us
- Polices
- FOIA

#### Popular

- PubMed
- PubMed Central
- Bookshelf
- PubChem
- Gene
- BLAST
- Nucleotide
- Protein
- GEO

#### Resources

- Literature
- Health
- Genomes
- Genes
- Proteins
- Chemicals

#### Actions

- Submit
- Download
- Learn
- Develop
- Analyze
- Research

NLM
 | 
NIH
 | 
HHS
 | 
USA.gov


PreferencesTurn off

External link. Please review our privacy policy.

|  |  |  |  |  |  |  |  |  |  |
| --- | --- | --- | --- | --- | --- | --- | --- | --- | --- |
|  | Определить языкАзербайджанскийАлбанскийАмхарскийАнглийскийАрабскийАрмянскийАфрикаансБаскскийБелорусскийБенгальскийБирманскийБолгарскийБоснийскийВаллийскийВенгерскийВьетнамскийГавайскийГаитянскийГалисийскийГолландскийГреческийГрузинскийГуджаратиДатскийЗулуИвритИгбоИдишИндонезийскийИрландскийИсландскийИспанскийИтальянскийЙорубаКазахскийКаннадаКаталанскийКиргизскийКитайский ТрадКитайский УпрКорейскийКорсиканскийКурманджиКхмерскийКхосаЛаосскийЛатинскийЛатышскийЛитовскийЛюксембургскийМакедонскийМалагасийскийМалайскийМалаяламМальтийскийМаориМаратхиМонгольскийНемецкийНепальскийНорвежскийПанджабиПерсидскийПольскийПортугальскийПуштуРумынскийРусскийСамоанскийСебуанскийСербскийСесотоСингальскийСиндхиСловацкийСловенскийСомалийскийСуахилиСунданскийТаджикскийТайскийТамильскийТелугуТурецкийУзбекскийУкраинскийУрдуФилиппинскийФинскийФранцузскийФризскийХаусаХиндиХмонгХорватскийЧеваЧешскийШведскийШонаШотландский (гэльский)ЭсперантоЭстонскийЯванскийЯпонский |  | АзербайджанскийАлбанскийАмхарскийАнглийскийАрабскийАрмянскийАфрикаансБаскскийБелорусскийБенгальскийБирманскийБолгарскийБоснийскийВаллийскийВенгерскийВьетнамскийГавайскийГаитянскийГалисийскийГолландскийГреческийГрузинскийГуджаратиДатскийЗулуИвритИгбоИдишИндонезийскийИрландскийИсландскийИспанскийИтальянскийЙорубаКазахскийКаннадаКаталанскийКиргизскийКитайский ТрадКитайский УпрКорейскийКорсиканскийКурманджиКхмерскийКхосаЛаосскийЛатинскийЛатышскийЛитовскийЛюксембургскийМакедонскийМалагасийскийМалайскийМалаяламМальтийскийМаориМаратхиМонгольскийНемецкийНепальскийНорвежскийПанджабиПерсидскийПольскийПортугальскийПуштуРумынскийРусскийСамоанскийСебуанскийСербскийСесотоСингальскийСиндхиСловацкийСловенскийСомалийскийСуахилиСунданскийТаджикскийТайскийТамильскийТелугуТурецкийУзбекскийУкраинскийУрдуФилиппинскийФинскийФранцузскийФризскийХаусаХиндиХмонгХорватскийЧеваЧешскийШведскийШонаШотландский (гэльский)ЭсперантоЭстонскийЯванскийЯпонский |  |  |  |  |  |  |

Звуковая функция ограничена 200 символами

|  |  |  |  |
| --- | --- | --- | --- |
|  |  | Настройки : История : Обратная связь : Donate | Закрыть |
