# Supplementary material for: A Genome-Scale Metabolic Model of 2,3-Butanediol Production by Thermophilic Bacteria Geobacillus icigianus
Source: Microorganisms. 2020 Jul 4;8(7):1002. doi: 10.3390/microorganisms8071002 (PMC7409357; doi:10.3390/microorganisms8071002)
Supplement: Supplementary file 1 [file microorganisms-08-01002-s001.zip › Geobacillus_icigianus_supplementary/model_files/Memote_results/MemoteReportFinalModel.html]

MemoteReportApp


 iMK1321  Expand All

Readme

2020-06-23 13:39

Independent Section  Contains tests that are independent of the class of modeled organism, a model's complexity or types of identifiers that are used to describe its components. Parameterization or initialization of the network is not required. See readme for more details. 

## Consistency

Stoichiometric Consistency |

100.0% |

X3

Mass Balance |

100.0% |

Charge Balance |

95.6% |

Metabolite Connectivity |

100.0% |

Unbounded Flux In Default Medium |

58.0% |

---

Sub Total |

93% |

X3

The Sub Total is the result of the following calculation. For more information please click on "Readme" in the top left of the report.

95.61+58.04+(3⋅100)+(5⋅100)=93.3895.61+58.04+(3⋅100)+(5⋅100)=93.38

## Annotation - Metabolites

Presence of Metabolite Annotation |

99.9% |

Metabolite Annotations Per Database | Info |

 pubchem.compound |

0.0% |

 kegg.compound |

78.5% |

 seed.compound |

99.9% |

 inchikey |

71.1% |

 inchi |

71.1% |

 chebi |

0.0% |

 hmdb |

0.0% |

 reactome |

0.0% |

 metanetx.chemical |

98.9% |

 bigg.metabolite |

65.6% |

 biocyc |

67.4% |

Metabolite Annotation Conformity Per Database | Info |

 pubchem.compound |

0.0% |

 kegg.compound |

96.0% |

 seed.compound |

99.9% |

 inchikey |

100.0% |

 inchi |

100.0% |

 chebi |

0.0% |

 hmdb |

0.0% |

 reactome |

0.0% |

 metanetx.chemical |

99.9% |

 bigg.metabolite |

100.0% |

 biocyc |

99.6% |

Uniform Metabolite Identifier Namespace |

100.0% |

---

Sub Total |

78% |

The Sub Total is the result of the following calculation. For more information please click on "Readme" in the top left of the report.

99.94+78.48+99.94+71.05+71.05+98.87+65.64+67.40+95.99+99.94+99.94+99.63+(4⋅100)+(8⋅0)(24⋅100)=78.3599.94+78.48+99.94+71.05+71.05+98.87+65.64+67.40+95.99+99.94+99.94+99.63+(4⋅100)+(8⋅0)(24⋅100)=78.35

## Annotation - Reactions

Presence of Reaction Annotation |

99.8% |

Reaction Annotations Per Database | Info |

 rhea |

46.4% |

 kegg.reaction |

62.5% |

 seed.reaction |

92.5% |

 metanetx.reaction |

90.9% |

 bigg.reaction |

53.5% |

 reactome |

0.0% |

 ec-code |

82.6% |

 brenda |

0.0% |

 biocyc |

48.0% |

Reaction Annotation Conformity Per Database | Info |

 rhea |

100.0% |

 kegg.reaction |

99.9% |

 seed.reaction |

99.9% |

 metanetx.reaction |

100.0% |

 bigg.reaction |

98.1% |

 reactome |

0.0% |

 ec-code |

96.9% |

 brenda |

0.0% |

 biocyc |

100.0% |

Uniform Reaction Identifier Namespace |

100.0% |

---

Sub Total |

82% |

The Sub Total is the result of the following calculation. For more information please click on "Readme" in the top left of the report.

99.82+46.42+62.47+92.54+90.93+53.52+82.64+48.03+99.94+99.94+98.09+96.90+(4⋅100)+(4⋅0)(20⋅100)=82.4999.82+46.42+62.47+92.54+90.93+53.52+82.64+48.03+99.94+99.94+98.09+96.90+(4⋅100)+(4⋅0)(20⋅100)=82.49

## Annotation - Genes

Presence of Gene Annotation |

99.6% |

Gene Annotations Per Database | Info |

 refseq |

0.0% |

 uniprot |

0.0% |

 ecogene |

0.0% |

 kegg.genes |

0.0% |

 ncbigi |

64.0% |

 ncbigene |

0.0% |

 ncbiprotein |

0.0% |

 ccds |

0.0% |

 hprd |

0.0% |

 asap |

0.0% |

Gene Annotation Conformity Per Database | Info |

 refseq |

0.0% |

 uniprot |

0.0% |

 ecogene |

0.0% |

 kegg.genes |

0.0% |

 ncbigi |

100.0% |

 ncbigene |

0.0% |

 ncbiprotein |

0.0% |

 ccds |

0.0% |

 hprd |

0.0% |

 asap |

0.0% |

---

Sub Total |

39% |

The Sub Total is the result of the following calculation. For more information please click on "Readme" in the top left of the report.

99.62+64.04+(1⋅100)+(18⋅0)(21⋅100)=38.6899.62+64.04+(1⋅100)+(18⋅0)(21⋅100)=38.68

## Annotation - SBO Terms

Metabolite General SBO Presence |

99.7% |

Metabolite SBO:0000247 Presence |

99.7% |

Reaction General SBO Presence |

99.4% |

Metabolic Reaction SBO:0000176 Presence |

99.6% |

Transport Reaction SBO:0000185 Presence |

98.6% |

Exchange Reaction SBO:0000627 Presence |

98.3% |

Demand Reaction SBO:0000628 Presence |

100.0% |

Sink Reactions SBO:0000632 Presence |

100.0% |

Gene General SBO Presence |

99.6% |

Gene SBO:0000243 Presence |

99.6% |

Biomass Reactions SBO:0000629 Presence |

100.0% |

---

Sub Total |

100% |

X2

The Sub Total is the result of the following calculation. For more information please click on "Readme" in the top left of the report.

99.69+99.69+99.40+99.57+98.62+98.33+99.62+99.62+(3⋅100)+(11⋅100)=99.5099.69+99.69+99.40+99.57+98.62+98.33+99.62+99.62+(3⋅100)+(11⋅100)=99.50

---

---

Total Score |

91% |

The Total Score is the result of the following calculation. For more information please click on "Readme" in the top left of the report.

(3⋅93.38)+(1⋅78.35)+(1⋅82.49)+(1⋅38.68)+(2⋅99.50)(3⋅100)+(1⋅100)+(1⋅100)+(1⋅100)+(2⋅100)=90.92(3⋅93.38)+(1⋅78.35)+(1⋅82.49)+(1⋅38.68)+(2⋅99.50)(3⋅100)+(1⋅100)+(1⋅100)+(1⋅100)+(2⋅100)=90.92

---

Total Score 

91%

Score per Category 

Export

0%10%20%30%40%50%60%70%80%90%100%scoreconsistencyannotation\_metannotation\_rxnannotation\_geneannotation\_sbosection

Specific Section  Covers general statistics and specific aspects of a metabolic network that are not universally applicable. See readme for more details. 

SBML

SBML Level and Version |

Errored |

FBC enabled |

Errored |

Basic Information

Model Identifier |

iMK1321 |

Total Metabolites |

1,589 |

Total Reactions |

1,676 |

Total Genes |

1,321 |

Total Compartments |

2 |

Metabolic Coverage |

1.27 |

Metabolite Information

Unique Metabolites |

1,567 |

Duplicate Metabolites in Identical Compartments |

2 |

Metabolites without Charge |

2 |

Metabolites without Formula |

0 |

Medium Components |

21 |

Reaction Information

Purely Metabolic Reactions |

1,405 |

Purely Metabolic Reactions with Constraints |

62 |

Transport Reactions |

145 |

Transport Reactions with Constraints |

6 |

Thermodynamic Reversibility of Purely Metabolic Reactions |

0.39 |

Reactions With Partially Identical Annotations |

0.00 |

Duplicate Reactions |

0.00 |

Reactions With Identical Genes |

0.68 |

Gene-Protein-Reaction (GPR) Associations

Reactions without GPR |

70 |

Fraction of Transport Reactions without GPR |

0.08 |

Enzyme Complexes |

839 |

Biomass

Biomass Reactions Identified |

1 |

Biomass Consistency |

Errored |

Biomass Production In Default Medium |

0.50 |

Unrealistic Growth Rate In Default Medium |

false |

Biomass Production In Complete Medium |

103.09 |

Blocked Biomass Precursors In Default Medium |

6 |

Blocked Biomass Precursors In Complete Medium |

6 |

Ratio of Direct Metabolites in Biomass Reaction |

0.10 |

Number of Missing Essential Biomass Precursors |

1 |

Energy Metabolism

Non-Growth Associated Maintenance Reaction |

1 |

Growth-associated Maintenance in Biomass Reaction |

false |

Number of Reversible Oxygen-Containing Reactions |

27 |

Erroneous Energy-generating Cycles | Info |

 MNXM3 |

Skipped |

 MNXM63 |

Skipped |

 MNXM51 |

Skipped |

 MNXM121 |

Skipped |

 MNXM423 |

Skipped |

 MNXM6 |

Skipped |

 MNXM10 |

Skipped |

 MNXM38 |

Skipped |

 MNXM208 |

Skipped |

 MNXM191 |

Skipped |

 MNXM223 |

Skipped |

 MNXM7517 |

Skipped |

 MNXM12233 |

Skipped |

 MNXM558 |

Skipped |

 MNXM21 |

Skipped |

 MNXM89557 |

Skipped |

Network Topology

Universally Blocked Reactions |

686 |

Orphan Metabolites |

86 |

Dead-end Metabolites |

63 |

Stoichiometrically Balanced Cycles |

396 |

Metabolite Production In Complete Medium |

771 |

Metabolite Consumption In Complete Medium |

798 |

Matrix Conditioning

Ratio Min/Max Non-Zero Coefficients |

0.00 |

Independent Conservation Relations |

232 |

Rank |

1357 |

Degrees Of Freedom |

319 |

Experimental Data Comparison

Growth Prediction |

Skipped |

Gene Essentiality Prediction |

Skipped |

Misc. Tests

Environment Python Version 3.6.10

 Platform Linux

 Memote Version 0.10.2

Package Versions

{"sqlalchemy":"1.3.16","ruamel.yaml":"0.16.10","pytest":"5.4.1","cobra":"0.17.1","gitpython":"3.1.1","numpydoc":"0.9.2","goodtables":"2.4.16","future":"0.18.2","pandas":"1.0.3","importlib-resources":"1.5.0","click":"7.1.2","click-configfile":"0.2.3","equilibrator-api":"0.1.26","lxml":"4.5.0","six":"1.14.0","depinfo":"1.5.3","requests":"2.23.0","Jinja2":"2.11.2","click-log":"0.3.2","sympy":"1.5.1","travis-encrypt":"1.1.2","pylru":"1.2.0","cookiecutter":"1.7.2","memote":"0.10.2","pip":"20.1","setuptools":"46.1.3","wheel":"0.34.2"}
