# Supplementary material for: A Genome-Scale Metabolic Model of 2,3-Butanediol Production by Thermophilic Bacteria Geobacillus icigianus
Source: Microorganisms. 2020 Jul 4;8(7):1002. doi: 10.3390/microorganisms8071002 (PMC7409357; doi:10.3390/microorganisms8071002)
Supplement: Supplementary file 1 [file microorganisms-08-01002-s001.zip › Geobacillus_icigianus_supplementary/model_files/Memote_results/MemoteReportInitialModel.html]

MemoteReportApp


 model\_CG  Expand All

Readme

2020-04-13 13:09

Independent Section  Contains tests that are independent of the class of modeled organism, a model's complexity or types of identifiers that are used to describe its components. Parameterization or initialization of the network is not required. See readme for more details. 

## Consistency

Stoichiometric Consistency |

100.0% |

X3

Stoichiometric inconsistency violates universal constraints:
1. Molecular masses are always positive, and
2. On each side of a reaction the mass is conserved.
A single incorrectly defined reaction can lead to stoichiometric
inconsistency in the model, and consequently to unconserved metabolites.
Similar to insufficient constraints, this may give rise to cycles which
either produce mass from nothing or consume mass from the model.
Implementation:
This test first uses an implementation of the algorithm presented in
section 3.1 by Gevorgyan, A., M. G Poolman, and D. A Fell.
"Detection of Stoichiometric Inconsistencies in Biomolecular Models."
Bioinformatics 24, no. 19 (2008): 2245.
doi: 10.1093/bioinformatics/btn425
Should the model be inconsistent, then the list of unconserved metabolites
is computed using the algorithm described in section 3.2 of the same
publication.

This model contains 0 (0.00%) unconserved metabolites:

[]

Mass Balance |

100.0% |

Charge Balance |

95.8% |

Metabolite Connectivity |

100.0% |

Unbounded Flux In Default Medium |

60.2% |

---

Sub Total |

94% |

X3

The Sub Total is the result of the following calculation. For more information please click on "Readme" in the top left of the report.

95.79+60.22+(3⋅100)+(5⋅100)=93.7295.79+60.22+(3⋅100)+(5⋅100)=93.72

## Annotation - Metabolites

Presence of Metabolite Annotation |

100.0% |

Metabolite Annotations Per Database | Info |

 pubchem.compound |

0.0% |

 kegg.compound |

78.7% |

 seed.compound |

100.0% |

 inchikey |

71.3% |

 inchi |

71.3% |

 chebi |

0.0% |

 hmdb |

0.0% |

 reactome |

0.0% |

 metanetx.chemical |

99.2% |

 bigg.metabolite |

65.5% |

 biocyc |

67.6% |

Metabolite Annotation Conformity Per Database | Info |

 pubchem.compound |

0.0% |

 kegg.compound |

96.0% |

 seed.compound |

100.0% |

 inchikey |

100.0% |

 inchi |

100.0% |

 chebi |

0.0% |

 hmdb |

0.0% |

 reactome |

0.0% |

 metanetx.chemical |

99.9% |

 bigg.metabolite |

100.0% |

 biocyc |

99.6% |

Uniform Metabolite Identifier Namespace |

100.0% |

---

Sub Total |

78% |

The Sub Total is the result of the following calculation. For more information please click on "Readme" in the top left of the report.

78.74+71.29+71.29+99.18+65.49+67.63+95.99+99.94+99.63+(7⋅100)+(8⋅0)(24⋅100)=78.3978.74+71.29+71.29+99.18+65.49+67.63+95.99+99.94+99.63+(7⋅100)+(8⋅0)(24⋅100)=78.39

## Annotation - Reactions

Presence of Reaction Annotation |

100.0% |

Reaction Annotations Per Database | Info |

 rhea |

46.8% |

 kegg.reaction |

62.9% |

 seed.reaction |

92.7% |

 metanetx.reaction |

91.5% |

 bigg.reaction |

53.4% |

 reactome |

0.0% |

 ec-code |

83.2% |

 brenda |

0.0% |

 biocyc |

48.3% |

Reaction Annotation Conformity Per Database | Info |

 rhea |

100.0% |

 kegg.reaction |

99.9% |

 seed.reaction |

100.0% |

 metanetx.reaction |

100.0% |

 bigg.reaction |

98.2% |

 reactome |

0.0% |

 ec-code |

96.8% |

 brenda |

0.0% |

 biocyc |

100.0% |

Uniform Reaction Identifier Namespace |

100.0% |

---

Sub Total |

83% |

The Sub Total is the result of the following calculation. For more information please click on "Readme" in the top left of the report.

46.76+62.89+92.75+91.49+53.42+83.15+48.32+99.94+98.20+96.82+(6⋅100)+(4⋅0)(20⋅100)=82.6046.76+62.89+92.75+91.49+53.42+83.15+48.32+99.94+98.20+96.82+(6⋅100)+(4⋅0)(20⋅100)=82.60

## Annotation - Genes

Presence of Gene Annotation |

100.0% |

Gene Annotations Per Database | Info |

 refseq |

0.0% |

 uniprot |

0.0% |

 ecogene |

0.0% |

 kegg.genes |

0.0% |

 ncbigi |

64.3% |

 ncbigene |

0.0% |

 ncbiprotein |

0.0% |

 ccds |

0.0% |

 hprd |

0.0% |

 asap |

0.0% |

Gene Annotation Conformity Per Database | Info |

 refseq |

0.0% |

 uniprot |

0.0% |

 ecogene |

0.0% |

 kegg.genes |

0.0% |

 ncbigi |

100.0% |

 ncbigene |

0.0% |

 ncbiprotein |

0.0% |

 ccds |

0.0% |

 hprd |

0.0% |

 asap |

0.0% |

---

Sub Total |

39% |

The Sub Total is the result of the following calculation. For more information please click on "Readme" in the top left of the report.

64.29+(2⋅100)+(18⋅0)(21⋅100)=38.8164.29+(2⋅100)+(18⋅0)(21⋅100)=38.81

## Annotation - SBO Terms

Metabolite General SBO Presence |

100.0% |

Metabolite SBO:0000247 Presence |

100.0% |

Reaction General SBO Presence |

100.0% |

Metabolic Reaction SBO:0000176 Presence |

100.0% |

Transport Reaction SBO:0000185 Presence |

100.0% |

Exchange Reaction SBO:0000627 Presence |

100.0% |

Demand Reaction SBO:0000628 Presence |

100.0% |

Sink Reactions SBO:0000632 Presence |

100.0% |

Gene General SBO Presence |

100.0% |

Gene SBO:0000243 Presence |

100.0% |

Biomass Reactions SBO:0000629 Presence |

100.0% |

---

Sub Total |

100% |

X2

The Sub Total is the result of the following calculation. For more information please click on "Readme" in the top left of the report.

(11⋅100)+(11⋅100)=100.00(11⋅100)+(11⋅100)=100.00

---

---

Total Score |

91% |

The Total Score is the result of the following calculation. For more information please click on "Readme" in the top left of the report.

(3⋅93.72)+(1⋅78.39)+(1⋅82.60)+(1⋅38.81)+(2⋅100.00)(3⋅100)+(1⋅100)+(1⋅100)+(1⋅100)+(2⋅100)=91.27(3⋅93.72)+(1⋅78.39)+(1⋅82.60)+(1⋅38.81)+(2⋅100.00)(3⋅100)+(1⋅100)+(1⋅100)+(1⋅100)+(2⋅100)=91.27

---

Total Score 

91%

Score per Category 

Export

0%10%20%30%40%50%60%70%80%90%100%scoreconsistencyannotation\_metannotation\_rxnannotation\_geneannotation\_sbosection

Specific Section  Covers general statistics and specific aspects of a metabolic network that are not universally applicable. See readme for more details. 

SBML

SBML Level and Version |

Errored |

FBC enabled |

Errored |

Basic Information

Model Identifier |

model\_CG |

Total Metabolites |

1,585 |

Total Reactions |

1,668 |

Total Genes |

1,316 |

Total Compartments |

2 |

Metabolic Coverage |

1.27 |

Metabolite Information

Unique Metabolites |

1,475 |

Duplicate Metabolites in Identical Compartments |

2 |

Metabolites without Charge |

0 |

Metabolites without Formula |

0 |

Medium Components |

118 |

Reaction Information

Purely Metabolic Reactions |

1,401 |

Purely Metabolic Reactions with Constraints |

62 |

Transport Reactions |

143 |

Transport Reactions with Constraints |

6 |

Thermodynamic Reversibility of Purely Metabolic Reactions |

0.39 |

Reactions With Partially Identical Annotations |

0.00 |

Duplicate Reactions |

0.00 |

Reactions With Identical Genes |

0.68 |

Gene-Protein-Reaction (GPR) Associations

Reactions without GPR |

69 |

Fraction of Transport Reactions without GPR |

0.07 |

Enzyme Complexes |

838 |

Biomass

Biomass Reactions Identified |

1 |

Biomass Consistency |

Errored |

Biomass Production In Default Medium |

103.09 |

Unrealistic Growth Rate In Default Medium |

true |

Biomass Production In Complete Medium |

103.09 |

Blocked Biomass Precursors In Default Medium |

6 |

Blocked Biomass Precursors In Complete Medium |

6 |

Ratio of Direct Metabolites in Biomass Reaction |

0.10 |

Number of Missing Essential Biomass Precursors |

1 |

Energy Metabolism

Non-Growth Associated Maintenance Reaction |

1 |

Growth-associated Maintenance in Biomass Reaction |

false |

Number of Reversible Oxygen-Containing Reactions |

28 |

Erroneous Energy-generating Cycles | Info |

 MNXM3 |

Skipped |

 MNXM63 |

Skipped |

 MNXM51 |

Skipped |

 MNXM121 |

Skipped |

 MNXM423 |

Skipped |

 MNXM6 |

Skipped |

 MNXM10 |

Skipped |

 MNXM38 |

Skipped |

 MNXM208 |

Skipped |

 MNXM191 |

Skipped |

 MNXM223 |

Skipped |

 MNXM7517 |

Skipped |

 MNXM12233 |

Skipped |

 MNXM558 |

Skipped |

 MNXM21 |

Skipped |

 MNXM89557 |

Skipped |

Network Topology

Universally Blocked Reactions |

690 |

Orphan Metabolites |

86 |

Dead-end Metabolites |

63 |

Stoichiometrically Balanced Cycles |

397 |

Metabolite Production In Complete Medium |

775 |

Metabolite Consumption In Complete Medium |

805 |

Matrix Conditioning

Ratio Min/Max Non-Zero Coefficients |

0.00 |

Independent Conservation Relations |

234 |

Rank |

1351 |

Degrees Of Freedom |

317 |

Experimental Data Comparison

Growth Prediction |

Skipped |

Gene Essentiality Prediction |

Skipped |

Misc. Tests

Environment Python Version 3.6.10

 Platform Linux

 Memote Version 0.9.12

Package Versions

{"goodtables":"1.0.0","click-configfile":"0.2.3","importlib-resources":"1.0.2","six":"1.12.0","travis-encrypt":"1.1.2","cobra":"0.16.0","gitpython":"3.0.3","numpydoc":"0.9.1","pytest":"5.2.1","future":"0.18.0","lxml":"4.4.1","depinfo":"1.5.1","Jinja2":"2.10.3","click":"7.0","ruamel.yaml":"0.16.5","equilibrator-api":"0.1.26","pylru":"1.2.0","cookiecutter":"1.6.0","pandas":"0.23.4","sqlalchemy":"1.3.10","click-log":"0.3.2","sympy":"1.4","requests":"2.22.0","memote":"0.9.12","pip":"9.0.3","setuptools":"45.2.0","wheel":"0.34.2"}

|  |  |  |  |  |  |  |  |  |  |
| --- | --- | --- | --- | --- | --- | --- | --- | --- | --- |
|  | Определить языкАзербайджанскийАлбанскийАмхарскийАнглийскийАрабскийАрмянскийАфрикаансБаскскийБелорусскийБенгальскийБирманскийБолгарскийБоснийскийВаллийскийВенгерскийВьетнамскийГавайскийГаитянскийГалисийскийГолландскийГреческийГрузинскийГуджаратиДатскийЗулуИвритИгбоИдишИндонезийскийИрландскийИсландскийИспанскийИтальянскийЙорубаКазахскийКаннадаКаталанскийКиргизскийКитайский ТрадКитайский УпрКорейскийКорсиканскийКурманджиКхмерскийКхосаЛаосскийЛатинскийЛатышскийЛитовскийЛюксембургскийМакедонскийМалагасийскийМалайскийМалаяламМальтийскийМаориМаратхиМонгольскийНемецкийНепальскийНорвежскийПанджабиПерсидскийПольскийПортугальскийПуштуРумынскийРусскийСамоанскийСебуанскийСербскийСесотоСингальскийСиндхиСловацкийСловенскийСомалийскийСуахилиСунданскийТаджикскийТайскийТамильскийТелугуТурецкийУзбекскийУкраинскийУрдуФилиппинскийФинскийФранцузскийФризскийХаусаХиндиХмонгХорватскийЧеваЧешскийШведскийШонаШотландский (гэльский)ЭсперантоЭстонскийЯванскийЯпонский |  | АзербайджанскийАлбанскийАмхарскийАнглийскийАрабскийАрмянскийАфрикаансБаскскийБелорусскийБенгальскийБирманскийБолгарскийБоснийскийВаллийскийВенгерскийВьетнамскийГавайскийГаитянскийГалисийскийГолландскийГреческийГрузинскийГуджаратиДатскийЗулуИвритИгбоИдишИндонезийскийИрландскийИсландскийИспанскийИтальянскийЙорубаКазахскийКаннадаКаталанскийКиргизскийКитайский ТрадКитайский УпрКорейскийКорсиканскийКурманджиКхмерскийКхосаЛаосскийЛатинскийЛатышскийЛитовскийЛюксембургскийМакедонскийМалагасийскийМалайскийМалаяламМальтийскийМаориМаратхиМонгольскийНемецкийНепальскийНорвежскийПанджабиПерсидскийПольскийПортугальскийПуштуРумынскийРусскийСамоанскийСебуанскийСербскийСесотоСингальскийСиндхиСловацкийСловенскийСомалийскийСуахилиСунданскийТаджикскийТайскийТамильскийТелугуТурецкийУзбекскийУкраинскийУрдуФилиппинскийФинскийФранцузскийФризскийХаусаХиндиХмонгХорватскийЧеваЧешскийШведскийШонаШотландский (гэльский)ЭсперантоЭстонскийЯванскийЯпонский |  |  |  |  |  |  |

Звуковая функция ограничена 200 символами

|  |  |  |  |
| --- | --- | --- | --- |
|  |  | Настройки : История : Обратная связь : Donate | Закрыть |
